# Proceedings from the 10th Annual Conference on the Science of Dissemination and Implementation: Arlington, VA, USA. 04-06 December 2017

**DOI:** 10.1186/s13012-018-0728-7

**Published:** 2018-03-28

## Introduction: What A Difference a Decade Makes

### A1 David Chambers^1^, Lisa Simpson^2^, Gila Neta^1^

#### ^1^ National Cancer Institute, Rockville, MD, USA; ^2^ AcademyHealth, Washington, DC, USA

In September 2007, the National Institutes of Health (NIH) hosted a one-day conference on its campus to spotlight leading researchers in the developing field of dissemination and implementation science. The conference, attended by several hundred people, was designed to expand interest in a set of NIH funding opportunity announcements that would generate studies to understand and improve upon processes for translating research evidence and transporting evidence-based health interventions into clinical and community practice settings. It was proceeded by a technical assistance workshop where seventy investigators brought concepts for the next set of studies. Beginning the next year, the annual conference expanded into a two-day program, featuring multiple plenary sessions, calls for panel, paper and poster sessions, and expanded networking and opportunities to discuss the challenges in the field.

In December 2017, we commemorated a decade of that journey with over 1200 researchers, practitioners and policymakers convening in the metro DC area for the 10^th^ Annual Conference on the Science of Dissemination and Implementation in Health. As evidence of the growth of this research community and its productivity, this tenth iteration of the conference series expanded to a third day of programming, balancing concurrent paper sessions, hundreds of posters and topical discussions with a keynote speaker and four plenary panels, all reflecting on the growth of the field and the journey ahead. Contained within this supplement are the abstracts reflecting the concurrent sessions and lunchtime discussions, all representing the variety of dissemination and implementation research supported by our conference sponsors, including the NIH, the Agency for Healthcare Research and Quality (AHRQ), the Patient Centered Outcomes Research Institute (PCORI), the Robert Wood Johnson Foundation (RWJF), and the US Department of Veterans Affairs (VA). An additional 391 papers were accepted for poster presentation and these can be viewed at the conference webapp (https://academyhealth.confex.com/academyhealth/2017di/meetingapp.cgi/ModulePosterSessions/0).

We also celebrated the fourth year of a partnership between the NIH and AcademyHealth in co-hosting the conference and were assisted by a multidisciplinary program planning committee which informed the plenary session development, recruitment of key speakers and the topics for lunchtime discussions. We organized concurrent sessions across ten tracks, with two track leads managing the call for abstract development, review process and selection of thematic sessions. We also convened a scientific advisory panel to advise on the overall conference, particularly highlighting the theme of the decade of progress and future directions to come.

We were pleased to welcome Dr. Sandro Galea, Dean of the School of Public Health at Boston College, who delivered the opening keynote address stressing the importance of understanding the underlying influences on population health and how they affect the targets of what should be implemented and where. Indeed, recognizing the interface between health care and population health is something that the next generation of dissemination and implementation studies will need to concentrate on as health and health care increase in their complexity. The conference featured four plenary panel sessions, two focusing on specific challenges within D&I science and two focusing on changes that have been observed in the past decade. The first two provided multiple perspectives on the generation, synthesis and determination of sufficiency of research evidence for implementation, as well as the potential synergies between health equity studies and D&I science. The latter two discussed a number of emergent areas in the field, including advances in measurement, technology, methods and the rise of learning healthcare systems, as well as a reflection on D&I research and practice within health systems and community settings. All were conducted through a town hall format, and benefitted greatly from the wise comments and probing questions of the audience.

The tracks through which concurrent sessions were organized included Behavioral Health, Big Data and Technology for Dissemination and Implementation Research, Clinical Care Settings (this year separated into two tracks: Patient-level interventions and System-level interventions due to the growth of content in this area), Global Dissemination and Implementation, Promoting Health Equity and Eliminating Disparities, Health Policy Dissemination and Implementation, Prevention and Public Health, and Models, Measures and Methods, along with a new track on Building the Future of D&I Research. The planning committee worked to enable conference participants to follow a consistent theme across the multiple sessions of the conference, although we recognized that many conference participants shared interests across tracks. This supplement is once again organized by these track themes.

The call for abstracts, including individual paper presentations, individual posters and panel presentations, resulted in 701 submissions, spread across the ten thematic tracks. Over one hundred reviewers from multiple disciplines, sectors, settings and career stages devoted their time to ensuring a comprehensive and expert review, and reviews were conducted within each track and coordinated by the track leads. For the final program, 29 oral abstract sessions, 12 panels, and 391 posters were presented over the three-day meeting, in addition to a “poster slam”. Slides for the oral presentations and panels (with the agreement of the authors) were posted on the conference website (http://www.academyhealth.org/events/site/10th-annual-conference-science-dissemination-and-implementation-health) and all abstracts were included on the conference webapp (https://academyhealth.confex.com/academyhealth/2017di/meetingapp.cgi). For the second year in a row, the poster slam allowed presentations that enabled the ten top scoring posters to be presented in rapid succession, sharing their key findings in five minutes each. Attendees at the poster slam were outspoken in their support for this session format.

This supplement has compiled the abstracts for presented papers, panel sessions, and discussion sessions from the 10th Annual Meeting on the Science of Dissemination and Implementation in Health: A Decade of Progress and the Path Forward. We are pleased to have the combined proceedings from the conference together in one volume once again, and look forward to the 11th Annual meeting, scheduled for December 3-5, 2018 in Washington, DC, which we hope will show ever increasing interest in the field and quality of the work underway.

## Discussion Forums

### D1 Advancing de-implementation research

#### Cheryl Anne Boyce^1^, Christian Helfrich^2,3^, Jennifer Curry^1^

##### ^1^ National Heart, Lung, and Blood Institute, Bethesda, MD, USA; ^2^ University of Washington, Seattle, WA, USA; ^3^ VA Puget Sound Health Care System, Seattle, WA, USA

As the dissemination and implementation field continues to develop, new challenges have emerged regarding unproven, ineffective and even harmful practices, resulting in the need to study de-implementation. Various terms have been used to describe de-implementation which complicates our understanding of its role in the implementation science process. Scientists and practitioners engaged in de-implementation engaged in a discussion forum to address de-implementation research, including definitional issues, strategies, models, and interventions that have the potential to reduce the continuation of ineffective or low-value practices.

Participants were challenged to think about the breadth of evidence-based, de-implementation research and to consider strategies with the greatest impact and their insights are summarized below. Recent reviews propose that de-implementation and de-adoption are common terminologies that conceptualize multidisciplinary perspectives on de-implementation. When de-implementation occurs, removal of ineffective, low-value practice is part of the successful implementation and aligns within existing implementation and Knowledge-to-Action frameworks. Psychological, neuroscience and behavioral theories hold great promise for de-implementation research including lessons learned from behavioral economics, intrinsic motivation, and theory of planned behavior.

The priority research recommendations were to examine when successful, rapid, de-implementation occurs and has the greatest impact. For example, after negative effects or harm, de-implementation is often distinct and immediate. Similarly, clinicians may stop treatment immediately after a new evidence-based guideline is released, or there is the withdrawal of the drug from the market. However, the underlying mechanisms for stopping behaviors are different from those responsible for replacing behaviors. The science of behavior change relevant to the beliefs and incentives for de-implementation by providers and organizational systems are complex and understudied. Barriers and facilitators that influence implementation may differ than those for de-implementation, such as financial, organizational, provider characteristics, type of interventions, etc. Multilevel de-implementation strategies across individuals, organizations, and communities also require further research. Notably, innovative technologies using electronic health records demonstrate potential to facilitate data collection of health-care practices and strategies and serve as intervention tools for de-implementation. While de-implementation research remains a challenge, the forum participants endorsed the need for the advancement of de-implementation research to improve healthcare quality and implementation science.

### D2 Enhancing cross-fertilization between organization science and implementation science

#### Michael I. Harrison^1^, Jodi Summers Holtrop^2^, Edmond Ramly^3^

##### ^1^ Agency for Healthcare Research and Quality, Rockville, MD, USA; ^2^ University of Colorado School of Medicine, Aurora, CO, USA; ^3^ University of Wisconsin School of Medicine and Public Health, Madison, WI, USA

Michael Harrison introduced the topic and explained its connection to AHRQ’s focus on the Learning Health System (LHS) as envisioned by the Institute of Medicine (http://www.nationalacademies.org/hmd/Reports/2012/Best-Care-at-Lower-Cost-The-Path-to-Continuously-Learning-Health-Care-in-America.aspx).

A lively discussion followed among around 100 attendees about cross-disciplinary collaboration on research teams among practitioners of Organization Science (OS) and Implementation Science (IS), with particular focus on research on LHSs.

OS applies a wide range of social and behavioral science disciplines, along with applied fields like management studies and information science. Participants described potential contributions of these fields to LHS research; their personal experiences as OS researchers or as collaborators with them; recommendations for steps to enhance LHS research and opportunities for further dialogue and cooperation between OS and IS researchers.

Attendees with limited exposure to OS expressed appreciation for the opportunity to hear about OS journals and data bases; OS and IS networks, forums, and resources that can help them learn more about organizational research on implementation. Attendees with substantial experience in healthcare organizational research expressed interest in creating a new track or didactic session at next year’s D and I meeting. The objective would be to enhance applications of organizational approaches to dissemination, implementation, the LHS, and other forms of delivery system transformation. Nearly all attendees asked to be updated about further possibilities for cross-fertilization between the fields, including creation of a virtual location that would provide resources and alert users to opportunities for further interaction. Meeting summaries prepared by Jodi Holtrop and Edmond Ramly were sent to attendees. These are available from Michael.Harrison@ahrq.hhs.gov. Michael is currently exploring web sites that might be appropriate settings for further OS-IS interchange.

### D3 Implementation science in low- and middle-income countries

#### Rachel Sturke^1^, Sandra Naoom^2^

##### ^1^Fogarty International Center, National Institutes of Health, Bethesda, MD, USA; ^2^Centers for Disease Control and Prevention, Atlanta, GA, USA

There has been a remarkable increase in interest in the field of implementation science and demand for implementation science training for global health and low- and middle-income country (LMIC) researchers continues to outpace supply. The goal of this forum was to serve as an opportunity for researchers and practitioners, policy-makers, and research funders to discuss existing training and education opportunities in implementation science for LMIC researchers, to discuss barriers and facilitators to establishing and accessing training programs, and priorities for training programs. The discussion was framed by the following questions: What are the competencies, metrics for these competencies, and core methodologies and tools being covered in current training programs? Are there key differences for LMIC setting that should be taken into account when framing IS training programs for LMIC researchers? Are there critical gaps in IS capacity in LMICs? What is needed to further build capacity in implementation science in LMICs? How can we cultivate mentors for early stage implementation researchers in LMICs? How can we more effectively support and encourage collaboration and coordination of implementation science capacity building initiatives?

Multiple themes emerged through the discussion. Participants described several existing training opportunities in implementation science for LMIC researchers, ranging from short courses to long-term programs. Participants noted that the field continues to lack a standardized taxonomy for implementation science, including a definition and methodologic components. Participants noted the need for implementation science capacity building for both researchers as well as practitioners and policy-makers, and that training competencies are not well differentiated for multiple end users. Participants noted that there is a lack of critical mass of expertise in implementation science in LMICs, including insufficient mentors and support for mentors. Finally, participants noted that implementation science approaches developed in high-income country settings may not be transferable to the LMIC context and the need to adapt these approaches (including frameworks and models) for the global context.

### D4 NCI listening forum: Multilevel methods and interventions

#### Erica Breslau, Sarah Kobrin

##### National Cancer Institute, Rockville, MD, USA

A goal of the listening forum was to hear from scientists engaged in exploring the progress and challenges of developing, implementing and analyzing the effects of multilevel interventions in healthcare settings. Many multilevel interventions found to effective in healthcare fail to translate into meaningful outcomes across different context, resulting implementation barriers that arise at different levels of healthcare delivery. For this discussion, multilevel interventions were said to be those that address health outcomes at two or more levels of the healthcare environment (i.e., individual, clinician team, healthcare organization or community setting where the healthcare organization is located), and intervene on different people or sets of people in each setting to affect change. A summary of the discussion topics are described below.

What priorities need to be addressed in multilevel methods and interventions? Research that examines how care is provided, who and what touches the patient, care provider, and the system behavior would be valuable so that investigators are able to answer, “when people are diagnosed what are providers actually doing?” Multilevel interventions are particularly needed for considering organizational behavior change (i.e., culture, structure, policies).

What multilevel approaches have you used? Oncology is trying to come up with system level measures that are not simply aggregates of individual level metrics. Parallel work on what is occurring at the provider, and clinic group and hospital level needs to be applied. Traditional measures are focused on one point in time versus quality process measures that examine real time. Stepped-wedge interventions may be considered the gold standard for multilevel interventions. To inform real-time learning, include evaluation as part of the intervention.

What can the NCI do to address these priorities? Conduct training with multiple stakeholders – new applicants, established investigators, reviewers, healthcare staff – so people come in teams to participate in training together. Identify datasets that would help advance multilevel methods. Smaller flexible mechanisms (R21/R33 or interactive R01) that might offer support for both an early and later phase study. Mechanisms that enable development of organizational measures and methods.

### D5 PCORI draft standards for studies of complex interventions: Public comment and discussion

#### Laura Esmail^1^, Brian S. Mittman^2, 3, 4^

##### ^1^ Patient-Centered Outcomes Research Institute, Washington, DC, USA; ^2^ Kaiser Permanente Southern California, Pasadena, CA, USA; ^3^ Department of Veterans Affairs Quality Enhancement Research Initiative, Los Angeles CA, USA; ^4^ Clinical Translational Science Institute, University of California Los Angeles, Los Angeles CA, USA

The PCORI Methodology Standards specify a set of minimal requirements for scientifically valid, transparent, and reproducible comparative effectiveness research. PCORI drafted new standards for studies of complex interventions (SCI), a broad category that includes dissemination and implementation strategies, delivery system interventions, and others. This session provided the opportunity for participants to learn about the new proposed standards, discuss, and provide feedback. Approximately 95 individuals attended the session, which included a 20-minute presentation and a 40-minute discussion period.

The draft standards include: (SCI-1) Fully describe the intervention and comparator and define their core functions; (SCI-2) Specify the hypothesized causal pathways and their theoretical basis; (SCI-3) Specify how adaptations to the form of the intervention and comparator will be allowed and recorded; and (SCI-4) Describe planned data collection and analysis.

Audience feedback addressed a broad range of issues. A key theme of the discussion related to the operationalization of SCI-3 and how to manage adaptations in a research environment. Specific questions addressed how to plan for adaptations *a priori*, how to ensure adequate monitoring and measuring of intervention fidelity, and how to manage the complexity of adaptations and the risks of making inferences regarding the value and/or feasibility of some adaptations over others based upon study results. Participants suggested that determining the core functions of the study intervention is a key first step to planning study adaptations and designing a data collection approach to track fidelity. If the study intervention is linked to core functions, then adaptations can be planned for *a priori* based upon these core functions. This approach also allows fidelity vs. adaptation to be monitored and assessed according to core functions.

The standards for studies of complex interventions are expected to have significant implications for study design, conduct and reporting of complex interventions and provide guidance for examining intervention mediators, moderators and mechanisms of action and adaptation to local contexts.

### D6 Stakeholder engagement and partnership building: best practices and lessons learned

#### Paul Wallace

##### AcademyHealth, Washington, DC, USA

In today’s environment, effective D & I Science requires robust skills in engaging stakeholders and developing new/different partnerships, with communities, patients, healthcare delivery systems, policymakers and others. A forum was conducted utilizing audience response software tools and facilitated discussion to assess audience experience, perspectives and priorities.

The audience, comprising a convenience sample of approximately 100 conference participants who self-selected this session, included a majority that identified themselves as researchers (46%) or clinicians (16%), with other perspectives present including research funders (10%), health system representatives (5%), policy makers, industry representatives and patient/caregiver advocates (all <5%) and 13% ‘others’.

When asked to provide single terms to associate with stakeholder engagement, the most common responses were ‘challenging’, ‘collaboration’, ‘essential’, ‘time and time-consuming’, ‘partnership’ and ‘important’. Discussion highlighted the tension between potential efficiencies and even synergies through partnerships and the complexity of sustaining shared commitments among stakeholders.

Participants were then asked about their own experiences with several specific stakeholder engagement opportunities while planning a study, conducting the study and disseminating study findings. For instance, study planning can involve stakeholders in protocol drafting, participant recruitment and data collection among other activities. Findings:

**Table:** 

Participant Experience	Engaging Stakeholders in one key activity	Engaging Stakeholders in more than one key activity	No experience engaging stakeholders
Planning the Study (91 responses)	24%	41%	35%
Conducting the Study (82 responses)	30%	43%	27%
Dissemination Of Findings (74 responses)	21%	55%	24%

As single terms to best reflect the dominant challenges to stakeholder involvement, the most frequent audience response was ‘time’ (23 responses from 23 unique participants of 157 submitted terms); discussion focused on time availability for both researchers and for other stakeholders. Other challenges offered by multiple audience members included ‘money’, ‘turnover’, ‘resources’ and ‘priorities’.

Finally, when asked for the highest priority for further investments to enhance stakeholder engagement, participants favored: Shared leadership strategies (39%), Communication strategies (36%), Researcher training of (13%), Stakeholders training (6%) and Stakeholder financial incentives (6%). Participants described experiences with leadership development and efforts to better communicate study intent, process and findings within collaborating stakeholder constituencies.

### D7 Using implementation science to improve health equity and reduce disparities

#### LeShawndra N. Price^1^, Suzanne Heurtin-Roberts^2^, Melissa C. Green Parker^1^, Dara R. Blachman-Demner^3^, Helen H. Cox^1^

##### ^1^ National Heart, Lung, and Blood Institute, Bethesda, MD, USA; ^2^ National Cancer Institute, Rockville, MD, USA; ^3^ Office of Behavioral and Social Science Research, National Institutes of Health, Bethesda, MD, USA

To date, there has been little exploration of the possible roles implementation science might play in improving health equity and reducing health disparities. This forum provided an opportunity to discuss approaches and leverage points for implementing evidence-based interventions and practices to promote health equity, as well as highlighted the use of community-engaged research to determine optimal and sustainable strategies for delivering evidence-based interventions to reduce disparities. After “health equity” and “health disparities” were defined (via Healthy People 2020), the following questions stimulated a robust discussion: What essential components, approaches or characteristics of successful implementation science are needed to improve health equity and reduce disparities? How can power differentials among stakeholders be considered in implementation trials? How might implementation frameworks facilitate or hinder conceptualization and design of studies to improve health equity? If elements of the existing implementation science framework lead to or exacerbate health disparities, can, or should, the field of implementation science seek to modify or eliminate these elements? Does Implementation Science that seeks to improve health equity and reduce disparities look any different from the Implementation Science in which researchers usually engage?

Forum participants considered the social determinants of health and an understanding of implementation contexts to be critical to implementation science contributions to building health equity. The need for better, more meaningful measures of health equity was also considered important. Participants suggested partnerships among different funding sources might improve implementation research sustainment and long-term success. Therefore, increasing collaborative funding opportunities for implementation projects focused on health equity was advised. Participants indicated that success among health equity projects is more likely when leveraging long-term relationships among researchers and stakeholders. Participants noted the importance of thorough stakeholder engagement in implementation research and power-sharing among *all* study participants. Additionally, problems with funding and sustainability after an implementation research project ends were mentioned as areas where disparities could possibly be exacerbated. Given the broad interest and limited time in the session, forum leaders look forward to expanding this discussion to inform novel efforts that bring implementation science to bear in addressing future health equity concerns.

## Behavioral Health

### S1 Long-term sustainment of an evidence-based healthy weight intervention in community mental health programs: challenges and opportunities

#### Gail Daumit^1^, Sarah Linden^1^, Elizabeth Stone^1^, Joseph Gennusa^1^, Alene Kennedy-Hendricks^2^, Stacy Goldsholl^1^, Arlene Dalcin^1^, Gerald Jerome^3^, Anna Duval^1^, Emma McGinty^2^

##### ^1^ Johns Hopkins University School of Medicine, Baltimore, MD, USA; ^2^ Johns Hopkins Bloomberg School of Public Health, Baltimore, MD, USA; ^3^ Towson University, Towson, MD, USA

###### **Correspondence:** Gail Daumit (gdaumit@jhmi.edu)

**Background:** Understanding how to sustain effective physical health interventions in mental health settings is critical to narrowing the mortality gap in serious mental illness (SMI). We assessed 5-year sustainment strategies, barriers, and facilitators for the ACHIEVE behavioral weight loss intervention. ACHIEVE resulted in clinically significant weight loss and included group and individual weight-management and group exercise sessions delivered by study interventionists. Mental health program staff led some video-assisted exercise sessions.

**Methods:** We interviewed 27 leaders and staff involved in diet and physical activity programming at seven psychiatric rehabilitation programs (PRPs) participating in the ACHIEVE trial, 5 years after study completion. We analyzed interviews using hybrid inductive/deductive coding approach. A priori codes were guided by published sustainability frameworks.

**Findings:** While all programs reported having some group exercise or healthy diet/weight management groups scheduled at least weekly, use of ACHIEVE exercise videos was the only element with fidelity to the original intervention. Programming did not follow evidence-based protocols or track consumer outcomes. PRP leaders and staff mentioned several barriers to continuing ACHIEVE consistent with prior research including staff turnover, funding and organizational capacity. The theme of mental health consumer engagement also emerged as a barrier. One leader said “…..it (ACHIEVE) kind of died down because of client’s (lack of) participation.” Interviews highlighted factors that could facilitate ACHIEVE sustainment, including organizational fit. In particular, leaders mentioned that ACHIEVE prompted a shift in organizational mission and culture toward physical health.. One said “….maybe if we brought back….the ACHIEVE protocol, maybe it would stick this time because all of us think differently about integrated heath and wellness for our clients now than we did (the) first time. It was a big new thing then and now it’s like, ‘Oh, yeah, this is what we do;”. Sites specifically asked for staff training in weight management and exercise.

**Implications for D&I Research:** Five years after implementing ACHIEVE in a clinical trial, mental health programs have cultures more supportive of improving physical health. However, they need additional implementation support, particularly support engaging consumers in healthy lifestyle behaviors. Development of implementation interventions addressing this and other barriers identified is a priority.

**Primary Funding Source:** National Institutes of Health

### S2 Tailored versus standardized implementation of measurement based care for depression in community mental health

#### Cara Lewis^1,2,3^, C. Nathan Marti^4^, Kelli Scott^2^, Elena Navarro^2^, Meredith Boyd^2^

##### ^1^ MacColl Center for Health Care Innovation, Kaiser Permanente Washington Health Research Institute, Seattle, WA, USA; ^2^ Indiana University, Bloomington, IN, USA; ^3^ University of Washington School of Medicine, Seattle, WA, USA; ^4^ Abacist Analytics, Austin, TX, USA

###### **Correspondence:** Cara Lewis (lewis.cc@ghc.org)

**Background:** A Cochrane review indicated variable impact of tailored implementation methods over standardized approaches in healthcare settings^1^. Optimal methods to identify determinants and inform tailoring remain unknown. This study compared standardized versus tailored implementation of measurement based care (MBC) on clinician-level (fidelity) and client-level (depression severity) outcomes in community behavioral health settings. MBC is an evidence-based practice that involves the use of standardized assessment (i.e., PHQ-9^2^) to guide psychotherapy practice^3^ and is used by fewer than 20% of providers in the United States.

**Methods:** This was a hybrid type II dynamic cluster randomized trial.^4^ The standardized condition included training, consultation, a clinical guideline, and electronic health record enhancements. The tailored condition began with a needs assessment, followed by tailored training based on rapid ethnography, the formation of an in-house implementation team, a clinic-specific guideline, and the use of fidelity data to inform implementation team strategy selection. Clinicians (*N*=164, *M*=42.6 years, *SD*=12.5; 79.2% female) and clients (*N*=507; 69.0% female; PHQ-9 *M=*17.43) from 12 clinics comprised the study participants. MBC fidelity was the primary clinician-level outcome scored on a 4-point scale: 0=PHQ-9 not administered; 1=administration of PHQ-9, 2=review of PHQ-9scores, and 3=discussion of PHQ-9 with clients. PHQ-9 scores at 12 weeks into treatment served as the primary client-level outcome; higher scores indicated more severe depression.

**Findings:** Preliminary mixed multilevel logistic regression models (sessions nested within clients, nested within clinicians, nested within site) revealed superior fidelity outcomes in the tailored condition but only for PHQ-9 completion (OR = 2.86); no differences were observed across conditions regarding review of PHQ-9’s or PHQ-9 discussion in session. However, no difference was observed between conditions on change in client PHQ-9 scores from baseline to week 12 of treatment.

**Implications for D&I Research:** These preliminary results suggest that this particular tailoring methodology holds promise for enhancing use of evidence based practices, but that the minimal behavior change observed may not be sufficient to influence client outcomes.

**Primary Funding Source:** National Institutes of Health

### S3 Improving institutional and provider sustainability of an evidence-based psychosocial intervention in pediatric oncology

#### Demetria McNeal^1^, OJ Sahler^2^, Robert Noll^3^, Diane Fairclough^1,4^, Megan Voll^3^, Elaine Morrato^1,5^

##### ^1^ ACCORDS, University of Colorado Anschutz Medical Campus, Aurora, CO, USA; ^2^ University of Rochester, Rochester, NY, USA; ^3^ University of Pittsburgh, Pittsburgh, PA, USA; University of Colorado School of Medicine, Aurora, CO, USA; ^5^ School of Public Health, University of Colorado Anschutz Medical Campus, Aurora, CO, USA

###### **Correspondence:** Demetria McNeal (demetria.mcneal@ucdenver.edu)

**Background**: Bright IDEAS Problem-Solving Skills Training (PSST), a psychosocial intervention to improve mental well-being for caregivers of children diagnosed with cancer, is a National Cancer Institute Research-Tested Intervention Program. An R25 training program was disseminated nationally in conjunction with professional association conferences and implemented to foster Bright IDEAS adoption among pediatric oncology psychosocial providers (PSPs). After 2 years, 196 PSPs have been trained reaching 49% of the U.S. childhood cancer centers (107/220). Despite encouraging rates for provider interest and institutional reach, factors affecting program sustainability post-training are unknown.

**Methods**: Semi-structured in-depth interviews (averaging 45 minutes) were conducted with 20 randomly selected PSPs from among 122 Bright IDEAS trainees with >12 months of post-training experience. Additionally, a 60-minute focus group was conducted with 6 PSPs who were selected as expert institutional trainers (having completed a train-the-trainer session). Domains of interest were Bright IDEAS adoption, local implementation experience, and program sustainability using Diffusion of Innovation Theory and RE-AIM as frameworks. Interviews were transcribed and data analysis was conducted using ATLAS.ti.

**Findings**: At the provider level, varying perspectives were expressed regarding capacity to incorporate Bright IDEAS into clinical workflow. Some PSPs found Bright IDEAS “more successful” when a target patient profile was established as a mechanism to select appropriate clients– for example: “clients experiencing a specific behavior-based problem”. Comparatively, some PSPs perceived Bright IDEAS as a challenging and even burdensome to translate into clinical practice – for example: “It is hard to use the step by step rigid protocol”. PSPs stated they practiced as solo psychosocial practitioners within multi-disciplinary clinical teams that defer to PSP judgement on best practices. PSPs stated institutional sustainability will require sustained training support until Bright IDEAS becomes a normative expectation.

**Implications for D&I Research:** Provider sustainability requires integration of research-tested protocols into routine clinical workflow. Institutional sustainability needs tailored strategies to support dynamic clinical settings across institutions. Longitudinal follow-up post training is imperative to ensuring the sustainability of an intervention; otherwise limited maintenance should be anticipated.

**Funding:** This study is supported by NIH Grant R01 CA 159013 and R25 CA65520

### S4 Implementation and effectiveness of a secondary risk screener for intimate partner violence (IPV): evidence to inform IPV screening practices in integrated care settings

#### Katherine Iverson^1,2^, Melissa Dichter^3,4^

##### ^1^ National Center for PTSD, VA Boston Healthcare System, Boston, MA, USA; ^2^ Boston University, Boston, MA, USA; ^3^ Center for Health Equity Research and Promotion, Department of Veterans Affairs, Philadelphia, PA, USA; ^4^ University of Pennsylvania Perelman School of Medicine, Philadelphia, PA, USA

###### **Correspondence:** Katherine Iverson (katherine.iverson@va.gov)

**Background:** Women who experience intimate partner violence (IPV) are among the most vulnerable patients seen in primary care. Veterans Health Administration (VHA) recommends annual screening for IPV as a clinical reminder to facilitate disclosure and connection with services. As per VHA recommendations, women who screen positive for IPV should receive secondary screening for risk of further severe violence. This secondary screening is intended to help clinicians gauge women’s current danger levels and expedite linkages to psychosocial care for those who screen positive on the risk assessment. This study aims to evaluate the adoption and penetration of this secondary screener and explore service outcomes.

**Methods:** We apply Proctor et al.’s (2011) evaluation framework within a hybrid investigation of key implementation and service outcomes associated with VHA’s recommendation to conduct secondary screening among IPV+ women. We extracted clinical records data to examine IPV primary and secondary screening records and timing and quantity of VHA psychosocial healthcare utilization among a sample of 774 IPV+ women screened at 11 facilities nationwide. We examined adoption (i.e., proportion of facilities administering secondary screening within the medical records) and penetration (i.e., proportion of eligible patients who received secondary screening at adopting sites). We also compared timeliness and quantity of subsequent psychosocial visits among women who were positive and negative on the secondary screener.

**Findings:** Only 3 of 11 facilities that implemented IPV screening programs adopted the secondary screener into routine care. At sites that implemented primary IPV screening, only 24% of IPV+ women received the secondary screener, suggesting low penetration. There were no differences among women who screened positive versus negative on the secondary screener in the timeliness of subsequent psychosocial visits (median of 6 days vs. 9 days, respectively, *ns*). Similarly, secondary screening results were not associated with quantity of subsequent psychosocial visits.

**Implications for D&I Research:** This is one of the first studies to apply an implementation evaluation framework to investigate IPV screening and response recommendations within a large, integrated healthcare system. The low adoption and penetration of the secondary risk screening, along with a lack of clinical service benefits, indicate a need to modify or de-implement secondary screening from clinical recommendations.

**Primary Funding Source:** Implementation Research Institute (IRI) Fellowship, NIMH 5R25MH08091607 (PI: Proctor) and VA HSR&D and QUERI

### S5 A decade of effectiveness-implementation hybrid trial roll-out and evaluation targeting sustainable behavioral interventions in acute care medical settings

#### Douglas Zatzick^1^, Doyanne Darnell^1^, Lawrence Palinkas^2^

##### ^1^ University of Washington, Seattle, WA, USA; ^2^ University of Southern California, Los Angeles, CA, USA

###### **Correspondence:** Douglas Zatzick (dzatzick@uw.edu)

**Background:** The Disseminating Organizational Screening and Brief Intervention Services (DO-SBIS) pragmatic trial simultaneously aimed to establish the effectiveness of feasibly scaled, high quality alcohol screening and brief intervention, while targeting expansion of American College of Surgeons’ (ACS) policy for the implementation of alcohol screening and intervention at trauma centers nationwide.

**Methods:** The investigation was a pragmatic cluster randomized trial, conducted between 2007-2013, in which 20 US trauma centers were randomized to usual care control (n=10) and intervention (n=10) conditions (patient N=878). Trauma center providers at intervention sites received a one day workshop training, and six months of feedback and coaching in motivational interviewing targeting alcohol consumption. All patients were followed-up at 6- and 12- months post-injury and revaluated to assess alcohol consumption. Multiple pragmatic trial design considerations were incorporated that facilitated rapid translation of trial results into policy, including readily implementable patient alcohol medical record phenotyping and screening, minimal patient exclusions, and intervention implementation by front-line trauma center providers with minimal behavioral health expertise. An ACS policy summit was scheduled in the final years of the trial to facilitate sustainable implementation of results derived from trial effectiveness findings. From 2014-2017, national trauma center surveys and Rapid Assessment Procedures were used to evaluate progress towards nationwide implementation.

**Findings:** Patients at intervention trauma centers demonstrated a statistically and clinically significant 8% reduction in hazardous drinking (Relative Risk=0.88, 95% Confidence Interval=0.79, 0.98). The policy summit successfully incorporated trial findings and informed nationwide requirement for alcohol screening and intervention. Survey data documented that prior to the requirement 79% of Level I trauma centers screened patients for alcohol versus 90% after the requirement. Before the requirement 41% of centers delivered evidence-informed intervention versus 65% after the requirement. Rapid Assessment Procedures identified marked variability in fidelity to high quality procedures in the wake of the requirement.

**Implications for D&I Research:** Effectiveness-Implementation hybrid trials can simultaneously target treatment outcomes and nationwide policy requirements. Regulatory implementation mechanisms successfully draw site level attention to the delivery of evidence-based practices but additional investigative and policy focus may be required to ensure the roll-out of high quality behavioral health screening and intervention procedures.

**Primary Funding Source:** National Institutes of Health

### S6 Clinician fidelity to suicide screening in the emergency department: results from the ED-safe study

#### Edwin Boudreaux^1^, Celine Larkin^1^, Jeffrey Caterino^2^, Ivan Miller^3^, Amy Goldstein^4^, Alexandra Morena^1^, Barbara Stanley^5^, Anne Manton^6^, Carlos Camargo^7^

##### ^1^ University of Massachusetts Medical School, Worcester, MA, USA; ^2^ Ohio State University, Columbus, OH, USA; ^3^ Clinical Psychology, Butler Hospital, Providence, RI, USA; ^4^ National Institute of Mental Health, Bethesda, MD, USA; ^5^ Columbia University, New York, NY, USA; ^6^ Journal of Emergency Nursing, Bourne, MA, USA; ^7^ Emergency Medicine Network, Massachusetts General Hospital, Boston, MA, USA

###### **Correspondence:** Celine Larkin (celine.larkin@umassmed.edu)

**Background:** Universal suicide screening in emergency departments is a recommended best practice, and was implemented in eight EDs as part of the ED-SAFE study. Fidelity to suicide screening was defined as a patient endorsing being asked a screening question that was documented in the medical chart as having been asked. We examine the pattern of screening fidelity across study phases and sites, and its association with patient characteristics.

**Methods:** The three-item Patient Safety Screener was implemented using strategies of staff training, audit and feedback, and performance improvement. Screening documentation increased significantly across the three time-points of ED-SAFE. To assess screening fidelity, we conducted 1194 unique patient interviews at each site at two time-points. Within hours of their presentation, patients with chart documentation of a negative screen were asked to recall whether they were asked each PSS-3 item: (1) depression, (2) suicidal ideation, and (3) suicide attempt. We used chi^2^ and Mann Whitney U tests to examine the associations of site, phase, and patient demographics on screening fidelity.

**Findings:** Patients who reported not being asked about depression were unlikely to be asked about suicidal ideation (55/426,13%) and were never asked about suicide attempt (0/371,0%). Screening fidelity varied significantly by study site (p<0.001), and fidelity to all three items increased significantly over time from 49.0% to 58.8% (chi^2^=11.54,p<0.005). There were no significant effect of sex, but younger patients were more likely to be asked about ideation (U= 144091.5,p=0.002) and attempt (U=162838.5,p=0.02). White patients were less likely than others to report being asked about depression (61.9% versus 68.8%, chi^2^=5.69,p=0.02) and ideation (63.6% versus 69.3%, chi^2^= 3.95,p=0.047), and Hispanic patients were more often asked about ideation (73.5% versus 64.0%, chi^2^=5.46,p=0.02). Patients with post-secondary education were more likely to be asked about depression (67.9% versus 39.0%, chi^2^=4.18,p=0.04).

**Implications for D&I Research:** Patient age, race, ethnicity, and education were significantly associated with screening fidelity, and we observed a clear drop-off in fidelity for the third (suicide attempt) item. Approaching patients soon after they presented likely minimized recall bias. This study may be the first to examine the effect of patient-level factors on screening fidelity.

**Primary Funding Source:** National Institutes of Health

### S7 Testing three strategies for implementing motivational interviewing on medical inpatient units: see one; do one; order one

#### Steve Martino^1,2^, Kimberly Yonkers^2^

##### ^1^ Veterans Health Administration, West Haven, CT, USA; ^2^ Yale University School of Medicine, New Haven, CT, USA

###### **Correspondence:** Steve Martino (steve.martino@yale.edu)

**Background:** General medical hospitals provide care for a disproportionate share of patients who misuse substances. Effective strategies to implement a motivational interviewing-based (MI) brief intervention for risky substance use on medical inpatient units are needed. Implementation frameworks suggest using strategies that are simple and compatible with existing practices of busy medical providers. This study examined the effectiveness of three strategies for increasing medical providers’ use of MI within a general medical hospitalist service. The strategies were: 1) a continuing medical education workshop that “shows” providers how to conduct MI (the control condition, called See One); 2) a “see one, do one” apprenticeship model involving workshop training plus live supervision of bedside practice (Do One); and 3) after learning about MI in a workshop, ordering it for the psychiatry consultation-liaison (CL) service clinical staff to conduct with the patient (Order One).

**Methods:** This Hybrid Type 3 effectiveness-implementation trial included providers (physicians, physician assistants, nurses) randomized to conditions and then assessed for their provision of MI to 40 inpatients. Primary outcomes were 1) percentage of MI sessions conducted among each provider’s consecutively enrolled study patients, 2) independently rated provider MI adherence and competence, and 3) percentage of adequately performed sessions. Secondary outcomes were 1) independently rated strength and frequency of patient statements that favor (change talk) or disfavor change (sustain talk), and 2) themes related to implementation facilitators and barriers identified through qualitative interviews.

**Findings:** Thirty-eight providers and 1173 patients participated. Order One providers conducted a significantly higher percentage of MI sessions (22%) than those in See One and Do One conditions (1% and 3%, respectively), with greater adherence and competence and percentage of adequately performed sessions. Process ratings of patients’ statements (under analysis) will be presented. Implementation themes focused on improving the sustainability of MI practice in hospital settings.

**Implications for D&I Research:** Giving inpatient medical providers the opportunity to “order” MI through CL services may increase opportunities for inpatients to receive brief interventions for risky substance use. The integration of behavioral health specialists into general hospitals may be superior to offering behavioral intervention training to medical providers.

**Primary Funding Source:** National Institutes of Health

### S8 Alcohol use disorder pharmacotherapy and treatment in primary care settings: the adapt-PC trial

#### Hildi Hagedorn^1,2^, Randall Brown^3,4^, Michael Dawes^5,6^, Eric Dieperink^2,7^, Donald (Hugh) Myrick^8,9^, Elizabeth Oliva^10^, Todd Wagner^11,12^, Jennifer Wisdom^13^, Alex Harris^10,12^

##### ^1^ Center for Chronic Disease Outcomes Research, Minneapolis VA Medical Center, Minneapolis, MN, USA; ^2^ University of Minnesota School of Medicine, Minneapolis, MN, USA; ^3^ William S. Middleton Memorial Veterans Hospital, Madison, WI, USA; ^4^ University of Wisconsin School of Medicine and Public Health, Madison, WI, USA; ^5^ South Texas VA Health Care System, San Antonio, TX, USA; ^6^ University of Texas Health Science Center of San Antonio, San Antonio, TX, USA; ^7^ Minneapolis VA Medical Center, Minneapolis, MN, USA; ^8^ Medical University of South Carolina, Charleston, SC, USA; ^9^ Center for Drug and Alcohol Problems, Ralph H. Johnson VA Medical Center, Charleston, SC, USA; ^10^ Center for Innovation to Implementation, Palo Alto VA Health Care System, Menlo Park, CA, USA; ^11^ Health Economics Resource Center, Palo Alto VA Health Care System, Menlo Park, CA, USA; ^12^ Stanford University School of Medicine, Palo Alto, CA, USA; ^13^ Graduate School of Public Health and Health Policy, City University of New York, New York, NY, USA

###### **Correspondence:** Hildi Hagedorn (hildi.hagedorn@va.gov)

**Background:** Despite the high prevalence of alcohol use disorders (AUDs), in a given year, only 12.1% of patients meeting AUD diagnostic criteria receive any treatment. Developing options for treatment within primary care (PC) settings is imperative to increase treatment access for patients with AUDs. The objective of this study was to refine, implement and evaluate an intervention to integrate AUD treatment options, particularly pharmacological options, into PC settings.

**Methods:** Three large Veterans Health Administration facilities participated in the intervention. Local substance use disorders providers and PC/mental health integration providers were trained to serve as implementation/clinical champions and received ongoing external facilitation. PC providers received access to consultation from clinical champions, educational materials, and a dashboard of patients with AUD for case identification. Veterans with AUD diagnoses received educational information in the mail. The mixed methods evaluation included an interrupted time series with matched controls to monitor change in facility-level AUD pharmacotherapy prescribing rates and qualitative interviewing of Veterans, champions and providers analyzed using CFIR constructs.

**Findings:** The odds of a patient receiving AUD pharmacotherapy increased significantly from 3.8% pre-implementation to 5.2% at the end of the implementation period. The overall intervention effect was not significantly different from the increase in control sites. However, results varied by site with Site 1 showing a significant increase, Site 2 showing a non-significant increase and Site 3 showing no change. While all sites experienced substantial implementation barriers, Sites 1 and 3 experienced lower levels of provider support for the intervention compared to Site 2 and Site 3 experienced lower levels of institutional support for the champion role compared to Sites 1 and 2. This suggests that an enthusiastic, well-supported champion may overcome low initial provider support, but missing both of these key ingredients undermines implementation.

**Implications for D&I Research:** This intervention provides a replicable, feasible and relatively low-cost method for integrating AUD treatment into PC settings. This strategy may only be effective with more “ideal” sites with provider support and adequately resourced champions. More focus on enhancing provider buy-in and negotiating dedicated time for champions may be necessary for less “ideal” sites.

**Primary Funding Source:** Department of Veterans Affairs

### S9 Implementation & sustainment facilitation as an effective adjunct to the addiction technology transfer center strategy

#### Bryan Garner^1^, Bryan Garner^2^

##### ^1^RTI International, O'Fallon, IL, USA; ^2^RTI International, Research Triangle Park, NC, USA

###### **Correspondence:** Bryan Garner (bgarner@rti.org)

**Background:** Given substance use among individuals living with HIV/AIDS is both prevalent and problematic, improving the integration of substance use treatment within AIDS service organizations is needed. The Substance Abuse Treatment to HIV care (SAT2HIV) Project was funded to experimentally test the effectiveness of an organization-focused strategy called Implementation & Sustainment Facilitation (ISF) as an adjunct to the current state-of-the-art Addiction Technology Transfer Center (ATTC) strategy. The current presentation present results of this cluster randomized implementation experiment.

**Methods:** Within the context of a Type 2 Implementation-Effectiveness Hybrid Trial, 25 AIDS service organizations and two brief intervention (BI) staff per ASO (N = 50) were randomized to either receive 1) the ATTC strategy (ATTC only) or 2) the ATTC strategy plus the ISF strategy (ATTC+ISF). Implementation effectiveness (i.e., consistency and quality of implementation) during the 6-month implementation phase was the primary outcome measure of interest. The evidence-based treatment being implementation was a motivational interviewing-based brief intervention.

**Findings:** Adjusted multilevel regression analyses, which adjusted for several BI staff characteristics (i.e., age, gender, race, ethnicity, education, work experience, hours worked per week, motivational interviewing experience, and perceived innovation-values fit), supported the effectiveness of the ISF strategy as an effective adjunct to the ATTC strategy (β = 2.36, *p* = .003).

**Implications for D&I Research:** Based on findings from the current cluster randomized experiment, the ISF strategy is an effective adjunct to the ATTC’s current state-of-the-art implementation strategy. The current finding is important given that it suggests ISF as a promising strategy to improve the integration of substance use treatment within AIDS service organizations. This finding is also of importance given that the ISF intervention may hold promise for helping implement other evidence-based treatments within AIDS Service Organizations or for helping implementing other evidence-based treatments within other setting types.

**Primary Funding Source:** National Institutes of Health

### S10 Improving implementation of psychological interventions to older adult cancer patients: convening older adults, caregivers, providers, researchers

#### Kelly Trevino (ket2017@med.cornell.edu)

##### Weill Cornell Medicine, New York, NY, USA

**Background:** Older adults (≥ 65 years) comprise 56% of new cancer diagnoses. Over 40% of older adults with cancer (OACs) report elevated distress that is associated with poor treatment response and greater healthcare utilization. Psychotropic medications are commonly employed but often inappropriate in this population. While psychological interventions are efficacious, safe, and cost-effective, over half of cancer patients with a psychiatric diagnosis do not receive mental health care. The aims of the project were to: 1) identify the barriers to implementation of evidence-based psychological interventions for OACs and 2) develop strategies for intervention implementation.

**Methods:** Thirty-five stakeholders (OACs, caregivers, mental health and oncology providers, and researchers) attended a one-day conference informed by an evidence-based approach that brings together key stakeholders to gain consensus on a topic. Stakeholders discussed barriers to and strategies for implementation of evidence-based psychological interventions to OACs. Qualitative analyses were conducted to identify themes. Stakeholders were sent an electronic survey assessing the impact of each barrier and strategy (1=“not at all;” 5=“a great deal”).

**Findings:** Three themes regarding barriers emerged: 1) OAC factors (e.g., stigma toward psychological services), 2) lack of OAC and provider knowledge regarding psychological services, and 3) facility factors (e.g., lack of funding). Barriers rated as having the most impact included limited patient resources (e.g., finances, support), medical team factors (e.g., lack of training in mental health), poor coordination between oncology and mental health, and stigma toward mental health. Three thematic implementation strategies emerged: 1) tailoring interventions for OACs; 2) increasing awareness of psychological interventions; and 3) integrating psychological interventions into oncology care. Strategies rated as having the most impact included modifications to intervention delivery (e.g., leveraging community organizations), improved coordination of oncologic and mental health treatment, and provision of psychological services information to institutional leaders.

**Implications for D&I Research:** OACs with cancer have high levels of untreated distress despite the presence of efficacious psychological interventions. This study addresses the gap between efficacy research and OAC access to psychological interventions. The barriers and strategies identified by diverse stakeholders provide guidance for the development of implementation strategies that can be tested in future studies to improve OAC access to evidence-based psychological interventions.

**Primary Funding Source:** American Federation for Aging Research

### S11 A perinatal depression decision aid: end-user involvement in the development, design, and pilot testing, for a large scale, statewide dissemination

#### Lee Anne Roman^1,2^, Jennifer Raffo^3^, McKenna Rubalcaba-Williamson^1^, Susan Henning^4^, Melinda Johnson^4^, Margaret Holmes-Rovner^1^

##### ^1^ Michigan State University, East Lansing, MI, USA; ^2^ Spectrum Health, Grand Rapids, MI, USA; ^3^ Michigan State University, Grand Rapids, MI, USA; ^4^ Spectrum Health, Grand Rapids, MI, USA

###### **Correspondence:** Lee Anne Roman (lroman@msu.edu)

**Background:** Maternal depression, common in one in five Medicaid-insured pregnant and postpartum women, has substantial health impacts and depression-related health care and work productivity costs. State-sponsored Medicaid Enhanced Prenatal Care (EPC) and home visiting programs require screening, are well-established, and have broad reach in the population at risk. In Michigan, EPC screens over 16,000/year pregnant women; however, less than a third of women with a positive screen initiate treatment. Given that depression screening has not had the expected impact on mental health care utilization, this paper describes a systematic developmental process, focused on end user involvement, to design and user test a depression Patient Decision Aid (PDA) for use in a statewide, community-based EPC and prenatal care settings.

**Methods:** The following sequential methods were used to develop and pilot test a PDA: 1) Process mapping strategy to identify barriers and opportunities for shared decision making 2) Prototype development followed by end-user structured survey review; 3) Graphic design to address cultural and literacy issues, followed by a focus group of Community Health Workers (CHWs) who reflect the characteristics of the population served; 6) Survey of end users/stakeholders; and 5) Pilot usability testing in real time within the provider-patient dyadic screening and decision making process.

**Findings:** The primary evaluation findings included: 1) Concern of patient readiness and embedding an evidence-based engagement intervention in the decision aid; 2) Addition of a conversation grid to support providers; 3) Sensitivity to words that stigmatize and finding alternatives (e.g. brain chemistry vs. hormones); 4) Addition of “watchful waiting” and “healthy habits” as legitimate treatment options; 5) Challenge of the amount of information for low health literacy patients; the need for action words, non-stigmatizing pictures of women, and a broad description of options.

**Implications for D&I Research:** The process mapping strategy was useful and engaged community stakeholders in the PDA process. The CHW feedback was critical and partnerships with those closest to the population at risk were invaluable. Tailoring to the primary and secondary contexts (home visiting and prenatal care providers) increased demand for the PDA even during the developmental process, with the Medicaid EPC ready for statewide dissemination and implementation testing.

**Primary Funding Source:** Agency for Healthcare Research and Quality

### S12 Choosing wisely: identification and prioritization of the determinants to overuse of advanced medical imaging for distant metastases in early-stage breast cancer

#### Janet Squires^1,2^, Ian Graham^1,2^, Jeremy Grimshaw^1,2^, Stephanie Linklater^2^, Megan Greenough^3^, Kristin Dorrance^2^, Mark Clemons^4^, Demetrios Simos^5^, Dawn Stacey^1^, Jean-Michel Caudrelier^4^, Geoff Doherty^4^, Maureen Trudeau^6^, Catherine Tsilfidis^2^, Angel Arnaout^4^

##### ^1^ University of Ottawa, Ottawa, ON, Canada; ^2^ Ottawa Hospital Research Institute, Ottawa, ON, Canada; ^3^ School of Nursing, University of Ottawa, Ottawa, ON, Canada; ^4^ Ottawa Hospital, Ottawa, ON, Canada; ^5^ Stronach Regional Cancer Centre, Newmarket, ON, Canada; ^6^ Sunnybrook Health Sciences Centre, Toronto, ON, Canada

###### **Correspondence:** Janet Squires (jasquires@ohri.ca)

**Background:** The Choosing Wisely campaign as well as international guidelines recommend against the routine use of advanced medical imaging in women with early stage breast cancer due to the extremely low yield of detection of distant metastasis; the unnecessary harm and distress it can induce on patients from false positive diagnoses; and the significant healthcare costs associated with these tests. The purpose of this presentation is to report on a national study to identify and prioritize the determinants to routine advanced medical imaging use in early stage breast cancer. This is Phase 1 of a larger research program focused on the de-implementation of ineffective practices in cancer care.

**Methods:** Semi-structured interviews were conducted across Canada with clinicians (surgical, medical, radiation oncologists) dedicated in breast cancer care. Data collection and analysis was informed by the Theoretical Domains Framework (TDF), a behavior change framework comprised of 14 theoretical domains. Determinants were first coded into the 14 TDF domains, codes were then transformed into specific belief statements, and similar belief statements grouped into broader themes. Clinicians were then asked to prioritize the themes for influence on their use of advanced medical imaging in patients with early stage breast cancer.

**Findings:** Thirty-six clinicians were interviewed. Twenty four themes emerged and were prioritized by a sample of 14 clinicians. The top five themes, receiving an average prioritization rating of > 7 (scale:1-10) scale were: 1) physician knowledge of evidence regarding efficacy and yield of staging tests, 2) physician knowledge of recommendations to not order staging tests to detect metastatic disease for early stage breast cancer, 3) physician conviction of the negative aspects of ordering unnecessary tests, 4) training and experience, and 5) physician confidence to manage early stage breast cancer patients without ordering staging tests.

**Implications for D&I Research:** These findings are being used to develop an implementation intervention to reduce the routine use of advanced medical imaging in patients with early stage breast cancer. Our intervention will help build the science of dissemination and implementation, and if effective, is expected to improve the care of women with breast cancer while promoting the responsible use of health care resources.

**Primary Funding Source:** Canadian Cancer Society

### S13 Implementation of practice-based intervention and changes in providers' behaviors related to HPV vaccination: results from a multiple baseline random selection study

#### Joan Cates^1^, Justin Trogdon^1,2^, William Calo^3^, Jamie Crandell^1^, Sandra Diehl^1^, Laurie Stockton^1^, Tamera Coyne-Beasley^1^

##### ^1^ University of North Carolina at Chapel Hill, Chapel Hill, NC, USA; ^2^ RTI International, Durham, NC, USA; ^3^ Penn State College of Medicine, Hershey, PA, USA

###### **Correspondence:** Joan Cates (joancates@unc.edu)

**Background:** An important barrier to preteen human papillomavirus (HPV) vaccination in the US is reluctance by both providers and parents to discuss sexually transmitted infections (STIs). Multilevel interventions that engage providers, parents and preteens in practice settings are promising but largely under-explored. To address this barrier, we developed *Protect Them*, a novel practice-based communication intervention. *Protect Them* includes strategies grounded in the domain of inner setting, an important construct of the Consolidated Framework for Implementation Research (CFIR) With data collected from providers in each enrolled practice, the present study reports on the use of intervention components and changes in providers' HPV vaccination behaviors.

**Methods:** We used a multiple baseline design to recruit two groups of primary care practices (k=28) at staggered nine-month intervals. Practices were eligible if they report vaccine administration to the North Carolina Immunization Registry and have ≥100 unvaccinated 11-12 year olds at baseline. Intervention components were print (brochures and posters) and interactive (web portal and online training) materials to enhance providers’ conversations with parents and preteens. Providers at each participating clinic completed a baseline survey, prior to implementation, and a post-intervention survey, one month after the nine month intervention. Both surveys assessed providers’ HPV vaccination behaviors, and their personal use of one or more of the intervention components.

**Findings:** Providers (n=89) were physicians (66%), nurse practitioners (28%), and physician assistants (6%). The majority (64%) reported using intervention components. In an intention-to-treat analysis, one month post intervention, providers reported significantly higher levels of HPV vaccine discussions with parents (p=.02), vaccine recommendation (p=.04), and vaccine administration (p=.0003) compared to baseline. Post intervention, providers had lower levels of concern about parent upset about an STI vaccination and also the time it takes to discuss HPV vaccination (p=.046 and .057, respectively). Most other variables about the likelihood of discussing sexual health topics did not change.

**Implications for D&I Research:** The intervention was associated with improvements in providers' HPV vaccination behaviors; most providers reported use of intervention components in their practice settings.

**Primary Funding Source:** National Institutes of Health

### S14 Do learning collaboratives increase knowledge and sustain practices?

#### Rochelle Hanson^1^, Benjamin Saunders^1^, Sonja Schoenwald^2^, Jason Chapman^2^, Angela Moreland^1^, Sam Peer^1^

##### ^1^ Medical University of South Carolina, Charleston, SC, USA; ^2^ Oregon Social Learning Center, Eugene, OR, USA

###### **Correspondence:** Rochelle Hanson (hansonrf@musc.edu)

**Background:** Adverse consequences of youth trauma exposure highlight the need for quality delivery of evidence-based treatments (EBTs). While several trauma-focused mental health EBTs exist, access and availability are not universal and an ongoing challenge centers on determining effective and scalable strategies to support their sustained, quality delivery. Learning Collaboratives (LC) include evidence-based strategies to increase EBT knowledge and skill. While the LC has been increasingly used to train clinical providers, it has not typically targeted broker professionals who identify, screen and refer youth for specialized (i.e., trauma-focused) treatment services, and create demand for quality EBT delivery. The Community-Based Learning Collaborative (CBLC) extends the LC by including clinical and broker participants in training and implementation to improve knowledge and change practice to promote EBT sustainment across communities.

**Methods:** Data are from an NIMH grant that is evaluating mechanisms by which the CBLC affects penetration and sustainment. This presentation focuses on changes in the knowledge and practices of participants in 6 CBLCs that were implemented as part of a statewide initiative to promote TF-CBT. Data from online surveys of 279 (42.3%) participants, completed pre and post CBLC included a 31-item therapist-report TF-CBT competence measure (a = .87) and 5 scales (a’s ≥ .87) that assessed broker knowledge and practices (e.g., knowledge about trauma and TF-CBT, family engagement practices, and evidence-based treatment planning). A subsample of participants (*n =* 45) completed semi-structured telephone interviews to obtain feedback about the CBLC and its impact on knowledge and professional practices.

**Findings:** Paired samples *t*-tests indicated significant improvements across all measures (*p <.* 001*).* For the interviews, qualitative content analyses with NVivo were conducted, informed by grounded theory approach, to identify emerging themes related to the impact of the CBLC (e.g., increased knowledge; networking; shared learning; team building). The presentation will include a discussion of the convergence and complementarity of quantitative and qualitative data related to relationships between CBLC participation and impact on sustainability of professional practices.

**Implications for D&I Research:** Findings provide support for the CBCL model as a training/implementation approach, with potential for sustainability of practices over time. Study limitations and future directions will be discussed.

**Primary Funding Source:** National Institutes of Health

### S15 Adherence to implementation strategy reporting recommendations in mental health: a scoping review

#### Cole Hooley, Takashi Amano, Lara Markovitz, Enola Proctor

##### Brown School of Social Work, Washington University in St. Louis, St. Louis, MO, USA

###### **Correspondence:** Cole Hooley (colehooley@gmail.com)

**Background:** Others have reported inadequate implementation strategy reporting. Insufficient reporting has limited efforts to conduct meta-analyses and reviews. This paper provides more pointed guidance to strengthen reporting efforts. Specifically, this paper seeks to 1) identify the adherence rates for each of the specified domains of the implementation strategy reporting guidelines, 2) identify problematic domain-specific reporting, and 3) conduct exploratory analysis to determine what influences adherence.

**Methods:** The search included implementation strategy, behavioral health, and evaluation constructs. The hedge was executed in Embase, Medline, Academic Search Complete, and PsychInfo. Exclusion criteria included: not original data, did not address mental health interventions and outcomes, and published prior to the implementation strategy reporting guidelines. 3,183 unique citations were identified. After applying the exclusion criteria and double-reviewing the titles/abstracts, 64 articles remained. Pertinent data were extracted from the articles, and each article was double-coded and assigned an adherence score for each guideline domain (eight domains, 0 – 2 points per domain, highest possible sum score 16). Linear regression was used for exploratory analysis. The dependent variable was the article’s adherence sum score. Independent variables included: protocol paper status, hybrid design, publication year, and number of strategies reported.

**Findings:** Of those articles extracted to-date (N=30), the adherence score ranged from 2 to 11 with a mean 6.23, and standard deviation 2.10. The most reported domains were: actor, action, target, and dose. Least reported domains included: definition, justification, temporality, and outcome. 83.3% of the studies reported no strategy definitions, 76.7% reported no justification for the strategies, and 70% did not report temporality. Furthermore, 46.7% did not connect the strategy to an implementation outcome. The average sum score increased after the publication of the reporting guidelines, though not to a statistically significant degree. The exploratory analysis yielded no significant outcomes.

**Implications for D&I Research:** Implementation strategy reporting continues to be a challenge. Adopting guidelines and including them in the reviewers’ criteria could improve adherence. Better reporting leads to stronger meta-analyses and reviews. Given that most studies utilized multiple strategies, requiring a specific table be included in the supplemental materials may be more effective. Study limitations and further recommendations will be discussed.

### S16 How organizational factors influence training impact over time within community mental health clinics

#### Victoria Stanhope^1^, Abigail Ross^2^, Mimi Choy-Brown^1^, Lauren Jessell^1^

##### ^1^Silver School of Social Work, New York University, New York, NY, USA; ^2^Graduate School of Social Service, Fordham University, New York, NY, USA

###### **Correspondence:** Victoria Stanhope (victoria.stanhope@nyu.edu)

**Background:** Training providers is a key implementation strategy with high costs but inconsistent results. To maximize training impact, we need to understand the interplay between training factors (dose) and organizational factors (e.g., leadership). This longitudinal mixed-methods study examined a one-year training process across seven organizations to explore the relationship between training dosage, organizational factors and implementation readiness over time.

**Methods:** Seven community mental health clinics participated in an NIMH-funded RCT of person-centered care planning (PCCP). Training consisted of providers (n=132) receiving a two-day in-person presentation on PCCP and one-year of monthly technical assistance (TA) calls. Trainers completed monthly site ratings of implementation leadership and readiness. Focus groups (n=14) were conducted with providers at each site to understand the training process. Descriptive and correlational analyses were used to calculate and compare implementation trajectories for each site. Paired sample t-tests compared implementation leadership during the first and second halves of the training period. Thematic analysis was used to analyze qualitative data

**Findings:** Trajectories indicated that levels of implementation readiness varied across clinics but most experienced a change towards a more positive trend at the mid-point of the training. Implementation leadership was positively correlated with implementation readiness across time points (r=0.69; p<0.001), with leaders being more proactive in the second half of the training (p=.03; df=6; t=2.73). Focus group findings revealed the presence and resolution of implementation barriers during the course of the training. Barriers included a lack of buy-in, a belief they were already doing PCCP, competing demands, and problems with electronic health records.

**Implications for D&I Research:** The study showed the training had less impact in the first six months compared with the second six months. Implementation leadership was also associated with changes in implementation readiness. Providers reported barriers related to competing demands, lacking necessary infrastructure to support implementation, and not feeling vested in the practice. This suggests that resolving barriers before proceeding with training and having a consistently engaged leadership throughout training may increase impact and decrease the training dosage needed for implementation.

**Primary Funding Source:** National Institutes of Health

### S17 Workplace-based clinical supervision as an implementation strategy: content, techniques, and client outcomes

#### Shannon Dorsey, Michael Pullmann, Rosemary Meza, Prerna Martin

##### University of Washington, Seattle, WA, USA

###### **Correspondence:** Michael Pullmann (pullmann@uw.edu)

**Background:** Workplace-based clinical supervision in public mental health is an understudied and underutilized resource for supporting evidence-based treatments (EBTs), despite the fact that it may offer a cost-effective way to support clinician fidelity to EBT.

**Methods:** Data come from a NIH funded study on supervision within a state-funded EBT implementation effort. In Phase I (“usual care” supervision), workplace-based supervisors audiorecorded supervision of a child trauma EBT (N = 28 supervisors, 98 clinicians). These audiorecordings were objectively coded. The coding measure captured intensity of 27 supervision domains, including 14 content areas (exposure, homework assignment/review) and 13 techniques (providing clinical suggestions, behavioral rehearsal, modeling). Coder reliability was excellent (ICC_(2,6)_ = .87). In Phase II, 48 supervisors and 212 clinicians participated in a randomized controlled trial (RCT). Clinicians were randomized to two supervision conditions that included “gold standard” techniques: symptom and fidelity monitoring (SFM) vs. SFM+ Behavioral Rehearsal (BR). Supervisors provided both conditions, with regular adherence and contamination checks. Child functioning data was collected from parents and children in both phases (N = 305).

**Findings:** Findings from Phase I coding suggested that several EBT content items occurred frequently (e.g., exposure occurred in 81% of sessions; coping skills in 76%), but coverage was mostly of moderate to low intensity. Some content items rarely occurred (clinician use of modeling; client behavioral rehearsal). Many gold standard techniques occurred rarely (review of actual practice: 5%; behavioral rehearsal: 16%) or at low intensity. However, symptom monitoring occurred frequently and often at moderate intensity. In the Phase II RCT, Generalized Estimating Equations revealed that SFM outperformed usual care for child report of Posttraumatic stress severity (Beta = -2.77, *p* = .02), while BR had less statistical support (Beta = -2.15, *p* = .06). There were no differences on parent report.

**Implications for D&I Research:** These findings suggest that workplace-based clinical supervision holds promise for supporting EBT implementation, with some supports. In particular, given that supervisors rarely use many gold standard techniques, and use of some (SFM), but not others (BR) was associated with improved client outcomes, strategies for helping workplace supervisors change their practices may be needed.

**Primary Funding Source:** National Institutes of Health

### S18 Feasibility, acceptability, and preliminary impact of the cornerstone mentoring program

#### Andrea Cole^1^, Michelle Munson^1^, Shelly Ben-David^2^, Beth Sapiro^3^, James Railey^1^, Victoria Stanhope^1^

##### ^1^Silver School of Social Work, New York University, New York, NY, USA; ^2^ School of Social Work, University of British Columbia, Kelowna, BC, Canada; ^3^ School of Social Work, Rutgers University, New Brunswick, NJ, USA

###### **Correspondence:** Andrea Cole (arc483@nyu.edu)

**Background:** Mentoring has shown moderate effectiveness for improving academic and mental health outcomes among youth with mental health conditions, but few of these mentoring programs exist within mental health settings and none of them recruit mentors who have lived experience with mental illness. The Cornerstone Mentoring Program (CMP) was designed to establish dyadic mentoring relationships for transition-age youth with serious mental illnesses. The CMP is embedded in Cornerstone, a multi-component psychosocial intervention. This study examined feasibility, acceptability, and preliminary impact of the CMP.

**Methods:** Within a Hybrid Type 2 trial, investigators developed an interview protocol on feasibility and implementation strategy domains (Powell et al., 2012). Face-to-face interviews were conducted with stakeholders (n=10) and multi-disciplinary experts (n=20) on programmatic aspects of the CMP. Concurrently, analysts coded case summaries written by mentors (n=20). Multiple coders analyzed the data using constant comparison. Iterative discussion(s) occurred over six months until saturation was met.

**Findings:** Feasibility data suggest that mentoring can be implemented within mental health settings and that mentors report mutually satisfactory relationships between mentors and mentees. Implementation data emerged on the policy context, with respondents discussing value-based payment and the importance of tracking non-billable tasks of mentors. Data also pointed to important areas of planning: integration of mentors within the clinic, regular team check-ins, and the increased use of technology by mentors. Data emerged on the importance of training for mentors, for example, on relationship skills, and the importance of supervision. Data suggest promising outcomes for youth participating in the overall Cornerstone model, with staff reporting improved engagement, mental health, social relationships, and functional outcomes.

**Implications for D&I Research:** Results indicate that mentoring in mental health settings is feasible, acceptable, and has promise. Since these data were collected as part of a Hybrid Type 2 trial, the designing and testing of an evidence-based practice, Cornerstone, is concurrent with the examination of implementation factors in a community-based agency. The results from the implementation study of the mentoring component can rapidly inform the ongoing development of the intervention and be integrated into further RCTs, which the research team is currently developing.

**Primary Funding Source:** National Institutes of Health

### S19 Using the coaching implementation strategy to implement evidence-based mental health practices in schools: effectiveness and feasibility of trails

#### Elizabeth Koschmann^1^, James Abelson^1^, Shawna Smith^1^, Kate Fitzgerald^1^, Anna Pasternak^1^, Amy Kilbourne^1,2^

##### ^1^ University of Michigan, Ann Arbor, MI, USA; ^2^ QUERI, Department of Veterans Affairs, Ann Arbor, MI, USA

###### **Correspondence:** Shawna Smith (shawnana@med.umich.edu)

**Background:** With 20-30% of school age children affected by mood and anxiety disorders, schools provide an ideal venue for improving access to evidence-based mental health practices (EBPs). In particular, training existing school professionals (SPs) to deliver mental health EBPs in the context of available student support services could substantially improve access. However, SP training opportunities in EBP are expensive and lack the follow-up supported practice necessary for ensuring effective EBP implementation. Coaching, an implementation strategy that provides expert-led post-training support and live practice, holds promise for improving the uptake and sustainability of EBPs among SPs in diverse school settings.

**Methods:** In this pilot Type II hybrid implementation-effectiveness study, we examined the feasibility and effectiveness of a novel coaching-based implementation strategy for integrating Cognitive Behavioral Therapy (CBT) into 24 diverse public school settings. The implementation strategy incorporated didactic training in CBT for SPs followed by live expert coaching during co-facilitation of CBT groups offered to students during school hours for 12-16 weeks. Feasibility was evaluated via success in recruiting and coaching SPs, and student retention in CBT groups. Mixed-effects models assessed effectiveness as over-time changes in SP confidence delivering CBT, frequency of CBT skill utilization, and perceptions of CBT utility for the school setting, as well as student symptom improvement.

**Findings:** SPs (N=53) from 24 public schools were recruited to participate in coaching. All SPs participated in training and 49 (92%) completed the full course of coaching. SPs saw significant improvements in CBT confidence (*B*^*sy*^=1.27; p<0.001), utilization (*B*^*sy*^=0.86; p<0.001), and attitudes towards CBT (*B*^*sy*^=0.75; p<0.001) following training and coaching. For student participants (n=293), average PHQ-9-measured depression decreased from 10.1 prior to CBT group participation to 7.7 at group end (p<0.001); and GAD-7-measured anxiety declined from 9.1 to 7.1 (p<0.001).

**Implications for D&I Research:** Delivery of EBPs in novel settings, including schools, provides a compelling means of increasing access and evidence-based practice effectiveness, but requires development, deployment, and assessment of novel implementation strategies. Coaching significantly improved SP ability to deliver CBT in schools and improved student mental health outcomes, furthering evidence that the coaching implementation strategy is a promising means of diffusing EBPs into community settings.

**Primary Funding Source:** Centers for Medicare and Medicaid Services

## Big Data and Technology for Dissemination & Implementation Research

### S20 Scale-up, spread, and sustainment of tele-collaborative care for bipolar disorder: lessons learned from a model-guided, mixed methods analysis

#### Mark S Bauer^1,2^, Lois Krawczyk^3^, Kathy Tuozzo^4^, Cara Frigand^1^, Sally Holmes^1^, Christopher Miller^1,2^, Erica Abel^7^, David Osser^3^, Aleda Franz^5^, Cynthia Brandt^5,6^, Meghan Rooney^7^, Jerry Fleming^3^, Eric Smith^1^, Linda Godleski^5^

##### ^1^ Center for Healthcare Organization and Implementation Research, VA HSR&D, Bedford/Boston, MA, USA; ^2^ Harvard Medical School, Boston, MA, USA; ^3^ VA Boston Healthcare System, Brockton, MA, USA; ^4^ VA National TeleMental Health Center, VA Connecticut, West Haven, CT, USA; ^5^ VA Connecticut Healthcare System, West Haven, CT, USA; ^6^ Yale Center for Medical Informatics, Yale School of Medicine, New Haven, CT, USA; ^7^ Richmond VA Medical Center, Richmond, VA, USA

###### **Correspondence:** Mark S Bauer (mark.bauer@va.gov)

**Background:** Health interventions delivered via clinical video teleconferencing have strong empirical support from clinical trials and structured demonstration projects. However, their implementation and sustainability under less structured clinical conditions has not been well demonstrated. Virtually nothing is known about scale-up and sustainment of such technology-based interventions

**Methods:** We conducted a follow-up analysis of the implementation and sustainability of a clinical video teleconference-based collaborative care model for individuals with bipolar disorder treated in the Department of Veterans Affairs (**VA**) in order to: (a) characterize the extent of implementation and sustainability of the program after its establishment, and (b) identify barriers and facilitators to its implementation and sustainability. We conducted a mixed methods program evaluation, assessing quantitative aspects of implementation according to the RE-AIM implementation framework. We conducted qualitative analysis of semi-structured interviews with 16 providers who submitted consults, utilizing the i-PARIHS implementation framework.

**Findings:** The program demonstrated linear growth in sites (n=35) and consults (n=915) from late 2011 through mid-2016. Analysis indicated sustained, statistically significant growth from year 1 to year 2 after program activation. Qualitative analysis identified key facilitators including: consult content, ease of use via electronic health record, and national infrastructure. Barriers included limited availability of telehealth space, equipment, and staff at the sites, as well as complexity in scheduling.

**Implications for D&I Research:** The program achieved a steady growth rate, as it: successfully filled a need perceived by providers, developed in a supportive context, and received effective facilitation by national and local infrastructure. Clinical video teleconference-based interventions, including multi-component collaborative care interventions for individuals with complex mental health conditions, can grow vigorously under appropriate conditions that address relevant barriers and facilitators. Results contribute to the knowledge of factors necessary to implement and sustain technology-intensive clinical interventions, and provide methods by which to do so.

**Primary Funding Source:** Department of Veterans Affairs

### S21 Using computational linguistics to scale out evidence-based mhealth interventions

#### Carlos Gallo^1,2^, Brian Mustanski^1,2,3^, C. Hendricks Brown^1,2^, Kevin Moran^3^

##### ^1^ Northwestern University Feinberg School of Medicine, Chicago, IL, USA; ^2^ Center for Prevention Implementation Methodology for Drug Abuse and HIV, Chicago, IL, USA; ^3^ Institute for Sexual and Gender Minority Health and Wellbeing, Chicago, IL, USA

###### Correspondence: Carlos Gallo (carlos.gomez.gallo@gmail.com)

**Background:** Adolescent men who have sex with men (AMSM) account for 76% of HIV diagnoses among all young people and continue to face increasing incidence. A number of evidence-based mobile Health interventions (*mHIs*) are designed to reach high risk HIV populations by sending scripted text messages of medication reminders and HIV prevention information. Yet, many *mHIs* suffer from low usage, which reduces their public health impact. Text messages often ignore the participant’s linguistic background, a key component demonstrated to enhance participants’ satisfaction, which can potentially amplify *mHIs* effects. In this study, we demonstrate how to optimize mHealth text-messaging interventions by increasing engagement to and satisfaction with the intervention in an efficient, scalable, and non-obtrusive manner.

**Methods:** We developed a computational linguistic method to analyze the linguistic style of text messages (M=17784) exchanged by AMSM participants (N=132) aged 14 to 18, in a completed, randomized controlled trial, Guy2Guy (G2G). This *mHI* sent prevention scripted messages and provided an interactive platform that matched AMSM into dyads for peer support and skill practice. Dyads were matched on age, geographical location, and sexual experience. We extracted linguistic features automatically and computed a linguistic similarity score between participants. Dyads were dichotomized in high or low engagement based on the number of messages exchanged. We trained a regression model to predict engagement and satisfaction based on peer linguistic similarity.

**Findings:** The messages exchanged within dyads vary widely (mean=269, sd=457). Linguistic similarity score positively predicted the number of messages exchanged in the peer support platform (p<0.002).

**Implications for D&I Research: Computational linguistics methods (CLM) have the potential to facilitate the scaling out of evidence-based mHealth HIV interventions**. First, participants’ linguistic style can reveal the linguistic inter-personal differences useful for tailoring the content and timing of text-messages. Second, detecting linguistic style is not easily done by observers even if trained. CLM are efficient, scalable, non-obtrusive, and automatic, which reduces the need to hire and train personnel dedicated to measuring key features of the mHIs delivery and implementation. This particular mHI improves HIV testing and entry into prevention services, particularly for high risk population, hence, it **helps eliminate HIV health disparities**.

**Primary Funding Source:** National Institutes of Health

### S22 Clinician perspectives on barriers and facilitators for implementing person-generated health data into clinical care

#### Arlene Chung^1,2^, David Gotz^1^, Bryce Reeve^3^, Ethan Basch^1^

##### ^1^ University of North Carolina at Chapel Hill School of Medicine, Chapel Hill, NC, USA; ^2^ Lineberger Comprehensive Cancer Center at UNC Chapel Hill, Chapel Hill, NC, USA; ^3^ Duke University School of Medicine, Durham, NC, USA

###### **Correspondence:** Arlene Chung (arlene_chung@med.unc.edu)

**Background:** As healthcare moves towards value-based care, there is an increasing interest for implementing person-generated health data (PGHD), such as digital device data and electronic patient-reported outcomes (ePROs), into clinical care. While potential benefits of PGHD are clear, less is known about clinician preferences for how PGHD should be displayed and used within clinical workflows and electronic health records (EHRs) and about the perceived barriers and facilitators for the implementing PGHD.

**Methods:** To explore acceptability and the barriers and facilitators of implementing PGHD (e.g., fitness trackers and ePROs) for remote monitoring of chronic conditions, we conducted 21 semi-structured interviews with oncologists (n=11) and primary care providers (n=10) from a large academic center. Interviews were audiotaped, transcribed verbatim, and coded independently. Coding disagreements were adjudicated by consensus. Themes were categorized into barriers and facilitators for implementing PGHD.

**Findings:** All clinicians expressed interest in implementing PGHD. While clinicians cited benefits for using PGHD to capture the longitudinal patient experience, strengthen communication and engagement, and decrease healthcare utilization, multiple barriers were noted. These included liability, lack of time/compensation for reviewing PGHD, burden of managing PGHD, concerns about device accuracy, lack of protocols for handling continuous data streams, and the need for care coordination to manage PGHD. PGHD from digital devices and ePROs were not perceived to have equal utility with fitness tracker data being perceived as the least useful. EHR data visualization, actionable alerts/notifications, and outsourcing management to nurses were cited as key facilitators for using PGHD. Clinically meaningful thresholds for PGHD were felt to be necessary, but not all data types currently have clear guidance. It was also felt that the context of the patient and their condition(s) should guide the frequency of data capture/collection.

**Implications for D&I Research:** To implement PGHD into clinical care for remote monitoring, considerations for workflow, data visualization, and processes are critical to ensure appropriate review and use. Existing EHR solutions may not address user’s needs or workflows, and are a barrier to implementation. This research highlights the challenges of integrating new data streams and the opportunities in implementation science to advance incorporating the patient experience into routine care processes.

**Primary Funding Source:** National Institutes of Health

### S23 Point-of-care prioritized clinical decision support reduces cardiovascular risk in adults with elevated cardiovascular risk: randomized trial

#### Patrick O'Connor, JoAnn Sperl-Hillen, Lauren Crain, Karen Margolis, Heidi Ekstrom, Deepika Appana, Gerald Amundson, Rashmi Sharma, Jay Desai

##### HealthPartners Institute, Minneapolis, MN, USA

###### **Correspondence:** Patrick O'Connor (patrick.j.oconnor@healthpartners.com)

**Background:** The potential of informatics-based clinical decision support (CDS) systems to improve chronic disease care outcomes has rarely been realized.

**Methods:** In this group-randomized nested cohort trial 20 primary care clinics were randomly assigned to usual care (UC) or CDS intervention arms to test the hypothesis that an electronic health record (EHR)-linked Web-based cardiovascular CDS system can reduce cardiovascular risk in adults age 18-75 years without diagnosed diabetes mellitus or cardiovascular (CV) disease, but with elevated blood pressure (BP), uncontrolled lipids, or current tobacco use at an index visit. The main outcome measure was predicted annual rate of change in 10-year CV risk (fatal or nonfatal heart attack or stroke) using the Framingham Risk Equations. Secondary objectives were assessment of CDS use rates and primary care provider (PCP) satisfaction with the CDS system. After entry of BP by the clinic rooming nurse, relevant data were extracted from the EHR, encrypted, and processed through Web-based clinical algorithms that (a) determined if the patient met intervention eligibility criteria, (b) identified evidence-based treatment options for any uncontrolled CV risk factors, and (c) prioritized treatment recommendations based on potential CV risk reduction. CV risk factors addressed in these study participants were control of lipids, BP, weight, tobacco, and appropriate aspirin use. Personalized treatment recommendations were printed in different versions and given to PCP and patient immediately before the visit.

**Findings:** The CDS system was used on a sustained basis at 71-77% of targeted visits of 7595 eligible study subjects, and PCPs reported 85% to 98% satisfaction with the CDS system. Predicted annual rate of change in 10-year CV risk was significantly better in CDS clinics than in UC clinics (-0.51% versus +1.69%; *P*<0.001). The predicted absolute difference in 10-year CV risk at 12 months post-index was -2.3%, favoring the CDS group (UC 24.8%, CDS 22.5%, *P*<0.001).

**Implications for D&I Research:** This CDS system had high use rates at targeted visits, high PCP satisfaction, and significantly improved predicted CV risk in targeted adults. Evaluation of this system in other care delivery settings and extension of this CDS system to other clinical domains should be considered.

**Primary Funding Source:** National Institutes of Health

### S24 Influencing clinical outcomes with automated sepsis time zero through process improvements and statistical validation

#### Karen Colorafi^1^, Joseph Colorafi^2^, Alyson D'Andrea^2^, Ken Ferrell^2^

##### ^1^ Arizona State University, Scottsdale, AZ, USA; ^2^ Dignity Health, Phoenix, AZ, USA

###### **Correspondence:** Joseph Colorafi (joseph.colorafi@dignityhealth.org)

**Background:** More than a million Americans experience severe sepsis each year, an overwhelming response to infection with a mortality rate nearing 50%. The early identification of sepsis is key to initiating therapy that saves lives. Traditionally, chart auditors identify time zero (the presentation of sepsis) post-discharge and calculate compliance with timely sepsis bundle orders retrospectively.

**Methods:** In order to alert clinicians to the risk of sepsis as early as possible through clinical decision support (CDS), we automated time zero and made it visible real-time through a proprietary sepsis application. Initially piloted at two facilities, we automated technology to all of our facilities within eight months and continue to add it into our standard EMR conversion scope. Outcomes measures of interest included: the positive predictive value (PPV) of our sepsis algorithm with and without automated time zero, year over year decline in mortality, and sepsis bundle compliance.

**Findings:** PPV improved more than 20% from the standard vendor to our optimized version. We noted an average 4% year over year decline in mortality across all facilities with the addition of automated time zero to our existing predictive sepsis algorithm. Average sepsis bundle compliance also improved 34% to 50%.

**Implications for D&I Research:** The automation of time zero was positively correlated with early intervention and the use of standard sepsis bundles, which are known to decrease mortality. Our agile implementation methodology in conjunction with Hadoop™ solution architecture allowed us to scale, to do in-house statistical validation of CDS, to design and build our proprietary application and deploy automated time zero as quickly as people and process would allow across 34 inpatient facilities and their emergency departments (ED). We saw an improvement in bundle compliance and uptake in app use. Future work includes the use of machine learning to improve the algorithm and the display of time zero across dashboards tailored to location-specific (e.g.: ED) needs.

### S25 Parade (Patients at Risk for Adverse Drug Events) model for outcome-driven admission medication reconciliation

#### Boryana Manz^1^, Manjula Julka^1^, Kristin Alvarez^1,2^, Varun Sharma^2^

##### ^1^ PCCI, Dallas, TX, USA; ^2^Parkland Health and Hospital System, Dallas, TX, USA

###### **Correspondence:** Manjula Julka (manjula.julka@phhs.org)

**Background:** About 5% of hospitalized patients in the United States experience an adverse drug event (ADE) - any harm experienced that is related to medication. ADEs especially occur during care transitions such as hospital admissions and are influenced by a complex etiology of patient, medication, provider, and socioeconomic-specific factors. ADEs adversely impact patient safety, clinical outcomes, hospital utilization, and healthcare costs. Proactive identification of patients at high risk and timely interventions by pharmacists, can significantly reduce potentially preventable ADEs. PARADE is a multifaceted predictive risk score developed for Parkland Hospital (Dallas, TX) to stratify newly admitted patients. It generates real-time actionable worklists within the electronic health record (EHR) that trigger timely and targeted interventions by care teams.

**Methods:** PARADE was developed in a retrospective, cohort study of adult hospitalizations. It conceptually captures medications/disease complexity, prior healthcare utilization, demographics, and social determinants of health. Parkland’s information technology platform facilitated merging of resource utilization, hospital operations, and clinical outcomes data. PARADE was trained and tested on 2016 data (50:50 split) using c-statistic and precision. The model reconciles multivariable logistic regression models for best practices and ADEs. Seamless EHR integration enables dynamically ranked worklists and chart synopsis for timely pharmacy team interventions targeting the highest-risk 5% of patients, marked in red. PARADE is calibrated to the unique care settings/population making it more accurate than published criteria.

**Findings:** Approximately 6000 encounters are screened, 80 consults made, and 700 interventions documented monthly. PARADE showed a c-statistic of 0.74 in the pilot. Preliminary data one month post-implementation shows that consults for high-risk patients have tripled without additional pharmacy resources. Long term impact on rates of ADEs, length of stay, and rehospitalizations will be presented in December 2017.

**Implications for D&I Research:** We developed PARADE on a framework for reconciling resource utilization, best practices, and clinical outcomes, which is easily replicable and scalable across diverse healthcare settings. This multifaceted predictive real-time risk score is EHR-integrated making it readily accessible and actionable. Pharmacy staff or other care team members can select patients for timely interventions in descending risk order, enabling efficient use of limited resources for improved outcomes.

**Primary Funding Source:** Lyda Hill Foundation

## Building the Future of D&I Research

### S26 Overview of speeding research interventions (SPRINT) training

#### April Oh^1^, Cynthia Vinson^1^, Anna Gaysynsky^1^, Tara Loomis^2^

##### ^1^ National Cancer Institute, Rockville, MD, USA; ^2^ ICorps, Venture Well, Hadley, MA, USA

###### **Correspondence:** April Oh (april.oh@nih.gov)

**Background:** SPRINT is a variation of the Lean LaunchPad® (LLP) developed by Steve Blank, taught at Stanford, Berkeley, Columbia, and Caltech, and adopted by the National Science Foundation and NIH for the I-Corps™ program. SPRINT provides behavioral interventionists with real-world learning experiences to successfully transfer knowledge into programs that benefit society. SPRINT provides a supported learning process: participants spend a significant amount of time between each of the lectures in the “marketplace,” talking to customers, and testing their hypotheses. During SPRINT, all team members engage with industry. By talking to customers, partners and competitors, participants encounter the chaos and uncertainty of commercializing interventions. Unique to the LLP approach, SPRINT emphasizes experiential learning, a flipped classroom and immediate feedback to engage students with real world entrepreneurship. Trainees learn by proposing and immediately testing hypotheses – they do, rather than plan to do. Unlike many approaches to entrepreneurship education, LLP does not rely on static case studies or fixed models; it challenges students to create their own business models based on information derived from personal engagement rather than secondhand market research. The goal of a traditional LLP course is to help students turn a business idea into a startup. For the SPRINT program, the focus is on helping researchers determine whether a commercialization pathway may work to advance the dissemination and implementation of their intervention more rapidly.

**Methods:** SPRINT is approximately eight weeks and begins with a three day in person workshop. During the first two days, the teams get out of the building, conduct customer discovery interviews and present their findings to the class. This process continues remotely using an online platform for six weeks.

**Findings:** Teams are expected to conduct a minimum of 30 interviews before the conclusion of the course. The class reconvenes in person and each team is expected to present their lessons learned and a brief video telling their story. The course is taught by two highly skilled and trained National I-Corps™ instructors, both of whom have extensive experiences in startup creation.

**Implications for D&I Research:** SPRINT helps train behavioral interventionists in how to design for dissemination and implementation

**Primary Funding Source:** National Institutes of Health

### S27 Witness cares, LLC

#### Deborah Erwin, Detric "Dee" Johnson, Patrick Emmerling

##### Roswell Park Cancer Institute, Buffalo, NY, USA

###### **Correspondence:** Deborah Erwin (deborah.erwin@roswellpark.org)

**Background:** Witness CARES started as an NCI-R01 multi-site randomized behavioral intervention to better understand factors influencing the decisions of African Americans to engage in (or not engage in) screening for colorectal cancer (CRC). SPRINT was an opportunity to be trained to fast-track our research to achieve our “long-term goal” to develop and disseminate effective intervention strategies to increase CRC screening rates for African Americans to eliminate disparities in morbidity and mortality.

**Methods:** In 8 weeks, we attended 4 days of intensive, in-person training at NCI, attended 20 hours of webinars, conducted 52 in-person or telephone interviews with stakeholders (e.g. insurance company representatives, business owners, patients, GI doctors), read 2 start-up manuals watched hours of videos, presented 10, 10-minute team progress reports, and produced a 3-minute video of our journey.

**Findings:** The Witness CARES team learned how to parlay 20 years of research experience in community-based interventions, social health capital and R01 results into a potentially viable business model. We developed a canvas of Key Partners, Key Activities and Resources for our Value Propositions, how to manage customer relationships, delivery channels, revenues and cost structures to serve our customer segments. Witness CARES became much more than a randomized study – it grew into a group of services potentially able to be commercialized in a contested health care market to benefit low income and African American men and women to access and receive CRC screening services, and possibly more. We incorporated, achieved NYS women and minority-owned business status, are in the process of obtaining resources through STARTUP NY, and a foundation for tech-transfer funding. We plan to submit our NIH/SBIR grant in 2018.

**Implications for D&I Research:** The SPRINT process helped the Witness CARES team to begin to visualize our study interventions through totally new perspectives. The training program provided crucial tools to teach behavioral investigators how to move interventions into the world of profit and loss through grounded business start-up practices. Transforming evidence-based interventions to deliver the products and services that “create gains” and “relieve pains” in the health care market place has excellent potential for broad dissemination.

**Primary Funding Source:** National Institutes of Health

### S28 Sprint training: development and implementation of a mobile behavioral pain program for patients with cancer

#### Tamara Somers, Sarah Kelleher

##### Duke University Medical Center, Durham, NC, USA

###### **Correspondence:** Tamara Somers (tamara.somers@duke.edu)

**Background:** Estimates suggest that 100 million US adults experience chronic pain and the annual cost is $365 billion. Three decades of work have shown that behavioral strategies for pain management are highly efficacious. Yet, this robust evidence remains poorly translated into clinical practice; there is low population-level availability of behavioral pain interventions. Advances in mobile health technologies could fill this void. However, there is limited knowledge on strategies for dissemination and implementation of mobile interventions. Our SPRINT team objective was to learn strategies to develop and widely implement a comprehensive mobile behavioral pain program (i.e., Pain Pac) for cancer patients.

**Methods:** Our SPRINT team consisted of a principle investigator and entrepreneurial lead. Stakeholder interviews were conducted to gain information on strategies for Pain Pac development and implementation.

**Findings:** We conducted 33 interviews with patients/providers (5), decision makers/payers (14), and experts (14). Interviews were in person (18), by phone (8), and through video-conferencing (7). Key findings were: a) healthcare systems are not now widely using mobile technology, but expect to be in the future; b) healthcare providers and patients are enthusiastic about mobile pain solutions but unclear how they will integrate into care; c) mobile pain solutions could improve patient care and decrease healthcare utilization and provider burnout; d) mobile behavioral pain interventions that link with medical record data are desirable. Stakeholders identified the value of such a program to be: mobile access to pain management, decreased pain and disability, improvements in psychological functioning, and safer and more effective than medication management (e.g., opioids). Channels for implementation were also identified (e.g., employers, medical centers, insurers).

**Implications for D&I Research:** Results provide direction for future Pain Pac D&I research. First, it will be important to carefully assess readiness for engagement in such a program by both healthcare systems and providers. Second, understanding optimal strategies for patient engagement in implementation of such a program is critical. Third, future work should assess implementation strategies (e.g., passive push to patient technology vs. healthcare provider recommendation) of Pain Pac carefully considering patient engagement and outcomes as well as cost and sustainability.

**Primary Funding Source:** National Institutes of Health

### S29 Improving implementation with the speeding research interventions (SPRINT) program

#### Anna Gaysynsky^1^, Alissa Ainbinder^2^, April Oh^1^, Tara Loomis^2^, Cynthia Vinson^1^

##### ^1^ National Cancer Institute, Rockville, MD, USA; ^2^ ICorps, Venture Well, Hadley, MA, USA

###### **Correspondence:** Anna Gaysynsky (anna.gaysynsky@nih.gov)

**Background:** Supporting behavioral scientists in the implementation and dissemination of their evidence based interventions may require new and innovative training programs that create a paradigm shift in the way researchers conceptualize and approach their work. Towards this end, the National Cancer Institute (NCI) launched the SPeeding Research INTerventions (SPRINT) Program, which focuses on commercialization concepts and methods, and is designed to foster an innovation ecosystem for behavioral interventionists. The program, which is led by instructors with extensive startup and teaching experience, provides real-world, hands-on training in how to successfully incorporate innovations in cancer control into successful products within a marketplace. The ultimate goal is to create scalable research-tested behavioral interventions that are ready to be put into real-world practice and will reach a large audience of users.

**Methods:** This presentation will share core evaluation findings and lessons learned about training teams of behavioral interventionists to apply commercialization and implementation science concepts to their work. The course was evaluated using a mixed-methods approach that collected both quantitative and qualitative data from SPRINT participants (n=20 teams consisting of 2-4 team members). Data came from a pre-course survey and a “post-course” survey administered to each cohort of participants, as well as two rounds of focus group data.

**Findings:** Evaluation results indicate that after the course, participants gained knowledge of business model concepts, particularly in the areas of “customer segments” and “channels”. Most respondents rated the course highly: 91-92% of participants rated the course as "good" or "very good" and 84-86% would recommend SPRINT to their peers.

**Implications for D&I Research:** Findings suggest the course “revolutionized” the way participants think about designing future interventions and implementing their current work. However, the data also reveal important barriers relevant to the field of implementation science, including a lack of familiarity with the language of translation and implementation, lack of institutional support and little recognition of the value of behavioral science in tech transfer, the difficulty in identifying a marketplace for behavioral interventions, and challenges in learning an unfamiliar “market-driven” approach. Findings also suggest a need for more reflection about the overlap and distinction between dissemination and implementation, and commercialization pathways.

**Primary Funding Source:** National Institutes of Health

### S30 Computer-facilitated 5A’s (Ask, Advise, Assess, Assist, and Arrange) for smoking cessation: using technology to promote provider adherence to clinical guidelines

#### Jason Satterfield^1^, Steven Gregorich^1^, Sara Kalkhoran^2^, Ricardo Munoz^1,3^, Maya Vijayaraghavan^1^

##### ^1^ University of California San Francisco, San Francisco, CA, USA; ^2^ Harvard Medical School, Boston, MA, USA; ^4^ Palo Alto University, San Francisco, CA, USA

###### **Correspondence:** Jason Satterfield (jason.satterfield@ucsf.edu)

**Background:** Innovative service delivery models are needed to improve primary care provider (PCP) adherence to the 5A’s for smoking cessation. While most PCPs “ask” and “advise” their patients about smoking, clinician adherence to “assist” and “arrange” remain low due to limited time, competing demands and insufficient provider confidence and skill. We evaluated the effectiveness of the Computer-Facilitated 5A’s (CF5A’s), a digital health intervention to improve 5A’s fidelity and adherence.

**Methods:** PCPs at 3 diverse, urban clinics were randomized into CF5A’s or usual care (UC) conditions. Adult smokers, defined as smoking 100 lifetime cigarettes and at least one cigarette in past week, were recruited in primary care waiting rooms and assigned to their provider’s condition. Intervention patients completed the CF5A’s on a computer tablet just prior to their PC appointment. Two tailored clinical summaries with intervention recommendations were generated; one for the PCP and one for the patient. Within 72 hours of the appointment, patients completed a post-visit interview about their receipt of the 5A’s during their primary care encounter. Patients could participate up to three times within the yearlong study.

**Findings:** N=221 primary care providers saw n=961 patients (n=412 intervention; n=549 control) for a total of n=1,340 visits. N=1,011 post-visit surveys were completed (75.4% response rate). After controlling for 4-level nesting effects, GEE models showed intervention PCPs were 32% more likely to “Assess” (OR 1.32; 95% CI, 1.01-1.72) and 45% more likely to “Assist” (OR 1.45; 95% CI, 1.08-1.93). When looking at first study visits only, intervention providers were 72% more likely to “Arrange” a follow-up visit (OR 1.72; 95% CI, 1.23-2.40), and 104% more likely to complete all 5A’s (OR 2.04; 95% CI, 1.35-3.07) but study visit did not influence other outcomes.

**Implications for D&I Research:** A computer-facilitated 5A’s delivery model was effective in improving provider adherence to the 5A’s. This relatively brief, low cost intervention utilized PCP social influence while saving PCP time and engaging patients. Similar digital health interventions have great potential to facilitate evidence-based smoking cessation and other behavioral counseling interventions. Future studies should help identify ways to promote and sustain technology implementation and integration with existing clinic flows.

**Primary Funding Source:** National Institutes of Health

### S31 Application of system dynamics to inform a model of adolescent screening, brief intervention, and referral to treatment (SBIRT) implementation in primary care settings

#### Shannon Mitchell^1^, David Lounsbury^2^, Zhi Li^3^, Robert Schwartz^1^, Jan Gryczynski^1^, Arethusa Kirk^4^, Marla Oros^5^, Colleen Hosler^5^, Kristi Dusek^1^, Barry Brown^6^

##### ^1^ Social Research Center, Friends Research Institute, Baltimore, MD, USA; ^2^ Albert Einstein College of Medicine, Bronx, NY, USA; ^3^ Institute for Systems Genetics, New York University, New York, NY, USA; ^4^ Total Health Care, Baltimore, MD, USA; ^5^ The Mosaic Group, Baltimore, MD, USA; ^6^ University of North Carolina at Wilmington, Wilmington, NC, USA

###### **Correspondence:** Shannon Mitchell (smitchell@friendsresearch.org)

**Background:** The implementation of screening and brief intervention within primary care settings poses significant challenges related to complexity of the setting as well as the coordination of staff. This can be especially challenging when behavioral health is being integrated into primary care settings in novel ways. The proposed presentation will describe the application of system dynamics (SD) modeling to better understand the influence of different implementation strategies on the effective implementation of adolescent screening and brief intervention for substance use in US urban primary care clinics.

**Methods:** Using data from a NIDA-funded cluster randomized trial of adolescent SBIRT implementation involving seven federally qualified health center sites, we examined the effect of varying quality and frequency of training and trouble-shooting efforts. Simulated over a 20-month intervention implementation period, we used our SD model to compare our ‘Basecase’ (calibrated) outcome [i.e., High quality on-going technical assistance (TA) with quarterly site-specific performance feedback reporting (PFR)] to five strategy scenarios.

**Findings:** Our SD model, supported by qualitative and quantitative data from the study, effectively represented the SBIRT intervention, which was calibrated to reflect actual monthly volume of adolescent primary care visits (N=10,090), screenings (N=5,452), positive screenings (N=1,363), and brief interventions (BIs; N=49). Decreasing PFR to twice per year (Bi-annual) as opposed to quarterly, and decreasing quality of TA by 50% served to reduce BI delivery by two-thirds (Scenario1 and Scenario4; 64.7% and 68.1% reduction, respectively, by month 20). Merely reducing the quality of TA by 25% was least detrimental (Scenario2; 36.2% reduction by month 20). Most detrimental to BI delivery were reductions in both TA and PFR (Scenario3 and Scenario5; 78.5% and 89.6% reduction, respectively, by month 20).

**Implications for D&I Research:** SD modeling is a robust method for comparative analyses of implementation strategies. This approach facilitates synthesis of multiple sources of information/data and can foster important insights about how to deploy limited resources for training and support in diverse clinical sites. Factors, such as behavioral health availability, can be modeled in order to optimize training frequency for adolescent SBIRT service delivery in primary care settings.

**Primary Funding Source:** National Institutes of Health

### S32 Implementation engineering

#### David Gustafson, Andrew Quanbeck, Randall Brown

##### University of Wisconsin – Madison, Madison, WI, USA

###### **Correspondence:** David Gustafson (dhgustaf@wisc.edu)

**Background:** This session: 1) presents findings of a study aimed at implementing a smartphone based relapse prevention program in three federally qualified health centers in Madison, WI, Bronx, NY, and Missoula, MT; 2) discusses the implications of that research; and 3) offer thoughts about approaching implementation, dissemination and sustainability from an engineering, mass production perspective.

**Methods:** An evidence-based mobile health (mHealth) system named “Seva” was introduced sequentially over 36 months at each site using a multiple baseline design. Clinicians offered Seva to up to 100 substance-using patients per clinic. Outcomes were organized using the RE-AIM framework.

**Findings:** Patients embraced the technology; nearly 60% of enrolled patients were actively using the system after 12 months, a remarkably high rate when compared to normal standards of mHealth use. Patients also got better: 6-month rates of risky drinking and illicit drug use declined by 44% and 34%, respectively. However, clinics have struggled to maintain use of Seva since grant funding ended.

**Implications for D&I Research:** Implementation, dissemination and sustainability are major issues in healthcare and in virtually every industry. In a sense change is easy. Every day millions of people decide to quit smoking, lose weight, exercise more, etc. And they do well for a week or two. The real challenge is sustainability. The same is true for organizational change. Engineering approaches suggest that efforts to implement and sustain change must be based on principles of mass production and automation. Traditionally these efforts have been heavily labor-based. Widespread dissemination often means that many implementers need to be involved. Some are outstanding. Others are not. Some champions stay. Some leave. Some lose interest. Key employees leave, and their departures highlight the need for newly adopted practices to be incorporated in employee training. Implementation efforts will vary in fidelity and results. The longer implementation takes, the more at risk sustainability efforts will be. However, there are parts of any implementation that can and must be automated in order for delays between “bench and bedside” to be significantly reduced. This presentation will describe ways we can engineer implementation and in doing so, speed adoption and sustainability.

**Primary Funding Source:** National Institutes of Health

### S33 Specifying implementation strategies used by seven primary care regional cooperatives: real-world meets theory

#### Cynthia Perry^1,2^, Jennifer Hemler^3^, Laura Damschroder^4^, Tanisha Tate Woodson^1^, Sarah Ono^1,5^, Deborah Cohen^1^

##### ^1^ Oregon Health & Science University, Portland, OR, USA; ^2^ VA Ann Arbor Healthcare System, Ann Arbor, OR, USA; ^3^ Rutgers Robert Wood Johnson Medical School, New Brunswick, NJ, USA; ^4^ Implementation Pathways, LLC, Ann Arbor, MI, USA; ^5^ VA Portland Health Care System, Department of Veterans Affairs, Portland, OR, USA

###### **Correspondence:** Cynthia Perry (perryci@ohsu.edu)

**Background:** Seven regional Cooperatives participate in the Agency for Healthcare Research and Quality’s EvidenceNOW initiative. Cooperatives are working with approximately 1500 small-to-medium-sized primary care practices across 12 states to improve cardiovascular preventive care and practice capacity. Cooperatives designed multi-component interventions to assist practices in their improvement efforts. AHRQ funded ESCALATES to conduct the National Evaluation of EvidenceNOW. The purpose of this work is two-fold: (1) to identify the implementation strategies used by the 7 Cooperatives and (2) use these data to empirically test a common theoretical framework for dissemination and implementation.

**Methods:** We analyzed data, using an inductive analytic approach, to identify Cooperatives’ implementation support strategies and activities, drawing upon multiple qualitative data sources: Cooperatives’ grant proposals, observations and interviews from annual Cooperative site visits, and Cooperative-made entries to an online diary we created for this project. Cooperatives provided feedback, and we refined our findings as needed. Next, four ESCALATES researchers mapped the inductively-derived activities of Cooperatives’ implementation support to the Expert Recommendations for Implementing Change (ERIC) framework with its 9 clusters of 73 strategies.

**Findings:** Cooperatives’ implementation support used ERIC strategies across all nine ERIC clusters of strategies. We needed to create new terminology to fully capture forms of support that are not represented in ERIC: for instance, “Data Infrastructure” is not fully described within ERIC, although some components are included in multiple strategies across four different ERIC clusters. These discrete strategies may better reflect the reality of on-the-ground implementation if they were organized under a single cluster. Furthermore, Cooperatives’ Facilitation includes use of ERIC implementation strategies from 8 different ERIC clusters; this strategy is an overarching strategy that relies on other more narrowly focused strategies. We offer refined ERIC definitions, identify new strategies, and reorganize ERIC clusters to better align with implementation strategies as they occur in the real world.

**Implications for D&I Research:** Qualitative data collected during EvidenceNOW, a large dissemination and implementation initiative, was critical to testing and refining the ERIC framework. This work advances Implementation Science by using empirical data to refine a commonly used theoretical structure of implementation strategies and strengthens it for real-world application.

**Primary Funding Source:** Agency for Healthcare Research and Quality

### S34 Advancing the literature on designing audit and feedback interventions: identifying theory-informed hypotheses

#### Heather Colquhoun^1^, Kelly Carroll^2^, Kevin Eva^3^, Jeremy Grimshaw^2,4^, Noah Ivers^5^, Susan Michie^6^, Anne Sales^7^, Jamie Brehaut^2^

##### ^1^ University of Toronto, Toronto, ON, Canada; ^2^ Ottawa Hospital Research Institute, Ottawa, ON, Canada; ^3^ University of British Columbia, Vancouver, BC, Canada; ^4^ University of Ottawa, Ottawa, ON, Canada; ^5^ Women's College Hospital - University of Toronto, Toronto, ON, Canada; ^6^ UCL Centre for Behaviour Change, University College London, London, United Kingdom; ^7^ VA Ann Arbor Healthcare System, Ann Arbor, MI, USA

###### **Correspondence:** Heather Colquhoun (heather.colquhoun@utoronto.ca)

**Background:** Audit and Feedback (A&F) is one of the most common strategies for helping health providers to implement evidence into practice. Despite being extensively studied, health care A&F interventions remain variably effective, with overall effect sizes that have not improved since 2003. The design of most health care A&F interventions has not been informed by substantial theoretical understanding of feedback from other areas of social science. We are seeking to bring this relevant knowledge to the study of healthcare A&F interventions in order to advance a stagnant literature. The objective of this study was to develop a comprehensive list of testable, theory-informed hypotheses about designing effective A&F interventions.

**Methods:** Semi-structured and in-depth interviews with theory experts from a range of disciplines (i.e., cognitive psychology, medical education, medical decision-making, industrial/organizational psychology, management, economics) were conducted. Over the course of 60-90 minute telephone interviews, and guided by detailed descriptions of several typical A&F interventions from the health care literature, interviewees described how they would approach the problem of designing better A&F interventions. Specific and theory-informed hypotheses were elicited from the interviews and member checked. The resulting hypotheses were independently assigned into themes in an iterative process by 3 coders.

**Findings:** We conducted 28 interviews. The volume and scope of identified hypotheses were large: we identified 313 unique theory-informed hypotheses and coded them into 30 themes. The 30 themes included hypotheses related to the following five categories: A&F recipient (seven themes), content of the A&F (ten themes), process of delivery of the A&F (six themes), behaviour that was the focus of the A&F (three themes), and other (4 themes).

**Implications for D&I Research:** Using a novel methodology and the aim to invigorate a stagnant science, we have identified a broad range of testable, theory-informed hypotheses that suggest ways to improve A&F interventions. Our approach of interviewing theorists from a broad range of behavioural and social science yielded significant depth and breadth of hypotheses. This work will serve as the foundation for a prioritization exercise to determine which hypotheses should be tested in future A&F trials, and has the potential to accelerate intervention optimization.

**Primary Funding Source:** Canadian Institutes of Health Research

### S35 A logic model for precision medicine implementation research

#### Maren Scheuner, Jane Peredo, Catherine Chanfreau

##### VA Greater Los Angeles Healthcare System, Los Angeles, CA, USA

###### **Correspondence:** Maren Scheuner (maren.scheuner@va.gov)

**Background**: Precision medicine (PM) promises to improve health through better diagnosis, prognosis, risk assessment and management. However, PM has not been systematically adopted because of its complexity, limited knowledge and expertise of most clinicians, and organizational barriers. Nonetheless, the demand for PM is increasing,

**Methods**: A Department of Veterans Affairs (VA)-sponsored conference was held to foster partnerships between key stakeholders for agenda-setting to impact PM policy, research, and delivery. About 80 individuals participated representing patients, clinicians, researchers, informatics experts, policy-makers, ethicists, and administrators/managers from VA and non-VA. An online survey about the value of PM outcomes was emailed to invitees before the conference; results were used to kick-off the discussion. Seven panels with 46 speakers and 4 moderators addressed the challenges, opportunities, and strategies pertaining to clinical genetics/genomics, population-based PM programs, ethics and equity, research, and perspectives of patients, policy-makers, and organizational leadership. Discussions were recorded and transcribed with dual independent analysis. Quotes describing challenges and opportunities were mapped to the Consolidated Framework for Implementation Research (CFIR), and strategies to the Expert Recommendations for Implementing Change (ERIC) compilation of implementation strategies.

**Findings**: 316 quotes described 89 challenges, 89 opportunities, and 138 strategies organized across 11 PM domains: dissemination and implementation (61), informatics (52), organizational/provider (38), outcomes (38), confluence of clinical practice and research (33), partnerships (31), ethics/equity (24), Veteran engagement (17), economics (12), and translational research (10). We identified 25 CFIR constructs across all 5 domains: 11 with challenges > opportunities; 9 with opportunities > challenges; and 5 with both equally. We identified 40 implementation strategies across all 9 ERIC categories: 16 high-importance/high-feasibility; 5 low-importance/high-feasibility; 6 high-importance/low-feasibility; and 13 low-importance/low-feasibility. We constructed a logic model consisting of assumptions, inputs (infrastructure, big data, resources); activities (PM practice, research, education), outputs (genetic diagnosis, learning system), outcomes (utility, health care utilization), and impacts (PM value, equity & access, economic indicators).

**Implications for D&I Research:** We have created a logic model with multi-stakeholder input that can be used for PM implementation research planning. Existing frameworks appear to be suitable for implementation research in PM, including CFIR and the ERIC compilation of implementation strategies.

**Primary Funding Source:** Department of Veterans Affairs

### S36 The identification of valuable implementation science constructs among US federal agencies

#### Allison Dymnicki^1^, David Osher^1^, Abraham Wandersman^1,2^, Robin Bzura^1^, Michelle Boyd^3^, Amanda Cash^3^, Daniel Duplantier^3^, Lindsey Hutchison^3^

##### ^1^ American Institutes for Research, Washington, DC, USA; ^2^ University of South Carolina, Columbia, SC, USA; ^3^ Office of the Assistant Secretary for Planning and Evaluation, U.S. Department of Health and Human Services, Washington, DC, USA

###### **Correspondence:** Allison Dymnicki (adymnicki@air.org)

**Background:** Federal agencies in the United States have been interested in understanding innovative approaches for disseminating and implementing results from dissemination and implementation (D&I) studies. Multiple initiatives led by the Department of Health and Human Services (DHHS) have been designed to increase the department’s knowledge and integration of findings from D&I studies in different program areas. The Office of the Assistant Secretary for Planning and Evaluation (ASPE) within DHHS has been engaged in efforts to understand more about factors associated with successful implementation of federally funded initiatives. The goal for the current contract, awarded to the American Institutes for Research (AIR), was to identify a set of D&I constructs that will help federal staff select, support, and monitor grantees in federally funded initiatives.

**Methods:** AIR completed three phases of research, with the guidance of ASPE, to identify the set of D&I constructs. First, we conducted a systematic environmental scan (where over 1600 abstracts were reviewed and 125 articles were coded) to identify the most studied D&I constructs. Second, we conducted 18 individual or group interviews with federal and non-federal experts about the results of the environmental scan with the goal of validating its findings. Third, we convened 16 individuals representing seven divisions within Department of Health and Human Services and 1 additional federal agency using a Delphi process. This approach helped the group reach consensus regarding a set of D&I constructs that they find useful when selecting, supporting, and monitoring grantees in federally funded initiatives.

**Findings:** Results from this three phase approach led to the identification of 11 D&I constructs, which will be defined and described during the presentation. These were: evidence strength, evidence relevance, implementation complexity, general capacity, intervention-specific capacity, perceived advantage, contextual fit, adaptation, fidelity of implementation, theoretical clarity, and data driven quality improvement.

**Implications for D&I Research:** This project’s findings highlight an innovative approach used to share findings from D&I research that has the potential to advance the extent to which these ideas are incorporated into the practices and policies of federal agencies and the systems in which they work.

**Primary Funding Source:** Office of the Assistant Secretary for Planning and Evaluation

### S37 Building future capacity for D&I research: results from a mentored training program

#### Margaret Padek^1^, Rebekah Jacob^1^, Melissa Franco^1^, Jon Kerner^2^, Anne Sales^3^, Enola Proctor^4^, Maureen Dobbins^5^, Ross Brownson^1,4,6^

##### ^1^ Prevention Research Center, Washington University in St. Louis, St. Louis, MO, USA; ^2^ Canadian Partnership Against Cancer, Toronto, ON, Canada; ^3^ VA Ann Arbor Healthcare System, Ann Arbor, MI, USA; ^4^ Brown School of Social Work, Washington University in St. Louis, St. Louis, MO, USA; ^5^ McMaster University, Hamilton, ON, Canada; ^6^ Alvin J. Siteman Cancer Center, Washington University School of Medicine, St. Louis, MO, USA

###### **Correspondence:** Margaret Padek (mpadek@wustl.edu)

**Background:** With the demand for D&I research capacity growing, there has been an emergence of training programs in various formats. While many have different disciplinary foci, trainee level, and time commitment, few have looked at the effectiveness of increasing D&I skills of their trainees. The Mentored Training in Dissemination and Implementation in Cancer (MT-DIRC) is a 2-year, NIH-funded fellowship that focuses on the mentoring aspect of training relationships and assesses skill changes among its fellows during their time in the program. This presentation will examine two cohorts’ skill acquisition over their time in the program and their overall mentoring connections.

**Methods:** D&I skills surveys were administered to doctorally-prepared MT-DIRC trainees at three time points (pre-program, then 6 months, and 18 months’ post initial institute attendance). Trainees rated their skill level on a 5-point Likert scale for 43 unique D&I research competencies. An additional survey identified their social network connections. Repeated measures ANOVA was used to examine skill improvements over time and Pearson’s correlation to examine relationships with D&I skills and number of mentoring connections.

**Findings:** Two cohorts (N= 26) have completed their training in the program and all sets of evaluations. In the aggregate, all 43 skills have shown significant improvement (p<0.05) from pre-test to 18 months with large effect size (Cohen’s d> 0.8 for all competencies). Example skills with particularly large improvements include: “Describe a range of D&I strategies, models, and frameworks” and “Identify common D&I measures & analytic strategies for your research questions.” Moderate correlation (r=.438) existed between the number of mentor connections (with faculty and other fellows) and D&I skills.

**Implications for D&I Research:** Fellows consistently indicated that they were more skilled toward the end of the 2-year training. Network data showed a positive relationship between number of mentor connections with D&I skill acquisition. Although the MT-DIRC program shows evidence of effectiveness in building skills, further work is needed to compare the effectiveness across the different training formats (e.g., in person delivery vs. distance learning). Mentored training in D&I science can build needed research capacity and is a promising strategy for future efforts in enhancing D&I research capacity.

**Primary Funding Source:** National Institutes of Health

### S38 Using an implementation framework to build implementation science capacity in Africa: the UNC/wits university collaboration

#### Rohit Ramaswamy^1^, Jabulani Ncayiyana^2^, Latifat Ibisomi^2^, Tobias Chirwa^2^, Kathryn Salisbury^3^, Audrey Pettifor^1^

##### ^1^ Gillings School of Public Health, University of North Carolina, Chapel Hill, NC, USA; ^2^ School of Public Health, University of the Witwatersrand, Johannesburg, South Africa; ^3^ Institute of Global Health and Infectious Diseases, University of North Carolina, Chapel Hill, NC, USA

###### **Correspondence:** Rohit Ramaswamy (ramaswam@email.unc.edu)

**Background:** Despite significant progress, HIV, Malaria and TB still resulted in over 1.6 million deaths in Africa in 2015, and millions more bear the burden of neglected tropical diseases (NTDs). African leaders have resolved to end AIDS, TB and Malaria by 2030 through better implementation and transformed health services. Critical to this is the capacity building of sufficient number of people to conduct implementation research (IR) to unblock barriers to program implementation. In 2016, Wits University in South Africa partnered with UNC Chapel Hill to develop MSc and PhD programs in Implementation Science (IS) to train African students to conduct IR in their home countries. Thirty students from ten African countries have enrolled since program inception.

**Methods:** The three systems of the Interactive Systems Framework guided program development. The *synthesis and translation system* involved the adaption of UNC content to the two-week format of the Wits curriculum. Five courses, logically sequenced across the research-practice continuum and jointly taught, constitute the core, with two courses focused specifically on IR content. The *delivery system* used a blended instructional model of online and face-to-face delivery for each course. Substantial attention is focused on the *support system* to ensure application of learning. Students developed IR project in their home countries under dual supervision of Wits faculty and local supervisors who were also trained on IR by UNC/Wits faculty. Students with promising research projects were selected for intensive coaching by UNC/Wits faculty, delivered through site visits and online.

**Findings:** Program evaluation used the four levels of the Kirkpatrick model. *Reaction* was positive with average evaluation scores of 4.7 out of 5 for the two content courses. *Learning* was demonstrated by 97% of students passing these courses. *Application* was demonstrated by thirteen completed theses researching implementation issues affecting delivery of HIV, TB, Malaria and NTDs interventions across Africa and student presentations at several global forums. While early to assess *impact*, several student projects address their governments’ implementation priorities.

**Implications for D&I Research:** Using implementation science approaches results in effective IR capacity building programs. Further research is needed to identify optimal strategies to make such programs available to more participants.

**Primary Funding Source:** National Institutes of Health

### S39 Curricular thinking in implementation science: a two course sequence with experiential learning

#### Anne Sales^1,2^, Gretchen Piatt^1^

##### ^1^ University of Michigan, Ann Arbor, MI, USA; ^2^ VA Ann Arbor Healthcare System, Ann Arbor, MI, USA

###### **Correspondence:** Anne Sales (salesann@umich.edu)

**Background:** The Health Infrastructures and Learning Systems (HILS) MS and PhD programs are first in the world, newly developed programs at the University of Michigan, designed to educate practitioners and scientists in learning health systems. Implementation science and practice are critical components of this emerging field; however, curricula are poorly developed, with many training programs focusing on methods of study design and evaluation rather than on core elements of implementation methods. Competencies exist, but are not well developed, and tend to be topical rather than oriented toward systematic development of knowledge, skills and attitudes. We propose to articulate and describe a two course sequence in implementation science in order to begin discussion of curriculum development in this emerging field.

**Methods:** Using peer-reviewed literature on existing training programs in implementation and dissemination sciences, and experience as both instructors and recipients of existing training programs, we developed a structured curriculum that incorporates use of process, determinants, and evaluation frameworks. The two-semester curriculum follows a systematic approach to articulating care gaps, finding evidence based solutions, mapping processes and assessing key determinants of implementation success, and designing implementation interventions to address negative determinants. Topics and techniques are scaffolded and increase in complexity through the course sequence.

**Findings:** Seven students matriculated through the first course in the sequence, offered in Spring 2017. Five are currently completing a summer implementation project focused on utilizing course learnings to develop and implement an initiative that addresses a current gap in care. Following their summer projects, these students will take the second course in Fall 2017, in which they will debrief their summer projects and learn more advanced topics in implementation research. Projects range from improving health data quality in Malawi to decreasing unplanned ED visits after bariatric surgery.

**Implications for D&I Research:** Articulating a clearly developed curriculum in a field that is dominated by short, episodic, and topically-focused training is complex. The primary goal of this presentation is to spark discussion among researchers involved in education and training to promote more systematic thinking and planning of curriculum in this important area.

### S40 Fostering international collaborations in implementation science

#### Gregory Aarons^1,2^, Amy Green^1^, Joanna Moullin^1,3^, Mark Ehrhart^4,5^, Simon Ducarroz^6^, Nick Sevdalis^7^, Henna Hasson^8,9^, Ulrica von Thiele Schwarz^8,10^, Sigrid James^11^, Cathleen Willging^12^

##### ^1^ University of California, San Diego, La Jolla, CA, USA; ^2^ Child and Adolescent Services Research Center, San Diego, CA, USA; ^3^ University of Technology Sydney, Sydney, Australia; ^4^ San Diego State University, San Diego, CA, USA; ^5^ University of Central Florida, Orlando, FL, USA; ^6^ Centre Hygée – Centre régional de Prévention des cancers, Saint-Priest-en-Jarez, France; ^7^ King's College London, London, United Kingdom; ^8^ Karolinska Institute, Stockholm, Sweden; ^9^ Center for Epidemiology and Community Medicine, Stockholm County Council, Stockholm, Sweden; ^10^ School of Health, Care and Social Welfare, Mälardalen University, Västerås, Sweden; ^11^ University of Kassel, Kassel, Germany; ^12^ Behavioral Health Research Center of the Southwest, Pacific Institute for Research and Evaluation, Albuquerque, NM, USA

###### **Correspondence:** Gregory Aarons (gaarons@ucsd.edu)

**Background:** Philosophical, scientific, cultural, and historical legacies shape national contexts in which Implementation Science (IS) develops and proceeds. Collaborations of IS researchers across national boundaries may foster a greater understanding of and attention to the unique issues and challenges that impact the conduct of IS research in diverse settings, while also enhancing external validity. However, little is known about the conditions that may pragmatically facilitate international collaborations in IS. The goal of the present study is to identify factors likely to advance fruitful and productive international collaborations in implementation research.

**Methods:** The authors participated in a 3-day symposium in the Pays De La Loire region of France. The group included IS researchers from France, Germany, Sweden, the United Kingdom, Australia, and the United States, with experience in IS in those countries and Spain, Norway, Switzerland, Mexico, and sub-Saharan Africa. Concept mapping (CM) was used to identify and prioritize key issues in international collaboration in IS. CM steps include: developing a focus question; brainstorming issues related to the focus question; sorting and rating statements; multidimensional scaling and hierarchical cluster analysis to identify common themes; and interpreting results. Although CM has been applied in IS-related studies, to our knowledge the cultivation of international collaborations in IS has not been addressed.

**Findings:** Developed through participatory means, the authors agreed on the following focus question: “What are the ways to foster international collaboration in IS in health and social care?” Fifty-six unique statements were developed. Preliminary analyses identified several predominant themes including, but not limited to, knowledge exchange and resource sharing (e.g., data, measures), funding for international projects, development of special interest groups/conferences, post- and pre-doctoral supervision and mentorship across countries, maximizing training opportunities (e.g., master classes, fellowships), use of technology (e.g., video conferencing) to promote engagement, inclusion of researchers (with appropriate expertise) from other countries, and adoption of well-defined deliverables (e.g., manuscripts, grant applications). Analyses will be complete by December 2017.

**Implications for D&I Research:** International collaborations have the potential to shape conditions likely to improve the quality and external validity of IS research, nurture new and early career researchers, and expand the scope and network of engaged implementation scientists.

**Primary Funding Source:** Borchard Foundation

### S41 Using formal training to transfer evidence-based implementation strategy knowledge and skills

#### JoAnn Kirchner^1,2^, Jeffrey Smith^1,2^, Katherine Dollar^3^, Mona Ritchie^1,2^, Lindsay Martin^4,5^, Terri Barrera^4,5^, Jan Lindsay^4,5^

##### ^1^ Central Arkansas Veterans Healthcare System, Department of Veterans Affairs, North Little Rock, AR, USA; ^2^ University of Arkansas for Medical Sciences, Little Rock, AR, USA; ^3^ Syracuse VA Medical Center, VA Center for Integrated Healthcare, Syracuse, NY, USA; ^4^ Michael E. DeBakey VA Medical Center, VA Center for Innovation in Quality, Effectiveness and Safety, Houston, TX, USA; ^5^ Baylor College of Medicine, Houston, TX, USA

###### **Correspondence:** Katherine Dollar (katherine.dollar@va.gov)

**Background:** Facilitation, an evidence based implementation strategy, has been applied in multiple contexts to implement innovations with varying complexity. However, little is known about how to transfer the complex skills needed to conduct implementation facilitation (IF) across health care settings. In this presentation, we will describe an IF training program, challenges and changes over time, and our evaluation of recent trainings.

**Methods:** In response to a request from a clinical partner, in 2011, we developed a two-day training program and companion manual to support IF scale up and spread. We recently revised the manual and training based on current research and emerging tools to support its application. Training components include IF knowledge, skills and core competencies; facilitator roles and activities across the implementation phases; products and processes that support facilitation; practical tips to ensure success; roleplaying challenging situations; group problem solving and IF application to the participant’s project. To evaluate the training program, an independent group conducted participant surveys in the first two cohorts of our revised training. Surveys were conducted prior to, immediately following, and 6 months after the revised IF training to document perceived and experienced IF knowledge and skills. This was supplemented with 6-month post training semi-structured interviews to document facilitators experiences with facilitation.

**Findings:** Since 2011, we have trained over 100 participants including clinical leaders, researchers who plan to apply IF in their own projects, and clinical facilitators. These trainings have supported the application of facilitation in more than four national clinical initiatives and 15 research programs. Over 20 participants completed the quantitative survey associated with our revised training program. Preliminary findings indicate substantial change in participant’s perceived IF knowledge and skills before and after the training. Six month surveys and interviews are currently being collected. We will report descriptive findings from these surveys as well as themes that emerged from the 6 month interviews.

**Implications for D&I Research:** Scholars have called for implementation researchers to address the handoff of evidence-based strategies to front line managers, policy makers and providers. This training program provides an example through which scale up and spread of implementation facilitation has been accomplished over the past six years.

**Primary Funding Source:** Department of Veterans Affairs

## Clinical Care Settings: Patient-level Interventions

### S42 Counterbalancing measures: identifying unintended consequences

#### David Aron^1,2^, Chin-Lin Tseng^3^, Orysya Soroka^3^, Leonard Pogach^4^

##### ^1^ Louis Stokes Cleveland VA Medical Center, Cleveland, OH, USA; ^2^ Case Western Reserve University School of Medicine, Cleveland, OH, USA; ^3^ VA New Jersey Health Care System, East Orange, NJ, USA; ^4^ Veterans Health Administration, Washington, DC, USA

###### **Correspondence:** David Aron (david.aron@va.gov)

**Background:** The concept of balancing measures is well known, but there are few empirical studies within narrow domains, e.g., glycemic control. Recent initiatives have focused on reduction in inappropriately intensive glycemic control (overtreatment). Our objective was to determine whether reductions in overtreatment were associated with increased undertreatment.

**Methods:** This pre-test/post-test study used cross-sectional Veterans Health Administration administrative data from calendar years (CYs) 2013 and 2016. Our primary outcome measure was facility level overtreatment rate in patients identified as the at-risk group (patients with A1c<6.0% and known risk factors: taking a diabetes drug known to have a relatively high frequency of hypoglycemia (insulin and/or sulfonylurea agents) plus having at least one of the following additional criteria: age 75 years or older, chronic kidney disease (defined as last serum creatinine measurement in a year greater than 2.0mg/dL or an *ICD-9-CM* diagnosis of cognitive impairment or dementia in ambulatory care. Undertreatment was measured as the proportion of patients with A1C>9%.

**Findings:** There was marked variation; facility overtreatment rates ranged from 3.54-20.64% and 3.70-15.75% at the facility level in 2013 and 2016, respectively. Overtreatment rates (mean±SD) fell from 9.15+2.94% to 8.37+2.34% (p<0.001). Undertreatment rates ranged from 5.76-16.86% and 6.80-18.68%. The Undertreatment rates mean (±SD) rose from 10.32+2.21% to 11.04+2.38% (p=0.001). Overtreatment rates were reduced in 57.2% of facilities; undertreatment rates were reduced in 42.0%. Both were reduced in 15.9%.

Over- and undertreatment rates were not correlated, but the absolute change in the overtreatment rate from 2013 to 2016 was inversely correlated with the change in the undertreatment rate (r= -0.405, p<0.001). The relative change in the overtreatment rate was inversely correlated with the relative change in the undertreatment rate (r= -0.342, p<0.001).

**Implications for D&I Research:** Our study indicates that the promotion of reduction of overtreatment may be associated with an increase in undertreatment in the same population of patients with diabetes. When adverse unintended consequences can be anticipated, it is incumbent upon systems to include mitigating actions such as counterbalancing measures to ensure that unintended harms are avoided.

**Primary Funding Source:** Department of Veterans Affairs

### S43 Sustainability of the Legacy for Children™ evidence-based parenting program in primary care and early childhood education: comparative case study

#### Marvin So, Akilah Heggs, Sophie Hartwig, Lara Robinson

##### Centers for Disease Control and Prevention, Atlanta, GA, USA

###### **Correspondence:** Marvin So (kun8@cdc.gov)

**Background:** Research suggests that many disparities in overall health are rooted in early childhood; the experience of poverty early in development has been associated with adverse health outcomes across the lifespan. Evidence-based, parent-focused preventive interventions such as Legacy for Children^TM^ (*Legacy*) can improve developmental outcomes for at-risk children. The public health impact of parenting programs could be substantial if these programs are successfully disseminated and sustained within primary care (PC) and other community-based settings. We employed a comparative case study approach to explore factors influencing sustainment of *Legacy* in distinct organizational contexts: two PC sites and two early childhood education (ECE) sites.

**Methods:** We examined quantitative and open-ended questions from organizational surveys completed by *Legacy* site representatives one year following initial implementation. These data were organized into analytic matrices according to the Sustainability stage of Fixsen et al.’s (2005) Implementation Stages framework. We used cross-case synthesis to identify how sites differentially perceive *Legacy*’s long-term sustainability.

**Findings:** Implementation barriers reported consistently across site types included: logistical/administrative support, collaboration with other agencies, and staff turnover. Challenges with participant schedule conflicts, transportation, and leaving the service area were noted as reasons for participant attrition. Differentiating PC findings suggest *Legacy* helps improve participant engagement with agency (e.g., increased use of co-located services) and meet agency goals of improving positive parenting practices. Distinguishing features of ECE sites included: the utilization of diverse strategies to retain participants, staff workload impeding implementation, and the implementation cost of childcare. All sites recommended expanding *Legacy* into other agencies and offered unique recommendations to facilitate implementation. However, few local actions were taken to support sustainability in year one.

**Implications for D&I Research:** Further research to understand the factors that impact program sustainability would help tailor dissemination of evidence-based parenting programs into organizations where families routinely access services, such as PC and ECE centers. To promote *Legacy*’s sustainability within these diverse contexts, we have developed sustainability tools and trainings for site use during pre-implementation and implementation phases. This presentation will explore how monitoring and resources for sustainability can be applied by stakeholders to promote health within clinical and non-clinical settings.

**Primary Funding Source:** Centers for Disease Control and Prevention

### S44 Team approaches to transforming inpatient heart failure care

#### Erin Blakeney, Kevin O'Brien, Susan Pambianco, Leah Spacciante, Danielle Lavallee, Brenda Zierler

##### University of Washington, Seattle, WA, USA

###### **Correspondence:** Erin Blakeney (erin2@uw.edu)

**Background:** Structured interprofessional bedside rounds (SIBR) have been associated with positive improvements in health care team communication, care quality, and outcomes. Our goal was to improve care team and patient outcomes through adaptation and implementation of a SIBR model through an Academic-Practice Partnership between School of Nursing and School of Medicine-based health services researchers and heart failure teams at an Academic Medical Center.

**Methods:** Since receiving HRSA funding in 2014, members of our Academic-Practice Partnership have been working collaboratively to implement a SIBR model on two cardiology units providing care to patients with heart failure. A systematic approach was used to adapt and implement the model, including: facilitation, data feedback to facilitate small tests of change, ongoing consultation, and leadership development. The project started with formation of an interprofessional Change Team from participating units and mixed methods data collection, including annual validated team surveys (i.e. Relational Coordination Survey) and periodic observations of rounds. Change and grant teams collaborated to adapt and implement the SIBR model. Beginning in 2015, targeted team training utilizing TeamSTEPPS concepts and SIBR simulation facilitated sequential implementation in both units.

**Findings:** Annual surveys and periodic observations revealed positive changes. Statistically significant improvements between baseline and follow up were demonstrated for each of the Relational Coordination Survey’s seven dimensions (e.g. Timely Communication, Mutual Respect). Observation of rounds (n=405) identified increases in both nurse participation (from 21% to 75%) and frequency of rounds occurring in patient rooms (from 15% to 93%). Qualitative improvements in team communication and care quality were also observed. In the 2017 team survey, respondents (n=106) were asked whether “Interprofessional bedside rounds add value” for various members of the care team. Respondents indicated that the model added value for them in their work role (86%), for patients (93%), for family members/caregivers (94%) and for the interprofessional care team (92%).

**Implications for D&I Research:** Collaborative development and implementation of SIBR holds great potential for improving interprofessional team communication, relationships, and care quality. Engaged change teams, regular leadership workshops, and objective measurements of progress provided important supports for sustaining and iterating work process changes and strengthened the Academic-Practice Partnership.

**Primary Funding Source:** Health Resources and Services Administration

### S45 Improving care for pregnant women with opioid use disorders: a learning collaborative approach to best practice implementation

#### Daisy Goodman^1,2^, Alexandra Zagaria^1^

##### ^1^ Dartmouth Institute for Health Policy and Clinical Practice, Geisel School of Medicine, Lebanon, NH, USA; ^2^ Dartmouth Hitchcock Medical Center, Lebanon, NH, USA

###### **Correspondence:** Daisy Goodman (daisy.j.goodman@dartmouth.edu)

**Background:** Opioid use disorder (OUD) has reached epidemic proportions among pregnant women the United States. Northern New England is among the most severely impacted regions, with 5-8% of pregnancies across Maine, New Hampshire and Vermont affected. Untreated, maternal OUD is associated with poor maternal and neonatal outcomes, including risk for infectious disease, overdose, premature birth, and neonatal withdrawal. Concurrent tobacco use disorder is present in 95% of this population, contributing to adverse outcomes. Hepatitis C is also common with implications for both neonatal and maternal health. Although evidence-based guidelines for the care of substance-affected pregnancies exist, the quality of clinical care provided varies widely. This pilot (1) developed and implemented a checklist and toolkit to standardize practice in the care of pregnant women with OUD, and (2) explored contextual factors contributing to quality gaps at 8 diverse maternity care sites across the three states.

**Methods:** We surveyed regional maternal-child health providers and interviewed representative patients to identify key areas of need. A checklist and toolkit were developed based on stakeholder input and implemented at partner sites using healthcare process improvement methods. A learning collaborative framework was used to support implementation. We collected baseline data from 8 sites on 30 process and outcome measures, including hepatitis C screening rate, treatment of concurrent psychiatric disorders, naloxone distribution, tobacco use and treatment, birthweight, gestational age at delivery, prenatal substance exposure, and breastfeeding.

**Findings:** Perinatal OUD is highly prevalent at participating sites (4-13%). Wide variation existed in prenatal care for women with OUD at baseline, and in maternal and neonatal outcomes. Preliminary comparisons from pre- to post-checklist implementation trend towards improvement in rates of hepatitis C screening (89.4% vs 97.56%, p=.128), provider discussion of Naloxone (10% vs 25%, p=0.059); breastfeeding education (57% to 73%, p=0.007); and tobacco cessation treatment (68% to 75%, p=0.029).

**Implications for D&I Research:** These findings demonstrate the potential of simple tools and learning collaboratives to improve management of perinatal OUD. Further research is needed on strategies to strengthen the impact and reach of implementation supports to transform care and outcomes for this vulnerable population of women and their infants.

**Primary Funding Source:** The March of Dimes

### S46 Structured implementation of surgical safety checklists to facilitate teamwork and behavior change to reduce postoperative mortality: safe surgery 2015 South Carolina

#### William Berry^1,2^, Lizabeth Edmondson^1^, Stuart Lipsitz^1^, George Molina^1,3^, Bridget Neville^1^, Sara Singer^2,3^, Aunyika Moonan^4^, Ashley Kay Childers^4,5^, Richard Foster^4^, Lorri Gibbons^4^, Atul Gawande^1,2^, Alex Haynes^3^

##### ^1^Ariadne Labs, Boston, MA, USA; ^2^ Harvard T.H. Chan School of Public Health, Boston, MA, USA; ^3^ Massachusetts General Hospital/ Harvard Medical School, Boston, MA, USA; ^4^ South Carolina Hospital Association, Columbia, SC, USA; ^5^ Clemson University, Clemson, SC, USA

###### **Correspondence:** William Berry (wberry@ariadnelabs.org)

**Background:** Surgical safety checklists have been proven to reduce postoperative complications and mortality. However, population-level implementation has demonstrated uneven results. Recognizing that effective use of this tool requires behavioral changes by surgical teams, a twelve-part program that based on prior experience introducing team-based tools into complex clinical environments was developed and deployed within the context of the Safe Surgery 2015 South Carolina program.

**Methods:** The program was made available to all hospitals performing surgery in the state of South Carolina. Participation was voluntary and each hospital formed a leadership team including both clinical and executive leaders. Formal assessment of patient safety culture was performed and the checklist modified to fit local needs in response to local feedback after piloting. Teams were trained in checklist use and progress at each site evaluated through periodic observation and feedback. Webinars, in-person meetings, and site visits were used to support implementation. After implementation, repeat assessment of safety culture was performed. All-payer discharge claims were linked to the state registry of vital statistics to assess trends in 30-day postoperative mortality.

**Findings:** At baseline, there was an inverse correlation between safety culture and risk-adjusted postoperative mortality (p<0.05). Fourteen hospitals, accounting for nearly 40% of the statewide surgical volume completed the program. This cohort improved across all dimensions of teamwork (p<0.05). Hospitals completing the program had a reduction in risk-adjusted 30-day postoperative mortality from 3.38% in 2010 to 2.84% in 2013 (p<0.05), with no significant change among other hospitals. (p=0.3281).

**Implications for D&I Research:** Prior attempts to introduce surgical safety checklists into widespread use have often used regulatory mandate without clear guidance on implementation, resulting in disappointing outcomes. Understanding that checklists are not standalone tools, but rather behavior-change interventions in a complex clinical environment, stepwise, structured implementation leads to more meaningful use and integration into clinical care. This is reflected in the changes in safe surgical practices that parallel reduction in perioperative mortality in South Carolina. This model should be considered for similar initiatives in the future. Additional research is necessary to understand the precise mechanisms for behavior change engendered by the checklist and its implementation.

**Primary Funding Source:** AHRQ, Branta and Rx Foundations

### S47 Implementation planning of a mhealth vaccination tool: a workflow study in four pediatric clinics

#### Stephanie Staras^1^, Natalie Rich^1^, Esaa Samarah^1^, Lindsay Thompson^1^, Michael Muszynski^2^, Elizabeth Shenkman^1^

##### ^1^ University of Florida College of Medicine, Gainesville, FL, USA; ^2^ Florida State University College of Medicine, Orlando, FL, USA

###### **Correspondence:** Stephanie Staras (sstaras@ufl.edu)

**Background:** Many mHealth interventiosn are not successfully implemented because of a failure to incorporate into the existing clinical workflow. Existing mHealth workflow studies primarily assess workflow change following implementation. This study aimed to study workflow before mHealth implementation to determine the best timing and personnel for implementation.

**Methods:** We conducted a direct observation, time-and-motion study with 13 adolescent visits across four pediatric clinics in North and Central Florida. Two trained observers used separate (provider and patient) standard, time-stamped, electronic data collection forms adapted from the Arkansas Foundation for Medical Care’s Work Flow Assessment Checklist suggested by the Health and Human Services Agency for Healthcare Research and Quality. We constructed flowcharts for each clinic’s workflow. To confirm the accuracy of the constructed flow charts and identify the best timing and staff for mHealth implementation, we conducted semi-structured interviews at each clinic.

**Findings:** We observed similar workflow structure across all four clinics and visit type (acute/well). Patients at each clinic checked in with office staff, waited to be called from the waiting room by a nurse or medical assistant (time range = 1 to 60 minutes, mean = 13 minutes), spent time waiting alone for the provider (time range = 2 to 25 minutes, mean = 12 minutes), and confirmed follow-up appointments with the front office staff as needed. In total, patients spent an average of 24 minutes (range = 3 to 75 minutes) without staff contact. All sites confirmed the observed workflow. Three of the four sites recommended front office staff offer the intervention to patients in the waiting room with nurses checking that the system had been received at triage. The fourth site currently uses a mHealth system in the exam room during well visits and chose to implement our intervention simultaneously.

**Implications for D&I Research:** Despite differences in clinic location, size, ownership, and patient visit type, their similar workflow structure suggests only minor workflow tailoring may be needed for mHealth implementation across primary pediatric care clinics. Further, our use of workflow to plan mHealth implementation may serve as a generalizable model for future mHealth adoption and integration.

**Primary Funding Source:** National Institutes of Health

### S48 Planning for the technology revolution: case-study-based guidelines for the implementation of novel information technology applications within an integrated healthcare system

#### Julian Brunner^1,2^, Tannaz Moin^2,3^, Alison Hamilton^2,4^, Ariel Lang^5,6^, Sabine Oishi^2^, Bevanne Bean-Mayberry^2^, Melissa Farmer^2^, Dawn Glover^2^, Erin Finley^7,8^

##### ^1^ UCLA Fielding School of Public Health, Los Angeles, CA, USA; ^2^ VA Greater Los Angeles Healthcare System, Los Angeles, CA, USA; ^3^ David Geffen School of Medicine at UCLA, Los Angeles, CA, USA; ^4^ University of California Los Angeles, Los Angeles, CA, USA; ^5^ University of California, San Diego, San Diego, CA, USA; ^6^ VA San Diego Healthcare System, San Diego, CA, USA; ^7^ South Texas Veterans Health Care System, San Antonio, TX, USA; ^8^ University of Texas Health Science Center at San Antonio, San Antonio, TX, USA

###### **Correspondence:** Julian Brunner (julian.brunner@va.gov)

**Background:** Web- and server-based information technology (IT) applications have the potential to revolutionize healthcare delivery by facilitating information transfer, care coordination, and access to services. However, implementation of IT interventions presents unique challenges, including the need to ensure protection of patient data and integrate successfully with existing health information systems. In order to consider requirements for effective IT implementation, we conducted case studies of two IT implementation efforts from the Department of Veterans Affairs (VA) healthcare system to identify areas of focus for planning and development.

**Methods:** We developed case studies of two implementation efforts developed using the Replicating Effective Programs (REP) framework: 1) a quality improvement project offering online delivery of the Diabetes Prevention Program (DPP), a web-based intensive lifestyle intervention, tailored for women Veterans with prediabetes; and 2) a research study implementing VA Coordinated Anxiety Learning and Management (CALM), a web-based cognitive behavioral intervention tailored for use with Veterans. Data sources included patient and stakeholder interviews, patient surveys, and qualitative reflections with research team members.

**Findings:** Despite the IT applications’ divergent content and context, the two case studies faced similar challenges, each with relevance for implementation, sustainment, and spread. Challenges fell broadly into the following eight categories: 1) negotiating contracts and/or partnerships in acquiring rights and/or access; 2) ensuring adequate protections of privacy for patients and other users; 3) ensuring stability and reliability of applications across sites and users; 4) collecting and integrating user feedback on application design and utility; 5) scaling up application-related resources to meet unpredictable demands; 6) determining rights and requirements for application testing and change; 7) introducing the application to potential users and guiding them through technological challenges; and 8) planning for data management and reporting, including integration with existing data systems.

**Implications for D&I Research:** With increasing recognition of the utility of IT applications as powerful mechanisms for delivery and coordination of care has come growing need to identify effective solutions for implementation of health IT. The proposed guidelines are intended to aid in planning, evaluation, and scale-up of IT implementation within complex healthcare systems.

**Primary Funding Source:** Department of Veterans Affairs

### S49 Implementing ehealth interventions for people living with HIV: the critical role of privacy preferences

#### Stephanie Marhefka^1^, Elizabeth Lockhart^1^, DeAnne Turner^1^, Shanda Vereen^1^, M. Margaret Dolcini^2^, Robert Glueckauf^3^, Julie Baldwin^4^

##### ^1^ University of South Florida, Tampa, FL, USA; ^2^ School of Social and Behavioral Health Sciences, Oregon State University, Corvallis, OR, USA; ^3^ Florida State University, Tallahassee, FL, USA; ^4^ Center for Health Equity, Northern Arizona University, Center, AZ, USA

###### **Correspondence:** Stephanie Marhefka (smarhefk@health.usf.edu)

**Background:** ehealth interventions have potential to increase access to care and expand the reach of evidence based programs. However, delivery of ehealth programs to people living with HIV (PLH) may need to be tailored to account for the inherent privacy concerns related to living with a sensitive health condition. We report findings from a qualitative study examining implementation challenges, preferences, and delivery modality of ehealth programs among PLH in Florida.

**Methods:** Participants were recruited after completing a statewide quantitative survey that examined technology use among PLH in Florida. Findings from that study suggested most PLH have access to the technologies necessary to participate in ehealth programs (i.e., mobile phones, Internet, etc.) but delivery may be complicated due to privacy and confidentiality concerns related to living with HIV. Stratified by technology access, selected participants (N=22) completed an in-person semi-structured interview (~1 hour) to better understand how ehealth programs can be implemented within this population.

**Findings:** Most participants were familiar with, and appreciated, text messaging for appointment reminders. Several participants were excited about an app specifically for PLH. Some features of an example app were redundant with systems PLH already had in place (e.g. calendars), and thus deemed of limited added value. Interest in and preferences for program delivery seemed to depend primarily on degree of transparency regarding HIV status. Those open about their HIV status (i.e., willing to tell others they are PLH) preferred programs that were easiest to access (e.g., delivered via text-messaging or non-password-protected apps), whereas those who kept their HIV status private were willing to experience greater inconvenience (e.g., web-based portals; apps with logins) so as to protect their privacy; they preferred HIV-specific information not be provided with any unsecure technology, but were open to HIV-specific communication when using secure methods.

**Implications for D&I Research:**

Successful uptake of ehealth technologies for PLH may depend on the availability of options tailored to: a) those who are transparent about their HIV status; and b) those who are more private. Implementation strategies for ehealth programs for PLH should ensure PLH are able to choose options that best suit their needs and preferences.

**Primary Funding Source:** National Institutes of Health

### S50 Tailoring implementation strategies for cardiovascular disease risk calculator adoption in primary care practice: results of an EvidenceNOW educational outreach intervention

#### Laura-Mae Baldwin^1^, Michael Parchman^2^, Leah Tuzzio^2^, Erika Holden^2^, Jennifer Powell^3^, Allison Cole^1^

##### ^1^ University of Washington, Institute of Translational Health Sciences, Seattle, WA, USA; ^2^ Kaiser Permanente Washington Health Research Institute, Seattle, WA, USA; ^3^ Powell & Associates, LLC, Ashville, NC, USA

###### **Correspondence:** Laura-Mae Baldwin (lmb@uw.edu)

**Background:** Accelerating the adoption of evidence-based practices (EBP) into practice is critical to improving population health. The AHRQ EvidenceNOW initiative, Healthy Hearts Northwest (H2N), is one of the seven cooperatives designed to test implementation strategies. H2N investigators developed and implemented a virtual educational outreach intervention to increase use of CVD risk calculation and prescription of statins for prevention of CVD. Here we use data gathered from the outreach visits to identify evidence-based implementation strategies most likely to promote successful CVD calculator implementation.

**Methods:** Five educators conducted virtual educational outreach visits in 44 H2N practices. The educators completed field notes after each visit describing practices’ current experience with the CVD risk calculator, including barriers and facilitators to implementing the calculator. The H2N study team is conducting a qualitative analysis of the field notes using the EPIS (Exploration, Preparation, Implementation, and Sustainment) framework to describe the facilitators and barriers to CVD risk calculator implementation. The EPIS framework provides a conceptual approach for considering challenges and opportunities in EBP implementation. We will use concept mapping with a group of five implementation scientists to map results of the analysis to the 73 evidence-based implementation strategies described by Powell and colleagues that might be most effective to promote the use of the CVD risk calculator in primary care practice settings.

**Findings:** The study team has collated the data from the 44 sets of field notes and assigned preliminary codes for the barriers and facilitators to CVD risk calculator implementation. We will complete field notes coding by mid-September 2017. We will conduct the Delphi process with the implementation scientist group in October 2017, then compile our findings and develop our presentation in November 2017.

**Implications for D&I Research:** This study asks whether a group of experienced implementation scientists are able to match implementation strategies to the barriers and facilitators that practices identify in order to tailor implementation of an evidence-based CVD risk calculator in primary care practice. Results will both assess the utility of the ERIC-generated implementation strategies and develops a model for tailoring their application in implementation research development.

**Primary Funding Source:** Agency for Healthcare Research and Quality

### S51 Implementation of an opioid withdrawal clinical pathway on an inpatient medical service

#### Kimberly Williams, Beverly Wilson, Jo Melson, Erin Booker, Sherry Hausman, Terry Horton

##### Christiana Care Health System, Newark, DE, USA

###### **Correspondence:** Kimberly Williams (kimwilliams@christianacare.org)

**Background:** Opioid-related inpatient hospital stays are increasing at alarming rates. Unidentified and/or poorly treated opioid withdrawal (OW) may be associated with inpatients leaving against medical advice (AMA) and increased health care utilization. To better understand the prevalence of this issue while improving clinical outcomes of this vulnerable population, we developed and implemented an OW clinical pathway to screen and treat OW in medical service inpatients.

**Methods:** The OW pathway included a two-item universal screening instrument to identify patients at risk for or currently experiencing OW; use of the validated Clinical Opiate Withdrawal Scale (COWS) to monitor OW symptoms and severity; a 72-hour Suboxone-based treatment protocol. Implementation of the automated pathway included programming changes to our inpatient electronic health record system and computer physician order entry system. Providers received education about the pathway and OW with an addiction medicine specialist available for consultation. All patients could access referral to community-based treatment. Outcomes measured included rates for OW screening, identification of current OW, leaving AMA, Suboxone prescriptions, and seven and 30-day readmissions.

**Findings:** After a five month pilot phase, the pathway was scaled-up within the two-hospital health system. Between December 2016 and May 2017, 72% (18,628/25,874) of admitted patients received the OW pathway screening. Of those, 2% (530/25,874) patients were identified at risk for OW and 0.4% (110/25,874) patients were identified being in active OW (COWS score ≥ 8). Of all health system patients discharged with an ICD10 OW diagnosis, 12% (9/117) left the hospital AMA; 41% (49/120) received Suboxone; 2% (2/120) and 6% (7/120) were readmitted within seven and 30 days, respectively.

**Implications for D&I Research:** Our study demonstrates a process for successfully implementing a clinical pathway to screen and treat medical service inpatients for OW. Critical lessons learned include the need to increase the dosing of the 72-hour Suboxone taper; the inclusion of a process fidelity measure where OW is systematically verified via the COWS score prior to Suboxone dosing to prevent precipitated withdrawal; and the importance of developing a robust and automated education and training process for new staff onboarding. Further evaluation is underway to validate the two-item universal OW screening instrument.

### S52 Local adaptations of a motivational interviewing intervention in community pharmacies do not negate benefits on medication adherence rates

#### Benjamin Teeter^1^, Jeremy Thomas^1^, Geoffrey Curran^1^, Appathurai Balamurugan^2^

##### ^1^ University of Arkansas for Medical Sciences, Little Rock, AR, USA; ^2^ Center for Health Advancement, Arkansas Department of Health, Little Rock, AR, USA

###### **Correspondence:** Benjamin Teeter (BTeeter@UAMS.edu)

**Background:** Many community pharmacies are implementing evidence-based cognitive services. Exploring the effect, if any, of modifications made to these services during implementation is important in determining success or failure. Previous research by Wiltsey Stirman and colleagues (2013) identified a framework with types and levels of intervention modification which could impact implementation and effectiveness outcomes. This study applied the framework to the implementation of a medication adherence intervention in the community pharmacy setting to determine the extent/type of modifications and their impact on adherence outcomes.

**Methods:** Across 3-sites, pharmacists were trained to provide a brief Motivational Interviewing (MI) intervention to at least 50 patients who were non-adherent to antihypertensive medications. Training included a three-hour online course in MI and in-pharmacy training on patient identification and documentation. Semi-structured interviews were conducted to determine modifications to the process of identifying eligible patients, MI interventions, and documenting the intervention. Directed content analysis was guided by the Wiltsey Stirman and colleagues’ framework. Pre-intervention and six month post-intervention adherence rates for the 50 patients who received the intervention at each pharmacy were collected. Paired samples t-tests were used to assess the impact of the intervention on adherence rates.

**Findings:** Modifications were made to the *context* of the intervention (e.g., telephone instead of in-pharmacy). Additionally, *content* modifications included ‘loosening the structure’ (e.g., reordering intervention steps), ‘drifting or departing’ (e.g., too busy to attempt), ‘adding elements’ (e.g., reminder cards), and ‘repeating elements’ (e.g., patient identification). There were statistically significant improvements in adherence from pre-intervention to six months post-intervention (74.1% to 84.5%; p<0.05) at each pharmacy regardless of the adaptation applied.

**Implications for D&I Research:** Modifications made during local implementation were classified using the Wiltsey Stirman and colleagues’ framework. Despite modifications, adherence rates improved and were consistent with expectations based on prior studies of similar interventions. These findings support previous implementation research on adaptability and suggest that the ability to tailor, adapt, or refine the intervention to meet the needs of the provider/setting may allow for success of the intervention. Future research on the impact of specific modifications will help determine the modifications that are detrimental and beneficial to patient outcomes and sustainability of services.

**Primary Funding Source:** Arkansas Department of Health

### S53 A pilot study testing the effectiveness, feasibility, and fidelity of implementing a shared decision making visit for lung cancer screening in the screening setting

#### Lisa M. Lowenstein, Myrna Cobos Barco Godoy, Zineb Zirari, Viola B. Leal, Ashley J. Housten, Jeremy J. Erasmus, Robert J. Volk, Members of the Lung Cancer Screening Collaborative Group

##### University of Texas MD Anderson Cancer Center, Houston, TX, USA

###### **Correspondence:** Lisa M. Lowenstein (LMLowenstein@mdanderson.org)

**Background:** The effectiveness and feasibility of implementing the required shared decision making (SDM) visit by the Centers for Medicare & Medicaid Services (CMS) for lung cancer screening (LCS) has not been established.

**Methods:** The Replicating Effective Programs framework guided our implementation project in three clinic settings within one organization. We held weekly meetings with all stakeholders during the pre-condition, pre-implementation, and implementation phases. Using a user-centered approach, we adapted an existing SDM intervention for LCS to be web-enabled and interactive. The SDM intervention contained a decision aid section for the patient to review prior to the clinical encounter and a decision coaching section that guided the clinician through the SDM visit ensuring fidelity to the CMS requirements. Clinicians participated in an hour-long training. Patients from the pre-implementation and implementation phase completed an anonymous survey assessing their knowledge (5 items) and perceptions of the SDM process (3 items from CollaboRATE). We conducted time-motion studies (TMS) during the pre-implementation and implementation phases to assess the impact on clinical workflow from patient check-in to check-out. To assess fidelity to the CMS requirements (e.g., benefits and harms of screening), we audio-recorded the clinical encounters. STATA was used for statistical analysis.

**Findings:** A total of 51 pre-implementation phase patients and 30 implementation phase patients completed the survey. The pre-implementation patients were less informed about the benefits and harms of LCS compared to the implementation patients (pre-implementation average % correct=51%, implementation average % correct=75%, p-value<0.001). The median pre-implementation CollaboRATE scores were lower than the median implementation CollaboRATE scores (pre-implementation=12.0±6.0, implementation=14.0±3.0, p-value=0.04). We collected 16 TMS during the pre-implementation phase and 30 TMS during the implementation phase. Implementing the SDM intervention did not statistically increase the average patient time from check-in to check-out in minutes (pre-implementation=84.0±18.7; implementation=86.1±19.0; p-value=0.72). The fidelity to the CMS requirements was high with a mean of 6.4 of 7 total possible points.

**Implications for D&I Research:** These data suggest that implementing the SDM visit using decision coaching in the screening setting resulted in patients being more informed and having a better SDM process. Additionally, implementing the SDM intervention did not increase patient time to complete LCS.

**Primary Funding Source:** The University of Texas MD Anderson Cancer Center Duncan Family Institute for Cancer Prevention and Risk Assessment

### S54 When an intervention is not designed for dissemination: developing a strategy to improve system-innovation fit

#### Shellie Ellis^1^, Tomas Griebling^1^, Bryan Weiner^2^, Christine Mackay^3,4^, Jessie Gills^4^, Andrew Zganjar^4^, Mugur Geana^5^, Brantley Thrasher^1^

##### ^1^ University of Kansas School of Medicine, Kansas City, KS, USA; ^2^ University of Washington, Seattle, WA, USA; ^3^ University of Kansas Cancer Center, Fairway, KS, USA; ^4^ University of Kansas Medical Center, Kansas City, KS, USA; ^5^ School of Journalism, University of Kansas, Lawrence, KS, USA

###### **Correspondence:** Shellie Ellis (sellis4@kumc.edu)

**Background:** Innovation adoption is a precursor to implementation. Yet, few strategies exist to improve the innovation-system fit of evidence-based interventions. Reframing evidence-based practices using marketing approaches can enhance their appeal, but requires understanding the goals and motivations of adopters. We sought to compile an inventory of innovation attributes that might motivate physicians to adopt a treatment innovation.

**Methods:** We pooled data from two qualitative studies to identify factors that motivate physicians to offer a treatment option. Both studies used a semi-structured interview guide based on the Theoretical Domains Framework. Study 1 included a national sample of academic and community urologists and focused on adopting a treatment option for prostate cancer. Study 2 included regional community urologists and focused on adopting a treatment option across all urological cancers. We reviewed transcripts to identify factors urologists identified as motivating either their own or other urologists’ behavior in treatment adoption.

**Findings:** We identified 18 considerations urologists in the two studies mentioned as being motivating factors in selecting a treatment. We framed them as attributes which might appeal to urologists in considering adoption of an innovation (Table 1).

**Implications for D&I Research:** Innovation adopters will not accept the motivations and goals of innovation designers. Innovations must be compatible with potential adopters’ existing drives. Our inventory identifies 18 potential drivers for innovation adoption. Additional research should assess these attributes for construct validity across target populations to identify clusters of attributes more important in particular contexts.

**Primary Funding Source:** ACS

**Table 1:** **(Abstract S54).** Attributes Important for Practicing Physicians’ Adoption of Innovations

Matching the right patient to the right treatment
Reducing vulnerability to legal action
Lessening the risk of patients’ decisional regret
Helping you adhere to practice guidelines
Reducing patient questions
Reducing repeat visits to discuss treatment options
Increasing the practice’s reputation as offering cutting edge treatment options
Decreasing your need to refer patients to other healthcare providers
Negatively impacting your practice’s bottom line
Positively impacting your practice’s bottom line
Differentiating your practice from other specialty practices in the area
Addressing public concerns about overtreatment
Making care more patient centered
Improving patient satisfaction
Reducing demands for more aggressive care than necessary
Reducing costs of care
Improving patient outcomes
Increasing patient adherence

### S55 The smoking treatment for Ontario patients (STOP) study: 10 years of implementation and dissemination in a variety of clinical settings

#### Laurie Zawertailo^1,2^, Rosa Dragonetti^1^, Sarwar Hussain^1^, Dolly Baliunas^1^, Peter Selby^1,2,3^

##### ^1^ Centre for Addiction and Mental Health, Toronto, ON, Canada; ^2^ University of Toronto, Toronto, ON, Canada; ^3^ Dalla Lana School of Public Health, University of Toronto, Toronto, ON, Canada

###### **Correspondence:** Laurie Zawertailo (laurie.zawertailo@camh.ca)

**Background:** The STOP program is a province-wide government funded smoking cessation treatment program designed to decrease smoking prevalence in Ontario Canada by increasing the availability and use of effective smoking cessation medications in combination with counselling support for smokers who are interested in quitting. The program evaluates the effectiveness of various methods of distributing free nicotine replacement treatment in order to inform the development of future cessation programs.

**Methods:** Since 2005, the program has investigated 12 different methods for distribution of free nicotine replacement therapy in combination with different types of counselling support to eligible smokers: these fall into two broad delivery methods – direct to smokers or through a healthcare provider intermediary (i.e. primary care clinic, addictions agency, community pharmacy, public health unit, specialized smoking cessation clinic). The program has evolved over the years and now includes research into the effectiveness of concurrent brief interventions for conditions that are highly prevalent in smokers; depression and risky alcohol use. The program has also engaged healthcare providers in various sectors including public health, primary care and addictions treatment.

**Findings:** Over 190,000 smokers have participated in the program since inception. Over the past 6 years we have focused on implementing the program in primary care settings and currently we have over 300 distinct organizations providing STOP. These organizations enroll over 7,000 new participants every quarter with quit rates at 6-months of approximately 35%, compared with unassisted quit rates which fall around 1 to 3% at the same time period.

**Implications for D&I Research:** Implementing and maintaining an evidence-based smoking cessation treatment program in hundreds of settings across a large geographic area and in diverse settings can be achieved using a hub-and-spoke approach. Our STOP team served as the hub that provided online training, computer decision support and medication management, allowing us to manage all aspects of the program remotely with no site visits. The spokes included the various sites across the province including remote communities with little access to treatment. This model can be used to implement other health behavior management programs. We are currently expanding this model to support alcohol screening as well as identification of depression.

**Primary Funding Source:** Ontario Ministry of Health and Long-term Care

### S56 The teach project: facilitating a decade of practice change through provision of tobacco cessation training

#### Myra Fahim^1,2^, Rosa Dragonetti^2^, Megan Barker^2,3^, Mathangee Lingam^2^, Sheleza Ahad^2^, Arezoo Ebnahmady^2^, Peter Selby^2,3,4^

##### ^1^ School of Social Work, University of Windsor, Windsor, ON, Canada; ^2^ Centre for Addiction and Mental Health, Toronto, ON, Canada; ^3^ Dalla Lana School of Public Health, University of Toronto, Toronto, ON, Canada; ^4^ University of Toronto, Toronto, ON, Canada

###### **Correspondence:** Rosa Dragonetti (Rosa.Dragonetti@camh.ca)

**Background:** Despite an existing wealth of knowledge in evidence-based tobacco dependence treatment (TDT), healthcare providers (HCP) cite limited self-confidence, efficacy and training as a barrier to providing effective TDT. To address these barriers,, the Training Enhancement in Applied Cessation Counseling and Health (TEACH) Project, a knowledge translation (KT) initiative, was initiated in 2006 to provide interdisciplinary HCPs with evidence-based training in TDT.

**Methods:** The Knowledge to Action Framework, was utilized to operationalize the development and application of TEACH. Over the past 10 years, TEACH has implemented a 43.5 hour certificate program utilizing best practice in adult education and the latest advancements in cessation research. Robust training activities and evaluation processes have been developed to facilitate and sustain the KT process. Collaborative and experiential learning is ensured through principles in effective instructional design and is re-assessed iteratively through pre/post assessments, formative and summative evaluations, and follow-ups post-training. Due to an increasing demand for accessible, effective and low cost training, TEACH successfully transitioned its courses online. Following training, practitioners are provided access to a wide range of Community of Practice (CoP) activities including, webinars, listservs, Practice Champions, and ongoing leadership trainings.

**Findings:** Since 2006, 5,623 HCPs have been trained representing 37 disciplines and 1300 organizations. CoP activities include 106 webinars offered, 52 clinical practice vignettes, 321 Practice Leaders and 801 HCPs subscribed to an active listserv. Pre/post assessment data has shown a significant increase in self-reported feasibility, importance and confidence in changing practice, with 82.96% of trained HCPs setting practice goals. At 6-month follow up, 96.15% of HCPs reported KT activities within their organizations/communities.

**Implications for D&I Research:** The use of a KT framework and best practices in adult education to guide the operationalization of the project has highlighted the effectiveness of TEACH in sustaining practice change.

**Primary Funding Source:** Ontario Ministry of Health and Long-term Care

### S57 Balancing fidelity with fit: collaboratively adapting cessation treatment models to improve health equity for indigenous communities

#### Megan Barker^1^, Lisa Beedie^3^, Ryan Ting-A-Kee^1^, Rosa Dragonetti^1^, Peter Selby^1,2,4^

##### ^1^ Centre for Addiction and Mental Health, Toronto, ON, Canada; ^2^ Dalla Lana School of Public Health, University of Toronto, Toronto, ON, Canada; ^3^ Cancer Care Ontario, Toronto, ON, Canada; ^4^ University of Toronto, Toronto, ON, Canada

###### **Correspondence:** Megan Barker (Megan.Barker@camh.ca)

**Background:** The Centre for Addiction and Mental Health’s (CAMH) STOP Program is an Ontario-wide initiative that delivers free commercial tobacco cessation to eligible Ontarians who wish to quit smoking. Since 2005, STOP has treated over 190,000 smokers. Despite this reach, Indigenous Peoples, who have some of the highest rates of commercial tobacco use worldwide, had been previously unable or unwilling to access the STOP model in its traditional form.

**Methods:** Through a community-based participatory approach, CAMH collaborated with Cancer Care Ontario’s Aboriginal Tobacco Program (ATP) to adapt the STOP Program with Indigenous communities, primarily with Aboriginal Health Access Centres (AHACs) which serve as community-led, primary health care organizations in Ontario. To ensure STOP with AHACs meets the needs of communities served by each AHAC, STOP staff work closely with AHACs to ensure fidelity of the model while supporting flexibility and fit (e.g., offering flexible implementation aligned with existing programming and increasing access to medication for those in remote communities). Tailored resources and training for AHACs were co-created with Indigenous community members (CAMH Engagement Circle, n=56) to foster implementation of evidence- and wise-based approaches to cessation. A STOP with AHACs Community of Practice has been formed to (1) co-create a collaborative research model aligned with Indigenous Research Principles (2) modify data collection forms to ensure a strength-based approach and (3) provide collaborative opportunities across Indigenous communities throughout Ontario.

**Findings:** To date, 7 Indigenous health organizations across 14 sites, including 5 AHACs (50% of AHACs in Ontario) are currently implementing the program and a total of 499 clients have received medication and counselling. 10 additional sites (including the remaining AHACs) have indicated interest in offering the program. An online training program and two toolkits have been co-created to support healthcare providers in delivering cessation interventions with Indigenous Peoples.

**Implications for D&I Research:** Balancing program fidelity (i.e., evidence-based cessation) with flexibility and fit (i.e., meeting and responding to community and organizational needs) is crucial to successful, sustained implementation of treatment models. Adapting this program plays an important role in improving health equity among Indigenous Peoples and advances community services and programs.

**Primary Funding Source:** Ontario Ministry of Health and Long-term Care

### S58 Economic evaluation of an effective quality improvement intervention and its implementation

#### Amy Cohen^1,2^, Eric Slade^3,4^, Alison Hamilton^1,2^, Alexander Young^1,2^

##### ^1^ Department of Veterans Affairs, Los Angeles, CA, USA; ^2^ University of California Los Angeles, Los Angeles, CA, USA; ^3^ Department of Veteran's Affairs, Baltimore, MD, USA; ^4^ University of Maryland, Baltimore, MD, USA

###### **Correspondence:** Amy Cohen (amy.cohen@va.gov)

**Background:** Economic evaluations are rarely performed in mental health quality improvement efforts. EQUIP was a clinic-level controlled trial in the Veteran’s Administration (8 sites across 4 states, N=801 patients with schizophrenia) of a 12-month intervention designed to increase utilization and impact of high priority evidence-based practices for this vulnerable population. The intervention increased utilization of both weight and employment services (by 2.3 times) resulting in less weight gain (-12 lbs/individual) but not increased employment, a more distal outcome. The economic evaluation estimated the average VA cost-consequences and cost-effectiveness of EQUIP, compared to usual care, in achieving improvements.

**Methods:** We supplemented EQUIP study records on dedicated staff time and other resources utilized for program implementation and operation with data on healthcare utilization and costs. In addition to direct costs, we examined indirect costs associated with use of other VA outpatient services. Indirect costs were examined by comparing use of outpatient mental health, primary care, and rehabilitation services between EQUIP and usual care sites, controlling for spending during the 6-month period prior to EQUIP.

**Findings:** The one-time cost of the setting up EQUIP was $14,385 per site. These costs include the effort and salary of the staff who prepared the intervention, marketed the project, and engaged existing services. The average annual cost of delivering EQUIP was $1,075 per patient. In the EQUIP group, VA outpatient health care costs increased by $1,195 per person compared to the 12-month period preceding baseline. By contrast, in the usual care group, outpatient costs increased by $1,810 per person, or by $615 more per person than in EQUIP. Most of these savings were the result of lower utilization of intensive outpatient mental health services for those receiving EQUIP. Thus, our estimates suggest that 57% of the $1,075 per person direct cost of EQUIP was offset by lower outpatient costs for other services.

**Implications for D&I Research:** Cost-effectiveness analyses of evidence-based practices are scarce and those that have been conducted typically only include the cost of the practice and not the effort to implement and ensure utilization. This evaluation which includes those aspects is a model for other such evaluations.

**Primary Funding Source:** Department of Veterans Affairs

### S59 Implementing an early labor management program: shifting the culture of obstetrical care

#### Rachel Breman (rbreman1@umaryland.edu)

##### School of Nursing, University of Maryland, Baltimore, MD, USA

**Background**: Research shows a higher risk of cesarean when women are admitted to the hospital in early labor. The Knowledge-to-Action Framework guided an implementation study assessing the adoption of an early labor support program. An interdisciplinary team designed a new triage protocol and an early labor lounge with evidence-based activities. The program’s objective was to shift the culture of admitting low risk women too early in their labor process with the end of goal of decreasing the cesarean delivery rate.

**Methods**: In this multi-method study, a purposive sample of 25 hospital staff were interviewed. Interviews were recorded, transcribed and coded by a two-person team. A convenience sample of 67 first time mothers completed an electronic survey after they delivered and prior to discharge. The Birth Satisfaction Tool captured satisfaction. Additional data were collected on prenatal provider and mode of delivery.

**Findings**: Staff interviews identified emerging themes for barriers and facilitators. Barriers included a lack of protocol knowledge and variation in buy-in to the program. Facilitators were empowering women during labor and the use of tools from the early labor lounge. Survey data from the women showed barriers including 33% indicating their prenatal provider did not discuss planning for labor to start spontaneously. Facilitators from the surveys found that 43% used the lounge; 93% of these indicated the lounge helped their partner coach them through early labor and 89% would recommend its use. 21% of the surveyed women delivered by cesarean, however, during this same time period the rate was 30% for this population. Initial analysis shows those surveyed had a high level of birth satisfaction.

**Implications for D&I Research:** The early labor lounge provides laboring women a high level of support and satisfaction. Understanding context of active labor admission needs further research. While an interdisciplinary team designed the program, some staff had difficulty not admitting women to a bed. Furthermore the mother’s birth plans need to be fully understood. Safety is another topic requiring further investigation because some staff raised concerns about patient safety with lounge use, yet overuse of cesarean was also recognized as a safety issue.

### S60 Implementing a systematic approach to shred decision-making regarding end-of-life care with renal dialysis patients

#### Sarah Goff^1^, Nwamaka Eneanya^2^, Talaya Martinez^3^, Jamie Klingensmith^1^, Casey Garvey^4^, Lewis Cohen^1^, Mark Unruh^3^

##### ^1^ University of Massachusetts Medical School - Baystate, Springfield, MA, USA; ^2^ Massachusetts General Hospital, Boston, MA, USA; ^3^ University of New Mexico, Albuquerque, NM, USA; ^4^ Boston University, Boston, MA, USA

###### **Correspondence:** Sarah Goff (sarah.goffmd@baystatehealth.org)

**Background:** Renal dialysis patients have better experiences with end-of-life (EOL) when health professionals elicit their care preferences. Although patients desire these discussions, they rarely occur. Potential barriers include ambiguity about prognosis, lack of health care provider preparedness, time, and perceived potential for harm to the patient.

**Methods:** We conducted a Type 1 Hybrid Effectiveness-Implementation study of an intervention to improve concordance of EOL preferences with actual care for dialysis patients in Massachusetts and New Mexico. Key implementation activities included: 1) in-depth interviews with dialysis patients about experiences with EOL discussions; 2) training social workers (SWs) and nephrologists to use shared decision-making principles in EOL discussions; 3) identifying barriers/facilitators to implementation; 3) checking intervention fidelity; and 4) obtaining feedback about implementation and intervention.

**Findings:** Most interviewees desired EOL discussions with their physician or someone they trusted in the unity, yet some felt there was no one they trusted sufficiently. SWs attended an 8-hour training session plus additional booster training sessions. Although feedback on the training was positive, some SWs resisted participation in the intervention. Convening nephrologists for training was challenging, but a video created by the research team fostered training. Additional barriers included IRB delays due to concerns about conducting the study in dialysis units, coordinating schedules for intervention sessions, nephrologists and SWs declining to have intervention sessions recorded, and difficulty recruiting patients. Twenty-three of 125 intervention sessions were checked for fidelity using 8 criteria. A median of 5/8 (range 0-8) criteria were met. The criteria least frequently met included asking patients to summarize in their own words (11/23;47.8%) and discussing prognosis (11/23;47.8%). Results of the effectiveness portion of the study are pending.

**Implications for D&I Research:** This study identified potential barriers to implementing an intervention to improve concordance of EOL care with dialysis patient preferences. Potential solutions to problems identified include systematically incorporating EOL discussions into standing annual care reviews for all patients regardless of prognosis and engaging key stakeholders, such as SWs, in the early stages of implementation planning. Establishing nephrologists and SWs as part of the intervention and implementation team early in the design process may result in less resistance to fidelity checks.

**Primary Funding Source:** Patient-Centered Outcomes Research Institute

### S61 Pilot cluster randomized trial of hospitals: toolkit improves screening for risky drug and alcohol use

#### Robin Newhouse^1^, Meg Johantgen^2^

##### ^1^School of Nursing, Indiana University, Indianapolis, IN, USA; ^2^School of Nursing, University of Maryland, Baltimore, MD, USA

###### **Correspondence:** Robin Newhouse (newhouse@iu.edu)

**Background:** One in four hospital admissions are related to substance use. An efficacious intervention, Screening Brief Intervention and Referral to Treatment (SBIRT), is now included as a standard measure of acute care quality by The Joint Commission to promote better identification and treatment of patients at risk for unhealthy alcohol use. The purpose of this study was to test two approaches [evidence-based (EBP) approach vs standard education] to implementing nurse initiated SBIRT in acute care hospitals.

**Methods:** A mixed methods, cluster randomized trial of hospitals tested the intervention (EBP approach) effects on SBIRT adoption in inpatient nursing units (measured by medical record review at baseline and six months post implementation). Seven hospitals (3 standard education and 4 EBP approach) in the eastern United States selected one medical or surgical or combined unit to participate. All hospitals received SBIRT training and an evidence-based implementation toolkit (algorithms, teaching tools, staff training materials). A random sample of medical records were reviewed for SBIRT adoption [baseline (N=416) and 6-months (N=418)] post implementation; 83 nurses from 6 sites responded to an implementation survey on randomly selected days. Post intervention interviews were conducted with site coordinators to assess implementation strategies.

**Findings:** Both groups significantly improved screening [23.6% to 58.4% for alcohol and 21.6% to 51.4% for drugs and brief interventions from baseline to six months (27.3% to 35.3%).There were no differences in detection of unhealthy use, receiving brief intervention, prescription for substance abuse drug, or referral for treatment. Nurses reported a high level of screening post intervention (79% screened their last patient) with acceptable time commitment (N=75, M=3.45 minutes, sd=3.2 minutes). Site coordinators reported that the toolkit was effective to enhance implementation.

**Implications for D&I Research:** Implementation of the SBIRT toolkit improves screening for unhealthy drug and is implementable by nurses in acute care hospitals.

**Primary Funding Source:** The Robert Wood Johnson Foundation

## Clinical Care Settings: System-level Interventions

### S62 Scale-up and spread of collaborative care for general mental health teams

#### Christopher Miller^1,2^, Bo Kim^1,2^, Mark Bauer^1,2^, Tracey Smith^3,4^, Kendra Weaver^5^

##### ^1^ Center for Healthcare Organization and Implementation Research, VA Boston Healthcare System, Boston, MA, USA; ^2^ Harvard Medical School, Boston, MA, USA; ^3^ South Central MIRECC, Center for Innovation in Quality, Effectiveness, and Safety, North Little Rock, AR, USA; ^4^ Baylor College of Medicine, Houston, TX, USA; ^5^ Office of Mental Health and Suicide Prevention, Department of Veterans Affairs, Mountain Home, TN, USA

###### **Correspondence:** Christopher Miller (christopher.miller8@va.gov)

**Background:** Spreading evidence-based practices is an ongoing challenge. Many successful health innovations are never widely disseminated, or are disseminated only after significant delays. Implementation frameworks suggest that several factors—including characteristics of the innovation and the staff whose practices would need to accommodate it—need to be addressed to achieve successful spread. Challenges in establishing spread are especially great for large, complex innovations such as the Collaborative Care Model (CCM), a flexible approach to organizing healthcare services within a clinic or team. While the evidence base for the CCM in healthcare is robust, few studies have explored the implementation, scale-up, and spread of CCM-based care in real-world settings.

**Methods:** In this presentation, we describe the scale-up and spread of collaborative care, following the launch of a nine-site stepped-wedge randomized controlled trial. These efforts relied on blended facilitation (featuring internal and external facilitators), working with clinical teams for 6-12 months. We achieved this scale-up in partnership with VA’s Office of Mental Health and Suicide Prevention (OMHSP) and National Transformational Coach Program. Specifically, we developed a two-day curriculum (supported by ongoing consultation) to train Transformational Coach (“t-Coach”) Captains, who are helping additional mental health teams adopt the CCM.

**Findings:** We trained 13 t-Coach Captains during our on-site training in September, 2016. A total of 34 VA medical centers provided signed letters of agreement in early 2017 requesting facilitation support. Three of these facilities later withdrew from the program, and two additional sites expressed interest (resulting N=33 sites). To date, facilitators are in the process of providing ongoing facilitation at 25 of those medical centers. The mental health care of more than 20,000 Veterans has been impacted by these facilitation efforts, and additional spread is planned for subsequent years.

**Implications for D&I Research:** This presentation describes the spread of a complex care delivery model—the CCM—to mental health teams nationally. This presentation will discuss themes critical to the success of these implementation efforts, including: collaborations with VA operational entities, the alignment of our implementation efforts with the priorities of the teams adopting the CCM, and the provision of ongoing consultation for both facilitators and frontline staff.

**Primary Funding Source:** Department of Veterans Affairs

### S63 Opportunities for expanding access to overdose education and naloxone: using an implementation science framework to identify strategies in community health centers

#### Michele Clark^1^, William DeJong^2^, Emily Feinberg^2^, Maura Fagan^3^, Alexander Walley^3^, Mari-Lynn Drainoni^2,3,4^

##### ^1^ JSI Research & Training Institute, Inc. (JSI), Boston, MA, USA; ^2^ Boston University School of Public Health, Boston, MA, USA; ^3^ Boston University School of Medicine, Boston, MA, USA; ^4^ Edith Nourse Rogers Memorial Veterans Hospital, VA Center for Healthcare Organization and Implementation Research (CHOIR), Bedford, MA, USA

###### **Correspondence:** Michele Clark (michele_clark@jsi.com)

**Background:** Naloxone, an opioid antagonist, is a vital tool for preventing opioid overdose deaths. Opioid overdose education combined with naloxone rescue kit distribution (OEND) has been shown to be a safe, feasible, and effective intervention at the community-level. Community health centers (CHCs) are a promising location for expanding OEND, yet little is known about the implementation strategies and factors in these settings.

**Methods:** We conducted a mixed-methods study to document OEND strategies employed by early adopter CHCs in eight Massachusetts communities with high opioid overdose fatality rates. Relevant program documents and transcripts of semi-structured interviews and focus groups with 29 clinic staff were deductively analyzed using pre-determined constructs from the Consolidated Framework for Implementation Research (CFIR). Participant- and clinic-level survey data were used to explore associations between prominent constructs and clinic and participant OEND implementation levels.

**Findings:** Nine CFIR constructs (1-9) across the Inner Setting, Intervention Characteristics, Outer Setting, and Process domains were found to determine the nature and course of OEND implementation. 1) *Tension for change* and presence of a 2) *champion* served as catalysts. 3) *Adaptability* figured prominently as CHCs utilized a mix of approaches, including internal clinic distribution, issuing prescriptions, utilizing pharmacy standing orders, and referring to community programs. Also influencing the approach were 4) naloxone *cost* reimbursement mechanism, 5) attention to *patient needs and resources*, and 6) *networks and communication* within the CHC and affiliated pharmacy. Several strategies supported ongoing implementation, including 7) a harm reduction *culture*, 8) *engaging clinic staff* with training and technical assistance based on role and readiness stage, and 9) *reflecting and evaluating* on OEND performance data and staff experience.

**Implications for D&I Research:** This study demonstrated that OEND can be integrated into CHC primary care services and modified based on the context. The CFIR-guided analysis revealed that successful implementation requires a systems-level response, grounded in a team-based care model, pharmacy partnerships, visible indicators of destigmatizing substance use disorders, and efforts to minimize patient barriers. The CFIR constructs identified in this study can enhance the effectiveness of future clinic-based OEND initiatives. Future research can assess the relative impact of identified constructs on patient naloxone access outcomes in CHC settings.

### S64 Organizational culture and climate as moderators of enhanced outreach for persons with serious mental illness: results from a cluster-randomized trial of adaptive implementation interventions

#### Shawna Smith^1^, Daniel Almirall^2^, Katherine Prenovost^1,3^, David Goodrich^3^, Kristen Abraham^4^, Celeste Liebrecht^1,3^, Amy Kilbourne^1,5^

##### ^1^ University of Michigan, Ann Arbor, MI, USA; ^2^ University of Michigan Institute for Social Research, Ann Arbor, MI, USA; ^3^ Center for Clinical Management Research, VA Ann Arbor, Ann Arbor, MI, USA; ^4^ SMITREC, VA Ann Arbor, Ann Arbor, MI, USA; ^5^ QUERI, Department of Veterans Affairs, Ann Arbor, MI, USA

###### **Correspondence:** Shawna Smith (shawnana@med.umich.edu)

**Background:** Organizational characteristics, including culture and climate, can influence uptake of evidence-based practices (EBP). However, studies have yet to examine whether culture/climate moderate comparative effectiveness of different implementation strategies, including strategies that are more resource-intensive. We examined how entrepreneurial culture and task climate moderated the effects of two different adaptive implementation interventions (immediate vs. delayed enhancement) for improving uptake of an outreach program (Re-Engage) at Veterans Health Administration facilities not initially responsive to a standard implementation strategy.

**Methods:** Patients (n=3,075) at N=89 sites not initially responsive to a REP-based implementation of Re-Engage (updated documentation for <80% of listed patients) were randomized to two adaptive implementation interventions: immediate E-REP (N=40), which added provider coaching for 6 months followed by REP for 6 months; or delayed E-REP (N=49), which continued REP for 6 months followed by E-REP only for non-responsive sites. Site uptake data was merged with site-aggregated culture and climate measures from the VA All Employee Survey (AES). We hypothesized that sites with lower entrepreneurial culture or task climate scores would benefit more from immediate E-REP than sites with higher scores. Three-level mixed-effects logit models were used to test whether organizational culture/climate moderated the effects of immediate vs. delayed E-REP on updated documentation, attempted contacts, and completed contacts 12 months post-randomization.

**Findings:** Contrary to our hypothesis, sites with higher entrepreneurial culture and task climate measures benefitted more from immediate vs. delayed E-REP. After 12 months, patients at sites with high entrepreneurial culture had a 41 percentage point higher predicted probability of having updated documentation under immediate E-REP than delayed; at low entrepreneurial culture sites this difference was only 4 percentage points. Similarly, high task climate sites saw 34 percentage point higher predicted probability under immediate E-REP, compared to 6 percentage point difference with low task climate. Similar effects were seen for attempted but not completed contact.

**Implications for D&I Research:** Sites with more entrepreneurial culture and better task climate improved uptake more under immediate provision of an enhanced implementation strategy than those with lower scores. Sites with less entrepreneurial cultures or lower task climate may need additional implementation intervention augmented to E-REP to see full benefits.

**Primary Funding Source:** Department of Veterans Affairs

### S65 Preventing organizational relapse after implementation: a new model for sustainability

#### Diane Powers, Deborah Bowen

##### University of Washington, Seattle, WA, USA

###### **Correspondence:** Diane Powers (powersd@uw.edu)

**Background:** It can take years before an innovation becomes the new status quo. During that time organizations are vulnerable to partial or complete relapse and face many forces that promote relapse. Adapting the concept of relapse prevention from the field of addictions we created a process to support clinics implementing Collaborative Care (CC) for depression. Organizational relapse prevention results in a tailored plan for each clinic that identifies early warning signs of relapse and pre-planned course corrections designed to prevent small deviations in new practices from becoming a complete reversion to old practices. Clinics need this to sustain innovation until the status quo is reset.

**Methods:** We tested an organizational relapse prevention model in eight primary care clinics that enrolled in two cohorts one year apart. CC requires medical and behavioral health clinicians to practice in new ways that differ from their original training. Clinics received 2.5 years of implementation support, including organizational relapse prevention. Each relapse prevention plan included specific processes for measuring and monitoring early warning signs and clinic-specific interventions to employ for each. Clinics used a registry that facilitates CC by monitoring patient-level clinical outcomes as well as clinic-level processes of care. Systematic case review by a psychiatric expert is an evidence-based process of care that predicts patient-level outcomes. Registry data was analyzed to measure depression outcomes and use of evidence-based practices during the implementation support phase and for one year thereafter among the four clinics in the first cohort.

**Findings:** Three of four clinics maintained or improved the proportion of patients receiving systematic case review while one clinic relapsed on this indicator. The proportion of patients experiencing response or remission of depression symptoms was similarly analyzed during and after the active implementation support phase. Both measures were lower one year later but remained higher than the established benchmark for FQHCs. Qualitative data revealed clinics appreciate a method to manage organizational relapse and use it to sustain implementation.

**Implications for D&I Research:** System-level interventions to prevent relapse are an important component of any implementation effort. This model of organizational relapse prevention shows promise and could be adapted to support implementation of other innovations.

**Primary Funding Source:** Corporation for National and Community Service (Social Innovation Fund), The John A. Hartford Foundation, Margaret A. Cargill Foundation, The Helmsley Charitable Trust, Rasmussen Foundation, Lewis County Board of Commissioners

### S66 Attributes of facilitators as individual knowledge brokers

#### Patti Grota (grota@uthscsa.edu)

##### Nursing, UT HEALTH OF SAN ANTONIO, San Antonio, TX, USA

**Background:** Knowledge brokering is a complex activity that provides a link between research producers and end users. The greatest barrier to successful knowledge brokering is lack of evidence about how brokering works and the factors that influence its effectiveness. Although it can be carried out by groups, organizations or an individual, there has been an increasing interest in the individual knowledge broker (KB) or facilitator. Because little is understood about the characteristics of a successful KB, there is no particular education or training. Thus, there is a need for further research to define the characteristics of an effective KB in a variety of settings and among different health care professionals.

**Methods:** An integrative literature review was conducted between January 2000 and July 2016 to describe the characteristics of facilitators as KBs. Multiple data bases including PubMed, Cochrane, CINAHL, and Scopus were used to search key words including KB and facilitator. In addition, a hand search of *Implementation Science* was completed. Additional publications were identified through snowballing. A total of 55 full text publications were appraised by two independent reviewers using The Critical Appraisal Skills Program Systematic Review Checklist with 18 selected for the final cohort. Disagreements between the reviewers were resolved with consultation from a third reviewer.

**Findings:** Attributes of KB were identified and extracted into six domains of the facilitation competency framework building on Goleman's mixed model of emotional intelligence. Examples of attributes identified in the literature under each domain are: self-confident and intuitive under self-awareness; flexible and committed under self-management; empathetic and respectful under social awareness, engaging and empowering under relationship management; organized and resourceful under skills; and clinical and process expert under knowledge and understanding. The extent to which these attributes contribute to facilitation remains unknown.

**Implications for D&I Research:** Effective teams are led by effective facilitators. This study begins to identify and categorize attributes that have been described as important characteristics of individual KBs in the literature. Yet, there have been no studies to validate the contribution of these attribute(s) as predictors of effective KB. Expansion of KB theoretical frameworks is needed to strengthen dissemination and implementation science.

### S67 Stages of the innovation implementation process: piloting a Medicaid population health management intervention in community pharmacies

#### Mrs. Kea Turner^1^, Christopher M. Shea^1^, Chelsea Phillips Renfro^2^, Stefanie Ferreri^2^, Morris Weinberger^1,3^, Troy Trygstad^2,4^

##### ^1^ UNC Gillings School of Global Public Health, Chapel Hill, NC, USA; ^2^ UNC Eshelman School of Pharmacy, Chapel Hill, NC, USA; ^3^ Center for Health Services Research in Primary Care, Durham Veterans Affairs Health Care System, Durham, NC, USA; ^4^ Pharmacy Programs, Community Care of North Carolina, Raleigh, NC, USA

###### **Correspondence:** Kea Turner (keat@email.unc.edu)

**Background:** Approximately 250 North Carolina community pharmacies are participating in the Community Pharmacy Enhanced Services Network (CPESN)—a Medicaid population health management intervention. With CPESN, community pharmacies must deliver medication management services to high-risk patients (e.g., comprehensive medication reviews), document those services, coordinate care with other providers, and receive payment based on a combined value-based and fee-for-service model. CPESN is new for many community pharmacies, and therefore guidance is needed to support effective implementation. The goal of this study is to identify key decisions and actions needed at each stage of the CPESN implementation process.

**Methods:** We conducted 30 ~45-minute interviews with pharmacists who oversaw CPESN implementation. To obtain diverse perspectives on implementation, we selected respondents who represented a mix of pharmacy types (e.g., independent, federally qualified health centers, and chain pharmacies) and performance (high and low). Interviews were conducted between June – July 2017, transcribed verbatim, and analyzed for themes organized around Roger’s Stages in the Innovation Process in Organizations: agenda-setting, matching, redefining and restructuring, clarifying, and routinizing.

**Findings:** Participants identified improving medication management for high-risk patients and receiving additional reimbursement as reasons for joining CPESN (agenda-setting). To decide whether CPESN was appropriate for their pharmacy, participants described weighing factors such as innovation-values fit, staffing, and leadership support (matching). During the restructuring and redefining stage, pharmacies described developing protocols for delivering medication management services and building relationships with providers. Participants discussed scaling up CPESN (clarifying) by partnering with community organizations to reach additional patients. For sustainability, participants described hiring personnel supportive of CPESN and rewarding personnel for participation (routinizing). Barriers to implementation included resistance among providers, pharmacy personnel, and patients, and lack of communication with case managers.

**Implications for D&I Research:** When introducing PHM interventions into community pharmacies, key decisions and actions are needed at different stages of the innovation process—which involves patients, pharmacy staff, providers, and case managers. Findings suggest future research may be needed to assess the acceptability of pharmacy-based, PHM interventions among providers, pharmacy staff, and patients. Additionally, research is needed to test implementation strategies that strengthen partnerships among community pharmacies, providers, and case managers.

**Primary Funding Source:** The North Carolina Translational and Clinical Sciences Institute

### S68 Improving inter-hospital transfer for critically ill patients

#### Emily Finn^1^, John Sather^2^, Andrew Ulrich^2^, Vivek Parwani^2^, Kevin Sheth^2^, Charles Matouk^2^, Laura Pham^2^, Sarwat Chaudhry^2^, Beth Hodshon^2^, Arjun Venkatesh^2^

##### ^1^Yale Center for Healthcare Innovation, Redesign and Learning (CHIRAL), Yale University, New Haven, CT, USA; ^2^ Yale School of Medicine, New Haven, CT, USA

###### **Correspondence:** Emily Finn (e.finn@yale.edu)

**Background:** Patients with atraumatic intracerebral hemorrhage (ICH) or subarachnoid hemorrhage (SAH) diagnosed at community hospitals often require inter-hospital transfer (IHT) for neurocritical care. While necessary, IHT introduces risks including multiple handoffs and potential delays in intervention. Project goals were twofold: define the baseline IHT process for atraumatic ICH/SAH patients, and develop and implement an intervention to standardize care processes, enhance communication, reduce safety threats, and improve clinical outcomes.

**Methods:** This mixed-methods study included two phases: problem analysis and intervention. Problem analysis involved building a multi-disciplinary team representing all essential hospital services (emergency medicine, neurology, neurosurgery, and the IHT coordination center) and utilized five methodologies: chart review (n=1338), process mapping (17 participants over seven sessions), semi-structured interviews (n=32), real-time surveys (n=115), and content analysis of IHT requests (n=26). Findings were shared with stakeholders for validation. A multi-modal intervention was developed and vetted through meetings with all involved roles and services. The intervention, launched in May 2017, includes a redesigned IHT acceptance process, patient arrival notification system, provision of clinical guidelines, and electronic medical record (EMR) improvements. The intervention is continuously evaluated through an EMR-based dashboard, case-by-case audit, and content analysis of IHT requests.

**Findings:** Problem analysis provided deep insight into existing concerns, indicating three challenges: gaps in clinical knowledge and practice, insufficient communication, and lack of a structured IHT pathway. Specifically, 30% of real-time survey respondents felt they received an inadequate handoff; the tertiary-care emergency department (ED) never spoke with the IHT accepting service; and ED-based care missed timeliness benchmarks. Preliminary intervention evaluation indicates success. Case audits describe improved interdisciplinary engagement; the dashboard shows reduced median ED length of stay (from 266 minutes in April 2017 to 186 in June); and content analysis has identified challenges that have been resolved; for instance, in the first month, the correct service was called to accept IHT in 40% of cases; by the second month, this increased to 68%.

**Implications for D&I Research:** This work highlights the strengths and limitations of different quantitative and qualitative implementation science approaches, and reinforces the benefits of a multi-modal study design, continuous evaluation, and engagement of a multi-disciplinary team for improving high-risk care transitions.

**Primary Funding Source:** Agency for Healthcare Research and Quality

### S69 Applying a work-reduction model to develop a scalable toolkit for SDM implementation in cardiovascular risk reduction

#### Aaron Leppin^1^, Kasey Boehmer^1^, Ian Hargraves^1^, Megan Branda^1^, Sara Dick^1^, Glyn Elwyn^2^, Nilay Shah^1,3^, Alex Alexander^1^, Victor Montori^1^

##### ^1^ Mayo Clinic, Rochester, MN, USA; ^2^ Dartmouth Institute for Health Policy & Clinical Practice, Dartmouth College, Lebanon, NH, USA; ^3^ Optum Labs, Cambridge, MA, USA

###### **Correspondence:** Aaron Leppin (leppin.aaron@mayo.edu)

**Background:** Shared decision making (SDM) is promoted for discussions about initiating statin therapy. The Statin Choice Decision Aid (SCDA) is an effective SDM intervention that has been implemented in index health systems, but has not achieved widespread adoption and scale. Implementation toolkits are appealing options for facilitating the adoption of interventions across organizations, yet little is known about how to design toolkits to maximize their impact. We sought to design a toolkit to facilitate the passive adoption of the SCDA by orienting its components to counteract the work stakeholders had to do to adopt the tool in diverse health systems.

**Methods:** We conducted multi-level and mixed methods (survey, interview, observation, focus group) characterizations of the contexts of 3 health systems (n=86, 84, and 26 primary care clinicians) as they pertained to the impending implementation of the SCDA. We merged diverse data within the implementation outcome domains of feasibility, appropriateness, and acceptability. We applied normalization process theory to categorize the types and amount of work (coherence, cognitive participation, collective action, reflexive monitoring) stakeholders did to adopt the tool and judged how this work was allocated to influence the implementation outcome domains at 6 and 18 months. We used clinician surveys and IP address-based tracking to calculate SCDA usage across the systems over time. After assessing the types and value of work, we developed a targeted, multi-component toolkit.

**Findings:** The three contexts differed at baseline in regards to feasibility, acceptability, and appropriateness of implementation. The work of adopting the tool was allocated in complex and interdependent ways to optimize these domains and systems that did this strategically had higher uptake (1.1, 5.2, 2.9 uses per clinician per month at 6 months; 0.4, 3.8, 2.1 at 18 months, respectively). The resulting toolkit included context self-assessments, webinars, EMR integration guides, video demonstrations, and an implementation team manual.

**Implications for D&I Research:** Toolkits are appealing options for scaling effective interventions across clinical settings because they are easy to take up and deploy. We present a theory-based approach for designing toolkits that targets their construction toward reducing the work that stakeholders must do to influence early implementation outcomes important for adoption.

**Primary Funding Source:** National Institutes of Health

### S70 Responding to the challenges of context: adaptation of an implementation strategy and a psychosocial intervention for VA's supportive housing program

#### Megan McCullough^1,2^, Sonya Gabrielian^3,4,5^, Ella Koosis^3^, Bruzios Kathryn^1,6^, Vera Yakovchenko^1^, Jeffrey Smith^7,8^, David Smelson^1,6^

##### ^1^ Center for Healthcare Organization and Implementation Research, Bedford, MA, USA; ^2^ Boston University School of Public Health, Boston, MA, USA; ^3^ VA Greater Los Angeles, Los Angeles, CA, USA; ^4^ University of California, Los Angeles, Los Angeles, CA, USA; ^5^ Implementation Research Institute, Washington University at St Louis, St Louis, MO, USA; ^6^ University of Massachusetts Medical School, Worcester, MA, USA; ^7^ Central Arkansas Veterans Healthcare System, North Little Rock, AR, USA; ^8^ University of Arkansas for Medical Sciences, Little Rock, AR, USA

###### **Correspondence:** Megan McCullough (megan.mccullough@va.gov)

**Background:** Maintaining Independence and Sobriety through Systems Integration, Outreach, and Networking- (MISSION) is an evidence-based intervention that uses peers and clinicians to engage homeless adults with co-occurring mental health and substance use disorders in care. Using implementation facilitation as a strategy, MISSION was implemented in three Department of Veterans Affairs Greater Los Angeles’ (VAGLA) permanent supportive housing programs. However, persistent barriers to implementation--challenging organizational context and complex patient population--demonstrated facilitation’s limitations as a stand-alone strategy. We aimed to adapt our implementation strategy and the MISSION intervention to increase uptake.

**Methods:** We administered organizational readiness to change and implementation climate surveys to VAGLA staff trained in MISSION (n=47). We also conducted semi-structured interviews with a purposive subset of staff (n=24). Our implementation team engaged VAGLA stakeholders in routine consultation meetings and tracked meeting minutes in a standardized template; we conducted semi-structured interviews with implementation team members (n=3) and reviewed facilitation tracking and team meeting notes. The Consolidated Framework for Implementation Research (CFIR) was used to guide the interview questions and data analyses, which facilitated dynamic adaptations to our implementation strategy and the intervention.

**Findings:** Data indicated that VAGLA’s organizational context posed significant barriers to implementing MISSION, despite facilitation. Through the facilitation process, we identified additional implementation strategies needed: 1) development and distribution of educational and reference materials that increase ease of stakeholder adoption; 2) identification and preparation of internal champions; 3) informing of local opinion leaders; 4) network weaving; 5) clinical supervision; 6) partnership with clinical training programs, e.g., social work interns; and 7) promotion of adaptability (intervention tailoring to meet program needs). With the seventh strategy, we initiated adaptations to the intervention itself, separating the sessions performed by peers and clinicians and retrofitting existing services to MISSION to minimize duplication.

**Implications for D&I Research:** This project adds to existing work surrounding adaptations to implementation strategies and evidence-based interventions to meet the complex contextual needs of services for vulnerable populations. Sensitive to the delicate balance between adaptation and fidelity, we are tracking each adaptation to our implementation strategy and the evidence-based intervention and closely following changes in uptake.

**Primary Funding Source:** Department of Veterans Affairs

### S71 Enhancing care of the aged and dying in prisons: the process of going to scale

#### Susan Loeb^1^, Valerie Myers^2^, Brenda Baney^3^, Sophia Strickfaden^2^, Erin Kitt-Lewis^1^, Rachel Wion^1^, Tiffany Jerrod^2^, Janice Penrod^1^

##### ^1^ College of Nursing, Pennsylvania State University, University Park, PA, USA; ^2^ Klein Buendel, Inc., Golden, CO, USA; ^3^ College of Health & Human Development--The Methodology Center, Pennsylvania State University, University Park, PA, USA

###### **Correspondence:** Erin Kitt-Lewis (eak114@psu.edu)

**Background:** Prisons are facing increased demands in caring for aged and dying inmates. Our prior research adapted *Best Practices* in end-of-life (EOL) care for corrections through our Toolkit for Enhancing EOL Care in Prisons—our *New Scale-up Idea*. This was our entrée to improve the care for dying prisoners by fostering a community of learners that stimulated system change. Significant advances in enhancing EOL care were reported; however, there was limited geriatric content and the Toolkit format was cumbersome. Transformation of the Toolkit was essential for *Scale-up*. The purpose of this presentation is to describe *Going to Scale* using our Enhancing Care of the Aged and Dying in Prisons (ECAD-P) project. Our research is guided by the Institute for Healthcare Improvement’s Framework for Going to Full Scale dissemination approach. The framework has five phases: *Best Practice Exists/New Scale-Up Idea, Set-up, Develop the Scalable Unit, Test of Scale-up,* and *Go to Full Scale.*

**Methods:** We embarked on the *Set-up* of a newly branded ECAD-P product, which was funded by a Phase I Small Business Technology Transfer (STTR) grant [R41AG049570]. An Expert Advisory Board (EAB; n=4) included representatives from corrections, geriatrics, and hospice. An environmental scan (n=11), a modified Delphi survey (n=6), and in-person usability study (n=16) were conducted. Analytic approaches included: content analysis; geriatric content identification; and acceptability, feasibility, and usability evaluation using qualitative observation approaches and a validated measure for assessing technology-based products.

**Findings:** The Phase I study established: proof of concept; three prototypical modules (developing *Scalable Unit*); specifications document, and Niche report, which informed the commercialization plans for our current STTR Phase II study [R42AG049570]. In Phase II we continue *Scalable Unit* development, vetting with our EAB and Community Advisory Board, and conducting usability testing prior to launching *Test Scale-up*. Phase III (*Go to Full Scale*) will be informed by Phase II findings.

**Implications for D&I Research:** Expanded testing of the ECAD-P product will: optimize the scalable unit for broader dissemination; establish the effectiveness of the training; provide critical insights relevant to dissemination of the commercial product; and position the research team to study broad dissemination and implementation outcomes.

**Primary Funding Source:** National Institutes of Health

### S72 Fidelity to and adaptation in implementation strategies to support primary care practices in improving cardiovascular preventive care

#### Bijal Balasubramanian^1,2,3^, Deborah Cohen^4^, Shannon Sweeney^5^, Miguel Marino^4^, David Ezekiel-Herrera^4^, Rikki Ward^3^, Leah Gordon^4^, Benjamin Crabtree^5^, Leif Solberg^6^

##### ^1^ University of Texas Health Science Center at Houston, Houston, TX, USA; ^2^ Harold C. Simmons Comprehensive Cancer Center, Dallas, TX, USA; ^3^ UT Health School of Public Health in Dallas, Dallas, TX, USA; ^4^ Oregon Health & Science University, Portland, OR, USA; ^5^ Rutgers Robert Wood Johnson Medical School, New Brunswick, NJ, USA; ^6^ Health Partners Institute, Health Partners, Minneapolis, MN, USA

###### **Correspondence:** Bijal Balasubramanian (bijal.balasubramanian@utsouthwestern.edu)

**Background:** Interventions to support primary care practice improvement are most likely to be successful if they balance **fidelity** to evidence-based interventions with allowing **adaptations** to fit the needs of individual practices. Rigorous assessment of both aspects is needed to evaluate *how and why* an intervention was effective, but few studies have assessed both within the context of a multi-site initiative.

**Methods:** Seven regional cooperatives participating in EvidenceNOW – an initiative spanning 12 states to improve cardiovascular preventive care - implemented interventions providing external support to 200 primary care practices in each region (n=1400 practices). Disparate data to assess fidelity and adaptation were collected by each cooperative through facilitator logs-- including date, duration, and mode (email, web, in-person) of encounters with practices—and retrospectively harmonized by the national evaluation team of EvidenceNOW. Qualitative observations were collected through document review, site visits, online diary entries, and semi-structured interviews. Using convergent, mixed-methods design, we compared planned implementation strategies with facilitator log data.

**Findings:** Intervention fidelity and adaptation emerged as a multi-level construct operational at the cooperative and practice levels. At the cooperative level, external practice facilitators were encouraged to use an overarching conceptual framework and specific support activities with each participating clinic, but there was considerable variation in the extent to which cooperatives encouraged and allowed tailoring those activities to the needs and desires of individual practices. At the practice level, facilitator interview and log data showed significant variation across practices in number, duration, and mode of facilitator encounters as well as in types of support activities provided. For example, facilitators in one cooperative delivered the planned 15 visits over the intervention period while others tailored the number and duration of facilitator visits to the needs of the practice so that number of visits ranged from 5 to 48 over one year.

**Implications for D&I Research:** This study demonstrates a method to combine qualitative and quantitative data on fidelity and adaptation from multiple sites that can be used by other implementation initiatives. Rigorously assessing balance between fidelity and adaptations can generate findings about how interventions resulted in intended outcomes, not just that they did.

**Primary Funding Source:** Agency for Healthcare Research and Quality

### S73 How practice context impacts external facilitation strategies to promote implementation of evidence-based guidelines: a comparative case analysis in small independent practice and federally qualified health centers

#### **Donna Shelley**^1^, Allison Pastel^1^, Paulomi Niles^1,2^, Erin Rogers^1^, Deborah Padgett^3^

##### ^1^ New York University School of Medicine, New York, NY, USA; ^2^ New York University Rory Meyers College of Nursing, New York, NY, USA; ^3^ Silver School of Social Work, New York University, New York, NY, USA

###### **Correspondence:** Donna Shelley (donna.shelley@nyumc.org)

**Background:** HealthyHearts NYC, funded through AHRQ’s national EvidenceNOW initiative, is studying the effectiveness of practice facilitation (PF) to support changes in 277 small independent practices (SIPs) and Federally Qualified Health Centers (FQHCs) (<10 clinicians) to improve cardiovascular disease (CVD) related outcomes. We present data on how practice characteristics influence variations in PFs’ roles and strategies for capacity building and quality improvement.

**Methods:** A cross-case comparative analysis. Twelve cases were selected based on practice size (3 FQHCs and 9 SIPs), borough, and baseline composite CVD outcome measure. Sites were recruited from two practice networks, Community Health Care Association of NY State (CHCANYS) and NYC Department of Health’s Primary Care Information Project (PCIP). Informed by Dogherty’s taxonomy of facilitation and the Consolidated Framework for Implementation Research, qualitative data collection included semi-structured PF interviews (n=12), provider and staff interviews (n=24), and one ethnographic site observation (n=12).

**Findings:** Differences in PF strategies for FQHCs and SIPs reflecting differences in infrastructure and capacity included: 1) team-based approach in FQHCs vs. PFs working with frontline doctors or staff in SIPs; 2) PF role differences, *external facilitator* for FQHCs vs. *clinician extender* in SIPs; 3) focus on internal community linkages for FQHCs vs. external linkages for SIPs and; 4) basic EHR use to support visit-based care vs. EHR innovations to support population health management. Similarities among both SIPs and FQHCs included challenging patient populations (immigrants, mentally ill), data mistrust, lack of EHR expertise/optimization, and financial insecurities/pressures.

**Conclusions:** PFs require a remarkable wide range of skills that include: leadership, communication and interpersonal skills and fluency with EHR functionality in order to tailor their approach to practice characteristics and specific challenges faced by SIPs and FQHCs. FQHCs and SIPs face many similar challenges that demonstrate a need for ongoing practice facilitation and support from organizations like CHCANYS and PCIP.

**Implications for D&I Research:** Further understanding of how facilitation enables practice improvement in different settings and the multilevel organizational factors driving PF adaptations will enhance the design and evaluation of strategies for improving adoption of evidence-based practices in the wide range of primary care practice settings in the US.

**Primary Funding Source:** Agency for Healthcare Research and Quality

### S74 Effective strategies for scaling up evidence-based practices in primary care: a systematic review

#### Ali Ben Charif^1^, Hervé Tchala Vignon Zomahoun^1^, Annie LeBlanc^1^, Léa Langlois^1^, Luke Wolfenden^2^, Sze Lin Yoong^2^, Christopher M Williams^2^, France Legare^1^

##### ^1^ CHU de Québec Research Centre - Université Laval, Quebec City, QC, Canada; ^2^ School of Medicine and Public Health, The University of Newcastle, Newcastle, Australia

###### **Correspondence:** Ali Ben Charif (ali.ben-charif.1@ulaval.ca)

**Background:** While an extensive array of existing evidence-based practices (EBPs) has the potential to improve patient outcomes, little is known about how to implement EBPs on a larger scale. Therefore, we sought to identify effective strategies for scaling up EBPs in primary care.

**Methods:** We conducted a systematic review (PROSPERO ID: CRD42016041461) with the following inclusion criteria: (i) *study design*: randomized and non-randomized controlled trials, before-and-after (with/without control), and interrupted time series; (ii) *participants*: primary care-related units (e.g., clinical sites, patients); (iii) *intervention*: any strategy used to scale up an EBP; (iv) c*omparator*: no restrictions; (v) *outcomes*: no restrictions. We searched Medline, PubMed, Embase, PsycINFO, Web of Science, CINAHL, and the Cochrane Library from database inception to August 2016, and consulted clinical trial registries and grey literature. Two reviewers independently selected eligible studies, then extracted and analyzed data following the PRISMA statement. We extracted components of scaling-up strategies and classified them into five categories: infrastructure, policy/regulation, financial, human resources-related, and patient involvement. We extracted scaling-up process outcomes, such as coverage, and provider/patient outcomes. We validated data extraction with study authors.

**Findings:** We included 14 studies. They were published since 2003 and primarily conducted in low/middle-income countries (n=11). Most were funded by governmental organizations (n=8). The clinical area most represented was infectious diseases (HIV, tuberculosis and malaria, n=8), followed by newborn/child care (n=4), depression (n=1), and preventing seniors’ falls (n=1). Study designs were mostly before-and-after (without control, n=8). The most frequently targeted unit of scaling-up was the clinical site (n=11). The component of a scaling-up strategy most frequently mentioned was human resources-related (n=12). All studies reported patient/provider outcomes. Five studies reported scaling-up process outcomes, but no study quantitatively reported a coverage of 80% achieved in combination with a favorable impact.

**Implications for D&I Research:** We found few studies assessing strategies to scale up EBPs in primary care settings. It is uncertain whether any strategies were effective as most studies focused more on patient/provider outcomes and less on scaling-up process outcomes. Minimal consensus on the metrics of scaling-up studies are needed for assessing the scaling up of EBPs in primary care.

**Primary Funding Source:** The Quebec SPOR-SUPPORT Unit

### S75 Development of a pediatric quality measure set for the military health system

#### Terry Adirim^1^, Olivera Jovanovic^2^, Andrew Plummer^3^, Jill Sterling^3^, Thomas Newton^3^, Edmund Chan^4^, Lynch Sean^5^, Kamila Mistry^6^

##### ^1^ Office of the Assistant Secretary of Defense, Health Affairs, Department of Defense, Falls Church, VA, USA; ^2^ OASD-HSP&O, Department of Defense, Falls Church, VA, USA; ^3^ Defense Health Agency, Department of Defense, Falls Church, VA, USA; ^4^ Office of the Assistant Secretary of Defense - Health Affairs, Department of Defense, Falls Church, VA, USA; ^5^ Substance Abuse and Mental Health Administration, Department of Health and Human Services, Rockville, MD, USA; ^6^ Agency for Healthcare Research and Quality, Rockville, MD, USA

###### **Correspondence:** Terry Adirim (ealtaa@comcast.net)

**Background:** The Military Health System (MHS) is an integrated health care system composed of direct care and purchased care that serves over 9.4 million beneficiaries including 2 million children. The current MHS quality measures dashboard includes few measures specific to children and may not reflect the needs of military children.

**Methods:** Internal stakeholders across the MHS were engaged, including pediatric leads for Army, Navy, Air Force and the National Capital Region and as well as federal interagency experts from multiple agencies (e.g. AHRQ, CDC, CMS, HRSA, and SAMHSA) in a collaborative systematic, evidence-informed process to develop a framework and core set of prioritized pediatric measures for implementation. Multiple meetings were convened to identify and consider dimensions essential for a pediatric dashboard. Elements considered include: health care domains; measure concepts, and measure specifications for pediatric health care quality that had potential to address MHS system-level needs. Measures were selected and prioritized primarily through iterative expert reviews using a defined measure prioritization framework. Participants were provided with a set of criteria, informed by published approaches used in the selection of national quality measures. Each iteration of review resulted in the narrowing of measures for consideration removing those deemed aspirational, posing implementation challenges, requiring further testing and validation, not aligning with MHS priorities, and/or viewed as less valuable with regard to their potential impact to improve health care delivery and outcomes of the MHS pediatric population.

**Findings:** The final result is a consensus-based core set of 26 quality measures for implementation that is tailored to the unique needs of the MHS pediatric population. During the process, new methodology for measures selection, a pediatric quality measure framework and an inventory of pediatric health care performance measures were also developed.

**Implications for D&I Research:** The MHS engaged in a novel systematic, data driven, evidence informed process that used active stakeholder engagement from project onset through its conclusion to align core measures with existing system-level needs and health care priorities of military children. Our evidence informed process of measures selection is generalizable to other integrated health care systems.

### S76 Using an array of implementation strategies to improve success rates of pharmacist-initiated medication therapy management services in community pharmacies

#### Rachel Stafford^1^, Jeremy Thomas^1^, Nalin Payakachat^1^, Tiffany Diemer^2^, Michele Lang^2^, Brooke Kordsmeier^2^, Geoffrey Curran^1^

##### ^1^ University of Arkansas for Medical Sciences, Little Rock, AR, USA; ^2^ Pharmacy, The Kroger Company, North Little Rock, AR, USA

###### **Correspondence:** Rachel Stafford (rastafford@uams.edu)

**Background:** With the introduction of the Center for Medicare and Medicaid Services (CMS) Star Measure program has come a greater expectation of implementing Medication Therapy Management (MTM) services in community pharmacies. To meet the growing demand of these services, pharmacies have sought out various methods of supporting pharmacists to provide MTM. Known barriers for completing MTM include inadequate time to complete the services, insufficient staffing, lack of sufficient compensation, billing difficulty, lack of interest among patients (including refusing MTM services when offered), inadequate training/experience, and lack of support from management. The objective of this study was to evaluate the impact of a financial incentive alone versus the incentive plus a package of individualized implementation strategies on the success rate of MTM services.

**Methods:** To increase MTM completion rates the Kroger Company implemented a financial incentive for pharmacists in one of its market services areas (29 supermarket pharmacies). The following year the incentive was continued and an array of training and support strategies were implemented—a 4-hour active-learning MTM training (covering MTM services and recommended workflows), setting a monthly MTM goal for each pharmacist, weekly prompts, monthly progress reports (audit and feedback), and facilitation by a visiting clinical team. The numbers of MTM claims and success rates for 2013 (no intervention), 2014 (financial incentive alone), and 2015 (incentive plus required training and ongoing support) were compared.

**Findings:** A total of 7,038 claims were extracted from 2013-2015. The number of completed claims had increased from 1,385 in 2013 to 3,265 in 2015. The total MTM success rates rose significantly from 42.9% in 2013 to 49.0% in 2014 (p= .001) and to 64.0% in 2015 (p< .001).

**Implications for D&I Research:** A financial incentive plus a package of individualized implementation strategies increases success rates of MTM services compared to a financial incentive alone. These findings should be of use both to pharmacies and pharmacy systems looking to improve their MTM services and to implementation scientists interested in the utilization of implementation strategies in a currently under-studied location, community pharmacies. A follow-up qualitative study is being used to determine stakeholder perspectives on the relative impact and importance of strategies.

### S77 Shattering the wall of silence: evolution from deny and defend to communication and resolution

#### Timothy McDonald (t.b.mcdonald1315@gmail.com)

##### Center for Open and Honest Communication in Health, MedStar Institute for Quality and Safety, Columbia, MD, USA

**Background:** In 1999, Kraman and Hamm reported on an unlikely risk management strategy, namely being open and honest with patients after medical harm. Since then, a handful of innovators from academic medical centers in California, Illinois and Michigan have demonstrated reductions in medical liability by adopting early resolution, disclosure, and more recently communication and resolution programs (CRP).

**Methods:** We describe the evolution of medical liability and risk management models employed within health centers and systems with closed medical staffs and which are self-insured. Medical liability and patient safety related outcomes reported in the published literature are presented along with the evolution of CRP from 1999 to 2016. We examined the impact of different models of medical liability and risk management from academic medical centers within the published literature, their impact on outcomes of liability and risk, and their potential to improve healthcare safety.

**Findings:** Evidence is clear, being open and honest with patients and family members early after patient harm events including medical error reduces medical liability while improving patient safety. The first evidence of improvement observed by all innovators was an increase in event reporting. Second, organizations adopting a CRP approach demonstrate significant reductions in claims, lawsuits, and costs related to medical liability (including excess insurance, defense costs). Time to closure of claim and the proportion of the settlement going to the patient and family also improve with early communication with patients and families after harm. In a demonstration project, we examined the feasibility of taking CRP to community hospitals. Of the 9 hospitals (from 3 health systems) engaged in the demonstration project, only one hospital was able to successfully adopt CRP, conduct early communication, and has sustained implementation. Leadership and the inability to influence physician insurers to participate in CRP prevented full adoption of all elements of the CRP program.

**Implications for D&I Research:** There are systemic challenges preventing the diffusion of CRP despite decades of evidence to their effectiveness. The impact of policy, payer reform, healthcare professional credentialing and training, stagnant health system leadership and insurance reform all culminate in creating an intractable barrier to change.

**Primary Funding Source:** Agency for Healthcare Research and Quality

### S78 A systems approach to communication and resolution program implementation

#### David Mayer (david.b.mayer@medstar.net)

##### Quality and Safety, MedStar Health, Columbia, MD, USA

**Background:** Evidence from academic health centers indicate that effective processes to promptly communicate with patients and families after harm events are linked to reductions in claims, lawsuits, and improved patient safety profiles. Despite evidence from early adopters and widespread support from patients, communication and resolution programs remain the exception in healthcare. We report on the early patient safety and medical liability outcomes from a large community health system in the three years after implementing the AHRQ Communication and Optimal Resolution (CANDOR) program.

**Methods:** CANDOR implementation across 10 hospitals of the MedStar Health system (MSH) was initiated in April 2014. The CANDOR program effectively links event reporting, event review and investigation, early communication with patients and families, care for the care team, and rapid resolution of patient harm events, including disclosure of medical error to enhance patient safety within an organization. Enhancements to published CRP models included integration of human factors and high reliability organization principles, and patient and family engagement as foundational to the implementation process. We describe the early medical liability and patient safety outcomes observed from April 2014 through June 30, 2017.

**Findings:** CANDOR implementation resulted in sustained improvements in adverse event reporting. Reports increased from a low of 968 reports per month in the year preceding implementation to 2,500 reports per month post-implementation. Importantly, CANDOR implementation resulted in a 44% reduction of serious safety events compared to pre-implementation. This reduction occurred concurrent with a 30% increase in clinical activity across the health system. From 2014 to 2016, hospital safety culture improved, resulting in improvements in 11 or 12 domains of the AHRQ hospital survey on patient safety culture. During the 3-years after CANDOR implementation, the health system has reported a growing reduction in medical liability costs, yielding a nearly $37M savings in FY2017.

**Implications for D&I Research:** Despite encouraging results within the first three years post-CANDOR implementation, challenges to widespread adoption remain. Continued work on policy, engaging physician liability insurers, and further engagement of patients as partners is needed to move the field from innovation to early adopters.

**Primary Funding Source:** Agency for Healthcare Research and Quality

## Global Dissemination and Implementation

### S79 Applying the consolidated framework for implementation research (CFIR) to explain performance heterogeneity during Mozambique’s implementation of HPV demonstration projects

#### Caroline Soi^1^, Sarah Gimbel^1,2^, Baltazar Chilundo^3^, Vasco Muchanga^3^, Luisa Matsinhe^4^, Kenneth Sherr^1^

##### ^1^ University of Washington, Seattle, WA, USA; ^2^ Health Alliance International, Seattle, WA, USA; ^3^ Universidade Eduardo Mondlane, Maputo, Mozambique; ^4^ Health Alliance International, Maputo, Mozambique; ^5^ University of Washington, Seattle, WA, USA

###### **Correspondence:** Caroline Soi (soic@uw.edu)

**Background:** Since 2012 Gavi has provided financial support to introduce the HPV vaccine in low and middle income countries (LMIC), however funding has been contingent on establishing a demonstration project prior to national scale-up, in order to gauge effectiveness of delivery models. Although by 2016, most beneficiary countries in Africa had completed demonstration projects, few countries have been able to effectively scale HPV delivery nationwide. An important barrier is the dearth of published, country-specific implementation recommendations. We employed the Consolidated Framework for Implementation Research (CFIR) as a lens to identify drivers of heterogeneous implementation performance during Mozambique’s 2-year demonstration project. Mozambique presents a compelling example as the country conducted demonstration projects in three different districts with extremely different resources, disease burdens, health system capacity and cultural mores.

**Methods:** A post implementation interpretive evaluation was undertaken. Thirty-five key informant interviews were conducted with district and health facility immunization staff, Ministry of Education managers and teachers across the three demonstration districts as well as with central level key informants from MOH, research institutes and immunization program partners. We employed an analysis process previously described by Damschroder, et al.^**1**^ to explain the drivers and barriers to implementation success. Two researchers coded separately and content analysis followed CFIR construct themes.

**Findings:** 18 constructs emerged from informants’ responses as implementation influencers. *Adaptability* was identified as an important construct as delivery modalities needed to meet differing levels of girl school attendance, thus expanding outside of school-based delivery was needed in one district and was challenging. *Available resources* varied across the three sites, with one site receiving direct GAVI support, whilst others received primarily state-based support. These latter sites reported considerably more implementation bottlenecks, manifested in the examples of weaker *structural characteristics* and *organizational incentives*. Health workers beliefs in vaccines’ *relative priority* and *organizational culture* of personal sacrifice to undertake program activities drove performance. Advocacy and social mobilization through *opinion, religious, political leaders and champions* generated higher demand*.*

**Implications for D&I Research:** Our findings demonstrate the expanding applicability of the CFIR in LMICs, with its breadth being a strength that allows for selection of relevant constructs within broader domains, without compromising robustness of results.

**Primary Funding Source:** Gavi, the Vaccine Alliance

### S80 Feasibility, acceptability, and appropriateness of the vaginal menstrual cup for short term non-surgical management of vesicovaginal fistula (VVF) among potential users and stakeholders

#### Nessa Ryan (ryann01@nyu.edu)

##### College of Global Public Health, New York University, New York, NY, USA

**Background:** Vesicovaginal fistula (VVF) is a permanent childbirth injury resulting in urinary incontinence and subsequent psychosocial and economic burden. Traditional management requires surgical repair; however, surgery is mostly inaccessible or delayed leaving women little option to control constant urinary leakage. The vaginal menstrual cup, an insertable, reusable medical device, has recently been implemented for menstrual hygiene management (MHM) in LMICs but has not been evaluated among women with VVF for short-term management of urinary leakage. Feasibility, acceptability and appropriateness outcomes among women with VVF and fistula stakeholders (clinicians, programmers, policy makers, community members) have not been explored.

**Methods:** This work utilized an ethnographic approach to gather pre-adoption implementation data within a sequential exploratory mixed methods design informed by the Consolidated Framework for Implementation Research (CFIR). Results of a survey among women with fistula seeking repair in a health facility in Ghana (N=11) informed additional in-depth interviews in clinical and community settings and an e-survey of stakeholders (N=20). Acceptability among women with VVF was measured by affective (feelings of comfort); cognitive (beliefs of ease of use and of interference with normal activity for self, of utility for others); and behavioral factors (intention to use the cup for longer time). These topics which were expanded upon during in-depth interviews which were analyzed using grounded theory. Stakeholder e-surveys identified facilitators and barriers to implementation.

**Findings:** Acceptability and appropriateness among women was high as most could easily insert (72.73%), remove (72.73%), and comfortably wear (100%) the cup and most would recommend its use. Women used various coping and self-care strategies to manage their condition that will inform cup implementation for women who cannot access surgery or for whom surgery was not successful. Stakeholders revealed various implementation facilitators and barriers.

**Implications for D&I Research:** This study improves our understanding of the inner and outer context of implementing this novel intervention. Lessons learned for enablers and disablers of successful implementation of the menstrual cup for MHM in LMICs can be compared to facilitators and barriers to implementation of the cup for management of fistula in LMICs.

**Primary Funding Source:** New York University

### S81 Understanding the context of implementing cervical cancer screening programs in India using a community based participatory approach

#### Prajakta Adusl^1^, Purnima Madhivanan^2^

##### ^1^ National Cancer Institute, Rockville, MD, USA; ^2^ Florida International University, Miami, FL, USA

###### **Correspondence:** Prajakta Adusl (prajakta.adsul@nih.gov)

**Background:** Cervical cancer is the second most common cancer in India among women aged 30-69 years. Screening for precancerous lesions in population based programs has shown to reduce mortality associated with cervical cancer. Uptake of cancer screening among asymptomatic populations, however, can be affected by several individual and system level contextual factors. Developing an understating of these factors can be critical for the successful implementation and sustainability of screening programs.

**Methods:** Applying a community-based participatory approach, we used the photovoice methodology to explore women’s perceptions about cervical cancer screening programs in the community. We recruited 14 women between 30-60 years, residing in rural villages around Mysore, Karnataka, India. After appropriate consent, each participant was provided with a digital camera and trained in the methods and the ethics of photovoice. During the photo collection, participants met individually with the researchers to discuss the meaning of the photographs using the SHOWED technique (What do you **S**ee here? What’s really **H**appening here? How does this relate to **O**ur lives? Why does this problem **E**xist? What can we **D**o about it?). To expand our qualitative inquiry, we also interviewed 30 physicians and conducted focus groups discussions with 32 community health workers in the same community.

**Findings:** Several themes emerged from the data and are discussed using a socioecological perspective to reveal the context in which cervical cancer screening takes place in a rural Indian community. At the individual level, women at-risk for cervical cancer described considerable misinformation about cancer and cancer screening tests based on their limited understanding. Interactions of the women with family member and peer community groups had important influences towards women participating in the cervical cancer screening programs. Women perceived that lack of female physicians and appropriate health care services were an important barrier in seeking healthcare. At a community level, women reported widespread beliefs of stigma and fear associated with cancer diagnosis prevalent in the community.

**Implications for D&I Research:** Study findings helped identify elements of the social and cultural context of rural communities thereby providing a rich understanding of factors influencing of cervical cancer screening which can be integrated into pre-intervention capacity development in the future.

**Primary Funding Source:** National Institutes of Health

### S82 Scaling up chlorhexidine in Nigeria: systematically implementing and studying a scale-up effort in the “real world”

#### Jenna Wright^1^, Olayinka Umar-Farouk^2^, Jim Ricca^3^

##### ^1^ Broad Branch Associates / Maternal and Child Survival Program, Washington, DC, USA; ^2^ Save the Children / Maternal and Child Survival Program, Abuja, Nigeria; ^3^ Jhpiego / Maternal and Child Survival Program, Washington, DC, USA

###### **Correspondence:** Jenna Wright (jmw206@gmail.com)

**Background:** To accelerate progress reducing neonatal mortality, in 2016 the federal Government of Nigeria (FGN) adopted a five-year plan to scale-up application of chlorhexidine gel to the umbilical cord to prevent newborn sepsis. The USAID-financed Maternal and Child Survival Program (MCSP) is supporting national and state governments in their scale-up planning and implementation, offering an opportunity for systematic examination of the barriers and facilitators to an effective scale-up process in a “real world” environment. MCSP is using the lens of the Consolidated Framework for Implementation Research (CFIR) for an analysis of the experience.

**Methods:** MCSP solicited the opinions of key national, state and external participants about barriers and facilitators to successful scale-up in terms of intervention characteristics, outer setting and inner setting, using a Scalability Checklist and Readiness for Organization Change tool. Identified barriers were mapped to planned implementation processes in national and state plans.

**Findings:** In terms of intervention characteristics, partners identified the main barrier as a common perception among clients and providers that use of the chlorhexidine causes delayed cord separation, interfering with cultural norms. A communication strategy was designed to address this. Inner setting barriers included low capacity of public and private providers to counsel clients and accurately report utilization. Partners addressed this barrier through engaging with opinion leaders like professional associations, hospital managers, traditional birth attendants and community pharmacy vendors to increase their counseling, service delivery, and reporting capacity. The main outer setting barriers identified involved difficulties in the procurement and distribution system. A public-private partnership was established between national and state governments and private sector partners to address these barriers. Additional keystone strategies include the establishment of a multi-stakeholder management team at national and state levels to oversee scale-up and the collection and use of key data for iterative learning and adaptive management by these teams.

**Implications for D&I Research:** Nigeria is applying a theory-driven planning and implementation process to increase the chance of successful scale-up. We’ve established a systematic way to obtain structured information on the progress of scale-up. Early experience shows that this marriage of “systematic practice” with “simplified research” is a feasible paradigm that can be applied elsewhere.

**Primary Funding Source:** USAID

### S83 Barriers and facilitators to task sharing an integrated substance use intervention in a peri-urban, South African HIV care setting

#### Jessica Magidson^1^, Lena Andersen^2^, John Joska^2^, Bronwyn Myers^3^, Christopher Seitz-Brown^4^, Christopher Funes^5^, Kristen Regenauer^5^, Christina Borba^6^, Steven Safren^7^

##### ^1^ Massachusetts General Hospital/Harvard Medical School, Boston, MA, USA; ^2^ University of Cape Town, Cape Town, South Africa; ^3^ South African Medical Research Council, Parow, South Africa; ^4^ University of Maryland, College Park, College Park, MD, USA; ^5^ Massachusetts General Hospital, Boston, MA, USA; ^6^ Boston Medical Center, Boston, MA, USA; ^7^ University of Miami, Miami, FL, USA

###### **Correspondence:** Jessica Magidson (jmagidson@partners.org)

**Background:** Substance use disorders (SUDs) are a barrier to successful HIV treatment and prevention efforts in South Africa—the country with the largest HIV/AIDS burden globally. Yet, there have been few implementation science studies to evaluate how to integrate SUD treatment into HIV care in South Africa using task sharing (e.g., using paraprofessionals for intervention delivery). Using RE-AIM as a study framework, we assessed provider and patient perspectives on barriers and facilitators to task sharing an integrated behavioral intervention to treat SUDs and address antiretroviral therapy (ART) adherence in HIV care.

**Methods:** This first phase of a Type 1 hybrid-effectiveness implementation trial included semi-structured individual interviews with patients and providers at two HIV care sites in Cape Town, South Africa; patients with moderate/severe substance use involvement (WHO-Assist score≥4) and detectable HIV viral load, and HIV and SUD treatment providers across a range of disciplines and roles were enrolled. Participants gave feedback on barriers and facilitators to task sharing an integrated, cognitive behavioral therapy (CBT)-based SUD treatment in this setting. Interviews were transcribed from isiXhosa into English and analyzed with thematic analysis using NVivo v. 11.

**Findings:** Patients (*n*=19) were 54% female, mean age=39.5 (*SD*=8.2), and 100% Black African. Prevalent substances were alcohol and marijuana. Mean WHO-Assist scores for alcohol were 15.75 (*SD*=8.0) and for marijuana 9.6 (*SD*=11.89). Providers (*n*=11) were 82% female, mean age=43.5 (SD=7.6), and 72.3% Black African. They included nurses, physicians, community health workers (CHWs), addiction and adherence counselors. Service-level barriers identified were a lack of trained addiction and mental health providers, stigma related to substance use and HIV/AIDS, and low patient awareness of existing SUD treatment services. Suggestions to address resource constraints included using (1) peers with a history of substance use (“recovery coaches”) as interventionists to deliver brief, integrated CBT-based counseling; and/or (2) CHWs to screen for substance use during routine home visits for patients who not engaged in HIV care to support linkage to both SUD and HIV treatment.

**Implications for D&I Research:** Peers and lay health workers may be useful for addressing substance use and improving HIV outcomes using task sharing in resource-constrained care settings in South Africa.

**Primary Funding Source:** National Institutes of Health

### S84 Qualitative post intervention assessment of implementation effectiveness and sustainability of strategies for implementing tobacco use treatment guidelines in the Vietnam public healthcare system

#### Nancy VanDevanter^1^, Milkie Vu^2^, Trang Nguyen^3^, Nam Nguyen^3^, Donna Shelley^4^

##### ^1^ Rory Meyers College of Nursing, New York University, New York, NY, USA; ^2^ Emory University, Atlanta, GA, USA; ^3^ Institute of Social and Medical Sciences, Hanoi, Vietnam; ^4^ New York University School of Medicine, New York, NY, USA

###### **Correspondence:** Donna Shelley (donna.shelley@nyumc.org)

**Background:** Tobacco cessation services are not widely available in low middle-income countries (LMICs) where smoking rates remain high. In Vietnam 45% of males are current smokers. We recently completed a two-arm randomized control trial comparing the effectiveness of two strategies for implementing tobacco use treatment (TUT) guidelines in community health centers (CHCs) in Vietnam. All sites received training and patient education materials. Intervention sites received a system for referring smokers to a village health worker (VHW) for multisession counseling. Guided by the Consolidated Framework for Implementation Research (CFIR), we conducted a post-intervention qualitative analysis of factors that facilitate or challenge implementation and sustainability of screening and treatment of TU in CHCs in Vietnam.

**Methods:** Semi-structured interviews (N=42) were conducted with healthcare staff and VHWs in 18 CHCs. Interviews were transcribed verbatim and translated. Two investigators analyzed the transcripts using an inductive and deductive approach to develop codes and identify themes relevant to the study research questions.

**Findings:** Interviews explored five domains of the CFIR (Intervention characteristics, Outer and Inner settings, Characteristics of individuals, and Process). Facilitators of program implementation included perceived importance of and need for smoking cessation services, ease of integrating the TUT intervention into routine care, and a high level of satisfaction with the quality of intervention components. Challenges to implementation included patient barriers (e.g., not ready to quit) and limited VHW referral resource in some communes. The main challenge to sustainability is competing Ministry of Health (MOH) priorities that result in a relative lack of resources for TUT (e.g., training/materials) compared with other health programs.

**Implications for D&I Research:** In countries with a political context similar to Vietnam, sustainability of effective implementation strategies will depend on MOH prioritization and an associated commitment of resources, including ongoing training and integration with other programs. A robust planning process resulted in a good fit between current practice and the implementation strategies used to increase adoption of guidelines in CHCs with other programs. The CFIR can provide an important tool for planning and evaluating program implementation and potential for sustainability in a LMIC.

**Primary Funding Source:** National Institutes of Health

### S85 Implementation science for evidence-based integration of chronic care and HIV services in sub-Saharan Africa

#### Christopher Kemp^1^, Bryan Weiner^1^, Kenneth Sherr^1^, Linda Kupfer^2^, Peter Cherutich^1,3^, David Wilson^4^, Elvin Geng^5^, Judith Wasserheit^1^

##### ^1^ University of Washington, Seattle, WA, USA; ^2^ Fogarty International Center, National Institutes of Health, Bethesda, MD, USA; ^3^ National AIDS/STD Control Programme, Ministry of Health, Nairobi, Kenya; ^4^ Global HIV/AIDS Program, The World Bank Group, Washington, DC, USA; ^5^ UCSF, San Francisco, CA, USA

###### **Correspondence:** Kenneth Sherr (ksherr@uw.edu)

**Background:** HIV-related programs are among the first chronic disease services to perform at scale in Sub-Saharan Africa (SSA). However, non-communicable diseases (NCDs) are on the rise across SSA. NCDs are increasingly common among people living with HIV (PLHIV) and threaten the progress of HIV treatment programs. HIV programs offer a rich array of tools, models, and methods that could be adapted to meet the needs of integrated NCD services for PLHIV. IS can play a key role in learning how to most efficiently and effectively leverage HIV program investments to address NCDs. Our objectives were to review the use of IS to support the integration of NCD and HIV services in SSA, highlight key accomplishments and gaps, and articulate research priorities and capacity building needs for the future.

**Methods:** We conducted a PRISMA systematic review of the application of IS methods to integrated NCD and HIV programs in SSA, and used the results to inform development of an HIV/NCD IS research agenda. We searched PubMed, CINAHL, PsycINFO, and EMBASE. Study inclusion criteria included: written in English; evaluating interventions, programs, or services providing preventive and/or therapeutic NCD care through HIV platforms; based in SSA; and reporting at least one implementation outcome.

**Findings:** We screened 1661 studies, and 31 met inclusion criteria. We found that studies used only qualitative, economic, or impact evaluation methods. Only one study used a theoretical framework for IS research. Acceptability, feasibility, and penetration were the most frequently reported implementation outcomes. Adoption, cost, and fidelity were rare; sustainability was not evaluated. The 31 studies evaluated 26 different HIV/NCD integration programs. Half focused on cervical cancer. Over a third dealt with depression. Sixteen offered prevention or screening services, 16 offered referral services, and 13 offered treatment.

**Implications for D&I Research:** IS has a promising role in supporting HIV/NCD integration, although its impact will be limited unless theoretical frameworks, rigorous study designs, and reliable measures are employed. To help support use of implementation science we need to build sustainable IS capacity. Doing so in SSA and supporting IS investigators can help expedite the way forward in HIV/NCD integration.

**Primary Funding Source:** National Institutes of Health

### S86 Planning matters: sustaining evidence-based task-shifting strategies for hypertension control in Ghana

#### Juliet Iwelunmor^1^, Sarah Blackstone^2^, Jacob Plange-Rhule^3^, Joyce Gyamfi^4^, Gbenga Ogedegbe^4^

##### ^1^ Saint Louis University, St. Louis, MO, USA; ^2^ University of Illinois at Urbana Champaign, Champaign, IL, USA; ^3^ Kwame Nkrumah University of Science and Technology, Kumasi, Ghana; ^4^ Center for Healthful Behavior Change, New York University, New York, NY, USA

###### **Correspondence:** Juliet Iwelunmor (iwelunmorj@slu.edu)

**Background:** Task-shifting’ from physicians to nurses has been proposed as one response to the challenge of delivering large-scale, sustainable, and effective hypertension programs in resource-constrained contexts. However, the evidence for sustainability in sub-Saharan Africa is limited, and little is known about the planning process that advances task-shifting strategies on a large scale. This paper focuses on the sustainability planning process for a cluster randomized tasking shifting strategy for hypertension (TASSH) control in Ghana.

**Methods:** This study is guided by the Sustainability Analysis Process (SAP) methodology, a five-step process that enables planning for the sustainability of interventions in resource-constrained settings. It includes: a) an overview of the context; b) system boundaries; c) vision of sustainability; d) sustainability indicators; and e) verification and modification of indicators. We used in-depth interviews and focus group discussions with nurses involved with TASSH as well as key decision-makers within the community health centers, district hospitals and the Ghana Health Services. Research questions focused specifically on the consensus vision for TASSH sustainability and key sustainability indicators to monitor overtime. Data were analyzed by the research team using content analysis.

**Findings:** A total of 32 key stakeholders participated in this study. The findings highlight a common vision for TASSH sustainability (i.e. a *community-based mechanism with a motivated workforce engaged in reducing hypertension burden*) alongside key indicators at the intervention, organization and community levels that are effective, appropriate, and feasible to monitor overtime. At the intervention level, while conducting four annual visits with patients for at least 20 minutes were considered by stakeholders as appropriate and feasible, increasing fruits and vegetable consumptions were considered the most effective for improving patients’ blood pressure control. At the organizational and community/ecologic levels holding annual workshops with key TASSH leaders and nurses at the health systems level as well as with key stakeholders at the Ghana Health Services were considered the most effective, appropriate and feasible strategy for sustaining the TASSH program.

**Implications for D&I Research:** The persistent gap between evidence-based health interventions and actual health care delivery particularly in low-resource settings underscores the critical need to plan for the sustainability of task-shifting hypertension strategies implemented in resource-constrained settings.

**Primary Funding Source:** National Institutes of Health

### S87 Implementation science contributions to, and from, efforts to scale up health technologies in low and middle income countries

#### Brian Mittman^1,2^ (brian.s.mittman@kp.org)

##### ^1^ Kaiser Permanente Southern California, Pasadena, CA, USA; ^2^ QUERI, VA Greater Los Angeles Healthcare System, Los Angeles, CA, USA

**Background:** Implementation scientists have generated a rich body of theoretical frameworks and research approaches in recent years, and continue to leverage these to produce empirical findings, policy and practice guidance, and refinements to the frameworks and approaches themselves. Much of this work has addressed innovative evidence-based practices in health care, public health and social service settings. Less well-studied are implementation challenges related to innovative technologies. While often viewed as somewhat easier to implement than professional and organizational practices (due to the appeal of technological solutions, marketing by commercial firms, etc.), low rates of appropriate implementation and scale-up of health technologies persist and represent gaps in quality and outcomes. Implementation science offers promising tools for understanding and overcoming barriers to adoption and scale-up of health technologies, while studies of adoption and scale-up can help further develop and refine implementation science frameworks, research approaches and empirical evidence.

**Methods:** This presentation offers a summary and assessment of key implementation science frameworks, research approaches and findings as applied to the study of technology scale-up in low and middle income countries (LMICs). The presentation is based on a conceptual analysis of implementation science tools relative to the characteristics of implementation/scale-up challenges and the key features of the technologies of interest (innovations), local settings (internal context) and external context.

**Findings:** Health technologies differ from evidence-based practices in several implementation-related respects, including trialability, degree of adaptability, etc. Health technology scale-up in LMIC settings is challenged by several distinctive features of internal and external context, including infrastructure requirements (staffing, training, space, support, technical expertise), legal and regulatory factors, professional and consumer norms, and others. These factors have important implications for research, policy and practice efforts to understand and overcome implementation and scale-up challenges.

**Implications for D&I Research:** Research on technology scale-up in LMICs offers valuable opportunities to explore domains within implementation science frameworks (e.g., CFIR) that are under-studied in other domains of implementation science. Insights and findings from this research will help enrich the implementation science field by informing research on health technology scale-up in developed countries, including implementation and scale-up in resource constrained settings (e.g., safety net, public).

### S88 Scale up learning and experience to improve global reproductive health programming: ExpandNet’s origin and experience

#### Laura Ghiron^1^, Peter Fajans^1^, Ruth Simmons^1,2^

##### ^1^ Partners in Expanding Health Quality and Access, ExpandNet Secretariat, Davis, CA, USA; ^2^ School of Public Health, University of Michigan, Ann Arbor, MI, USA

###### **Correspondence:** Laura Ghiron (ljghiron@umich.edu)

**Background:** When family planning programs began in the 1950s the general belief was that introduction of new contraceptive technologies would be a panacea. However, failure to deliver the-then newly developed intrauterine device with quality of care throughout India showed that more than technology was needed. Since then evidence from scaling up small-scale research-based initiatives, such as Bangladesh’s Matlab project, demonstrated that well-implemented, quality family planning services can succeed.

**Methods:** As a result of earlier failures, in the early 1990s the World Health Organization developed a three-stage methodology for technology introduction which culminated in scaling up successfully tested approaches. As countries reached this stage, systematic guidance on scale up was needed. During the development of such guidance a global network of public health professionals called ExpandNet was established to advance the science and practice of scaling up.

**Findings:** ExpandNet’s scaling up framework rests on organizational principles from open-systems theory, identifying key elements and strategic choices for planning and implementing scaling up of health technologies and interventions. The need for practical guidance led to the development of tools to help implementers with scaling-up. *Nine Steps for Developing a Scaling up Strategy*, has been used to facilitate participatory strategic planning exercises in over 15 countries. A key lesson emerging is that successful introduction and scale up of specific technologies requires efforts to strengthen the health system and address the socio-cultural context in which they are provided. ExpandNet approaches were applied during expansion from initial to scale up cities/districts during the Urban Reproductive Health Initiatives in Kenya and Senegal which led to course corrections and shifting from a “proof of concept” to a “proof of implementation” approach. This entailed encouraging government to lead implementation and to work within routine program constraints, supporting future sustainability and scalability of the interventions. Increases in contraceptive prevalence were similar to that observed during the NGO-led phase.

**Implications for D&I Research:** How to ensure that health technologies including contraceptives are sustainably integrated into public and private health sectors and their delivery is appropriately adapted to the needs of local populations. Documentation and analysis of the scaling-up process is needed to better understand the determinants of success.

**Primary Funding Source:** Bill and Melinda Gates Foundation

### S89 Learning from three implementation strategies employed by a global NGO

#### Nathaniel Moller (Nathaniel.Moller@jhpiego.org)

##### Innovations, Jhpiego, Baltimore, MD, USA

**Background:** For more than 40 years, Jhpiego has focused on critical health issues in areas such as maternal, newborn and child health, malaria, family planning and reproductive health and has established a presence in more than 40 countries. Recognizing the need to accelerate the development, delivery and impact at scale of new approaches to priority problems, one of Jhpiego’s goals is to mobilize its global presence and technical leadership to work with partners in catalyzing and bringing to scale service delivery, process and technology-based innovations for maximum global health impact. To pursue this goal, several approaches have been utilized.

**Methods:** Three distinct strategies for introducing and scaling up global health technologies will be discussed. These approaches will be augmented by several case studies and will identify challenges and opportunities associated with each strategy.

**Findings:** Specifically, the following approaches will be discussed:

1. Public-Private Partnership (PPP): Partnerships between public and private innovators can bring together complementary skills, increasing the chances of successful implementation of technologies. Jhpiego has experience working with product development organizations, as well as developing products itself that have informed key learnings.
2. Enabling Others Using Innovation Frameworks: Open innovation is increasingly being used as a mechanism for bringing together the perspectives of a broad range of stakeholders to solve intractable problems. Jhpiego has employed several tools to facilitate open innovation challenges and will focus on detailing a framework that was utilized to position external product development organizations for successful implementation and scale-up of new technologies.
3. Creating Culture of Innovation at Country Level: One critical element for ensuring technologies are successfully implemented is a strong health system. Through its expansive in-country networks, Jhpiego utilizes its technical leadership in key health areas to strengthen health systems and create environments that are ripe for innovation and technology implementation. Specifically, the discussion will highlight learnings from Jhpiego’s efforts expanding access to safe surgery.

**Implications for D&I Research:** The discussion will incorporate key takeways from Jhpiego’s experiences implementing technologies in developing countries that can be utilized to better position future technologies for implementation and scale-up, leading to better healthcare and more lives saved.

**Primary Funding Source:** National Institutes of Health

### S90 Understanding sustainability of large donor-funded global health programs: a post-endline assessment of Alive & Thrive in Bangladesh and Vietnam

#### Corrina Moucheraud^1,2^, Haribondhu Sarma^3^, Tran Thu Ha^4^, Tahmeed Ahmed^3^, Nhung Doan^4^, Adrienne Epstein^2^, Jeffrey Glenn^2^, Hoang Hanh^4^, Sharmin Khan Luies^3^, Aninda Nishat Moitry^3^, Denise Payan^1,5^, Mahfuzur Rahman^3^, Md. Tariqujjaman^3^, Tran Thi Thuy^4^, Huong Tran^4^, Tran Tuan^4^, Thomas Bossert^2^, Margaret Kruk^2^

##### ^1^ UCLA Fielding School of Public Health, Los Angeles, CA, USA; ^2^ Harvard T.H. Chan School of Public Health, Boston, MA, USA; ^3^ International Centre for Diarrhoeal Disease Research, Bangladesh, Dhaka, Bangladesh; ^4^ Research and Training Centre for Community Development, Hanoi, Viet Nam; ^5^ RAND Corporation, Santa Monica, CA, USA

###### **Correspondence:** Corrina Moucheraud (cmoucheraud@ucla.edu)

**Background:** What factors shape the sustainability of donor-funded programs; and what program dimensions persist after the end of such donor support? This study is the first to undertake a mixed-methods cross-country post-endline evaluation of sustainability determinants and dimensions. Alive & Thrive (A&T) was an infant and young child feeding (IYCF) program, funded mainly by the Gates Foundation as “proof of concept” to assess whether multipronged action (interpersonal communication, policy change, social mobilization and mass media) could achieve IYCF results at-scale. The official A&T project period with Gates funding in Bangladesh and Vietnam ended in 2014.

**Methods:** In both countries, the interpersonal communication program component was implemented only in certain “intervention” areas; we conducted surveys with health workers and mothers/caregivers in intervention and comparison areas. Key stakeholders participated in qualitative interviews. Data were collected between January-June 2017.

**Findings:** More than two years after the end of this project, there are enduring effects. Caretakers and health workers in intervention areas had significantly higher IYCF knowledge scores than their counterpoints in comparison areas. In Bangladesh, health workers in intervention areas also had significantly higher reported self-efficacy and job satisfaction. Caregivers in both countries’ intervention areas reported higher rates of early initiation of breastfeeding and exclusive breastfeeding until the child is 6 months of age, versus caregivers in comparison areas. In Bangladesh these differences remained statistically significant in multivariable models (controlling for maternal age and educational attainment). Qualitative interviews highlighted important ways to foster sustainability, including the perceived benefits of a project, involvement of regional and organizational program champions, and securing funding for training, monitoring, and evaluation activities. Collaborative partnerships also played a key sustainability role by integrating IYCF practices into ongoing nutrition programs and activities after the end of donor funding.

**Implications for D&I Research:** These findings underscore the complexity of sustainability for large global health programs, and the importance of including sustainability assessments within D&I research. This mixed methods, multi-country study offers an example of how academic, NGO, funder, and government partners can collaborate on D&I research.

**Primary Funding Source:** FHI360/Gates Foundation

### S91 Scaling up breast, cervical and oral cancer screening in the limited resource setting: lessons from economic evaluations in India

#### **Sujha Subramanian**^1,2^ (ssubramanian@rti.org)

##### ^1^ RTI International, North Waltham, MA, USA; ^2^ International Agency for Research on Cancer, Lyon, France

**Background:** There is a growing need to create the evidence-base for implementing and translating findings from clinical trials to make them operational and scalable. Several clinical studies on cervical, breast and oral cancer screening have been conducted and lessons learned from these studies can be extrapolated to assess the cost and benefits of scaling up screening programs.

**Methods:** We developed a detailed framework to translate the benefits, harms and costs from clinical trials to the real world setting by identifying components that will be impacted during scale up. We used outcomes and resource use data from several large randomized screening trials in India as well as lessons learned from pilot programs to draw inferences on the benefits and cost of implementing large scale screening programs. Detailed primary cost data collected from clinical trials were used to generate activity-based costs (final estimates for breast cancer screening study will be available September 2017) that were categorized into fixed and variable components to estimate costs related to scaling up screening as well as implementing integrated screening programs.

**Findings:** The cost of screening using visual inspection (cervical and oral cancer) and clinical breast exams range from $6 to $15. The incremental cost per life years saved ranges from $156 to $750. Key issues in scaling up screening are related to compliance with diagnostic testing and quality of the screening process. The overall cost per person screened is projected to be lower in real world scale-up due to integration synergies and economies of scale; 34% to 51% of the cost though are related to diagnosis and treatment and cannot be reduced with integration.

**Implications for D&I Research:** Extrapolation of total cost from clinical trials to real-world implementation can be misleading and therefore activity-based costs should be used along with projected impacts on access, quality and adherence to guide program implementation. Screening programs incur both fixed and variable costs in implementing core activities and therefore there can be reductions in cost per person screened during scale up. It is important to consider both the impact on cost and effectiveness when scaling up screening programs.

### S92 Assessing the costs and estimating scale-up of testing pregnant women for curable sexually transmitted infections in Botswana

#### Adriane Wynn^1^, Corrina Moucheraud^1,2^, Jeffrey Klausner^3^, Arleen Leibowitz^4^

##### ^1^ UCLA Fielding School of Public Health, Los Angeles, CA, USA; ^2^ Harvard T.H. Chan School of Public Health, Boston, MA, USA; ^3^ UCLA David Geffen School of Medicine, Los Angeles, CA, USA; ^4^ UCLA Luskin School of Public Affairs, Los Angeles, CA, USA

###### **Correspondence:** Adriane Wynn (adriane.wynn@gmail.com)

**Background:** *Chlamydia trachomatis* (CT) and *Neisseria gonorrhoeae* (NG) are among the most common sexually transmitted infections worldwide. CT/NG infections during pregnancy are associated with adverse outcomes, including preterm birth and mother-to-child transmission of HIV – yet most countries do not routinely test pregnant women for CT/NG. Although new technologies have introduced the possibility of testing all pregnant women with highly sensitive, easy to use, rapid tests for CT/NG; cost remains an important system-level barrier to implementation. This study estimated the costs and outcomes of scaling up antenatal CT/NG testing and treatment in Botswana.

**Methods:** We estimated the costs and outcomes that would be associated with three different scale-up strategies: 1) sample collection and processing at the point-of-care, 2) samples sent for processing at centralized laboratories, and 3) a combination approach that would offer point-of-care testing at high-volume sites plus sample pooling (and testing at centralized laboratories) across lower-volume sites. This Excel-based model incorporated data on costs and outcomes from a pilot study of point-of-care CT/NG testing and treatment in Gaborone, Botswana.

**Findings:** Providing point-of-care CT/NG testing would treat the most women and cure the most infections, but at the highest cost (US$ 934/cured infection). The centralized laboratory scenario would see the lowest overall cost but the fewest number of women treated and cured (US$ 529/cured infection), due to loss to follow-up. The mixed scenario had the most favorable cost per outcome ratio (US$ 508/cured infection). Sensitivity analyses indicated that improved treatment rates would be critical for optimizing the costs per outcome for antenatal CT/NG testing.

**Implications for D&I Research:** This study developed an innovative method that can be adapted to estimate the costs and outcomes of scaling up testing and treatment programs targeting a variety of infections in many different settings. We believe the models can be used to generate evidence to help policymakers extrapolate findings from pilot studies and plan for scale-up within health system and budgetary constraints.

**Primary Funding Source:** National Institutes of Health

### S93 NCI’s center for global health-funded research portfolio: exploring implications for future dissemination and implementation research

#### James Alaro, Mishka Cira, Brenda Kostelecky, Gila Neta, Sudha Sivaram

##### National Cancer Institute, Rockville, MD, USA

###### **Correspondence:** James Alaro (james.alaro@nih.gov)

**Background:** As the burden of cancer is projected to increase in low and middle income countries (LMICs), dissemination and implementation (D&I) research can help develop strategies to control and prevent cancer. The National Institutes of Health (NIH), which includes the National Cancer Institute (NCI), support D&I research through grant opportunities that seek to **identify, develop, test, and evaluate** strategies to disseminate and implement evidence-based practices. A recent review of a trans-NIH funded portfolio in D &I research reported that participation by institutions in LMICs is limited. NCI’s Center for Global Health (NCI-CGH) was established in 2011 to advance NCI’s mission internationally, in part, by issuing funding announcements to foster collaborations in global cancer research. The purpose of this analysis was to analyze the NCI-CGH-funded portfolio to identify projects that address D&I research questions, employ D&I research frameworks and methodologies, and identify opportunities to advance D&I research in global settings.

**Methods:** We coded a sample of NCI-CGH grants funded between 2012 and 2016, using an adapted codebook originally developed for the trans-NIH D&I research portfolio. All three authors reviewed the proposals, evaluating the phase of implementation, use of D&I frameworks, employment of D&I study designs, implementation strategies, and measured outcomes, as well as study settings.

**Findings:** Analysis of 23 research projects suggested that 7 (30%) sought to answer D&I research questions. These seven proposed to identify (n=1), develop (n=3), and test (n=3) implementation strategies. Only two projects mentioned D&I frameworks and five measured implementation outcomes. All but one were in healthcare settings. An additional 3 out of the 23 (13%) projects were not D&I research but presented clear opportunities.

**Implications for D&I Research:** Investigators seeking to address cancer control priorities in global settings are asking questions that seek to adapt evidence from research to benefit community health and bring improvements and efficiencies to delivery of cancer prevention and treatment services. This is not always done using tools offered by D&I research. There is need to educate and provide for mentored research opportunities so that both established and early stage investigators in global settings are able to generate context specific data that informs evidence translation.

## Health Policy Dissemination and Implementation

### S94 Organizational- and system-level factors that influence the implementation of shared decision-making – a scoping review

#### Isabelle Scholl^1,2^, Allison LaRussa^1^, Pola Hahlweg^2^, Sarah Kobrin^3^, Glyn Elwyn^1^

##### ^1^ Dartmouth Institute for Health Policy and Clinical Practice, Dartmouth College, Lebanon, NH, USA; ^2^ University Medical Center Hamburg-Eppendorf, Hamburg, Germany; ^3^ National Cancer Institute, Rockville, MD, USA

###### **Correspondence:** Sarah Kobrin (kobrins@mail.nih.gov)

**Background:** Shared decision-making (SDM) is poorly implemented in routine care, despite its inclusion in many clinical practice guidelines and the Affordable Care Act. To date, no studies have synthesized the literature around organizational- and system-level factors that influence the implementation of SDM in routine care. Thus, the aim of this study was to compile a comprehensive overview on organizational- and system-level factors that influence the implementation of SDM in routine care.

**Methods:** We conducted a scoping review using the Arksey & O’Malley framework. The search strategy included an electronic search, combined with a secondary search. We included publications that reported on a project or study that aimed to promote implementation of SDM or other decision support interventions in routine health care. Titles and abstracts were screened and full texts were assessed for eligibility by the review team. We conducted a qualitative thematic analysis of the organizational- and system-level factors that were extracted from the included full-texts.

**Findings:** After screening 7,746 records and assessing 355 full texts for eligibility, 49 publications on 32 implementation projects were included. Most of the implementation projects (N=26) were conducted in the US. Several organizational-level factors were described to influence the implementation of SDM in the different studies. They comprise organizational leadership, culture, teamwork, workflows, resources, and priorities. System-level factors identified to influence the implementation of SDM included aspects of incentivization, education and licensing, and policies and guidelines. Some of the included papers discussed approaches to changing identified organizational- and system-level factors, including reorganization of workflows, implementation of multidisciplinary teams, a push for culture change, a push for new legislation, as well as financial incentives.

**Implications for D&I Research:** A broad range of organizational- and system-level characteristics are found to influence implementation of SDM in routine care. Health care organizations that plan to implement SDM should carefully consider the role of organizational-level characteristics that promote or impede implementation. Using implementation and organizational theory could be a useful way of complementing and addressing the identified factors. Health policy could foster SDM implementation by designing legislation that supports the use of a SDM process, as well as by expediting payment reforms that incentivize SDM performance.

**Primary Funding Source:** Commonwealth Fund

### S95 Evaluating the sustainability of healthcare delivery innovations in Massachusetts community hospitals: the chart investment program

#### A. Rani Elwy^1,2^, Elisa Koppelman^1^, Victoria Parker^1,3^, Trina Johnson^1^, Lisa Chan^1^, Sara Bachman^4^, David Rosenbloom^1^, Jessica Lang^5^, Chris Louis^1^

##### ^1^ Boston University School of Public Health, Boston, MA, USA; ^2^ Center for Healthcare Organization and Implementation Research, Department of Veterans Affairs, Boston, MA, USA; ^3^ Peter T. Paul College of Business & Economics, University of New Hampshire, Durham, NH, USA; ^4^ Center for Innovation in Social Work and Health, Boston University School of Social Work, Boston, MA, USA; ^5^ Massachusetts Health Policy Commission, Boston, MA, USA

###### **Correspondence:** A. Rani Elwy (relwy@bu.edu)

**Background:** Phase 2 of the Community Hospital Acceleration, Revitalization and Transformation (CHART) program invests $60 million into 27 community hospitals with a goal of enhancing their delivery of efficient, effective care and preparing them for a value-based payment environment. One year into this two year program, part of our evaluation focused on the sustainability of each community hospital’s customized innovation to reduce readmissions and/or emergency department revisits among its most vulnerable patients.

**Methods:** In-person interviews with key hospital leaders and staff took place between September-December 2016. Interviews, audio-recorded and transcribed verbatim, examined whether CHART programs produced lasting changes within the hospital and explored patterns in program sustainability. A directed content analysis approach was used to identify data corresponding to 10 sustainability themes. We assessed the reliability and validity of our coding frame through joint coding by four analysts on two transcripts. Each coded transcript was discussed in depth until consensus on coding definitions was reached. Following this process, six analysts independently coded between 30-40 transcripts. Data were checked and entered into the NVivo software package for ease of organization and reporting.

**Findings:** We completed 225 interviews with key stakeholders across all 27 hospitals. We found evidence for hospitals’ expected sustainability of innovations through their successful community partnerships, such as with skilled nursing facilitites (SNFs), community coalitions and physician private practices. These partnerships enhanced the community’s capacity to care for vulnerable patients, thereby reducing readmissions and return ED visits. Hospital leaders indicated that program continuance would be based on perceptions of return on investment as well as improvements in specific metrics such as ED returns for behavioral health patients or readmissions from SNFs. ACO readiness was an impetus for CHART participation, with stakeholders noting the upcoming MA Medicaid ACO. Health information technology was largely viewed as a barrier to program sustainability, due to the additional costs and lack of interoperability across partners.

**Implications for D&I Research:** Assessing sustainability of healthcare delivery innovations during the implementation process allows stakeholders to understand their implementation strengths and create strategies for addressing weaknesses, prior to implementation end.

**Primary Funding Source:** Massachusetts Health Policy Commission, through a contract to Boston University School of Public Health

### S96 Identifying priority strategies for implementing policies on immediate postpartum long-acting reversible contraception in states

#### Charlan Kroelinger^1^, Isabel Morgan^2^, Carla DeSisto^3^, Christi Mackie^4^, Ellen Pliska^4^, David Goodman^1^, Kristin Rankin^3^

##### ^1^ Centers for Disease Control and Prevention, Atlanta, GA, USA; ^2^ Association of Schools and Programs of Public Health, Washington, DC, USA; ^3^ School of Public Health, University of Illinois at Chicago, Chicago, IL, USA; ^4^ Association of State and Territorial Health Officials, Arlington, VA, USA

###### **Correspondence:** Charlan Kroelinger (ckroelinger@cdc.gov)

**Background:** Beginning in 2014, the Association of State and Territorial Health Officials, in partnership with the Centers for Disease Control and Prevention convened a group of states to participate in the Immediate Postpartum Long-Acting Reversible Contraception (LARC) Learning Community (LC), to facilitate cross-state collaboration in implementation of policies increasing access to immediate postpartum LARC. This study identified priority implementation strategies within eight state-defined domains to support successful policy implementation, using interview data from 13 participating state teams.

**Methods:** Semi-structured, qualitative, key informant interviews were conducted with state team members (e.g., state health officials, Medicaid medical directors, maternal and child health directors, family planning directors, hospital administrators, and clinicians) participating in the LARC LC. Audio recordings of key informant interviews were transcribed, and excerpts were organized by state. Implementation strategies were identified within excerpts through independent, double-coding into a web-based application for mixed methods research, with strategies verified through consensus. Qualitative analysis included review and grouping of strategies reported by state, weighting of priority domains, and identification of cross-cutting strategies that intersected between multiple domains.

**Findings:** Leading domains identified by states included provider training, outreach, and reimbursement; stakeholder partnership was identified as cross-cutting, with states describing the role of partnership within each domain. Every state team discussed strategies to offer provider training, including hands-on training and education to address misperceptions about LARC. Twelve state teams reported engaging in or planning outreach efforts, such as toolkit development and dissemination, and public education campaigns. Eleven state teams addressed provider and facility reimbursement by developing resources on device and insertion fee billing and coding. All states leveraged partnerships to support information-sharing, identify provider champions, and pilot immediate postpartum LARC programs in select delivery facilities.

**Implications for D&I Research:** Implementing immediate postpartum LARC policies in states involves leveraging partnerships to develop and apply strategies from the facility-level to the state-level. Successful strategy implementation is based on systems change, through multi-state collaboration, peer-to-peer learning, and strategy sharing activities. Identifying key champions and piloting programs in individual facilities are scalable activities that strengthen state efforts to improve access to immediate postpartum LARC, a public health prevention service for postpartum women.

**Primary Funding Source:** Centers for Disease Control and Prevention

### S97 Harnessing policy to de-implement low value care

#### David Howard (david.howard@emory.edu)

##### Emory University, Atlanta, GA, USA

**Background:** Many costly treatments are adopted into routine practice in the absence of evidence that they help patients. This talk will describe how to harness existing policies to promote the de-implementation of low value care.

**Methods:** Using case studies, I will describe three policies to promote de-implementation.

1. Conduct trials. High dose chemotherapy for breast cancer was rapidly adopted in the 1990s despite concerns about its cost and effectiveness. Based on these concerns, the government funded trials to compare high dose chemotherapy to standard treatment. Using data from the bone marrow transplant registry, I show that negative trial results led to rapid abandonment of this treatment.
2. Decrease payment levels. The Affordable Care Act requires CMS to identify and reduce payment rates for “misvalued services.” I will discuss the evidence on payment rates and low value care generally and present data showing how payment reductions for intensity modulated radiation therapy, a costly form of radiation therapy, affected the use of this treatment in breast cancer patients.
3. Whistleblower lawsuits. Medicare and other payers have a hard time identifying low value care because billing records lack critical details about patients’ conditions. By allowing private parties to receive a large share of damages, the whistleblower provision of the False Claims Act provides incentives for clinical staff with knowledge of overtreatment to divulge it. Using AHRQ’s HCUP data, I will describe how suits alleging overuse of percutaneous coronary intervention and implantable cardiac defibrillators led to reductions in the use of these treatments.

**Findings:** Producing evidence, decreasing payment levels, and supporting whistleblower lawsuits have the potential to reduce low value care.

**Implications for D&I Research:** Most of the policy-related discussion on de-implementing low value care has focused on reimbursement schemes that shift risk to providers, but these schemes often exclude specialists, who care for the most costly, complex patients. Researchers should consider how to harness existing policies to better align evidence and policy in specialty care.

### S98 Evaluating policies for integrating smoking cessation into lung cancer screening delivery

#### Paul Krebs^1^, Steven Zeliadt^2,3^, Hannah Johnson^3^, Laura Feemster^3^, Deborah Klein^4^, Kristina Crothers^5^, David Au^2^, Jaimee Heffner^6^

##### ^1^ NYU School of Medicine, New York, NY, USA; ^2^ Center of Innovation for Veteran-Centered and Value-Driven Care, Veterans Health Administration, Seattle, WA, USA; ^3^ University of Washington, Seattle, WA, USA; ^4^ Swedish Medical Center, Seattle, WA, USA; ^5^ University of Washington Medicine, Seattle, WA, USA; ^6^ Fred Hutchinson Cancer Center, Seattle, WA, USA

###### **Correspondence:** Paul Krebs (paul.krebs@nyumc.org)

**Background:** Medicare’s coverage policy decision for lung cancer screening requires that providers certify they conducted tobacco counseling. Assessing how screening programs have implemented this requirement and the value of this policy may be facilitated by longitudinal administrative tobacco use data in electronic health records (EHR).

**Methods:** We examined EHR data for 6,874 patients who were current smokers at the time of their initial lung cancer screening test at 9 VA Medical Centers between 2013-2016. We examined subsequent updates to tobacco use data 1 year following their index exam through March 2017 to identify the frequency of documented cessation. We also qualitatively explored associations between quit rates and cessation support available to patients receiving screening at the 9 facilities.

**Findings:** Most patients (80.8%) had updated tobacco use information recorded in the EHR following their baseline screen. Overall, 11.3% of current smokers had indicated they had quit smoking within the year. Quit rates varied considerably across the 9 screening centers, ranging from a high of 19.3% to a low of 0.4%. The screening centers with the highest quit rates reported conducting activities to actively promote smoking cessation including enrolling patients in a clinical trial, while the centers with the lowest cessation rates reported relying on providers to individually integrate cessation into lung cancer screening care delivery.

**Implications for D&I Research:** The 11.3% quit rate observed in this study is lower than the quit rate of 23.5% at 3 years reported in the National Lung Screening Trial. These data suggest that reimbursement policies relying on process measures, such as indicating yes/no whether a clinical activity was conducted, are likely to be ineffective implementation strategies, as they do not address the quality of the process and do not incentivize providers to focus on the longer-term outcome of smoking cessation. Novel approaches to using longitudinal EHR data to quantify the outcomes of an intended policy can serve as a valuable measure of implementation quality.

**Change in documented smoking status at 1 year**

**Table:** 

Updated status indicates not currently smoking	779	11.3%
Updated status indicates continued current smoker	4,777	69.5%
No updated tobacco use status	1,318	19.2%

**Primary Funding Source:** Department of Veterans Affairs

### S99 The implementation of the affordable care act provisions regarding smoking cessation treatments in state Medicaid programs

#### Sara McMenamin^1^, Sara Yoeun^1,2^, Helen Halpin^3^

##### ^1^ University of California, San Diego, La Jolla, CA, USA; ^2^ AcademyHealth, Washington, DC, USA; ^3^ School of Public Health, UC Berkeley, Berkeley, CA, USA

###### **Correspondence:** Sara McMenamin (smcmenamin@ucsd.edu)

**Background:** Four sections of the Affordable Care Act (ACA) address the expansion of Medicaid coverage for recommended smoking cessation treatments for: 1) pregnant women (Section 4107), 2) all enrollees through a financial incentive (1% FMAP increase) to offer comprehensive coverage (Section 4106), 3) all enrollees through Medicaid formulary requirements (Section 2502), and 4) Medicaid Expansion enrollees (Section 2001). The purpose of this study was to monitor state Medicaid program progress in implementing these provisions and provide recommendations as to where further guidance might be needed from the Centers for Medicare and Medicaid Services (CMS) to reach full implementation.

**Methods:** From January through June, 2017, data were collected and analyzed from 51 Medicaid programs (50 states plus D.C.) through a web-based survey and review of benefits documents to assess coverage policies for smoking cessation treatments. All 50 states and DC replied for a 100% response rate.

**Findings:** 46 Medicaid programs have increased coverage for smoking cessation treatments post-implementation of the ACA by implementing one or more of the four smoking cessation treatment provisions. 27 of 51 Medicaid programs (53%) had fully implemented Section 4107 by covering comprehensive treatments for pregnant women without cost-sharing. Section 4106 was fully implemented by 10 of the 17 Medicaid programs (59%) that had applied for an increase in their FMAP by offering comprehensive benefits without cost-sharing. All 51 Medicaid programs (100%) had implemented section 2502 by offering coverage for smoking cessation drugs. Of the 32 states with Medicaid expansion programs, only 6 (19%) had fully implemented provisions of Section 2001 by covering comprehensive prevention benefits without cost-sharing or prior authorization. Overall, only 11 Medicaid programs (22%) had fully implemented all applicable smoking cessation treatment provisions.

**Implications for D&I Research:** The ACA was successful in improving and expanding state Medicaid coverage of effective smoking cessation treatments. Yet, more than three-quarters of Medicaid programs have not fully implemented all four provisions. Further guidance from CMS may be needed to achieve full implementation of the provisions regarding Medicaid coverage of recommended smoking cessation treatments.

**Primary Funding Source:** The Robert Wood Johnson Foundation

### S100 The impact of two triggered palliative care consultation approaches on consult implementation in oncology

#### Lisa DiMartino^1^, Bryan Weiner^2^, Laura Hanson^3^, Morris Weinberger^3,4^, Sarah Birken^3^, Katherine Reeder-Hayes^3^, Justin Trogdon^3^

##### ^1^ Research Triangle Institute, Research Triangle Park, NC, USA; ^2^ University of Washington, Seattle, WA, USA; ^3^ University of North Carolina at Chapel Hill, Chapel Hill, NC, USA; ^4^ Durham Veterans Affairs Health Care System, Durham, NC, USA

###### **Correspondence:** Lisa DiMartino (ldimartino@rti.org)

**Background:** Studies show palliative care delivered concurrently with cancer treatment improves outcomes, yet palliative care integration with inpatient oncology is underused. This may be, in part, because effective implementation of palliative care consults in oncology is logistically challenging for healthcare organizations. A promising approach to improve integration is a triggered palliative care consultation (TPCC). This study evaluated the impact of two TPCC approaches on consistency and quality of consult implementation, operationalized as uptake and timeliness, on solid tumor medical and gynecologic oncology services at a large academic hospital.

**Methods:** The study timeframe was January 2010 to June 2016. TPCC in gynecologic oncology began in 2014 and was supported by a single strategy (one-page guideline using clinical criteria to initiate a consult); TPCC in medical oncology began in 2015 and was supported by multiple strategies (e.g. training, chart review to identify cancer patients with metastatic disease and uncontrolled symptoms, clinician prompting). Palliative care consult information was chart abstracted and linked to hospital encounter data. We compared the effect of a single strategy vs. usual care, and multiple strategies vs. a single strategy on implementation (i.e., uptake and timeliness). Difference-in-differences modified Poisson regression models evaluated whether implementation differed after TPCC; we estimated adjusted relative risk (aRR), controlling for patient demographic and clinical characteristics.

**Findings:** Our sample included 5,873 medical oncology and 3,889 gynecologic oncology hospitalizations. Overall, 8.8% of medical oncology and 11.0% of gynecologic oncology inpatient encounters involved palliative care consultation. In regression analyses, TPCC supported by a single strategy in gynecologic oncology was associated with greater uptake vs. usual care (aRR: 1.45, p<.05), and TPCC supported by multiple strategies in medical oncology was associated with greater uptake vs. a single strategy (aRR: 2.34, p<.001). Across all comparisons, TPCC did not have a significant impact on timing of consults.

**Implications for D&I Research:** Our study findings are timely and add to the growing evidence base indicating TPCC can promote the use of palliative care for cancer inpatients. Across two inpatient oncology services, TPCC supported by multiple strategies had the greatest impact on uptake. How the strategies affect the sustained use of palliative care consults remains to be investigated.

**Primary Funding Source:** National Institutes of Health

### S101 Implementation of policy recommendations for shared decision-making about lung cancer screening: patient and clinician perspectives

#### Renda Wiener^1^, Hasmeena Kathuria^2^, Elisa Koppelman^3^, Rendelle Bolton^4^, Karen Lasser^2,3,5^, Belinda Borrelli^6^, Christopher Slatore^7^, David Au^8^, Jack Clark^3^

##### ^1^ Center for Healthcare Organization & Implementation Research, Department of Veterans Affairs, Bedford, MA, USA; ^2^ Boston University School of Medicine, Boston, MA, USA; ^3^ Boston University School of Public Health, Boston, MA, USA; ^4^ ENRM Veterans Hospital, VA HSR&D Center for Healthcare Organization and Implementation Research (CHOIR), Bedford, MA, USA; ^5^ Boston Medical Center, Boston, MA, USA; ^6^ Boston University Henry M. Goldman School of Dental Medicine, Boston, MA, USA; ^7^ Veterans Health Administration, Portland, OR, USA; ^8^ Center of Innovation for Veteran-Centered and Value-Driven Care, Veterans Health Administration, Seattle, WA, USA

###### **Correspondence:** Renda Wiener (renda.wiener@va.gov)

**Background:** Clinical practice guidelines recommend shared decision-making (SDM) for lung cancer screening, and in an unprecedented coverage decision SDM is now required to receive Medicare reimbursement for lung cancer screening. However, little is known about how this policy has been implemented and the degree to which SDM about lung cancer screening is achieved in real-world settings. We sought to characterize patient and clinician impressions of communication and decision-making about lung cancer screening and perceived barriers to achieving SDM in practice.

**Methods:** We conducted a qualitative study entailing semi-structured interviews and focus groups at four hospitals (three Veterans Health Administration, one urban safety net). We enrolled 36 clinicians who refer patients for lung cancer screening and 49 patients who had undergone lung cancer screening in the prior year. Following principles of grounded theory, we analyzed transcripts to characterize communication and decision-making about lung cancer screening. Our analysis focused on the guideline-recommended and policy-required components of SDM and barriers to achieving SDM in real-world settings.

**Findings:** Implementation of policy to integrate SDM for lung cancer screening into clinical practice was haphazard with little organizational support for these efforts. Consequently, clinicians varied in the information shared with patients, and few used decision aids. Most clinicians believed they explained the rationale and gave some (often purposely limited) information about screening’s trade-offs. By contrast, most patients reported receiving little information about screening or its trade-offs; several did not realize the CT was intended as a screening test for lung cancer. Most participants did not perceive that significant deliberation occurred. Although all were aware of the recommendations for SDM, clinicians perceived insufficient time, competing priorities, difficulty accessing decision aids, and limited patient comprehension as barriers to realizing SDM in practice.

**Implications for D&I Research:** Most conversations about lung cancer screening did not achieve guideline-recommended and policy-required SDM with a full discussion of benefits and harms and use of a decision aid. Consequently, many patients remain uncertain about lung cancer screening’s rationale, trade-offs, and process. Without organizational support and infrastructure to promote necessary changes to clinical care, implementation of the policy requirement for SDM surrounding lung cancer screening is unlikely to succeed.

**Primary Funding Source:** Department of Veterans Affairs

### S102 Economics in dissemination and implementation research

#### Todd Wagner^1,2^ (Todd.Wagner@va.gov)

##### ^1^ Health Economics Resource Center, Palo Alto VA Health Care System, Menlo Park, CA, USA; ^2^ Stanford University School of Medicine, Palo Alto, CA, USA

**Background:** Dissemination and Implementation (D&I) research aims to formulate the most effective strategy for implementing evidence-based practices. A critical component of D&I research is economic analysis because it provides insightful data points regarding the cost of implementation, and possible downstream effects (both intended and unintended). With economic data, health care decision makers who are responsible for managing national, regional, or local health care budgets can make informed decisions.

**Methods:** D&I research in the U.S. Department of Veterans Affairs (VA) is conducted through the Quality Enhancement Research Initiative (QUERI), which funds a national network of 15 programs to implement evidence-based clinical practices into routine care. We conducted a needs assessment to understand the needs for economics support across the 15 programs. We interviewed the principal investigators and co-investigators of each program; 14 of the 15 programs participated.

**Findings:** Our needs assessment resulted in three main findings: (A) **Gap in knowledge**. While the majority of the programs believed that addressing the economic questions were critical to the long-term success of the initiatives, only a third of the programs proposed any detailed economic analyses with an economics budget to conduct a cost analysis. (B) **Lack of economic expertise**. Programs expressed lack of investigators with the requisite skill and experience to conduct economic analysis, insufficient funds to include economic analysis as part of their program, and difficulty in recruiting economists, when funds existed. (C) **Confusion on costs to include in economic analysis.** The programs struggled with distinguishing between intervention and implementation costs. There was also uncertainty on how to deal with site level variation in cost.

**Implications for D&I Research:** We have partnered with QUERI to provide economics support for three QUERI programs. In addition, we are developing a toolkit for other D&I programs that want to include economic endpoints. We will share with the audience this toolkit, which includes a library of micro-costing forms, cost analysis plans from past studies, and a recent literature summary on the cost of nurse turnover.

**Primary Funding Source:** Department of Veterans Affairs

### S103 The brokering of research evidence compared to the use of claims in the formulation of federal policies to combat childhood obesity, 2000-2014

#### Matthew Weber, Itzhak Yanovitzky

##### Rutgers University, New Brunswick, NJ, USA

###### **Correspondence:** Matthew Weber (matthew.weber@rutgers.edu)

**Background:** Dissemination and implementation has made significant strides in understanding how research evidence is implemented in policymaking processes. Nevertheless, a more complete understanding of the use of research evidence in the process of policymaking making is needed. This study examines the brokering of research evidence from suppliers into legislation through hearings on U.S. federal policies to curb childhood obesity from 2000-2014.

**Methods:** This work focuses on an analysis of 57 key congressional hearings, as well as the associated congressional records and bills, that took place during the 15-year period, and that focused on legislative activity related to the curbing of childhood obesity. Social networks were created with connections recorded between the suppliers of evidence (legislators and invited experts testifying) and the evidence supplied. In addition, attributes were created representing scope, type (claim vs. evidence), context, and timing of research evidence use. The study used social network analysis to examine the different types of research evidence used, and to examine the types of attributes that impacted the degree to which others would cite research that had been introduced in a hearing.

**Findings:** Social network analysis focused on knowledge brokering; the findings show that certain actors were particularly well-suited to broker research evidence as a result of their perceived expertise or position within the legislative network. In addition, there were distinct differences in the results when the network of claims was considered as compared to the network of evidence use. In many ways, the network of claims was larger and included more policymakers and advocates, but the network of provision of research evidence demonstrated a higher degree of expertise.

**Implications for D&I Research:** The results of this study show a stark difference in the types of evidence being introduced in congressional hearings. In many cases, there are broad networks of claims that are introduced and repeated, but lack actual evidence to support key arguments. On the other hand, there are key knowledge brokers who introduce research evidence and thelp share policy agendas to move policy forward. The attributes of key knowledge brokers are discussed in detail.

**Primary Funding Source:** William T. Grant Foundation

### S104 US mayors’ evidence dissemination preferences: towards evidence-based city policies

#### Jonathan Purtle, Rosie Mae Henson, Amy Carroll-Scott, Jennifer Kolker, Ana Diez Roux

##### Drexel University School of Public Health, Philadelphia, PA, USA

###### **Correspondence:** Jonathan Purtle (jpp46@drexel.edu)

**Background:** City governments have the authority to implement of evidence-based health policies (e.g., mandatory paid sick leave). The spread of these policies can be accelerated by disseminating evidence in ways that reflect the preferences of city mayors and their senior staff, but formative dissemination research has not been conducted with these audiences. The study aims were to: 1) characterize the dissemination preferences of US mayors and their senior staff, and 2) identify differences in dissemination preferences between US mayors and senior staff.

**Methods:** A cross-sectional, multi-modal (telephone, post-mail, e-mail) survey of the mayoral administrations of all US cities with a 2015 population ≥50,000 was conducted in fall-winter 2016. The survey was completed by 101 mayors and 127 senior staff (N=228, response rate=30.1%). The dependent variables were six items that assessed the importance of different features of disseminated evidence and six items that assessed the trustworthiness of evidence from different sources. The primary independent variable was whether the respondent was a mayor or senior staff and covariates were education level and geographic region. Multivariable logistic regression and bi-variate analyses were conducted.

**Findings:** The features of disseminated evidence most frequently identified as very important were evidence telling a story (82.7%), being concise (79.3%), and relevant to constituents (79.0%). Political feasibility (46.6%) was identified as very important least frequently. Universities (83.2%) were identified as very trustworthy most frequently, followed by philanthropies (48.4%), industry (29.9%), constituents (26.5%), and advocacy groups (23.7%). For all six features of disseminated evidence, mayors were less likely than senior staff to assign a rating of very important. The difference was significant for evidence being relevant to constituents (AOR=0.36, 95% CI=0.17, 0.75) and approached significance for evidence about cost-effectiveness (AOR=0.53, 95% CI=0.27, 1.03). Mayors were also less likely than senior staff to rate five-of-the-six dissemination sources as very trustworthy. However, mayors were significantly more likely than senior staff to rate media as very trustworthy (AOR=3.02, 95% CI=1.04, 8.77).

**Implications for D&I Research:** US mayors and their staff have strong, and somewhat heterogeneous, dissemination preferences. Findings can inform the design and testing of dissemination strategies that are tailored to reflect the preferences of elected and appointed city policymakers.

**Primary Funding Source:** The Robert Wood Johnson Foundation

### S105 Academic research-policy translation strategies to improve the use of evidence in health policy development, enactment and implementation: a 3-part embedded case study

#### Beth McGinty^1^, Sameer Siddiqi^1^, Sarah Linden^2^

##### ^1^ Johns Hopkins Bloomberg School of Public Health, Baltimore, MD, USA; ^2^ Welch Center for Prevention, Epidemiology, and Clinical Research, Johns Hopkins University, Baltimore, MD, USA

###### **Correspondence:** Beth McGinty (bmcginty@jhu.edu)

**Background:** Academic institutions have considerable interest in translating the research they produce into policy, but lack evidence-based strategies to do so. Strategies should include concrete, actionable steps that can be widely adopted. Study of academic research-policy translation initiatives that have successfully led to the development, enactment, and/or implementation of evidence-based policy will contribute to the development of such strategies.

**Methods:** We conducted a three-part embedded qualitative case study of academic research-policy translation initiatives that resulted in evidence-based policy: Case 1, a partnership between an advocacy organization and researchers from multiple universities focused on advancing evidence-based firearm policy; Case 2, a single university-foundation partnership to develop evidence-based policies to curb the opioid epidemic; and Case 3, a partnership between researchers from three universities, all in the same state, and a state legislator to develop evidence-based harm reduction legislation. We conducted semi-structured interviews with 25 key-informants across cases: eight researchers, seven policymakers, and ten “intermediaries,” or advocates, foundation representatives, or others who ‘go-between’ researchers and policymakers. We assessed informants’ perceptions of strategies that led to successful research-policy translation (e.g. formal partnership with an intermediary versus no such partnership) and barriers and facilitators to academic research-policy translation. Transcripts were iteratively coded by two analysts using hybrid inductive/deductive coding. Cross-case analyses identified convergent and divergent themes.

**Findings:** Across cases, informants identified engagement with local partners and effective knowledge brokers as key strategies supporting their advancement of evidence-based policy. Perceived/actual conflict about the strength of the evidence among researchers was cited as a key barrier. Case 1 informants perceived an effective intermediary, i.e. the advocacy partner in that case, as a key facilitator to research-policy translation, particularly in the policy implementation phase. Across all cases, informants held divergent views about the role of dissenting stakeholders: some encouraged dissenting opinions, while others felt limiting dissent helped build trust and consensus around sensitive topics.

**Implications for D&I Research:** Study results can inform the development of academic research-policy translation strategies, for example institutional partnerships with intermediary groups. Future research should rigorously evaluate such strategies across a variety of institutional contexts and health issues.

**Primary Funding Source:** Johns Hopkins Bloomberg School of Public Health Faculty Innovation Award

## Models, Measures, and Methods

### S106 When is a replication a replication? An interview study of how intervention researchers manage adherence and adaptations in the research process

#### Ulrica von Thiele Schwarz^1,2^, Knut Sundell^2^, Ulrika Förberg^2,3^, Henna Hasson^2,4^

##### ^1^ Mälardalen University, Västerås, Sweden; ^2^ Karolinska Institutet, Stockholm, Sweden; ^3^ Karolinska University Hospital, Stockholm, Sweden; ^4^ Center for Epidemiology and Community Medicine, Stockholm County Council, Stockholm, Sweden

###### **Correspondence:** Ulrica von Thiele Schwarz (ulrica.schwarz@mdh.se)

**Background**: For an intervention to be considered evidence-based (i.e. EBIs), findings need to be replicated. This may include direct replication (exact copy of the study) or, more often, testing the intervention under different circumstances (such as in a new country). Yet, with variation in context, adaptations may be needed. How this is managed influence the generalisability of EBIs. This study aims to explore the how principle investigators of intervention studies describe what adaptations they make, how these are reported in publications as well as their reasoning about adaptations and adherence when conducting replication studies.

**Methods:** A database of behavioural health intervention studies conducted in Sweden (n=139) was used to identify studies that reported adaptations (n=36). All principal investigators (n=21) were invited to participate in semi-structured telephone interviews and twenty agreed, covering 33 interventions. Manifest content analysis was used to identify types of adaptations, and qualitative content analysis was used to explore reasoning and reporting of adaptations and adherence.

**Findings**: The most common adaptation was adding components and modifying the content to the target population and setting. When reasoning about adaptations and adherence, the researchers were influenced by four main factors: whether their implicit aim was to replicate or improve an intervention; the nature of evidence outlying the intervention such as manuals, theories and core components; the nature of the context, including approaches to cultural adaptations and constraints in delivering the intervention; and the needs of clients and professionals. Reporting of adaptations in scientific journals involved a conflict between transparency and practical concerns such as word count.

**Implications for D&I Research:** Researchers responsible for replicating interventions in a new context face colliding ideals when trying to protect the internal validity of the study while considering adaptations to ensure intervention-context fit. A difference between those emphasising direct replications to achieve stability of knowledge of effects (stressing adherence) and those stressing that interventions need to improve over time (giving room for adaptations) emerged. Overall, the findings raise questions about the role of replications in developing an evidence-base, as well as how generalizability and variation in context should be managed in the evidence-to-practice pathway.

**Primary Funding Source:** Forte

### S107 The research to practice pipeline – evidence-based colorectal cancer screening guidelines: putting them into practice and scaling them up

#### Beverly B. Green^1,2^, Laura Mae Baldwin^1^, Gloria D. Coronado^3^, Jennifer K. Coury^4^, Malaika Schwartz^1^

##### ^1^ University of Washington School of Medicine, Seattle, WA, USA; ^2^ Kaiser Permanente Washington Health Research Institute, Seattle, WA, USA; ^3^ Center for Health Research, Kaiser Permanente Northwest, Portland, OR, USA; ^4^ Quality Improvement, Care Oregon, Portland, OR, USA

###### **Correspondence:** Beverly B. Green (green.b@ghc.org)

**Background:** There is strong evidence that colorectal cancer (CRC) screening decreases CRC mortality. However, only 62% of age-eligible adults are up-to-date with screening, with lower rates among low-income (47%) and Hispanic populations (47%). Evidence-based strategies can increase CRC screening uptake and decrease disparities. However, little is known about whether these strategies can be adapted, scaled-up, and maintained in diverse settings. Hybrid effectiveness/implementation studies provide a framework for accelerating the research to practice pipeline.

**Methods:** We present 3 hybrid effectiveness/implementation studies which demonstrate the continuum from research to broad-scale dissemination. Hybrid 1 studies focus on an optimally delivered implementation strategy. Can it work in an ideal setting? Hybrid 2 studies focus on both implementation and effectiveness outcomes. Can it work in a real-world setting? Hybrid 3 studies focus on factors necessary for scale-up and maintenance. Can it be used widely and are adaptations needed for different settings?

**Findings:** The Hybrid 1 study is a patient-level randomized trial implemented in an integrated healthcare system (4673 patients). The research team used an EHR-linked registry to mail fecal kits to patients for CRC screening. The primary outcome was the effectiveness (CRC screening uptake) compared to usual care. Qualitative assessments explored factors related to implementation success. The Hybrid 2 study is a pragmatic cluster clinic-level randomized trial implemented at safety net clinics (26 clinics, 42,000 patients). The intervention used embedded EHR tools (adapted from hybrid 1) and trained clinic personnel to deliver the mailed interventions. Mixed methods outcomes included adoption, reach, variation in clinic implementation, effectiveness, and maintenance. The Hybrid 3 study is an observational demonstration pilot being conducted by two Medicaid insurance providers (2 states, 11,200 patients) comparing two implementation strategies that address Hybrid 2 implementation barriers. The research team provides evaluation and consultative support only. Mixed-method outcomes include comparative reach, effectiveness, costs, and sustainability of the programs.

**Implications for D&I Research:** We compare strategies for advancing effective implementation of evidence-based clinical guidelines into widespread practice. Hybrid models can be used to inform broad scale dissemination of effective strategies to increase CRC screening and decrease screening disparities. We also make recommendations for improving the model.

**Primary Funding Source:** National Institutes of Health

### S108 Scaling-out evidence-based interventions to new populations and/or health care delivery systems: improving efficiency in implementation process

#### Gregory Aarons^1,2^, Marisa Sklar^1^, Brian Mustanski^3,4,5^, Nanette Benbow^3,5^, C. Hendricks Brown^3,5^

##### ^1^ University of California, San Diego, La Jolla, CA, USA; ^2^ Child and Adolescent Services Research Center, San Diego, CA, USA; ^3^ Northwestern University Feinberg School of Medicine, Chicago, IL, USA; ^4^ Institute for Sexual and Gender Minority Health and Wellbeing, Chicago, IL, USA; ^5^ Center for Prevention Implementation Methodology for Drug Abuse and HIV, Chicago, IL, USA

###### **Correspondence:** Gregory Aarons (gaarons@ucsd.edu)

**Background:** Evidence-based interventions (EBIs) are often adapted to fit different populations or different service settings. Researchers and community stakeholders often raise concern with EBI effectiveness with new populations or in different settings. Does every such EBI adaptation need to be re-evaluated, or can we “borrow strength” from prior effectiveness trials? We introduce a new concept called “scaling-out” in which an EBI is efficiently adapted to a new delivery system or population. We define population fixed/novel system scaling-out as implementation where an EBI is delivered through a different delivery system to the same or very similar population where it has previously been tested. System fixed/novel population scaling-out extends the reach of an existing intervention to a different population within a similar service system. Following the Exploration, Preparation, Implementation, Sustainment (EPIS) and Dynamic Sustainability Frameworks, and modern mediation analysis, we describe methodology to “borrow strength” from results of existing studies to the proposed ecological context, the health delivery system, target population, and characteristics of the EBI itself.

**Methods:** Approaches invoking external validity and cultural adaptation are applied to scaling-out. We present research designs for scaling-out that assess similarity with previous studies so that evidence-based inferences regarding effectiveness can be made with a higher degree of confidence and lower burden.

**Findings:** We propose combining new data with evidence from previous trials. To reduce time and scope of evaluation, we propose to first test for equivalence in core elements of the mediation model leading to proximal targets. A second strategy compares uptake of the EBI in the new versus old context. We illustrate these methods with examples of prevention programs now being adapted for HIV and primary care.

**Implications for D&I Research:** Under a range of conditions, there is reason to expect EBIs adapted to new settings will produce similar impact as they had in previous studies. However, there has historically been great burden and time lag to demonstrate effectiveness with a new population or in a new setting. The proposed scaling-out approach would dramatically reduce the time that EBIs can be evaluated as adapted for new settings and/or populations.

**Primary Funding Source:** National Institutes of Health

### S109 What attributes of context are relevant to dissemination and implementation? Perspectives from healthcare professionals, health system stakeholders, and change agents internationally

#### Janet Squires^1,2^, Ian Graham^1,2^, Alison Hutchinson^3,4^, Kainat Bashir^2^, Anne Sales^5^, Craig Mackie^2^, Kristin Dorrance^2^, John Lavis^6^, Janet Curran^7^, Jill Francis^8^, Susan Michie^9^, Jamie Brehaut^2^, Noah Ivers^10^, Jocelyn Vine^11^, Tom Noseworthy^12^, Michael Hillmer^13^, Jeremy Grimshaw^1,2^

##### ^1^ University of Ottawa, Ottawa, ON, Canada; ^2^ Ottawa Hospital Research Institute, Ottawa, ON, Canada; ^3^ Deakin University, Geelong, Australia; ^4^ Centre for Quality and Patient Safety Research, Monash Health, Melbourne, Australia; ^5^ VA Ann Arbor Healthcare System, Ann Arbor, MI, USA; ^6^ McMaster University, Hamilton, ON, Canada; ^7^ School of Nursing, Dalhousie University, Halifax, NS, Canada; ^8^ School of Health Sciences, City University London, London, United Kingdom; ^9^ University College London, London, United Kingdom; ^10^ Women's College Hospital - University of Toronto, Toronto, ON, Canada; ^11^ IWK Health Centre, Halifax, NS, Canada; ^12^ University of Calgary, Calgary, AB, Canada; ^13^ Ontario Ministry of Health and Long-Term Care, Toronto, ON, Canada

###### **Correspondence:** Janet Squires (jasquires@ohri.ca)

**Background:** Context is increasingly recognized as an important factor in the development, delivery and understanding of dissemination and implementation strategies. However, conceptual clarity about what comprises context is lacking, which has significantly hindered progress in dissemination and implementation research. This study was undertaken as part of a large research program on the development of implementation interventions that consider and address context issues. The purpose of this study was to identify attributes of context, as perceived by healthcare professionals and health system stakeholders internationally, that can facilitate or hinder the use of research evidence in clinical practice and that are relevant to dissemination and implementation interventions.

**Methods:** We conducted a multi-inquiry of context. First, we undertook a secondary analysis of qualitative interviews conducted with healthcare professionals on their perceived barriers and enablers to using research in clinical practice. Second, we conducted semi-structured interviews with health system stakeholders and change agents responsible for implementation interventions, programs, and change processes focused on improving healthcare professionals’ use of research in clinical practice. Both inquires were conducted in four countries: United States, Canada, United Kingdom, and Australia. All interviews were analyzed inductively using constant comparative analysis.

**Findings:** A total of 157 interviews with healthcare professionals across 12 unique datasets and settings were analyzed and resulted in the identification of 129 attributes of context perceived as relevant to dissemination and implementation. We interviewed 39 health system stakeholder and change agents which resulted in 168 attributes of context; 48 of which had not previously surfaced in the secondary analysis of healthcare professional interviews. Combined, the two inquiries provided 172 unique attributes of context perceived relevant to dissemination and implementation activities.

**Implications for D&I Research:** Our findings are useful to both researchers and health system stakeholders involved in dissemination and implementation. Researchers will be able to use the findings to guide *a priori* assessments of context to design implementation interventions that consider context. Health system stakeholders will be able to use our findings to pragmatically guide their implementation efforts and to assess the transferability and scale-up potential of successful dissemination and implementation interventions between different contexts.

**Primary Funding Source:** Canadian Institutes for Health Research

### S110 Comparing controlled implementation trial designs: strengths, limitations, and indications for stepped wedge designs

#### Mark S Bauer^1,2^ (mark.bauer@va.gov)

##### ^1^ Center for Healthcare Organization and Implementation Research, VA HSR&D, Bedford/Boston, MA, USA; ^2^ Harvard Medical School, Boston, MA, USA

**Background:** Controlled implementation trials are essential for advancing the science of implementation. Complexities often arise in implementation situations that preclude the use of traditional randomized controlled clinical trial designs. For instance, randomization is often at the site or provider level, which limits sample size and restricts the numbers of covariates for matching, balancing, or adjusting analyses. Moreover, implementation interventions are frequently complex, constraining how many sites can be supported simultaneously. Additionally, policy considerations may require that all participants receive implementation support. Several of these issues can be addressed, while maintaining scientific rigor, by utilizing randomized stepped wedge implementation designs, which extend support to all participants but randomize the time at which they receive support.

**Methods:** We will stimulate audience discussion by describing an ongoing hybrid type III randomized controlled implementation trial funded by the VA QUERI to support the implementation of evidence-based team care in general mental health clinics. The clinical intervention was technical assistance to establish teams based on the Collaborative Chronic Care Model, while the implementation strategy was blended internal-external facilitation. Nine sites were identified through a national call for participation, and randomized to receive 12 months of implementation support in one of three waves beginning at four-month intervals. Sites in each wave were balanced according to a computer-generated algorithm to minimize confounding by decreasing potential time-trends in site characteristics. Implementation outcomes include provider and patient perceptions of collaborativeness of care, while intervention outcomes included subject-level health outcomes and care satisfaction.

**Findings:** Strengths of the stepped wedge design include the ability to utilize each site as its own control while maintaining inter-site comparisons, analysis of secular trends due to external forces, and ability to commit to provide implementation support to all participating sites. Limitations of the design include inability to fully balance the design (by having intervention precede nonintervention) and the relatively longer protocol duration compared to a pure parallel-groups randomization.

**Implications for D&I Research:** Stepped wedge designs deserve consideration when addressing questions that require intensive implementation support for a relatively small number of sites in which site balance and secular trends are a particular concern.

**Primary Funding Source:** Department of Veterans Affairs

### S111 Comparing controlled implementation trial designs: strengths, limitations, and indications for SMART designs

#### Shawna Smith (shawnana@med.umich.edu)

##### University of Michigan, Ann Arbor, MI, USA

**Background:** Recent years have witnessed a growing menu of effective implementation strategies for enhancing uptake of evidence-based practice (EBP). Implementation strategies vary in the barriers to uptake addressed, as well as cost and intensity. An *adaptive implementation intervention* is a multi-stage implementation intervention that adapts to specific site dynamics using decision rules that recommend how implementation strategies should be modified to optimize EBP uptake. However, implementation scientists may lack information *a priori* to design optimal adaptive sequences. For example, scientists may not know whether it is better to start with the most intensive strategy and step down for responsive sites, or start with the least intensive strategy and step up for non-responsive sites. Sequential multiple-assignment randomized trial (SMART) designs are multi-stage randomized trials wherein each stage corresponds to a critical decision about strategy. SMARTs inform optimal implementation strategy sequences by randomizing sites at each stage to different implementation strategy options.

**Methods:** The Adaptive Implementation of Effective Program Trial (ADEPT) study will be used to illustrate adaptive implementation interventions. ADEPT applied a SMART design to test different sequences of implementation strategies among 78 community-based sites from Michigan and Colorado. Each site initially received training and technical support to implement a Collaborative Care Model (CCM) using Replicating Effective Programs (REP). After 6 months, non-responding sites (<10 patients receiving CCM) were randomized to augment REP with either External Facilitation (EF) or more intensive External + Internal Facilitation (EF/IF). After a further 6 months, non-responsive EF-only sites were re-randomized to continue EF or add IF for another 6 months.

**Findings:** Strengths of SMART designs include the unique informing of sequences of adaptive implementation interventions; personalization of decision rules through effect moderation analyses; and examination of comparative effectiveness of implementation strategies. Limitations include a potential need for a larger number of sites and limited ability to speak to EBP effectiveness.

**Implications for D&I Research:** As the menu of effective implementation strategies continues to grow, the development of sequences of implementation support that are adapted to ongoing site needs offer new opportunities for expediting uptake. SMART designs allow implementation scientists to develop such adaptive implementation interventions.

**Primary Funding Source:** National Institutes of Health

### S112 Comparing controlled implementation trial designs: strengths, limitations, and indications for interrupted time series designs

#### Christine Lu (christine_lu@hphc.org)

##### Harvard Medical School & Harvard Pilgrim Health Care Institute, Boston, MA, USA

**Background:** Health policies are a major topic for investigation in implementation science. Since randomized trials of policies are rarely possible, quasi-experimental studies using longitudinal designs may provide the strongest evidence about intended and unintended policy impacts. Sources of routine data often exist, and appropriate statistical methods are available, that can be used for longitudinal analyses to estimate policy effects.

**Methods:** This presentation will discuss the interrupted time series method for policy evaluation using two policy examples: (1) in 2004, the US Food and Drug Administration (FDA) required that manufacturers add a boxed warning to labels on antidepressant medications regarding evidence of increased risk of suicidality in children and adolescents. (2) In 2009, the FDA mandated a label change for leukotriene inhibitors (LTIs) to include neuropsychiatric adverse events as a precaution. We conducted two separate longitudinal studies using data from multiple health systems to examine the impacts of these two safety warnings on use of medications and health services. In the antidepressant study, the warning was followed by an abrupt and sustained reduction on antidepressant use; simultaneously, there were significant increases in psychotropic drug poisonings (proxy for suicide attempts) among adolescents. In the LTI study, change in labeling was followed by an abrupt decrease in use, but this was not sustained.

**Findings:** Strengths of interrupted time series designs include robustness to many confounds that hinder observational studies including history and maturation biases. Limitations include inability to identify co-interventions as possible confounders. Additionally, while ITS may be used to examine multiple interventions that are rolled out sequentially, this method requires multiple observations before and after each intervention to be stable. Moreover, when policies are implemented, there are often anticipatory effects and/or secondary responses.

**Implications for D&I Research:** Using these two examples, this presentation illustrates the challenges of implementation research. Interrupted time series designs are among the strongest methods for understanding intervention or program impacts. Such quantitative investigations can be coupled with qualitative research to understand the intervention itself and help explain the identified intervention effects.

**Primary Funding Source:** National Institutes of Health

### S113 Operationalizing the Consolidated Framework for Implementation Research into a mixed methods measure: the CFIR Index

#### Mehret Assefa, Mark McGovern

##### Stanford University, Palo Alto, CA, USA

###### **Correspondence:** Mehret Assefa (mehret.assefa@stanford.edu)

**Background:** The Consolidated Framework for Implementation Research (CFIR) is widely used in D&I research; however, in most studies its application has been qualitative and formative. Although essential, advantages to quantitative include accurate measurement and reproducibility. Therefore, we designed and operationalized a quantitative version, the CFIR Index. We report on three different applications of the CFIR Index within three phases: preparation; implementation; and sustainment.

**Methods:** Data was obtained from onsite visits, including interviews, document review, and observations. CFIR Index items were refined, definitions developed, and an administration and scoring guide drafted. The current version (3.0) is a 31-item 5-point rating scale of specific barriers and facilitators across four dimensions: 1) *Perceptions of the Intervention*; 2) *Perceptions of the System and Community*; 3) *Perceptions of the Program*; and 4) *Perceptions of the Clinicians Who Will Use the Intervention*. An original dimension, *Process*, was excluded because of its focus on aspects associated with the implementation strategy. Preliminary psychometric analyses on reliability (internal consistency and inter-rater agreement) and validity (predictive and discriminant) were conducted. CFIR Index data were obtained across three separate NIDA studies and three EPIS phases: 1) Exploration: one organization’s implementation of a multi-component behavioral therapy for youth with SUD; 2) Implementation: forty-nine organizations’ implementation of integrated services for co-occurring SU and MH disorders; and 3) Sustainment: seven organizations’ sustainment of an integrated cognitive therapy for comorbid PTSD and SUD, and an individual addiction counseling approach.

**Findings:** In sustainment and preparation phase studies, reliability of the measure was supported by estimates of inter-rater agreement and internal consistency. Predictive validity was supported by differentiation of high versus low implementation sites, and discriminant validity by observed distinctions between the two implemented practices. In the implementation phase study, *Perceptions of the Program* was highly correlated with organizational capacity of integrated services for co-occurring disorders. Baseline CFIR scores were significantly predictive of the magnitude of increases in organizational capacity from baseline to one year follow-up assessment across both groups.

**Implications for D&I Research:** The CFIR Index appears promising as a useful and replicable measure of contextual mediators and moderators to a D&I project. Standardized measures are critical to the evolution of D&I science.

**Primary Funding Source:** National Institutes of Health

### S114 Leveraging the gap: a mixed methods study employing deviance methodology to understand and improve fidelity to best practices in a childcare nutrition intervention

#### Taren Swindle^1^, Leanne Whiteside-Mansell^1^, Susan Johnson^2^, Geoffrey Curran^1^

##### ^1^ University of Arkansas for Medical Sciences, Little Rock, AR, USA; ^2^ University of Colorado, Anschutz Medical Campus, Aurora, CO, USA

###### **Correspondence:** Taren Swindle (tswindle@uams.edu)

**Background:** Fidelity to the core, evidence-based components of an intervention should increase the size of the effect on outcomes of interest. However, variation in fidelity between implementers is common. Understanding the factors contributing to this variation can inform implementation improvements. Using an explanatory sequential mixed methods design, the purpose of this study was to (a) identify positive and negative deviant cases using quantitative fidelity data from a previous implementation of a nutrition intervention (WISE) in early care and education settings and (b) determine barriers and facilitators to fidelity through qualitative interviews with deviant cases.

**Methods:** Quantitative. A direct fidelity assessment was developed consistent with guidelines from Schoenwald et al (2011). Items indicating fidelity were assessed on a 1 to 4 scale. Data were collected by trained observers in 42 classrooms during each month of the school year. Fidelity scores were created for educators on each of four evidence-based components considered key to the WISE intervention. An aggregate score for each component was created for each teacher. Qualitative. Our qualitative sampling strategy prioritized two groups of educators who were positive deviants or who were negative deviants on all components. Those with variation in fidelity by component were recruited secondarily. We also interviewed directors from 7 agencies reflected in our educator participant pool for a total sample size of 36. We developed an interview guide with questions targeting each construct of the i-PARiHS framework. Rapid coding of the interviews was completed by two coders demonstrating reliability. This led to informed modifications to the interview guide throughout data collection.

**Findings:** Quantitative data identified 3 cases who were positive deviants on all 4 components; 2 were recruited for qualitative interviews. There were 24 cases who were negative deviants on all components; 10 were recruited for qualitative interviews. The remaining cases were positively deviant for some components and negatively deviant on others. Key themes for each component by each i-PARiHS construct will be presented.

**Implications for D&I Research:** The study of cases at the extreme ends of the fidelity spectrum may provide unique perspectives on barriers and facilitators to implementation of evidence-based interventions.

**Primary Funding Source:** National Institutes of Health

### S115 Tracking and assessing use of research evidence in public policymaking processes: a theory-grounded methodology

#### Itzhak Yanovitzky, Matthew Weber

##### Rutgers University, New Brunswick, NJ, USA

###### **Correspondence:** Itzhak Yanovitzky (itzhak@rutgers.edu)

**Background**: Over the past decade, research about the use of research evidence in health policymaking has increasingly recognized that research use unfolds within a social ecology of relationships, organizational settings, and policy contexts that are not fully captured by existing methodologies. Policy documents are frequently used as a source of data on health policymakers’ use of research evidence, but their analysis is mostly focused on tracking citations to research evidence as opposed to tracking use of research evidence as it emerges from the stable practices and norms, or routines, that govern policymakers’ interactions in the policymaking context.

**Methods**: We introduce policy document analysis methodology that combines content analysis with thematic analysis to capture information about the interests and actions of individual policymakers as well as about the policy discourse they engage in. Because policy decisions and actions are principally achieved through persuasion and bargaining, research evidence is typically used in the context of making or refuting arguments. Accordingly, our document coding scheme is theoretically grounded in theories of persuasion and information processing that explain the effect of evidence use on policymakers’ attention to, comprehension, and interpretation of research evidence in the context of policy deliberations.

**Findings**: We demonstrate the application of this methodology to the analysis of a comprehensive set of policy documents (bills, hearings, floor debates, and policy reports) concerning federal policies to decrease childhood obesity from 2000-2014. These documents were coded manually by trained coders to extract variables measuring the scope, type, context, and timing of research evidence use by policymakers. We illustrate how the application of this coding scheme produces a more accurate and nuanced account of use of research evidence by policymakers.

**Implications for D&I Research:** The ability to track and analyze use of research evidence by policymakers is crucial to the development of effective dissemination and implementation strategies. Policy documents provide valid and reliable means to track research evidence use, but only if the analysis “respects” the unique context and aspects of the policymaking process that drive the scope, nature, and timing of research evidence use.

**Primary Funding Source:** William T. Grant Foundation

### S116 Synthesis of complex interventions using hierarchical multivariate meta-regression

#### Kristin J. Danko^1^, Issa J. Dahabreh^2^, Noah M. Ivers^3^, Jeremy M. Grimshaw^1,4^

##### ^1^ University of Ottawa, Ottawa, ON, Canada; ^2^ Center for Evidence Synthesis in Health, Brown University, Providence, RI, USA; ^3^ Women's College Hospital - University of Toronto, Toronto, ON, Canada; ^4^ Ottawa Hospital Research Institute, Ottawa, ON, Canada

###### **Correspondence:** Kristin J. Danko (kdanko@ohri.ca)

**Background:** Dissemination and implementation (D&I) research often involves the evaluation of complex interventions. Standard meta-analysis methods do not adequately capture the effects of the components of complex interventions or allow for the flexible assessment of effect heterogeneity. For example, standard meta-analysis methods typically reduce data into simple pairwise comparisons such that only components that differ between arms contribute to the estimation of intervention effect. The effect of each component is, therefore, estimated from partial data, and the effects of different components cannot be disentangled, making it difficult for decision-makers to correctly identify the most promising interventions for implementation or future research. We explored whether meta-regression methods can better capture the effects of complex interventions.

**Methods:** Using a systematic review of 272 quality improvement trials that assessed at least one of 12 component strategies for diabetes management, we implemented a series of hierarchical models to isolate the effects of the quality improvement strategies. The models can leverage different assumptions depending on background knowledge (e.g., grouping of variants of components into component categories, ignoring co-interventions not captured by coding taxonomy, assuming additivity and linearity of effects). They can be extended to assess interactions between strategies or effect modification by population and contextual factors, and to account for data limitations (e.g., missing data from cluster trials).

**Findings:** Compared to standard methods, our approach is able to isolate the effects of individual components and results in different effectiveness rankings of the 12 strategies. Model extensions to explore interactions and effect modification, as well as to account for missing data, are feasible and produce results that are of substantive interest. The models can be used to generate predictions of the effects of novel combinations of the quality improvement strategies.

**Implications for D&I Research:** Our approach is an improvement over standard meta-analysis methods for assessing the effects of complex interventions. Broader use of these methods can guide future D&I policy and research.

**Primary Funding Source:** Canadian Institutes for Health Research (CIHR)

### S117 A guided discussions method for observing and reflecting on implementation phenomena: a multi-study evaluation of "brief reflections"

#### Erin Finley^1,2^, Alexis Huynh^3^, Melissa Farmer^3^, Bevanne Bean-Mayberry^3^, Tannaz Moin^3,4^, Sabine Oishi^3^, Jessica Zuchowski^3^, Karen Dyer^3^, Alison Hamilton^3,5^

##### ^1^ South Texas Veterans Health Care System, San Antonio, TX, USA; ^2^ UT Health Science Center, San Antonio, San Antonio, TX, USA; ^3^ VA Greater Los Angeles Healthcare System, Los Angeles, CA, USA; ^4^ David Geffen School of Medicine at UCLA, Los Angeles, CA, USA; ^5^ University of California Los Angeles, Los Angeles, CA, USA

###### **Correspondence:** Erin Finley (finleye@uthscsa.edu)

**Background:** Ethnography has been proposed as a valuable method for understanding how implementation occurs within dynamic healthcare contexts, and yet can be time-intensive and costly, making it impractical for use in formative or summative evaluation. The current study assesses feasibility and utility of a pragmatic, ethnographically-informed strategy of guided discussions being used by the VA-funded EMPOWER QUERI to aid in documenting and encouraging reflection on key implementation events, actors, and processes, including adaptation.

**Methods:** The EMPOWER QUERI is conducting three projects to implement innovative care models in VA women’s health for high-priority health concerns – prediabetes, cardiovascular risk, and mental health – according to an adapted version of the Replicating Effective Programs (REP) framework. Drawing on tenets of rapid qualitative research, we developed a semi-structured method of guided “brief reflections”. Reflections are completed as 30-60 minute guided telephone discussions with study team members (including PIs and/or site-based staff) at monthly or bi-monthly intervals, facilitated by a member of the implementation team. Topics include recent activities, challenges, and problem-solving efforts, as well as changes to the intervention, implementation plan, or local/national context. Discussion notes are coded to reflect key domains of interest (e.g., intervention modifications per the Stirman framework), and data are triangulated with other qualitative and quantitative assessments as needed to inform evaluation and implementation activities.

**Findings:** Twenty-three structured reflections were completed during the initial study period, documenting pre-implementation, implementation, and sustainment activities on a periodic basis. Coded reflections provided detailed, near-real-time information on implementation events, actors, adaptations, and context factors; data were distinct from information captured in semi-structured stakeholder interviews conducted at pre- and post-implementation intervals. Reflections created little burden for project teams, and have proven valuable as part of a multi-method strategy for evaluating implementation across widely varying project teams, interventions, and sites.

**Implications for D&I Research:** Few methods exist to aid in operationalizing ethnography in implementation research, and those that do often require significant investment and/or burden for staff and participants, reducing their utility in pragmatic implementation. This semi-structured method shows potential as a feasible and low-burden approach for observing and reflecting on implementation phenomena in complex, multi-site studies.

**Primary Funding Source:** Department of Veterans Affairs

### S118 "How will the Rural Transitions Nurse Program fail?" A brainwriting, premortem exercise to identify barriers to implementation

#### Heather Gilmartin^1^, Brandi Lippman^1^, Emily Lawrence^1^, Marina McCreight^2^, Chelsea Leonard^1^, Ashlea Mayberry^1^, Lynette Kelley^1^, Robert Burke^2^

##### ^1^Denver/Seattle Center of Innovation, Denver VA Medical Center, Denver, CO, USA; ^2^Denver/Seattle Center of Innovation, VA Eastern Colorado Healthcare System, Denver, CO, USA

###### **Correspondence:** Heather Gilmartin (heather.gilmartin@va.gov)

**Background:** Brainwriting is a non-verbal, written group activity intended to generate ideas. Unlike verbal group brainstorming, brainwriting aims to create a psychological safe environment where all feel comfortable to contribute. A pre-mortem exercise is a method to identify threats and weaknesses in a project prior to implementation. In a brainwriting pre-mortem exercise, multidisciplinary healthcare teams are asked to imagine that a project has been rolled-out and failed. The team member’s task is to generate plausible reasons for the project’s failure prior to implementation, which will inform adaptations to the intervention and engage program recipients early in the implementation process. The primary objective of our project was to generate actionable information related to potential opportunities for failure of a Rural Transitions Nurse Program at five diverse VA facilities. Our second objective was to test the feasibility and effectiveness of the group brainwriting pre-mortem exercise in diverse healthcare settings.

**Methods:** Group brainwriting pre-mortem exercises were conducted during site visits at five VA Medical Centers and five primary care sites over three months. Participants were given 30 minutes to write down reasons for program failure, pass documents to others to continue idea generation, and then discuss as a group. Results were mapped to the Practical, Robust Implementation and Sustainability Model (PRISM) framework using a descriptive, exploratory approach.

**Findings:** In total, 116 (range: 2-26) employees participated in the pre-mortem brainwriting exercises, generating a total of 353 ideas which mapped to 20 of 36 PRISM domains. These ideas informed adaptations to the Transitions Nurse Program implementation strategy. Participants reported satisfaction with the activity and work processes of their group, and strongly disagreed that they were afraid to give ideas to the group, or were worried their ideas would be criticized by the others.

**Implications for D&I Research:** Brief, pre-mortem brainwriting exercises are a feasible, effective, and impactful implementation strategy to identify potential failure points and identify solutions to inform intervention adaptations. The method encourages participants to share ideas in a psychologically safe environment, allowing for diverse viewpoints to be recorded, and generating buy-in for novel programs from those who the program will impact.

**Primary Funding Source:** Department of Veterans Affairs

### S119 Enhancing multilevel stakeholder engagement in implementation research: perspectives of implementation scientists

#### Alison Hamilton^1,2^, Susan Frayne^3,4^, Elizabeth Yano^1,2^

##### ^1^ Center for the Study of Healthcare Innovation, Implementation & Policy, VA Greater Los Angeles, Los Angeles, CA, USA; ^2^ University of California Los Angeles, Los Angeles, CA, USA; ^3^ Center for Innovation to Implementation, VA Palo Alto, Palo Alto, CA, USA; ^4^ Stanford University, Stanford, CA, USA

###### **Correspondence:** Alison Hamilton (alisonh@ucla.edu)

**Background:** Engagement of stakeholders at multiple institutional levels is a well-recognized component of effectively implementing best practices in healthcare delivery. Stakeholders (including patients and caregivers, providers, administrators) have a critical role to play across the implementation process, and a variety of models and strategies for stakeholder engagement have been proposed. The objective of this paper is to describe how experienced investigators have engaged stakeholders in their implementation and intervention studies, and what benefits and challenges have been encountered.

**Methods:** We conducted a literature review to identify current and former VA investigators with histories of stakeholder-engaged implementation/intervention research; the initial list was reviewed with VA leadership and additional names were added as participants recommended colleagues. Thirty-one experts nationwide were invited to participate in semi-structured interviews to discuss stakeholder engagement efforts; a total of 29 investigators (17 clinicians, 12 non-clinicians) participated. Interviews were conducted between February-March 2016. Matrix analysis procedures were used to identify strategies, benefits, and challenges.

**Findings:** Participants described their most common strategies for engagement, including eliciting formative feedback on interventions, measures, tools, and methods; advisory committees; incorporating stakeholder advisors (e.g., partners); and including Veterans on research teams. Strategies were typically aimed at engaging stakeholders at multiple levels of the healthcare system and throughout the implementation process. Participants also offered insight into the often-intertwined benefits and challenges of stakeholder engagement, including the importance of learning and listening, remaining attentive to power dynamics, embracing humility, and sustaining relationships over time. Most participants expressed a desire--even a moral imperative--to improve engagement efforts in the dissemination phase of projects.

**Implications for D&I Research:** Stakeholder engagement is a critical component of effective implementation and can aid in: identifying priority research topics; selecting interventions and tools to fit stakeholder needs and contexts; adapting/tailoring implementation strategies; adjusting implementation as needed; promoting buy-in; supporting consumer demand and engagement; addressing barriers and challenges; planning for and achieving sustainability, scale-up, and spread; informing policy and practice; and fostering trust, transparency, and reciprocity. Stakeholder engagement requires time, understanding, commitment, and experience. Added emphasis on developing skills and methods for engaging stakeholders is likely to be of considerable benefit in improving the success of implementation efforts.

**Primary Funding Source:** Department of Veterans Affairs

### S120 Gaining consensus on factors influencing farm to school implementation

#### Eunlye Lee^1^, Carol Smathers^2^, Pat Bebo^2^, Jarrod Dalton^3^, Darcy Freedman^1^

##### ^1^ Case Western Reserve University, Cleveland, OH, USA; ^2^ Ohio State University, Columbus, OH, USA; ^3^ Cleveland Clinic, Cleveland, OH, USA

###### **Correspondence:** Eunlye Lee (exl182@case.edu)

**Background:** Fruit and vegetable intake among US children is consistently below dietary guideline recommendations. Farm to school (F2S) interventions are recommended strategies to improve fruit and vegetable intake among school-aged children. Given variability in readiness and capacity within communities and among practitioners, an assessment tool of community readiness and capacity is a potentially useful means for optimizing F2S intervention implementation. Using consensus modeling methods, this study identified and prioritized factors to inform F2S implementation by public health and community nutrition practitioners working in low-income settings.

**Methods:** Semi-structured interviews (n = 18) and focus groups (n = 23) with community stakeholders (n = 194) in nine Ohio counties were conducted in 2015 to identify facilitators of and barriers to F2S interventions. The consensus modeling approach included five iterative phases: 1) thematic analysis of 41 transcripts; 2) operationalization of themes into measureable indicators; 3) expert panel (n = 14) meeting to prioritize themes and indicators; 4) refinement of themes and indicators; and 5) pilot testing to assess face and content validity.

**Findings:** The consensus modeling approach yielded four themes and 17 indicators associated with community readiness and capacity to implement F2S interventions. The themes represent school capacity (SC), networks and relationships (NR), organizational and practitioner capacity (OPC), and community resources and motivations (CRM). Exemplary indicators respective to each theme are: “To what extent do school food service guidelines in your service area support F2S projects?” (SC), “To what extent are there champions for F2S projects in your service area?” (NR), “To what extent do you spend time each month seeking out or connecting with community stakeholders such as agricultural coordinators, school cafeteria managers, or school wellness committees to increase support for implementation of F2S projects?” (OPC), and “To what extent are parents and students in your service area aware of F2S project opportunities such as school gardens and salad bars at school?” (CRM).

**Implications for D&I Research:** Findings highlight a range of indicators of community readiness and capacity derived from multiple domains needed to support F2S interventions. Results offer guidance for tailoring intervention delivery based on levels of community, practitioner, and organizational readiness and capacity.

**Primary Funding Source:** Centers for Disease Control and Prevention

### S121 Stakeholder perspectives and use of implementation science measurement tools

#### Heather Halko^1^, Cameo Stanick^2^, Caitlin Dorsey^3,4^, Bryan Weiner^5^, Byron Powell^6^, Cara Lewis^3,4,5^

##### ^1^ University of Montana, Missoula, MT, USA; ^2^ Hathaway-Sycamores Child and Family Services, Pasadena, CA, USA; ^3^ Indiana University, Bloomington, IN, USA; ^4^ MacColl Center for Health Care Innovation, Kaiser Permanente Washington Health Research Institute, Seattle, WA, USA; ^5^ University of Washington, Seattle, WA, USA; ^6^ Gillings School of Global Public Health, University of North Carolina at Chapel Hill, Chapel Hill, NC, USA

###### **Correspondence:** Heather Halko (heather.halko@umconnect.umt.edu)

**Background:** Implementation science research has made significant progress toward reducing the research-to-practice gap. However, stakeholders rarely utilize implementation science measures to guide and evaluate implementation of evidence-based practices (EBPs), despite the existence of psychometrically valid and reliable measures. This pattern will likely constrain implementation of EBPs within community settings and warrants further investigation on stakeholder perspectives of measurement in the context of implementation.

**Methods:** Using purposeful sampling techniques, 15 stakeholders involved in EBP implementation in behavioral health settings were interviewed about their use and perceptions of implementation science measures. The Consolidated Framework for Implementation Research (CFIR; Damschroder et al., 2009) and Implementation Outcomes Framework (IOF; Proctor et al., 2011) were used to guide collection and analysis of qualitative data.

**Findings:** Though qualitative data analyses are still ongoing, preliminary results from five transcripts confirmed that few stakeholders use implementation science measurement tools. In fact, most stakeholders responded to questions pertaining to implementation measure use by describing clinical outcome measures solely, or not using measures at all. Stakeholders who endorsed using implementation measures often described using measures of fidelity to assess EBP implementation. Other frequently endorsed themes emphasized the importance of 1) compatibility between measures and organizational context or work flow, 2) structural characteristics of the organization (e.g., size) impacting resource allocation for measurement, 3) quality of leadership engagement in the implementation process, and 4) availability of resources to support and evaluate implementation efforts. Statements related to implementation process codes most often reflected a lack of planning or a lack of formally evaluating implementation success.

**Implications for D&I Research:** Stakeholder use of implementation science measures in community settings remains limited. Preliminary qualitative coding analysis suggests that stakeholders prioritize measures that are compatible with existing workflows, though most stakeholders reported that limited resources or organizational/leadership support deterred their ability to evaluate implementation outcomes. Furthermore, some stakeholders reported their agency having limited knowledge of the types of measures available to assess implementation. Through an identification of barriers and facilitators to measurement use, results could guide targeted efforts to develop, disseminate, and improve use of implementation measures, which would ultimately improve successful implementation of EBPs in community care.

**Primary Funding Source:** National Institutes of Health

### S122 The implementation strategy you cannot go without: how to build partnerships for implementation research

#### Juan Villamar^1,2^, Sheppard Kellam^3^, C. Hendricks Brown^1,2^

##### ^1^ Northwestern University Feinberg School of Medicine, Chicago, IL, USA; ^2^ Center for Prevention Implementation Methodology for Drug Abuse and HIV, Northwestern University, Chicago, IL, USA; ^3^ Johns Hopkins University, Baltimore, MD, USA

###### **Correspondence:** Juan Villamar (juan.villamar@northwestern.edu)

**Background:** A cornerstone of implementation research, including the successful implementation of evidence-based clinical and preventive interventions (EBIs), is a strong partnership between the implementation researcher and a health delivery system, along with it’s delivery agents. Implementation researchers working within a health delivery system identify and use blended implementation strategies that address the following questions: Can an EBI be adopted? Can providers be trained to deliver it? Will train providers choose to deliver it? Will eligible patients receive it? Can the system sustain it? However, the largest cluster of implementation strategies identified by Waltz et al (2015) is the “development of stakeholder interrelationships”, what we call partnership building. Yet, little training exists for implementation researchers on how to develop successful partnerships. We present guidance, with measurable processes, on how to develop partnerships for implementation research.

**Methods:** Based on the public health tenant of “Don’t get kicked out of the community”, Kellam (2012) and Brown (2012) describe a model for developing partnerships in prevention research. Building on this work, we present an implementation strategy to develop partnerships for implementation research. The key principles of this strategy are: 1) Draw the system’s political map and who is required for implementation support; 2) Engage the system from top down, listening first, working through and building trust; 3) Search for mutual-self interests and create a partnership agenda that reflects mutual self-interests 4) Detect internal organizational change as a result of the partnership building process and measure it through unobtrusive measures; 5) Anticipate and manage partnership crises to sustain the partnership.

**Findings:** We discuss the developmental process of developing partnerships for implementation research, how these principles are used in practice with examples, and the role of the implementation researcher in building and measuring the partnership processes.

**Implications for D&I Research:** Research that yields EBIs is not enough to achieve the promise of Health for All. We advance implementation research by providing guidance to implementation researchers for building strong, measurable partnerships for implementation research, supporting other implementation strategies in a blended approach, and improving delivery of EBIs.

**Primary Funding Source:** National Institutes of Health

### S123 Combining and testing organizational and psychological theories to predict implementation

#### David Mandell, Kelsie Okamura, Jessica Fishman

##### University of Pennsylvania, Philadelphia, PA, USA

###### **Correspondence:** David Mandell (mandelld@mail.med.upenn.edu)

**Background:** As a nascent field, implementation science has borrowed constructs and theories from other disciplines, including organizational dynamics and social psychology. The causal models commonly applied in implementation studies derive from theories of behavior change, including the theory of planned behavior. The theory of planned behavior posits that intentions (one’s motivation or willingness to perform an action) are the most proximal determinants of behavior. Intentions are influenced by attitudes, subjective norms and self-efficacy. These models do not focus on specific organizational characteristics that may influence determinants of intention and the pathway between intentions and behaviors. They also were designed to measure specific behaviors (e.g., exercise, food choices), but have been rarely applied to the complex repertoires of behaviors that comprise most psychosocial interventions. Further complicating the use of these theories in implementation science, the associated constructs of intentions, attitudes, norms and self-efficacy have not always been operationalized in implementation research the way originally designed and validated. Perhaps as a result of these complicating factors, empirical findings regarding the utility of these models in predicting implementation has been mixed.

**Methods:** We describe the research our group has conducted to combine theories of behavior change with organizational theory to develop a causal model of implementation, building on a model first proposed by Williams and Glisson. Our model hypothesizes two pathways for organizational influence on individual behaviors. Organizational variables can either directly affect beliefs that influence attitudes, subjective norms and self-efficacy. They also can moderate the association between intentions and behavior.

**Findings:** We describe our work in defining implementation behaviors, testing the predictive validity of the theory of planned behavior in this context, and testing the best fit of organizational variables and pathways. Our data comes from four experimental and observational school-based studies of use of evidence-based practices among ~200 teachers working with students with autism. We relied on standardized measures of specific evidence-based practices of interest, and validated measures of intentions, determinants of intentions, and organizational constructs previously found to be associated with implementation.

**Implications for D&I Research:** This work has the potential to move the field towards causal theories in implementation science.

**Primary Funding Source:** National Institutes of Health

### S124 Methodological challenges and innovations for testing integrated organizational and psychological causal theory in implementation science

#### Nathaniel Williams^1^, Steven Marcus^2^

##### ^1^ Boise State University, Boise, ID, USA; ^2^ University of Pennsylvania, Philadelphia, PA, USA

###### **Correspondence:** Nathaniel Williams (natewilliams@boisestate.edu)

**Background:** The development of integrated, cross-level causal theories that explain how organizational and individual level factors influence clinicians’ implementation of evidence-based practices in healthcare systems presents unique methodological challenges related to model testing. Challenges include the nesting of clinicians within service settings, the presence of antecedents and outcomes at multiple levels, and the need to test complex, cross-level indirect (i.e., mediated) and conditional indirect effects (i.e., moderated mediation) hypothesized as causal mechanisms. Overcoming these methodological challenges is critical to integrating and testing causal theory, identifying mechanisms in implementation science, and to developing optimally effective and efficient implementation strategies. However, a recent systematic review of randomized controlled implementation trials in mental health services found that none of the trials supported a hypothesized mediator and methodological deficiencies related to conceptualizing and testing cross-level mediation were highly prevalent.

**Methods:** As a step toward addressing this gap, we present state-of-the-art methodological “best practices” that implementation scientists can use to meaningfully conceptualize and test multilevel causal theories that integrate organizational and individual-level causal determinants.

**Findings:** Emphasizing a conceptual and applied approach, we demonstrate how to specify and test cross-level mediation models using mixed effects regression analyses (i.e., hierarchical linear models). Further, we describe problems that arise in testing these models and outline state-of-the-art solutions for addressing these challenges. We conclude by discussing the need for further innovation in methods development to more adequately test emerging multilevel causal theories that integrate organizational and individual behavior change constructs to explain implementation.

**Implications for D&I Research:** This presentation will propose concrete strategies investigators can use in their work and present specific examples of these methods from our own research.

### S125 The use of psychological and organizational theories to predict implementation of evidence-based practices in school settings

#### Melanie Pellecchia^1^, Jill Locke^2^, Jessica Fishman^1^, Ming Xie^1^, David Mandell^1^

##### ^1^ University of Pennsylvania, Philadelphia, PA, USA; ^2^ University of Washington, Seattle, WA, USA

###### **Correspondence:** Melanie Pellecchia (pmelanie@upenn.edu)

**Background:** The number of children with autism in special education has increased in recent years, which places a financial burden on public schools. Schools are legally required to provide evidence-based practices (EBPs) for children with disabilities. EBPs for students with autism are intensive, and teachers implement these EBPs with varying levels of fidelity. However, the mechanisms that predict fidelity are not clearly understood.

**Methods:** We tested the ability to predict teacher EBP fidelity of a conceptual model that combines psychological theories of behavior with organizational theory. We will present data that tested this model from a field-based evaluation of the implementation of several EBPs for students with autism in a large urban school district. We queried 67 teachers from 51 schools enrolled in a randomized trial to learn about their intentions to implement EBPs using standardized and validated measures of intentions and determinants of intentions (i.e., attitudes, norms, and self-efficacy). Principals, teachers, and classroom staff also completed standardized measures of organizational culture and climate, implementation climate, and leadership. Teachers’ use of EBPs was calculated using standardized direct observational measures of the specific EBPs of interest.

**Findings:** Regression analyses were used to: (1) predict the association between teachers’ intentions to use EBPs at baseline and their use of EBPs following one year of training; and (2) associations between each organizational-level factor and use of EBPs. Preliminary findings suggest that strength of intentions and determinants of intentions vary considerably across and within classroom staff for different practices. Measures of intentions specific to the practice of interest account for more variance than measures of intentions or attitudes towards EBPs in general. Using a single item measuring intention to use an EBP accounted for 42% of the variance in whether teachers used this EBP. Organizational measures were not as strongly associated with EBP implementation; only supportive and perseverant leadership were significantly associated with EBP use (p=.02 and p=.04, respectively). Leadership both influenced the determinants of intentions (attitudes, norms and self-efficacy) and also moderated the association between intentions and implementation (p=.03).

**Implications for D&I Research:** Results provide evidence for the proposed causal theory.

**Primary Funding Source:** National Institutes of Health

### S126 Using psychological and organizational theories to predict implementation of evidence-based practices in community settings

#### Rinad Beidas^1^, Emily Becker-Haimes^1^, Nathaniel Williams^2^

##### ^1^ University of Pennsylvania, Philadelphia, PA, USA; ^2^ Boise State University, Boise, ID, USA

###### **Correspondence:** Rinad Beidas (rbeidas@upenn.edu)

**Background:** Implementation frameworks posit that clinician and organizational variables are important for successful implementation. Empirical work suggests that clinician knowledge and attitudes, and organizational factors such as organizational culture are associated with implementation of evidence-based practice (EBP). This work has elucidated potential targets for implementation strategies; however it has not identified causal relationships among variables of interest. We build upon this prior research by testing a conceptual model, modified from one proposed by Williams and Glisson, that combines the Theory of Planned Behavior with organizational constructs that affect EBP implementation. The model hypothesizes that proficient organizational cultures, in which clinicians experience norms and behavioral expectations to prioritize improvement in client well-being and exhibit competence in up-to-date treatment models, increase clinicians’ intentions (i.e., motivation) to use EBP, which subsequently results in increased EBP use.

**Methods:** Data were drawn from an observational study in the City of Philadelphia, where a number of EBPs for psychiatric disorders have been implemented over the past decade. We examine intentions to implement parent training techniques for youth with externalizing disorders as a mediator between proficient organizational culture and clinicians’ actual use of parent training techniques in a sample of 145 clinicians nested within 28 organizations. Data was collected in 2015 using gold-standard approaches. Proficient organizational culture was measured using the Organizational Social Context measurement system. Intentions were measured using established question stems developed from the social psychological literature. Use of EBP was measured using the Therapy Procedures Checklist-Family Revised.

**Findings:** Hypotheses were tested via the product of coefficients approach for multilevel mediation which incorporates mixed effects regression models. The indirect effect incorporated an organization-level independent variable (proficient culture), a clinician-level mediator (intentions), and a clinician-level outcome (EBP use), referred to as 2-1-1 mediation. Results supported our hypothesized model; clinicians in more proficient cultures used parent management techniques with youth with externalizing disorders more than clinicians in less proficient cultures and this relationship was partially mediated by clinician intentions (proportion mediated = 38%).

**Implications for D&I Research:** Findings are discussed in terms of their implications for building causal theory in implementation science by marrying psychological and organizational theories of behavior change.

**Primary Funding Source:** National Institutes of Health

## Prevention and Public Health

### S127 Regulations to expand naloxone access and use: citizens’ understanding as a barrier to adoption

#### Dennis Watson, Brad Ray, Lisa Robison, Philip Huynh, Joan Duwve

##### Indiana University-Purdue University Indianapolis, Indianapolis, IN, USA

###### **Correspondence:** Dennis Watson (dpwatson@iu.edu)

**Background:** Opioid overdose is the leading cause of accidental death in the United States. To date, forty states have crafted regulations to expand access and use of naloxone, a medication that quickly reverses an opioid’s effects in the body, as an overdose death prevention strategy. Lay responders (i.e., family, friends, or other users) often arrive at overdoses before emergency services and therefore could save lives with naloxone administration. However, lay responders may be unable to access naloxone due to its status as a prescription medication or unwillingness due to fear of stigma or criminal consequences. In order to address these barriers, Indiana instituted two related policies: The first, expands naloxone access to all citizens who know someone at risk of opioid overdose. The second “Good Samaritan law” protects lay responders from criminal liability and requires health departments to educate individuals before providing the drug. Although well-intentioned, little is known regarding the effectiveness of these measures in encouraging naloxone uptake and use.

**Methods:** We included pre- and post-administration postcard surveys inside 4,527 naloxone kits distributed at local health departments in 36 counties. Kits have been distributed in 3 waves, which began in September of 2016.

**Findings:** 1,279 surveys (1,195 pre- and 84 post-administration) have been returned as of July 7, 2017. Responses demonstrate 18% of lay responders are unaware of the Good Samaritan law. Moreover, 27% of respondents report not calling 911 at the last overdose observed. For those indicating 911 was not called, the most frequent reasons cited were fear of the police (33%) and/or that the person who overdosed did not seem to require additional medical attention after naloxone administration (33%).

**Implications for D&I Research:** Regulations without robust public educational efforts are likely ineffective in increasing naloxone use by lay providers. Stronger efforts to educate the public have potential to improve awareness of the Good Samaritan law, as well as helping lay responders understand that individuals may still require subsequent medical attention after naloxone is delivered. Mistrust of the police is a more difficult barrier to address, as it is likely fueled by lay responders’ prior negative interactions with the criminal justice system.

**Primary Funding Source:** Centers for Disease Control and Prevention

### S128 Expansion and implementation of medication assisted treatment (MAT) in two rural communities

#### Brad Ray^1^, Johanne Eliacin^2^, Dennis Watson^1^

##### ^1^ Indiana University Purdue University Indianapolis, Indianapolis, IN, USA; ^2^ Roudebush VA Medical Center, Indianapolis, IN, USA

###### **Correspondence:** Brad Ray (bradray@iupui.edu)

**Background:** The Indiana Medication Assisted Treatment Project (IMATP) aims to evaluate the expansion and implementation of medication assisted treatment (MAT) (Suboxone or Methadone for opioid use and wrap-around recovery services) in two rural communities in Indiana by examining barriers and facilitators to services for individuals in need of opioid addiction treatment.

**Methods:** We are employing a longitudinal, mixed methods evaluation design that utilizes quantitative and qualitative data to develop a more complete picture of MAT program implementation and performance.

**Findings:** Our findings demonstrate the role of social contexts in implementation efforts of MAT services. Specifically, they identify how various social, cultural, and community factors affect the delivery of MAT in two distinct rural counties that experience elevated rates of opioid addiction and related HIV cases. Our findings indicate that the communities’ negative perceptions of MAT hinder services delivery through limitations of needed infrastructures, organizational resources, and community partnerships. Local cultural views of addictions and perceived appropriate treatment models that involve primarily strict abstinence and sole reliance on religious faith for recovery further undermine the implementation of MAT. Moreover, MAT consists of a wrap-around care model that provides medication as well counseling, healthcare, and other support services to clients at a “one -stop shop.” Based on staff and clients’ feedback, the success of MAT is based on this care model. However, plans to sustain MAT rely primarily on provision of the necessary medication. Without the inclusion of support services that address significant barriers to care, social, and mental health needs of clients, there are concerns that MAT services and implementation may not be as effective.

**Implications for D&I Research:** We provide a case example of how local contexts affect the implementation of addiction services. We find that despite intense media attention and multiple efforts to address the recent opioid epidemic in these communities, efforts to deliver and implement MAT services continue to face steep challenges that are grounded in deeply rooted cultural factors. We discuss our efforts to address these implementation and sustainability barriers within the broader context of opioid policies in Indiana and concerns about the potential criminalization of medication assisted treatment.

**Primary Funding Source:** SAMHSA

### S129 Implementation of planned outreach, intervention, naloxone, and treatment (POINT): an emergency department-based program to prevent fatal opioid overdose

#### Alan McGuire^1,2^, Dennis Watson^2^, Krista Brucker^3^

##### ^1^ Roudebush VA Medical Center, Indianapolis, IN, USA; ^2^ Indiana University-Purdue University Indianapolis, Indianapolis, IN, USA; ^3^ Indiana University School of Medicine, Indianapolis, IN, USA

###### **Correspondence:** Alan McGuire (abmcguir@iupui.edu)

**Background:** Opioid abuse and fatal overdose is a looming public health crisis, yet medication assisted treatment (MAT) is the standard of care for opioid dependence. Nonetheless, substantial barriers prevent patients from receiving and engaging in MAT. Burgeoning evidence demonstrates the emergency room as an acceptable and effective point of intervention with patients with opioid dependence; nonetheless, little is known regarding the critical elements of such an intervention or the process of implementing such a program. The current project documents the implementation and preliminary outcomes of Implementation of Planned Outreach, Intervention, Naloxone, and Treatment (POINT).

**Methods:** Data regarding implementation were collected via semi-structured interviews with program staff, observations of the intervention and team meetings, and review of program materials. Preliminary outcomes were gathered from quality assurance data culled from electronic health records. Qualitative data were analyzed using immersion/crystallization and quantitative data were analyzed descriptively.

**Findings:** Consensus critical program elements include a system to identify appropriate patients; staff embedded in the ED including a program coordinator and peer-provider who engage patients, address barriers to treatment, and link them with MAT; and establishment of a MAT clinic with expedited enrollment for POINT patients. Identified keys to implementation pertained to inner-setting (i.e., ER staff receptive to intervention, local champion), outer-setting (e.g., community addictions/mental health provider as willing partner, patient attitudes toward MAT), and implementation process (build partnerships early). The program was very acceptable (82 out of 116 patients approached were enrolled) and effective (44% of patients presented to at least one follow up appointment and 50% of those who made it to their first appointment were still engaged in treatment at six month follow up).

**Implications for D&I Research:** Results provide a roadmap for other emergency departments wishing to implement a program to link patients at a high risk of death due to opioid overdose with effective treatments. Findings also highlight the importance of engaging stakeholders at multiple system levels in the implementation process.

**Primary Funding Source:** Richard M. Fairbanks Foundation

### S130 Community-academic partnerships for implementation of parenting programs in underserved communities

#### Christina Studts

##### University of Kentucky College of Public Health, Lexington, KY, USA

###### **Correspondence:** Christina Studts (tina.studts@uky.edu)

**Background:** Behavioral parent-training (BPT) is effective in reducing child disruptive behavior problems. However, in rural Appalachia, few families receive BPT despite elevated risk for child behavior problems. In this project, we developed a Community Advisory Board (CAB) and harnessed academic-local public health partnerships to adapt an evidence-based BPT and pilot its implementation.

**Methods:** The Early Childhood CAB, comprised of community stakeholders, convened in 2013. The CAB guided the research team in interpreting data on the perspectives/preferences of community members regarding delivery of BPT. Upon selection of a specific BPT, the CAB reviewed existing training materials and identified aspects requiring adaptation for training of community health workers (CHWs) as interventionists. The research team also initiated partnerships with four local health departments and one local CHW program. Each agency director identified one CHW as interventionist, and the CHWs attended a 40-hour BPT training. CHWs then aimed to each enroll four families in piloting the adapted intervention. To offset agency costs, a one-time payment covered CHW time and travel. Our local partners also agreed to provide implementation data via established measures and key informant interviews. These data include implementation outcomes (feasibility, acceptability, fidelity, and costs) and related factors (characteristics of the inner setting, outer setting, intervention, interventionist, and process) derived from the Consolidated Framework for Implementation Research.

**Findings:** To date, eight CAB meetings have been held involving 12 stakeholders. The CAB continues to support the project via participant referrals and information dissemination. Five CHWs have enrolled as interventionists and completed required training. Each CHW has enrolled at least two families (N = 13), achieving 65% of our accrual goal in under five months. Key informant interviews and additional measures of implementation outcomes and factors are planned over the next six months.

**Implications for D&I Research:** A tenet of implementation research is stakeholder involvement. In this study, researchers engaged with stakeholders at multiple levels: through a CAB and via partnerships with local agencies. Without these partnerships, the ability to engage CHWs and families would have been severely limited. This example of multifaceted community partnerships can inform development of other implementation research in community settings.

**Primary Funding Source:** National Institutes of Health

### S131 Dissemination and implementation of the healthy eating and active living in the spirit (HEALS) intervention

#### Heather Brandt

##### Arnold School of Public Health, University of South Carolina, Columbia, SC, USA

###### **Correspondence:** Heather Brandt (hbrandt@sc.edu)

**Background:** Dissemination and Implementation of a Diet and Activity Community Trial In Churches (DIDACTIC) is a community-engaged project with African-American churches to implement a lay health educator (LHE)-delivered, evidence-based, diet and physical activity intervention. The intervention – Healthy Eating and Active Living in the Spirit (HEALS) – was developed with and for the African-American faith community and tested in a randomized controlled trial previously.

**Methods:** For the dissemination and implementation phase, a decentralized approach has been utilized in partnership with a community-based organization (CBO). The impetus for a decentralized approach was to build capacity for sustainability and institutionalization of the HEALS intervention during this phase. Additional strategies, such as a leadership development program and “hands-on” learning sessions to increase knowledge and skills, have been employed as implementation support strategies. The CBO – Faith-based African American Communities Empowered for Change (FACE) – has assumed the role of training mentors (LHEs who previously implemented the HEALS intervention) and LHEs, providing technical assistance to LHEs, tracking and monitoring implementation processes, and overseeing recruitment. University researchers provide guidance and support to FACE; however, this decentralized approach is led by FACE.

**Findings:** Preliminary results (i.e. intermediate outcome data) showed marginally positive changes in systolic blood pressure but no other observable improvements across myriad of behavioral and psychosocial variables. Preliminary intervention satisfaction data showed that 60% of participants reported the intervention to be very helpful, 69% were very satisfied with intervention delivery, and 95% would recommend the intervention to others. In terms of mentors and LHEs, we observed moderate retention of skills and knowledge and acceptable performance across assessment points. Church information was used to better understand the intervention environment. Observations were used to inform technical assistance activities.

**Implications for D&I Research:** Implementing a CBO-partners and LHE-delivered program establishes a pipeline for sustainability by increasing agency for delivery, and careful monitoring is needed. Results have led to changes to implementation and are used to enhance the dissemination of the intervention. Continued monitoring of the decentralized approach of delivery is essential to understanding how this type of approach may lead to sustainability and institutionalization of the intervention.

**Primary Funding Source:** National Institutes of Health

### S132 New England colorectal cancer screening learning collaborative for community health centers pilot

#### Lynn Basilio^1^, Randy Schwartz^2^

##### ^1^ New England Division, American Cancer Society, Framingham, MA, USA; ^2^ American Cancer Society, Atlanta, GA, USA

###### **Correspondence:** Randy Schwartz (randyschwartz09@gmail.com)

**Background:** In the United States, colorectal cancer (CRC) is the third most commonly diagnosed cancer and second leading cause of cancer deaths for men and women combined. Patients at increased risk for not undergoing CRC screening include individuals of lower socioeconomic status, uninsured and racial and ethnic minority groups. Oftentimes, these same individuals access medical care at Community Health Centers (CHCs). Nationally in 2012, CRC screening rates within CHCs was 30.2%. In New England, rates ranged from 32.3% (RI) to 52.6% (VT).

**Methods:** This pilot project was designed to increase CRC screening rates among participating CHCs and determine how the American Cancer Society can support quality improvement (QI) activities of CHCs to increase cancer screening and other activities.

**Findings:** The pilot was based on prior work by Taplin et al. and framed on the Institute for Health Care Improvement (IHI) Breakthrough Series Collaborative. The ACS Steps for Improving Colorectal Cancer Screening Rates: A Manual for Community Health Centers guided the evidence-based changes.

**Implications for D&I Research:** The learning collaborative model demonstrated how replication and scalability can be achieved, along with knowledge transfer contributing to sustainability.

**Primary Funding Source:** American Cancer Society

### S133 Implementing the youth empowerment solutions (YES) intervention: factors associated with fidelity

#### Andria Eisman^1^, Amy Kilbourne^1,2^, Alison Miller^1^, Peter Hutchinson^1^, Susan Morrel-Samuels^1^, Susan Franzen^1^, Laney Rupp^1^, Marc Zimmerman^1^

##### ^1^ University of Michigan School of Public Health, Ann Arbor, MI, USA; ^2^ QUERI, Department of Veterans Affairs, Ann Arbor, MI, USA

###### **Correspondence:** Andria Eisman (aeisman@umich.edu)

**Background:** Implementation of evidence-based programs (EBPs) with fidelity is critical to the effectiveness of youth development programs to support youths’ positive behaviors and reduce the risk of violence. Yet, youth rarely receive interventions as intended in community settings. Barriers to implementation fidelity for EBPs have not been widely studied for after-school youth development programs. This study examined factors related to implementation fidelity, including dose delivered and adherence to program protocol, guided by the Consolidated Framework for Implementation Research (CFIR), for an after-school program, Youth Empowerment Solutions (YES).

**Methods:** YES is an after-school youth development and violence prevention program focusing on participatory approaches to build skills, develop intergenerational partnerships and provide opportunities for participants to effect community change. The YES study was conducted in 12 middle and elementary schools in Flint, Michigan and surrounding Genesee County. The curriculum has 41 possible sessions, with 22 core program sessions. We collected process evaluation data on 33 YES groups from the schools over four years. We included intervention, provider, and context dimensions of CFIR as correlates to YES fidelity. We measured dose delivered using a sum score of the number of empowerment component areas completed during the program using school records. We measured adherence to program protocol using a sum score of the number of core program elements included using teacher-completed logs. We used linear regression models to investigate the relationship between implementation predictors and outcomes.

**Findings:** Among 28 groups who completed assessments (of the 33) 12% had a certified teacher facilitating the groups, 36% had a certified teacher as site coordinator, and 84% attended YES training. Having a certified teacher as site coordinator (contextual factor) was positively associated with dose delivered. YES teacher training, student engagement and having a certified teacher as facilitator did not predict dose delivered. We also found that have a certified site coordinator and YES training were positively associated with adherence.

**Implications for D&I Research:** We found that contextual (e.g., certified teacher as site coordinator), and intervention (e.g., program training) factors had notable implications on intervention fidelity. Schools incorporating administrative expertise and in-depth training may help ensure quality program delivery.

**Primary Funding Source:** National Institutes of Health

### S134 Assessing the effectiveness of training models for implementing health-promoting practices afterschool

#### Rebekka Lee^1,2^, Jessica Barrett^1^, James Daly^1^, Angie Cradock^1^, Catherine Giles^1^, Rebecca Mozaffarian^1^, Steven Gortmaker^1^

##### ^1^ Harvard T.H. Chan School of Public Health, Boston, MA, USA; ^2^ Harvard Clinical and Translational Science Center, Boston, MA, USA

###### **Correspondence:** Rebekka Lee (rlee@hsph.harvard.edu)

**Background:** Excess dietary intake and low physical activity contribute to childhood obesity. Out-of-school time programs serve 10.2 million U.S. children each year, and are important setting for early obesity prevention efforts. The Out-of-school Nutrition and Physical Activity (OSNAP) group-randomized trial demonstrated improvements in: children’s vigorous physical activity, the healthfulness of foods and beverages consumed, and health-promoting program policies. The goal of this study is to evaluate the effect of two training models to disseminate this intervention for broad population reach.

**Methods:** We used a 3-arm group-randomized trial to compare the effectiveness of in-person and online training models for scaling up the intervention with controls. One third of sites were randomized to the in-person train-the-trainer model: trained local YMCA facilitators conducted three learning collaborative meetings and technical assistance. One third of sites were assigned to the online model, consisting of self-paced monthly learning modules, training videos, interactive quizzes, and facilitated discussion board. Remaining sites served as controls. Fifty-one afterschool sites from three YMCA Associations in different regions of the country completed baseline and follow-up observations using a validated observation tool of afterschool nutrition and physical activity practices. We used multivariable regression models, accounting for clustering of observations, to assess intervention effects on an aggregate healthy practice primary outcome, and conducted secondary analyses of nine intervention goals (e.g. serving water, offering 30 minutes of physical activity). Cost data and an implementation survey aligned with the Consolidated Framework for Implementation Research were collected.

**Findings:** Changes in the primary outcome indicate that, on average, intervention sites achieved 0.44 additional OSNAP weekly goals compared to controls (p=0.02). The in-person arm achieved 0.51 additional goals (p=0.02), while the online arm showed positive trends but did not have significantly different effects from controls (+0.34, p=0.16). Achievement of intervention goals varied by training mode however, sites in both arms reported significant increases in fruits and vegetables served (p<0.05).

**Implications for D&I Research:** This study fills a critical gap between effectiveness research and scale up. We have established the effectiveness of two training models. We will discuss these outcomes in conjunction with cost and implementation data to identify “best buys” for future dissemination.

**Primary Funding Source:** National Institutes of Health

### S135 Increasing Australian schools’ implementation of a mandatory state-wide school healthy food policy: results of three randomised-controlled trials

#### Nicole Nathan^1,2,3^, Sze Lin Yoong^2^, Kathryn Reilly^4^, Rachel Sutherland^1^, John Wiggers^1^, Luke Wolfenden^2^

##### ^1^ Hunter New Engalnd Local Health District, Newcastle, Australia; ^2^ School of Medicine and Public Health, The University of Newcastle, Newcastle, Australia; ^3^ Hunter Medical Research Institute, Newcastle, Australia; ^4^ School of Medicine and Public Health, The University of Newcastle, Wallsend, Australia

###### **Correspondence:** Nicole Nathan (Nicole.Nathan@hnehealth.nsw.gov.au)

**Background:** Despite healthy school food policies being mandated by many jurisdictions in Australia and internationally for more than a decade, uptake has been limited. Without population wide implementation, the potential benefits of school policies will not be realised. Research investigating interventions to facilitate the implementation of health innovations, however, is limited. The aim of this paper is to assess the effectiveness of three randomised trials, of varying intensity, in supporting schools implementation of a healthy food policy mandated by the New South Wales (NSW) State Government.

**Methods:** Three randomised trials, with over 200 primary schools, were undertaken within the Hunter New England Region of NSW between February 2014 and June 2015. Implementation strategies varied across the three trials including such strategies as; executive support, training, resources, audit and feedback, communication strategies and ongoing support. The primary outcomes for the three trials were the proportion of schools with a canteen menu that did not contain foods or beverages (‘red’ and ‘banned’) restricted for sale under the policy; and the proportion of schools where healthy canteen items (‘green items’) represented more than 50% of listed menu items. Implementation of the policy was measured by menu audits at baseline and post-intervention (9-12 months following baseline) by dietitians, blinded to group allocation.

**Findings:** A dose-response relationship between implementation support and policy implementation was found. Results varied across the three trials from non-significant improvements for the primary trial outcomes to absolute improvements greater than 60%.

**Implications for D&I Research:** Increasing schools’ implementation of mandatory nutrition policies is possible however requires proactive implementation support strategies. The results provide different models of achieving policy implementation that could be considered by governments interested in maximising the impact of school nutrition polices.

**Primary Funding Source:** NSW Health, Australia

### S136 Implementation of a cross-sectoral approach yields improvements in youth and family behavioral health and reduces burden on justice and community service systems

#### Jennifer Rae, Suzanne Zerger

##### Centre for Addiction and Mental Health, Ottawa, ON, Canada

###### **Correspondence:** Jennifer Rae (Jennifer.Rae@camh.ca)

**Background:** A Regional Implementation Team worked with a service collaborative comprised of over 150 agencies to identify a systems-level gap in mental health and/or substance use services, select an evidence-based intervention, and refine and implement that intervention in five communities. The intervention, dubbed *Intersections*, connects youth who have had an initial contact with the police to services and supports to address issues contributing to their involvement with the justice system.

**Methods:** A mixed method study informed and refined the implementation process, including training assessments, interviews (with police, families, and other key stakeholders), case studies, and surveys. Performance measures have been imbedded to support decision- making and inform continuous quality improvement at clinical, organizational and regional levels. A community-designed, shared client database captures youths’ demographic characteristics, ratings on Child and Adolescent Needs and Strengths assessments, and referral dates, sources and wait times. Data are also captured on police contacts over time, and trends in emergency department use, to assess systems burden.

**Findings:** Between 2015 and 2017, officers from 22 police detachments in four large communities referred nearly 500 youth, ages 6-18, to give Intersections Workers (IW); just 8% declined services and the remainder continued to be engaged after 30 days of receiving services. IWs made 176 community referrals – matched to needs assessment results –most commonly to mental health services (44%) and education supports (18%); three-quarters of the youth had parents or caregivers engaged in the process, and IWs made 63 referrals on their behalf. Consistent themes in qualitative interviews indicate Intersections has engendered new collaborations across sectors and created new efficiencies in referral and treatment access for these youth. More than three-quarters (77%) of Intersections youth did not have further contact with police within three months after the first referral, and 71% did not have repeat contact after six months.

**Implications for D&I Research:** This study reveals implementation strategies key to the success diverse communities have employed to streamline access to care, improve behavioral health outcomes, and prevent youth justice involvement. Promising signs of sustainability – adherence to intervention fidelity, cross-sectoral key stakeholders remaining engaged – are supported and reinforced by community-designed data collection processes.

**Primary Funding Source:** Ministry of Health and Longterm Care

### S137 Systematic review of adaptations of public health evidence-based interventions

#### Cam Escoffery^1^, Patricia Dolan Mullen^2^, Erin Lebow-Skelley^1^, Regine Haardoerfer^1^, Elaine Böing^1^, Hallie Udelson^1^, Danielle Reece^1^, Richard Wood^2^, Marieke Hartman^3^, Shuting Liang^1^, Maria E. Fernandez^2^

##### ^1^ Rollins School of Public Health, Emory University, Atlanta, GA, USA; ^2^ University of Texas School of Public Health, Houston, TX, USA; ^3^ Academic Medical Center, Erasmus University College, Rotterdam, Netherlands

###### **Correspondence:** Cam Escoffery (cescoff@emory.edu)

**Background:** In the dissemination of evidence-based interventions (EBIs), little is known about how adaptation occurs. In using EBIs, there may be mismatches between the characteristics of the population, implementing agency, and/or community to the original program. Organizations sometimes adapt the EBI to correct mismatches. This systematic review uses Stirman and associates’ typology of modifications and common steps identified in a scoping study of adaptation frameworks to characterize adaptations in the field. The specific questions are: What are the reasons for the adaptation and common types of adaptations being made? What adaptation steps are used? What types of outcomes are assessed?

**Methods:** Articles were identified through searching PubMed, PsycINFO, PsycNET and CINAHL and included if they were: 1) published in English, 2) published after 1995, and 3) examined the adaptation process or outcomes of an adapted evidence-based, public health program/policy. Two researchers independently abstracted: 1) EBI characteristics, 2) reason for adaptation, 3) type of modifications, 4) adaptation steps, and 5) implementation/intervention outcomes.

**Findings:** We found 42 distinct programs, with 64% implemented in the U.S. Reasons for adaptation included the need for a culturally appropriate program (k=27; 64.3%), focus on a new target population (k=25; 59.5%), and the desire to implement the program in a different setting (k=24; 57.1%). Of 5 types (content, context, delivery, training and evaluation different types of adaptations, authors reported an average of 3.40 (SD=0.90)), with content being the most common. Among the adaptation process described, 37 (88.1%) conducted a community assessment, 31 (73.8%) determined the necessary changes based on assessments, 31 (73.8%) trained staff members, 30 (71.4%) consulted experts before adapting the materials, 37 (88.1%) prepared new materials, 35 (83.3%) implemented and 32 (76.2%) evaluated the adapted program. Evaluation outcomes included program implementation [acceptability (n=28; 66.7%), fidelity (k=22; 52.4%), feasibility (n=22; 52.4%), and changes in practice (n=9; 21.4%), behavior (k=30; 71.4%), and behavioral determinants (k=16, 38.1%)].

**Implications for D&I Research:** This study contributes to understanding adaptation in practice. We found inconsistences in the adaptation process, gaps in reporting of processes and changes made, and the need for capacity building. There is a necessity to elevate adaptation as a priority in D&I research.

**Primary Funding Source:** National Institutes of Health

### S138 Using implementation strategies that ensure engagement of public health organizations and other key stakeholders

#### Allison Dymnicki, Jason Katz, Xan Young, Mary Thorngren

##### American Institutes for Research, Washington, DC, USA

###### **Correspondence:** Allison Dymnicki (adymnicki@air.org)

**Background:** The American Institutes for Research is evaluating a five year initiative, funded by the Centers for Disease Control & Prevention (CDC), designed to improve the delivery and sustainability of evidence-based violence prevention efforts in local health departments (LHDs) and their community partners. Twelve LHDs from high risk communities have been selected to receive technical assistance and training (TTA) to develop their organizational capacity and the capacity of their community partners to implement youth violence (YV) prevention strategies. Outcomes for the initiative include increased visibility of YV as a public health approach, the formation of a multisector community coalition, and implementation of evidence-based strategies intended to prevent YV.

**Methods:** A mixed methods evaluation approach is being used to evaluate the TTA that is being provided, progress of the sites in achieving their short and long term outcomes, and reductions in behavioral health outcomes related to implementation of selected strategies. Data sources involve the evaluation of learning events, qualitative group interviews, quantitative surveys, and analysis of extant risk and protective factor and youth violence data. Information from these sources is reported back to the sites and CDC in continuous feedback loops designed to inform future TTA that is provided.

**Findings:** Three-year findings suggest that sites (in general) report having developed collaborative trusting relationship with TTA providers and that the majority of the learning objectives for in-person meetings and online learning events were met. Evidence suggests that several short-term outcomes have been accomplished including (1) expanding key partnerships and coalitions, (2) making progress towards selecting YVP programs, (3) reviewing strategic plans to more fully incorporate the public health approach, and (4) identifying additional resources for YVP.

**Implications for D&I Research:** Implementing multifaceted and multilevel prevention approaches designed to improve behavioral health outcomes among diverse and vulnerable populations requires the coordination and engagement of various sectors within a community. Few evaluations have assessed the effectiveness of TTA in supporting LHDs and their partners in doing this type of work. This evaluation suggests a range of approaches and types of supports that can help sites make progress in implementing implementation strategies that ensure engagement of LHDs and other key stakeholders.

**Primary Funding Source:** Centers for Disease Control and Prevention

### S139 Factors influencing the use of implementation tools to promote school health: a multi-level evaluation

#### Jennifer Leeman^1^, Jean Wiecha^2^, Jonathan Blitstein^3^, Maihan Vu^4^, Sallie Allgood^1^, Sarah Lee^6^

##### ^1^ School of Nursing, University of North Carolina at Chapel Hill, Chapel Hill, NC, USA; ^2^ RTI International, Boston, MA, USA; ^3^ RTI International, Durham, NC, USA; ^4^ Center for Health Promotion/Disease Prevention, University of North Carolina at Chapel Hill, Chapel Hill, NC, USA; ^6^ Centers for Disease Control and Prevention, Atlanta, GA, USA

###### **Correspondence:** Jennifer Leeman (jleeman@email.unc.edu)

**Background:** The Centers for Disease Control and Prevention (CDC) and others are developing tools to support implementation of evidence-based interventions (EBIs) in schools. These tools include electronic and print resources that provide guidance and support for assessing local contexts, selecting EBIs that fit, and adapting, implementing, evaluating, and sustaining EBIs. Little is known about how tools are used or factors that influence their use. To advance understanding of factors influencing the use of school health implementation tools, we conducted a national, multi-level evaluation of state-, school district-, and school-level use of four CDC tools designed to promote physical activity, nutrition, health education, and parent engagement.

**Methods:** The evaluation applied a mixed-methods, cross-sectional design that included surveys (n=69 from 43 states) and phone interviews (n=13 from 6 states) with state-level staff and phone and in-person interviews with district- and school-level staff (n=90 from 8 districts in 5 states). Descriptive analyses were applied to surveys and content analysis to interviews. Coding was guided by the Consolidated Framework for Implementation Research (CFIR).

**Findings:** The majority of those surveyed were aware of three CDC tools but were knowledgeable and confident in their ability to use only two. These same two tools were the ones most widely used and that states were most likely to have provided training and technical assistance on in the past year. Interviews provided insight into how tools were used and why use varied, with themes organized according to four CFIR constructs: (1) characteristics of tools, (2) inner setting, (3) outer setting, and 4) individual staff. Overall, tools were valued for the credibility of their source (CDC) and evidence strength and quality. Tools were too complex for use by school staff. However, if tools were adaptable and compatible with inner and outer setting factors, state and district-level staff were willing and able to translate tools for school use.

**Implications for D&I Research:** Implementation tools are essential to promoting broad-scale implementation of EBIs. This study illustrates how CFIR might be applied to evaluate factors influencing tools’ use and provides recommendations for designing tools that will fit within the multi-level systems involved in promoting, supporting, and implementing school health EBIs.

**Primary Funding Source:** Centers for Disease Control and Prevention

### S140 Randomized controlled trial of the getting to outcomes strategy's impact on implementation quality in community based settings

#### Matthew Chinman^1,2^, Joie Acosta^3^, Patricia Ebener^4^, Patrick Malone^5^, Jill Cannon^4^

##### ^1^ RAND Corporation, Pittsburgh, PA, USA;^2^ VA Pittsburgh Healthcare System, Pittsburgh, PA, USA;^3^ RAND Corporation, Arlington, VA, USA; ^4^ RAND Corporation, Santa Monica, CA, USA; ^5^ RAND Corporation, Durham, NC, USA

###### **Correspondence:** Matthew Chinman (chinman@rand.org)

**Background:** Effective implementation strategies that can be deployed at large scales are not widely available, but are sorely needed to facilitate evidence-based practices in low resourced community-based settings.

**Methods:** This presentation describes a Hybrid Type II, cluster-randomized controlled trial comparing two conditions: (1) 15 Boys & Girls Club (BGC) sites implementing an evidence-based, substance use prevention program called CHOICE for two years; (2) 15 similar BGC sites implementing CHOICE augmented with a two-year implementation strategy called Getting To Outcomes (CHOICE+GTO). All sites received training and manuals typical for CHOICE. GTO consists of its own manuals, training, and onsite technical assistance (TA). During the first year, TA providers helped CHOICE +GTO sites adopt, plan, and deliver CHOICE. Sites then received training on GTO’s evaluation and quality improvement steps, along with feedback reports summarizing their data, which were used in a TA-facilitated quality improvement process that yielded a revised plan for the second CHOICE implementation. The trial assessed whether GTO improves performance of key programming tasks (e.g., goal setting, planning, evaluation, quality improvement), CHOICE fidelity, and youth substance use outcomes (knowledge, attitudes, and behaviors around drug use). Performance was measured using ratings of a standardized, structured interview with participating staff at all 30 BGC sites after the first and second years of CHOICE implementation. Multiple elements of fidelity (adherence, classroom delivery) were assessed at all sites by observer ratings. Youth substance use outcomes were assessed via surveys before, and at 3 and 6 months after CHOICE.

**Findings:** By Year 2, CHOICE+GTO sites had significantly higher ratings of performance, classroom delivery, and adherence (e.g., 88% vs. 65% CHOICE activities fully implemented). Drug use was very low and thus did not differ between groups in either year. However in Year 2, youth in CHOICE+GTO sites showed improvement in drug knowledge and attitudes outcomes. CHOICE only youth showed no change.

**Implications for D&I Research:** This study is one of the first that assesses an implementation strategy’s impact on performance, implementation quality, and individual outcomes simultaneously and similarly in both study conditions. The findings suggest that GTO’s implementation support can help community-based settings achieve high levels of fidelity and outcomes.

**Primary Funding Source:** National Institutes of Health

### S141 The community preventive services task force: using systematic reviews to provide evidence-based recommendations and findings for public health interventions

#### Jamila Jones, Shawna Mercer

##### Centers for Disease Control and Prevention, Atlanta, GA, USA

###### **Correspondence:** Shawna Mercer (smercer@cdc.gov)

**Background:** The Guide to Community Preventive Services (The Community Guide) is a website that houses all the evidence-based findings and recommendations of the Community Preventive Services Task Force (CPSTF) as well as tools and resources to assist in dissemination, implementation, and evaluation of evidence-based practices. Dr. Jamila Jones, who serves as the Dissemination and Implementation Team Lead in the Community Guide Branch, will serve as the panel’s moderator and provide an overview of CPSTF and The Community Guide. She will explain the categories of CPSTF findings and explore how the Community Guide Branch at CDC works with external partners such as NIH.

**Methods:** The Community Guide conducts systematic reviews of interventions in many topic areas to learn what works to promote public health. CPSTF uses the results of these reviews to issue evidence-based recommendations and findings to the public health community. Community Guide reviews use methods that address the specific needs of public health. Methods are developed internally, approved by the CPSTF, and published in peer-reviewed journals.

**Findings:** Categories of Task Force Recommendations and Findings: Recommended, Recommended Against, and Insufficient Evidence. Each CPSTF finding is 1) based on the strength of the evidence of effectiveness in changing outcomes; 2) found through systematic reviews of published literature; 3) conducted by a team of experts on behalf of the CPSTF; and 4) meant to be used along with information about local needs, goals, and constraints.

**Implications for D&I Research:** Decision-makers in communities, health departments, nonprofits, government, businesses, and healthcare systems rely on CPSTF recommendations about effective public health interventions to better protect and improve population health. Information from The Community Guide is used to design national initiatives, prioritize effective programs, and inform research agendas and evaluation efforts.

**Primary Funding Source:** National Institutes of Health

### S142 Increasing knowledge utilization of preventive services and addressing research gaps

#### Jennifer Villani, Elizabeth Neilson

##### Office of Disease Prevention, National Institutes of Health, Rockville, MD, USA

###### **Correspondence:** Jennifer Villani (villani@nih.gov)

**Background:** The Community Preventive Services Task Force (CPSTF) is a panel of public health and prevention experts that provides evidence-based findings and recommendations about preventive services, programs, and policies to improve public health in community settings. The evidence reports and recommendations of the CPSTF are informed by research funded through the National Institutes of Health (NIH). In addition, the NIH Office of Disease Prevention (ODP) supports the CPSTF by identifying content experts, assessing activity to address research gaps, and disseminating findings, recommendations, and research needs to key stakeholders.

**Methods:** Through rigorous processes of reviewing evidence on preventive services topics, the CPSTF identifies needs in prevention research. They also issue “insufficient evidence”, or IE findings, which highlight gaps in the evidence base that prevent them from recommending for or against certain preventive services. The ODP disseminates CPSTF recommendations and findings through a variety of communication channels, helps assess awareness of research gaps within the context of NIH Institutes, Centers, and Offices, and identifies the barriers and facilitators to addressing research needs.

**Findings:** Since its inception in 1996, the CPSTF has issued 90 IE findings. The CPSTF has found that understanding of insufficient evidence statements/findings among clinicians, researchers, and policy-makers is limited. The NIH is collaborating with the federal agency that supports the CPSTF (i.e., the Centers for Disease Control and Prevention [CDC]) to disseminate findings and address research gaps.

**Implications for D&I Research:** The NIH and CDC are working to increase knowledge utilization of preventive services in community settings and to address knowledge gaps through targeted research.

**Primary Funding Source:** National Institutes of Health

### S143 Evaluating the use of community preventive services task force insufficient evidence findings within NIH

#### Anita Ellis (aqa5@cdc.gov)

##### Centers for Disease Control and Prevention, Atlanta, GA, USA

**Background:** The Community Preventive Services Task Force (CPSTF) has issued multiple Insufficient Evidence (IE) findings based on rigorous systematic reviews, and data show that these findings are often misunderstood and misinterpreted by researchers and other important users. The Office of Disease Prevention at NIH and the Community Guide Branch at CDC partnered to evaluate (1) the awareness and use of CPSTF IE findings among researchers and grant funders at NIH and (2) two IE Findings User Guides that were developed to provide information on how to use IE findings in the research development process.

**Methods:** The project team adopted CDC’s Six Step Evaluation Framework to develop the project’s design, purpose, targeted audiences, recruitment process, and the development of two IE finding User Guides (resources). Separate interview guides were created for both Researchers and Funders. Interview questions were drafted in August 2016 and tested internally at CDC through the cognitive interviewing process. Both interview guides were piloted with four NIH employees in October, and after team discussion, they were finalized with minor edits. The remaining interviews were conducted in early 2017. All interviews were recorded with permission from respondents, and the project interviewer entered notes and feedback directly into the interview guides as interviews took place. Interview data was de-identified and reviewed by two team members, who independently reviewed the notes and recordings of each interview, completed rating criteria for evaluation questions, and reconciled any differences. The team used Microsoft Excel and NVivo 11 for data analysis.

**Findings:** Overall, forty-two telephone interviews were conducted with grant funders, intramural researchers, and extramural researchers from 13 different Institutes, Centers, and Offices across NIH.

**Implications for D&I Research:** Findings from this project influenced decisions made regarding increasing awareness of The Community Guide and CPSTF among NIH ICOs and improving use of insufficient evidence findings among researchers and those who write funding opportunity announcements.

**Primary Funding Source:** National Institutes of Health

### S144 Findings from a collaborative evaluation project between NIH and the community guide

#### Megan Cotter^1,2^ (leq6@cdc.gov)

##### ^1^ Rollins School of Public Health, Emory Prevention Research Center, Atlanta, GA, USA; ^2^ Centers for Disease Control and Prevention, Atlanta, GA, USA

**Background:** When evidence gaps exist in the literature and there is insufficient evidence (IE) to determine whether an intervention works, the Community Preventive Services Task Force (CPSTF) suggests more research be done to assess effectiveness. Evaluators in the Community Guide Branch embarked on a project to explore how researchers and those who fund research at NIH use IE findings.

**Methods:** From October 2016 to April 2017, project evaluators conducted forty-two telephone interviews with grant funders, intramural researchers, and extramural researchers from 13 different Institutes, Centers, and Offices across NIH. Respondents were asked about their familiarity and prior use of CPSTF findings, resources from The Community Guide, and IE findings. They were also asked to provide feedback on a two-page IE Findings User Guide that had been drafted by staff in the Community Guide Branch. Project evaluators systematically analyzed responses to answer project evaluation questions.

**Findings:** Results showed that the majority of participants were not familiar with IE findings from the CPSTF and did not use insufficient evidence findings/evidence gaps as part of their research development process for various reasons. Participants were able to comment on the overall quality of the IE Findings User Guides, and the majority recommended making specific revisions before disseminating the User Guides. Overall, feedback indicated that the User Guides served as helpful reminders and reinforced important concepts about IE findings. Finally, project participants offered suggestions for how to improve collaboration and dissemination efforts with grant funding organizations like NIH.

**Implications for D&I Research:** IE findings and evidence gaps identified by CPSTF can and should play an important role in the research development process for public health research and funding organizations such as CDC and NIH. Findings from this project are already being used to strategize future collaboration efforts with partners like the NIH, to revise existing communication materials like the IE Findings User Guides, and to develop new dissemination materials aimed at research and funding organizations.

**Primary Funding Source:** National Institutes of Health

## Promoting Health Equity and Eliminating Disparities

### S145 The comprehensive care, community, and culture program (C4P): early lessons from C4P implementation

#### David Meltzer, Elizabeth Nida

##### University of Chicago, Chicago, IL, USA

###### **Correspondence:** David Meltzer (dmeltzer@medicine.bsd.uchicago.edu)

**Background:** Improving health requires interventions far beyond traditional medical care. The Comprehensive Care, Community, and Culture Program (C4P) seeks to address the complex medical and social needs of patients at increased risk of hospitalization, a group that accounts for most US health care spending and poor health outcomes and is disproportionately disadvantaged socioeconomically. C4P builds on the Comprehensive Care Program (CCP), which provides patients with inpatient and outpatient care from a physician with whom they have an established relationship and has produced promising evidence of improved patient experience and outcomes, and lower costs. Nevertheless, 30% of patients offered CCP care do not engage despite systematic efforts to reach them. C4P adds screening of unmet social needs, access to Community Health Workers and a community, arts and cultural program. As part of implementing C4P we seek to first understand unmet needs in this population.

**Methods:** Using a tool adapted from Health Leads, we have screened 227 patients for 17 unmet social need categories.

**Findings:** Early findings are: 1) Large variability in the prevalence of specific social needs, ranging from 3%-56% of patients. The most prevalent are: healthy eating/physical activity, engaging in enjoyable activities, health/dental insurance, transportation, and money for basic needs. 2) Needs are highly concentrated in a subset of patients. 3) Many needs co-occur, suggesting opportunities to intervene simultaneously, e.g. “healthy eating”/“food”, “companionship”/“engagement”, “insurance”/“employment.” 4) Unmet needs often persist, but new needs constitute a substantial fraction of unmet needs at any given time. 5) Patients with more unmet needs are less likely to resolve existing needs and more likely to develop new unmet needs. Qualitative interviews help us further understand patients’ needs within these categories and the dynamics of unmet needs over time, and highlight depression and social isolation as key factors.

**Implications for D&I Research:** Early findings suggest that needs are multifaceted and dynamic and that mental health issues create cross-cutting barriers to receiving services. Combining need-oriented and patient-specific interventions, especially psychosocial support and strong patient-provider relationships, has promise to holistically address complex patients’ unmet social needs, which has the potential to reduce healthcare utilization and improve patients’ health and experience.

**Primary Funding Source:** The Robert Wood Johnson Foundation

### S146 Addressing behavioral health in TANF to improve health equity among low-income caregivers

#### Mariana Chilton^1^, Jerome Dugan^2^, Sandra Bloom^1^, Layla Booshehri^2^, Falguni Patel^3^

##### ^1^ School of Public Health, Drexel University, Philadelphia, PA, USA; ^2^ College of Nursing and Health Professions, Drexel University, Philadelphia, PA, USA; ^3^ Center for Hunger-Free Communities, Drexel University, Philadelphia, PA, USA

###### **Correspondence:** Falguni Patel (fp76@drexel.edu)

**Background:** Caregivers of young children in Temporary Assistance for Needy Families (TANF) are required to complete 20hrs/week of work participation, and are hurried into employment, often without consideration for their health and their children’s wellbeing. For instance, TANF caregivers report high rates of work limiting health conditions, as well as Adverse Childhood Experiences (ACEs) which can transfer to their children through severe hardship, depression, and trauma-related behavior. Additionally, the racial and ethnic wealth gap among women in the United States continues to persist, where women of color have few to no assets, and little to no savings to help their families weather economic shocks, which in turn puts their health at greater risk.

**Methods:** This implementation study builds on the Building Wealth and Health Network (The Network) randomized controlled trial (RCT) that substituted standard TANF programming with trauma-informed peer support, financial education, and matched-savings accounts. The RCT showed significant reductions in depressive symptoms and hardship related to food, housing and utilities. In the Phase II implementation of The Network we revised the curriculum to be implemented over a shorter timeframe, which includes 16-weeks of trauma-informed financial empowerment and 12 months of matched-savings. We are carrying out rapid-cycle evaluation at baseline, and every three months over 12 months with Audio Computer-Assisted Self-Interview (ACASI) software to track economic hardship, behavioral health, and labor market outcomes.

**Findings:** At baseline, among the 225 members recruited thus far, 32.6% of participants have some college, 16.4% are employed, 52% are food insecure, and 25.5% report ≥4 ACES. Rapid-cycle evaluation results with 97 participants that completed Phase II reveal improvements in food security (47.4% to 67.9% at 12mo, *p*=0.006); and reductions in depressive symptoms for caregivers reporting ≥4 ACEs (79.2% to 46.2% at 12mo, *p*=0.04) and a significant increase in employment (18.1% to 54.7% at 12mo, *p* <0.0001). We are also examining associated cost savings to Medicaid behavioral health and TANF program expenditures.

**Implications for D&I Research:** Policy implications from our results identify the importance of linking Medicaid with TANF to promote a culture of health within TANF, and to improve health equity.

**Primary Funding Source:** The Robert Wood Johnson Foundation

### S147 Community participatory research to enhance multisector collaboration, accelerate alignment, and reduce policy cycle time to advance the culture of health

#### William Riley^1^, George Runger^2^, Gevork Hartoonian^2^, Michael Shafer^3^, Kailey Love^1^

##### ^1^ School for the Science of Health Care Delivery, Arizona State University, Phoenix, AZ, USA; ^2^ Center for Health Information Research, Arizona State University, Phoenix, AZ, USA; ^3^ University of Arizona, Tucson, AZ, USA

###### **Correspondence:** William Riley (William.J.Riley@asu.edu)

**Background:** In 2016, the Robert Wood Johnson Foundation launched the Systems for Action Research Program to test new mechanisms for aligning medical, social, and public health delivery systems to improve health and health equity. The Arizona State University received funding as one of the Systems for Action Research Centers and the focus of this project has been on health care, criminal justice, and social services coordination of individuals with mental illness and/or substance use disorders.

**Methods:** This presentation describes the ASU project and presents preliminary findings. Medicaid claims data for a large urban community in the southwest US was analyzed for a population of approximately 350,000 claimants. Using multisector databases including hospital discharges, Medicaid claims, jail bookings, correctional health services, probation supervision, homeless management information system, law enforcement, the court system, and social service agencies, we simulate a future state of better alignment to create a coordinated delivery system for behavioral health disorders with an improved impact on health, well-being and equity for the population. We use these data to compare patterns of care for physical health and severely mentally ill (SMI) patients as well as understand their respective determinants. We next apply these data to a multisector analysis to better understand the sequential intercept model to better align care processes and outcomes. To date, a multisector group of stakeholders, representing more than sixty individuals and organizations within Phoenix has been convened. Preliminary analysis has involved investigating how multisector services, delivery systems, and financing streams are currently aligned as well as their gaps.

**Findings:** Our findings provide a multisector analysis that follows patients across various treatment settings, law enforcement interventions, through the court system and final resolution including probation, incarceration, or discharge to the community. We also present distributions and frequencies associated with the sequential intercept at various stages in the process.

**Implications for D&I Research:** Very little research has been undertaken to understand multisector patterns and care utilization that includes healthcare, law enforcement, social service agencies, public health, and the court system. This is among the first research studies to assemble and match multisector cost and utilization.

**Primary Funding Source:** The Robert Wood Johnson Foundation

### S148 Reducing rural health disparities by improving access to evidence-based depression care for low-income primary care patients

#### Diane Powers, Jurgen Unutzer

##### University of Washington, Seattle, WA, USA

###### **Correspondence:** Diane Powers (powersd@uw.edu)

**Background:** Depression is the second leading cause of disability in the U.S. (after heart disease) and is associated with higher healthcare costs, reduced productivity, and lower incomes. This is exacerbated by limited access to providers and evidence-based treatments in rural areas. Rural residents are significantly more likely to live in poverty with limited access to resources like education, jobs, transportation, and broadband internet. Rural healthcare clinics face similar gaps in resources and capacity, making implementation of evidence-based practices more difficult. Unsurprisingly, states with the lowest population per square mile (Alaska, Wyoming, Montana) report the highest suicide rates.

**Methods:** Eight rural federally qualified health centers (FQHCs) in Alaska, Washington, Wyoming and Montana participated in a grant-funded initiative to implement Collaborative Care for depression. Collaborative Care applies chronic disease management principles (e.g. measurement-based, treatment-to-target) to depression treatment delivered in primary care, increasing access and reducing stigma. The AIMS Center provided training and implementation coaching for the initiative, tailoring this assistance in response to the unique challenges faced by rural primary care clinics and informed by experience supporting implementation in over 1,000 primary care clinics. Participating clinics used an online care management registry designed to facilitate delivery of care. Data from this registry was analyzed to determine depression outcomes and use of evidence-based processes of care among participating clinics.

**Findings:** Over 5,000 adults were engaged in depression treatment. Two thirds of patients were women and one third men, which aligns with existing data that women are twice as likely to be diagnosed with depression as men. Most patients are White (83%) with the second largest racial category American Indian/Alaska Native (8%). Mean PHQ-9 depression score at baseline was 16.0 (moderately severe) and 10.9 (mild) at last recorded visit. Treatment response was achieved by 42% of patients and treatment remission by 22%, which compares favorably to urban FQHCs.

**Implications for D&I Research:** Recent articles document barriers experienced by rural settings implementing evidence-based interventions and the detrimental effect this exerts on spread, exacerbating health disparities experienced by rural residents. This initiative proves effective implementation is possible when training and support is tailored to the unique needs of rural communities.

**Primary Funding Source:** Corporation for National and Community Service (Social Innovation Fund), The John A. Hartford Foundation, Margaret A. Cargill Foundation, The Helmsley Charitable Trust, Rasmussen Foundation, Lewis County Board of Commissioners

### S149 Collaborative care for depression: treatment outcomes for rural American Indians/Alaska natives as compared with non-indigenous patients

#### Deborah Bowen, Theresa Hoeft

##### University of Washington, Seattle, WA, USA

###### **Correspondence:** Deborah Bowen (dbowen@uw.edu)

**Background:** American Indians and Alaska Natives (AI/AN) experience significant health disparities in access to evidence-based depression care. Collaborative Care is an evidence-based approach that applies chronic disease management principles to depression and other common mental health disorders treated in primary care. Viewed through a social justice lens, Collaborative Care combats health inequities by improving both access to care and quality of care, a result demonstrated in more than 80 randomized controlled trials across a wide variety of clinical settings and diverse patient populations. Despite the extensive evidence base for Collaborative Care, none of the trials to date enrolled a sufficiently large cohort of American Indians and Alaska Natives (AI/AN) to permit comparison of treatment outcomes between indigenous and non-indigenous patients. This study compares depression outcomes for AI/AN patients receiving Collaborative Care at rural primary care clinics to non-indigenous patients also receiving Collaborative Care.

**Methods:** Eight primary care clinics in four states (Alaska, Washington, Montana, Wyoming) were recruited to participate in a Collaborative Care dissemination project focused on low-income patients. All of the clinics were Federally Qualified Health Centers (FQHCs) located in areas designated as medically underserved and/or healthcare provider shortage areas for primary care and/or behavioral health. No significant cultural adaptation was made to Collaborative Care at any clinic. Of a total of 5140 enrolled patients at 8 clinics, 688 were AI/AN and 3863 were Caucasian.

**Findings:** Average PHQ-9 scores at baseline did not differ significantly between AI/AN and Caucasian patients (37.1 versus 36.1 respectively) At the last treatment follow-up a slightly higher proportion of AI/AN patients had significant improvements in PHQ-9 scores, compared to Caucasian patients (61.1% vs 80.9%, respectively) Caucasian patients reported slightly fewer contact days and in-clinic visits during the treatment period compared with AI/AN patients. We will also examine the association of demographic, clinical characteristics and treatment indices with improvement in depression in these two groups.

**Implications for D&I Research:** This study provides significant evidence for the effectiveness of Collaborative Care for American Indians/Alaska Native people. It also provide a means of reducing a considerable health disparity for this population.

**Primary Funding Source:** Corporation for National and Community Service (Social Innovation Fund), The John A. Hartford Foundation, Margaret A. Cargill Foundation, The Helmsley Charitable Trust, Rasmussen Foundation, Lewis County Board of Commissioners

### S150 Facilitating sustainable collaborative care programs in rural settings using the stages of implementation completion (SIC)

#### Lisa Saldana, Jason Chapman, Holle Schaper, Mark Campbell

##### Oregon Social Learning Center, Eugene, OR, USA

###### **Correspondence:** Lisa Saldana (lisas@oslc.org)

**Background:** Tools to monitor implementation progress are needed to facilitate scale-up efforts. The Stages of Implementation Completion, an 8-staged measure of implementation process and milestones, was adapted for Collaborative Care (CC-SIC) to aid in increasing scale-up of sustainable Collaborative Care (CC) in rural settings. The CC-SIC was piloted in five primary care clinics serving low-income, adult populations in the rural West.

**Methods:** Using a well-established SIC adaptation process, the AIMS Center collaborated with the SIC team to operationalize the CC implementation process. Four missing data types were defined including activity: (1) not necessary because completed in a previous implementation; (2) not applicable to site; (3) completed but the date of completion is unknown; and (4) truly not completed. Implementation activities were observed for completion; dates on which the activities were completed were recorded and entered into the SIC website by an AIMS Center staff. Using a standardized SIC scoring protocol, Duration and Proportion scores were calculated for pre-implementation and implementation stages.

**Findings:** One site discontinued and the other four are ongoing. The discontinued site launched and entered Stage 8, but discontinued prior to achieving competency for sustainability. SIC data and purveyor report suggested this site did not engage in the implementation process to the same degree as other sites. Although the site reported their decision was based on lack of funding, this was not an impediment to similar clinics. On average, sites that achieved sustainment completed a high proportion of implementation activities in both pre-implementation and implementation phases (100% and 93%, respectively) and spent 250 days on pre-implementation activities. All four missing data designations were used; the discontinued site had a higher number of implementation activities truly not completed than the other sites. It also was the only program to never achieve the recommended caseload size per provider or develop an economic sustainability plan.

**Implications for D&I Research:** Observation of implementation behavior and assessment of missing steps in the process allowed for improved monitoring by purveyors and better understanding of activities necessary for sustainment. The CC-SIC accurately assessed implementation effectiveness and detected site variations suggesting the potential to aid future scale-ups of CC for diverse populations.

**Primary Funding Source:** National Institute of Mental Health (R01-MH097748), Corporation for National and Community Service (Social Innovation Fund), The John A. Hartford Foundation, Margaret A. Cargill Foundation, The Helmsley Charitable Trust, Rasmussen Foundation, Lewis County Board of Commissioners

### S151 A protocol for developing and testing diabetes self-management support (DSMS) strategies in African American churches: a cluster randomized hybrid type II trial

#### Gretchen Piatt^1^, Nik Koscielniak^1^, Martha Funnell^1^, Robin Nwankwo^1^, Diana Hall^1^, Michele Heisler^1,2^

##### ^1^ University of Michigan, Ann Arbor, MI, USA; ^2^ VA Ann Arbor Healthcare System, Ann Arbor, MI, USA

###### **Correspondence:** Gretchen Piatt (piattg@umich.edu)

**Background:** Evidence demonstrates the effectiveness of diabetes self-management education (DSME) in the short-term for improving clinical, psychosocial, healthcare utilization, and cost outcomes. However, a significant gap exists between current DSME practices and the infrastructure needed to foster sustainability of improved outcomes. Low-resource communities are often served by health care systems that lack resources and personnel for providing long-term diabetes self-management support (DSMS). This study protocol describes a four-phase approach to developing and testing implementation strategies to support DSMS uptake and sustainability of improved outcomes in 21 African American churches in three metropolitan areas.

**Methods:** Phase 1 was informed by the Consolidated Framework for Implementation Research and the Theoretical Domains framework. A stakeholder-driven approach, using focus groups and interviews, was used to apply information on barriers and facilitators to DSMS uptake in churches. This information informed the design of three DSMS implementation strategies. A 33-month, Hybrid Type II cluster randomized trial was then deployed to compare the strategies to each other and to usual care in Phase 2. Twenty-one churches were randomized to one of three strategies with 6-9 churches per group (21-23 individuals with diabetes, one parish nurse (PN), and two peer leaders (PL) per church). Phase 3 will include post-trial qualitative data collection to understand participant experiences with the strategies. Phase 4 will include a series of cost effectiveness analyses and simulation modeling to determine the long-term societal impact of the trial.

**Findings:** Twenty-one PNs and 28 PL were/are being trained, with a 30-hour curriculum, to facilitate DSMS at each church. 12/21 churches are actively implementing one of the three strategies. The three DSMS strategies are being compared to each other and to usual care on outcomes specified by the RE-AIM model (e.g. reach to churches, effectiveness of DSMS on diabetes indicators, adoption commitment of the church, implementation fidelity and acceptability, and maintenance of improved outcomes at 21 and 33 months.

**Implications for D&I Research:** Findings from this study will facilitate understanding of barriers/facilitators to implementing DSMS, how to design effective DSMS approaches, and the long-term impact of providing support. Further, this study demonstrates a systematic process to develop and test a tailored implementation strategy.

**Primary Funding Source:** National Institutes of Health

### S152 Developing strategies to adapt social skills training for supported housing participants

#### Sonya Gabrielian^1,2,3^, Alison Hamilton^1^, Alexander Young^1,2,4^

##### ^1^ VA Greater Los Angeles Healthcare System, Los Angeles, CA, USA; ^2^ University of California, Los Angeles, Los Angeles, CA, USA; ^3^ Implementation Research Institute, Washington University at St Louis, St Louis, MO, USA; ^4^ Desert Pacific Mental Illness Research, Education, and Clinical Center, Department of Veterans Affairs, Los Angeles, CA, USA

###### **Correspondence:** Sonya Gabrielian (sonya.gabrielian@va.gov)

**Background:** Permanent, community-based housing with supportive services (“permanent supported housing (PSH)”) improves housing and health for homeless-experienced persons. Unfortunately, some PSH participants prematurely lose their housing and become homeless. Social skills are a determinant of premature PSH exits. Though social skills training (SST) effectively improves participants’ social skills and functioning, these interventions are uncommon within homeless services. To improve PSH participants’ housing retention, we conducted a developmental formative evaluation (FE) to identify strategies to adapt effective SST interventions to fit PSH’s contextual needs.

**Methods:** Data collection reflected developmental FE’s key elements. To identify factors relevant to SST outcomes in psychiatric rehabilitation, we performed key informant interviews with national SST leaders (n=12). Key informants also identified potential barriers to and facilitators of SST implementation in PSH; discussed viable implementation strategies; and characterized perceptions of the feasibility and utility of SST in PSH. We also conducted semi-structured interviews with PSH participants (n=40) to glean their perspectives on the utility of SST in PSH. We performed thematic analyses of these data. We plan to convene an expert panel and focus groups with PSH participants and providers to gather information on determinants of current practice and implementation feasibility.

**Findings:** Key informants highlighted the importance of tailoring SST’s behavioral instruction (e.g., role plays) to participants’ real-life challenges in PSH. Implementation barriers included participants’ competing needs (e.g., food) and PSH’s lack of treatment mandates. Implementation facilitators included clinician competence and established fidelity scales. Implementation feasibility was highlighted by informants’ prior successes tailoring SST for other complex settings. Both participants and informants perceived SST as highly valuable within PSH; participants perceived particular value in certain SST domains, e.g., conversation skills. Engaging leaders and providers from the implementation site in the planned expert panel and focus groups will enhance stakeholder commitment and feasibility.

**Implications for D&I Research:** Through qualitative interviews with key informants and PSH participants, this FE highlights strategies to adapt SST for PSH’s contextual needs. In a hybrid type I trial, employing an implementation strategy guided by this FE, we will study the adapted intervention’s effectiveness on housing retention, while gathering data about barriers to and facilitators of implementation in routine PSH care.

**Primary Funding Source:** Department of Veterans Affairs

### S153 Implementation of health equity interventions: influence of external organizational context

#### Elizabeth Durkin, Kathryn Gunter, Robert Nocon, Jenna Jablonski, Scott Cook, Marshall Chin

##### University of Chicago, Chicago, IL, USA

###### **Correspondence:** Elizabeth Durkin (edurkin@medicine.bsd.uchicago.edu)

**Background:** *The Roadmap to Reduce Disparities* is a framework for reducing health disparities that recommends providers implement strategies at levels internal to the organization (patient, provider, microsystem, organizational) as well as external (community and policy). Using two organizational theories- -resource dependence and institutional theory -- we analyzed data from three sites that combined delivery system with payment reform to reduce disparities in order to elucidate the impact of healthcare organizations’ external context (aka “outer setting”) on implementation.

**Methods:** We conducted 38 semi-structured interviews at three grantee sites of *Finding Answers*, an RWJF-funded disparities-reduction initiative. Reviewers developed a codebook iteratively and analyzed transcripts with a modified template approach, supplemented with review of annual reports and meeting notes.

**Findings:** All three grantee sites depended on outside actors for resources and institutional legitimacy, but two of the sites primarily utilized internal strategies (i.e., creating or bolstering team processes, enhancing use of EHR; more closely integrating social and behavioral health services). The third intervention was significantly more complex vis a vis the need to engage with external actors: service delivery shifted from clinics to community-based partners; mid-level practitioners expanded their scope of practice; care teams connected “virtually” vs. being physically co-located; and care innovations necessitating state regulatory changes were introduced. The practice changes threatened professional ideologies and disrupted partner and competitor relationships. The resulting community resistance contributed to initial implementation delays and ongoing challenges to program uptake, and spurred attempts to leverage political capital. These delays impeded team members from meeting new pay-for-performance targets, deflating their motivation to earn incentives. Unlike the two other sites, implementation of this intervention was highly dependent on actors outside the organization, yet the need to strategically account for those challenges was unrecognized during the design phase.

**Implications for D&I Research:** Implementation of delivery system and payment reform interventions to reduce disparities should utilize strategies at the community and policy levels when warranted by the complexity in the external organizational context (i.e., when dependence on external actors for resources and legitimacy are greater). Payment reform designs should be flexible enough to vary across organizations depending on their need to engage with external actors for successful implementation.

**Primary Funding Source:** The Robert Wood Johnson Foundation

### S154 Tailoring implementation of VA’s patient-centered medical home to the needs of women veterans using evidence-based quality improvement

#### Elizabeth Yano^1,2^, Alison Hamilton^1,2^, Ismelda Canelo^1^, Britney Chow^1^, Emmeline Chuang^2^, Julian Brunner^1,2^, Lisa Rubenstein^1,2,3^

##### ^1^ VA Greater Los Angeles Healthcare System, Los Angeles, CA, USA; ^2^ UCLA Fielding School of Public Health, Los Angeles, CA, USA; ^3^ RAND Corporation, Santa Monica, CA, USA

###### **Correspondence:** Alison Hamilton (alisonh@ucla.edu)

**Background:** VA policy guidance for patient-centered medical home (PCMH) implementation was not originally adapted for special populations, including women Veterans (WVs), whose numerical minority, comorbid physical and mental health (MH) burdens, and need for gender-specific care complicates primary care (PC) delivery. We tested an evidence-based quality improvement (EBQI) implementation strategy for tailoring PCMH to meet WVs’ needs.

**Methods:** We tested EBQI in a cluster randomized trial across 12 medical centers, using an unbalanced 2:1 allocation to accommodate anticipated variations in EBQI implementation (8E:4C). EBQI included multilevel regional stakeholder panel meetings for consensus on QI priorities, EBQI team training, external practice facilitation, formative feedback, and across-site collaboration calls. Feedback reports were drawn from baseline patient surveys, provider/staff surveys, key stakeholder interviews, PC teamlet interviews, and quality metrics by gender.

**Findings:** EBQI VAMCs completed 1-3 QI projects each over 24-months, including improved follow-up of abnormal breast cancer screening (27% increase in documentation, 6-day average decline in follow-up), follow-up of abnormal cervical cancer screening (<50% to 85% received correct recommendations), testing/reporting cervical cytology (72% to 96% compliance), assignment of new patients to designated WH providers (75% to 100%), visit comprehensiveness (0% to 80% obtaining labs before first appointment), PCMH team functioning (increased team climate scores and quality metrics, reduced fractured days and burnout), residents’ trauma-sensitive communication with WV patients (increased knowledge, communication, satisfaction scores), and proactive identification of WVs in MH crisis/distress (pre-visit MH handoffs; improved patient, provider, staff satisfaction). Multiple QI projects are being spread regionally.

**Implications for D&I Research:** Sites engaged in EBQI made substantial gains in a wide range of QI targets aligned with regional priorities and adapted to local contexts, with the support of research-delivered technical support and formative data but otherwise without additional direct funding. This partnered research demonstrates early EBQI impacts fostering employee engagement and implementation and diffusion of promising practices. Given its effectiveness, we are now supporting and evaluating spread of this evidence-based implementation strategy.

**Primary Funding Source:** Department of Veterans Affairs

## Poster Slam

### S155 Experiences of primary care physicians and staff after lean workflow redesign

#### Dorothy Y. Hung^1^, Michael I. Harrison^2^, Quan Truong^1^, Xue Du^1^

##### ^1^ Palo Alto Medical Foundation Research Institute, Mountain View, CA, USA; ^2^ Agency for Healthcare Research and Quality, Rockville, MD, USA

###### **Correspondence:** Michael Harrison (Michael.Harrison@ahrq.hhs.gov)

**Background:** Learning healthcare systems are implementing LEAN process redesign to improve efficiency and quality, but little is known about workforce responses to such initiatives. We examine physician and staff experiences following Lean-based redesign of workflows among primary care teams. Changes included: standardization of equipment and patient education materials in all exam rooms, streamlining of call center functions, co-location of physicians and medical assistants in a shared workspace, and creation of new workflows for all care teams.

**Methods:** Lean redesigns were implemented and scaled across 46 primary care departments in a large ambulatory care delivery system. We fielded 1,164 baseline and 1,333 follow-up surveys to physicians and non-physician staff after a system-wide implementation of Lean redesigns (average 73% response rate). The surveys assessed beliefs about Lean changes, perceptions of work environments, physician and staff engagement, and job-related burnout. We conducted multivariate regression to detect changes in work experiences as reported on baseline and follow-up surveys, adjusting for respondent characteristics and clustering of within-clinic responses.

**Findings:** Compared to baseline, respondents reported improved teamwork, more participation in clinic decisions, increased sense of personal motivation and work satisfaction, and less tendency to depersonalize patients after implementing Lean redesigns. However, respondents also perceived post-Lean clinic environments as busier and experienced greater emotional exhaustion in delivering patient care. More specifically, physicians reported experiencing a more stressful clinic and job-related burnout, although they also reported higher levels of ownership and commitment, teamwork, and participation in decision making. Non-physician experiences were similar, with some differences including lower burnout in the form of depersonalizing patients, and more engagement or personal motivation after redesigning workflows.

**Implications for D&I Research:** A central goal of Lean implementation is to empower and engage the workforce in daily work processes. This goal was realized, but redesigns did not appear to moderate long-standing challenges in primary care. To help practices cope with increasing burdens such as physician burnout, redesigns must create improvements that directly benefit care providers without overtaxing an already overstretched workforce. Program implementers may need to be particularly sensitive to these issues when planning new initiatives to facilitate care team workflows.

**Primary Funding Source:** Agency for Healthcare Research and Quality

### S156 US State Legislators’ attitudes towards mental health treatment effectiveness: stigma and implications for dissemination

#### Jonathan Purtle^1^, Felice Le-Scherban^1^, Xi Wang^1^, Paul Shattuck^2^, Enola Proctor^3^, Ross Brownson^3,4^

##### ^1^ Drexel University School of Public Health, Philadelphia, PA, USA; ^2^ A.J. Drexel Autism Institute, Drexel University, Philadelphia, PA, USA; ^3^ Washington University in St. Louis, St. Louis, MO, USA; ^4^ Alvin J. Siteman Cancer Center, Washington University School of Medicine, St. Louis, MO, USA

###### **Correspondence:** Jonathan Purtle (jpp46@drexel.edu)

**Background:** Policymakers are unlikely to implement policies that scale-up evidence-based mental health treatments if they do not perceive mental health treatments as effective. Targeted dissemination strategies can potentially improve policymakers’ attitudes towards treatment effectiveness, but formative research is needed to design these strategies. The study aimed to: 1) determine the prevalence and correlates of positive attitudes towards mental health treatment effectiveness among US state legislators, and 2) assess differences in dissemination preferences between legislators who do and do not perceive mental health treatments as effective.

**Methods:** A cross-sectional, multi-modal (telephone, post-mail, e-mail) survey of state legislators (N=268, response rate=18.0%) was conducted in winter-spring 2016. The dependent variable was strong agreement with the statement that “mental health treatments can help people with mental illness lead normal lives.” Independent variables were mental illness stigma (0-14 point scale), political party, gender, education, and history of mental health treatment seeking. Dissemination preferences were assessed by 14 items about preferred sources and features of mental health evidence. Multi-level (legislator, state), multivariable logistic regression bi-variate analyses were conducted.

**Findings:** Sixty-one percent of legislators strongly agreed that mental health treatments were effective. These legislators had significantly lower mental illness stigma scores than legislators who did not strongly agree that mental health treatments were effective (mean: 4.0 [SD=3.9] vs 7.1 [SD=3.4], p≤.001) and were significantly more likely to be Democrat (54.4% vs 30.1%, χ^2^=20.3, p≤.001), female (42.2% vs 21.5%, χ^2^=12.3, p≤.001), and report having sought mental health treatment (27.0% vs 13.3%, χ^2^=7.02, p=.008). After adjusting for legislator- and state-level covariates in logistic regression models, legislators in the highest stigma score quartile had 90% lower odds (aOR=.09, 95% CI=.03, .27) of strongly agreeing that mental health treatments are effective than legislators in the lowest stigma score quartile. Legislators who strongly agreed that mental health treatments were effective preferred receiving mental health evidence from advocacy organizations (57.8% vs 43.9%, χ^2^=4.93, p=.026) and universities (35.4% vs 17.8%, χ^2^=9.85, p=.002).

**Implications for D&I Research:** Dissemination strategies that spread information about evidence-based mental health treatments to state legislators are unlikely to be effective unless they also reduce stigma towards people with mental illness and communicate evidence through the preferred channels.

**Primary Funding Source:** National Institutes of Health

### S157 The integration quotient: use of claims data to assess implementation of integrated healthcare services

#### Michael S. Shafer^1^, George Runger^2^, William J. Riley^3^, Gevork Hartoonian^2^

##### ^1^ College of Public Service & Community Solutions, Arizona State University, Phoenix, AZ, USA; ^2^ Center for Health Information Research, Arizona State University, Phoenix, AZ, USA; ^3^ School for the Science of Health Care Delivery, Arizona State University, Phoenix, AZ, USA

###### **Correspondence:** Michael S. Shafer (michael.shafer@asu.edu)

**Background:** The implementation of the ACA has launched a number of innovations in healthcare delivery. Among those innovation has been a renewed emphasis on better integration of behavioral health services with primary care services. Unfortunately, few measures exist to assess the degree to which healthcare systems are implementing behavioral health integration.

**Methods:** Medicaid claims data for a large urban community in the southwest US was analyzed for a population of approximately 350,000 claimants. Using ICD and procedure codes, these claims were sorted into two categories of behavioral health procedures and primary care procedures. Individual patient *Integration Quotients* (IQ) were calculated as a ratio of behavioral health claim volume and value to primary care claim volume and value. Comparative analysis were conducted to evaluate the IQs among those individuals identified to be seriously mentally ill, experiencing general mental health and/or substance use disorders, with those individuals with no identifiable mental health or substance use disorder. Year over year comparisons were calculated to assess the impact which state Medicaid agency policies were having upon IQs, along with secondary analyses to identified patient and provider characteristics.

**Findings:** Claims data analysis reveal striking patterns distinguishing IQs by patient group (SMI, GMH/SA, non-BH) and provider. IQs were found to vary based upon total health care claim value and by provider type and group. Finally, time series analysis, demonstrated change in ratios over time for some patient groups (e.g., SMI), but not others, in alignment with recently enacted state medic policy directives.

**Implications for D&I Research:** These preliminary results suggest that use of claims data to assess the degree of healthcare integration that patients are experiencing may be a non-invasive, cost-effective method for assessing health care integration.

**Primary Funding Source:** The Robert Wood Johnson Foundation

### S158 A pragmatic trial testing mailed reminders with and without fecal immunochemical testing (FIT) to increase colorectal cancer screening in Medicaid populations

#### Jewels Rhode^1^, Alison Brenner^1,2^, Dana Baker^3^, Rebecca Drechsel^4^, Marcus Plescia^4^, Tom Wroth^5^, Daniel Reuland^1,2^, Stephanie Wheeler^1^

##### ^1^ Lineberger Comprehensive Cancer Center, University of North Carolina at Chapel Hill, Chapel Hill, NC, USA; ^2^ University of North Carolina at Chapel Hill, Chapel Hill, NC, USA; ^3^ Community Care Partners of Greater Mecklenburg, Charlotte, NC, USA; ^4^ Mecklenburg County Public Health Department, Charlotte, NC, USA; ^5^ Community Care of North Carolina, Raleigh, NC, USA

###### **Correspondence:** Jewels Rhode (jrhode@email.unc.edu)

**Background:** Colorectal cancer (CRC) is the third most common cause of cancer death in the United States. Despite strong evidence that CRC screening is effective at reducing incidence and mortality from colorectal cancer, it is underutilized. FIT is highly effective, serves as a stand-alone screening strategy, and is both less expensive and requires less preparation than some other CRC screening alternatives. Although health insurance coverage is an important predictor of CRC screening completion, Medicaid insured populations traditionally have had lower CRC screening rates than other insured populations. Evidence suggests that mailed reminders may be an effective method to improve CRC screening rates among vulnerable populations. This study evaluates the comparative effectiveness of a mailed CRC screening reminder, with and without an included FIT kit, in North Carolina Medicaid beneficiaries.

**Methods:** In partnership with the Mecklenburg County Public Health Department and the NC Medicaid managed care program, Community Care of North Carolina (CCNC), a pragmatic randomized control trial was conducted among participants aged 52-64 living in Mecklenburg County, with no record of recent CRC screening, and no history of CRC or major mental illness. Participants were randomized to FIT group: a letter encouraging CRC screening, a FIT kit with instructions, and a return mailer; OR Reminder group: a letter encouraging CRC screening, instructions for obtaining a FIT kit. Negative FIT results were communicated by mail to recipients and their providers. Participants with abnormal results were contacted by CCNC patient navigators to ensure follow-up colonoscopy.

**Findings:** There were 1,371 participants included in the study (716 FIT; 655 Reminder). In the Reminder group 147 participants requested FITs. In the FIT group, 21%(151) returned FIT kits compared with 13%(85) in the Reminder group (difference 8%;4%,12% p<0.01). Two hundred and fifteen FITs were negative, 18 were positive, and four were invalid. To date, 8 participants with positive FITs completed follow-up colonoscopies.

**Implications for D&I Research:** Including FIT kits with a reminder letter resulted in higher CRC screening rates compared with a reminder letter alone Medicaid beneficiaries. These findings suggest that implementing system-level CRC screening programs that include mailed FIT from health departments with patient navigation for abnormals appears feasible and effective.

**Primary Funding Source:** University Cancer Research Fund

### S159 Effect of occupational policy on implementation of workplace sun safety and outdoor workers’ sun protection practices in a randomized trial

#### Barbara Walkosz^1^, David Buller^1^, Allan Wallis^2^, Mary Buller^1^, Peter Andersen^3^, Michael Scott^4^, Richard Meenan^5^, Gary Cutter^6^

##### ^1^ Klein Buendel, Inc., Golden, CO, USA; ^2^ School of Public Affairs, University of Colorado Denver, Denver, CO, USA; ^3^ School of Communication, San Diego State University, San Diego, CA, USA; ^4^ Mikonics, Inc., Santa Fe, NM, USA; ^5^ Center for Health Research, Kaiser Permanente Northwest, Portland, OR, USA; ^6^ University of Alabama at Birmingham, Birmingham, AL, USA

###### **Correspondence:** Barbara Walkosz (bwalkosz@kleinbuendel.com)

**Background:** Comprehensive approaches to occupational sun safety combine policy and education to reduce solar ultraviolet radiation exposure that causes skin cancer, the most common U.S. cancer costing $8.1 billion annually. Policy adoption is effective only if it stimulates implementation of workplace sun safety.

**Methods:** The *Sun Safe Workplaces* (*SSW*) intervention, based on principles of Diffusion of Innovations Theory, was tested in a randomized controlled trial (n=98 public employers in Colorado with outdoor workers in public works, public safety and/or parks and recreation). *SSW* advocated that senior managers adopt formal sun safety polices and provided training and printed/electronic messages for outdoor workers. After finding that *SSW* increased policy adoption at intervention worksites, participating public employers were contacted 2-years after the intervention to conduct surveys with line supervisors and outdoor workers on implementation of workplace sun safety and personal protection practices (M=68 public employers [69%] followed-up; n=365 line supervisors, n=1,555 outdoor workers).

**Findings:** Increased proportions of line supervisors reported employers had implemented worksite sun safety in the intervention group (free/reduce price sunscreen – intervention: M=0.89, control: M=0.64; F=8.83, p=0.003; employer communicated about sun safety – intervention: M=0.94, control: M=0.68; F=15.15, p<0.001) and in workplaces adopting a sun protection policy (line supervisor communicated about sun safety – policy: M=0.84, no policy: M=0.68; F=7.86, p=0.005). Likewise, more outdoor workers recalled receiving sun safety messages in the intervention group (written message – intervention: M=0.70, control: M=0.49; F=13.94, p<0.001; information from coworker/supervisor – intervention: M=0.68, control: M=0.55; F=6.15, p=0.013; training – intervention: M=0.69, control: M=0.50; F=6.82, p=0.009) and in workplaces adopting a policy (information from coworker/supervisor – policy: M=0.68, no policy: M=0.56; F=4.39, p=0.036). Outdoor workers’ sun protection practices also improved in the intervention group (combined sun safety practices – intervention: M=3.47, control: M=3.33; F=10.44, p=0.002; proportion sunburned on the job – intervention: M=0.37, control M=0.45; F=3.90, p=0.048) and in workplaces adopting a policy (combined sun safety practices – policy: M=3.45, no policy: M=3.34; F=5.50, p=0.023).

**Implications for D&I Research:** Promoting policy adoption improved implementation of worksite sun safety and sun protection practices of employees. Formalized policies should result in sustained implementation of skin cancer prevention.

**Primary Funding Source:** National Institutes of Health

### S160 From employee education to organizational policy adoption: building a pathway for sustained implementation of an occupational sun protection program

#### David Buller^1^, Barbara Walkosz^1^, Peter Andersen^2^, Michael Scott^3^, Gary Cutter^4^

##### ^1^ Klein Buendel, Inc., Golden, CO, USA; ^2^ School of Communication, San Diego State University, San Diego, CA, USA; ^3^ Research, Mikonics, Inc., Santa Fe, NM, USA; ^4^ University of Alabama at Birmingham, Birmingham, AL, USA

###### **Correspondence:** David Buller (dbuller@kleinbuendel.com)

**Background:** Sustained prevention programs are essential to achieve long-term health benefits and return on investment of national and local resources. Sun protection of outdoor workers is important to reduce their exposure to ultraviolet radiation and prevent skin cancer, the most common U.S. cancer costing $8.1 billion annually. Guided by Diffusion of Innovation Theory (DIT), our team has conducted a series of randomized controlled trials (RCTs) on a pathway to achieving sustained implementation of occupational sun protection.

**Methods:** The research started with the testing of *Go Sun Smart (GSS)* in an RCT with North American ski areas (n=28). *GSS* was a workplace intervention comprised of training and promotional materials aimed at improving personal sun protection of outdoor workers. A second RCT (n=68 ski areas) evaluated standard industry methods for disseminating *GSS* in comparison to DIT-based strategies. *Sun Safe Workplaces (SSW)*, a comprehensive intervention combining worksite policy adoption with *GSS*’ employee education, was subsequently tested in an RCT with public employers in Colorado (n=98). Theoretically, policy should sustain implementation by clarifying need and responsibility for sun safety and countering management turnover.

**Findings:** *GSS* significantly reduced sunburns (p<0.05) and increased sun protection (p<0.05) of employees at intervention worksites compared to controls. DIT-based strategies produced greater program implementation (p<0.01) and employee sun safety than industry dissemination methods (i.e., recall of *GSS* message was greatest at ski areas receiving DIT-based strategies [p=0.001] and message recall was related to greater sun protection [p<.001]). In a follow-up assessment 5-7 years after dissemination, employees continued to report greater sun protection at employers implementing 9 or more sun safety messages (p=0.037); however, turnover in management was a barrier to sustained implementation (p<0.001). *SSW* successfully promoted policy adoption: 51.2% of intervention employers had a sun safety policy compared to 32.6% of controls (p<0.01). Intervention managers reported greater implementation of workplace sun safety than control managers (e.g., provision of wide-brimmed hats [p<0.05]).

**Implications for D&I Research:** Successful strategies for program implementation that utilized policy and education for occupational sun protection were developed based on DIT principles. The next step is to identify cost-effective methods for scaling up the SSW intervention nationwide.

**Primary Funding Source:** National Institutes of Health

### S161 Implementation theory extended to self-implemented "programs": self-implemented HIV testing

#### Joseph Catania^1^, M. Margaret Dolcini^2^, Gary Harper^3^, Ryan Singh^1^

##### ^1^ College of Public Health and Human Sciences, Oregon State University, Corvallis, OR, USA; ^2^ School of Social and Behavioral Health Sciences, Oregon State University, Corvallis, OR, USA; ^3^ University of Michigan School of Public Health, Ann Arbor, MI, USA

###### **Correspondence:** Joseph Catania (catania1951@comcast.net)

**Background:** We extend implementation models developed by Fixen and colleagues to encompass self-implemented public health programs (e.g., Malaria “net” prevention, pregnancy self-testing, water-jar purification). For instance, self-implemented programs typically contain training and core components that must be implemented with fidelity to produce outcomes, and may require adaptions to optimize fit between components and the individual. A primary difference from organization-based formulations is that the client is both the implementer and recipient of program training.

**Methods:** We examined self-implementation fidelity and proximal antecedents within an effectiveness study of OraSure’s oral HIV self-test (Oral-SIT) for African American men-who-have-sex-with-men (AAMSM; 17-24 yrs.) [N = 181]. AAMSM have the highest rate of HIV infection and are the most likely to be undiagnosed. OraSure’s kit includes written and video training components. Eligible participants were either HIV negative or HIV status unknown, with no history of Oral-SIT self-implementation (37% < high school education).

**Findings:** Eleven-percent had low fidelity (≥ 2 errors among 7 core components; behavioral observations). The most common error was incorrectly recording incubation start/end times (56%). Using logistic regression, we examined two models a) AAMSM who made ≥ 2 errors vs. none/one, and b) AAMSM who made “timing” errors (vs. no timing errors). Independent variables included knowledge acquired from training materials, and person variables hypothesized to impact performance (e.g., social stigma, education, HIV-testing experiences). AAMSM who made numerous errors differed in terms of a) a greater proportion made timing errors (i.e., incorrectly recorded specimen incubation period, prematurely removed the specimen swab from incubation) (p = .04), and b) had significantly (p = .03) less prior exposure to oral testing (i.e., by others). AAMSM committing timing errors were significantly less likely to have acquired correct timing information from the trainings (p = .001) and were more likely to report high levels of social stigmatization (p = .005). Education levels were unrelated to either outcome.

**Implications for D&I Research:** The results indicate that the training component needs improving to reduce timing problems and social stigma concerns. Further, public health education that exposes men to oral testing kits may facilitate self-implementation fidelity. Implications for a theory of self-implementation will be discussed.

**Primary Funding Source:** National Institutes of Health

### S162 Integrating HIV care into an opioid treatment program in Tanzania: the IMAT program

#### Saria Hassan^1,2^, Alexis Cooke^3^, Haneefa Saleem^4^, Dorothy Mushi^5^, Jessie Mbwambo^4^, Barrot Lambdin^2^

##### ^1^ Yale School of Medicine, New Haven, CT, USA; ^2^ RTI International, San Francisco, CA, USA; ^3^ University of California, Los Angeles, Los Angeles, CA, USA; ^4^ Johns Hopkins Bloomberg School of Public Health, Baltimore, MD, USA; ^5^ Muhimbili University of Health and Allied Sciences, Dar Es Salaam, Tanzania

###### **Correspondence:** Saria Hassan (saria.hassan@yale.edu)

**Background:** The prevalence of HIV among people who inject opioids (PWIO) in Dar-Es-Salaam, Tanzania is 42%, compared to 7% in the general population. Despite successful rates of PWIO enrollment in an opioid treatment program (OTP), rates of HIV+ OTP clients initiating anti-retroviral therapy (ART) was low at 33% in 90-days. This ART initiation gap is a missed opportunity to curb further spread of HIV. To address this, we engaged the community of OTP patients and providers to design an Integrated Methadone and Antiretroviral Therapy (IMAT) program – integrating HIV care into the OTP clinic at Muhimbili National Hospital, Dar-Es-Salaam*.*

**Methods:** Formative mixed methods research, described elsewhere, informed the design and implementation strategy of IMAT. OTP providers (3 nurses, 3 clinicians) were trained in comprehensive HIV care and treatment. Newly diagnosed HIV+ OTP clients and those not on ART received counseling and were prescribed ART. Those on ART received CD4/viral load testing and counseling. Clients were seen by the provider at enrollment, initiation or 3-months maintenance visits. We evaluate IMAT implementation using programmatic and qualitative data applied to 3 dimensions of the RE-AIM framework.

**Findings:** *Reach:* 98% of HIV+ clients engaged in IMAT. *Effectiveness:* 90-day ART initiation rate for newly diagnosed HIV clients increased 65% (95% CI: 40-85%; p<0.001) from 33% to 98%; proportion of HIV+ clients on ART increased 38% (95% CI: 2-88%; p<0.001) from 71% pre-IMAT to 97% post IMAT. *Adoption:* all trained nurses and clinicians participated fully in IMAT. 77% of HIV+ clients received comprehensive HIV care through IMAT including ART at the OTP clinic; 21% of HIV+ clients received HIV counseling and monitoring services but ART at an outside clinic. In-depth interviews with clients and providers 6-months post-implementation showed overall satisfaction with IMAT and the implementation process but concerns around limited resources.

**Implications for D&I Research:** We successfully integrated HIV care into an OTP clinic in Tanzania with increased rates of ART initiation. We specify the implementation process using guidance from Proctor *et. al* (2013) enabling replication in other settings. Future work will evaluate remaining RE-AIM components of implementation and maintenance, address resource limitations and consider scale up to other OTP clinics in the region.

**Primary Funding Source:** National Institutes of Health

### S163 Use of social network analysis to inform the design, dissemination, implementation, and sustainability of health behavior interventions: findings from a systematic review

#### Matthew Lee^1^, Elizabeth Gage-Bouchard^2^, Danielle M. Crookes^1^, Lina H. Jandorf^3^, Deborah Erwin^2^, Rachel Shelton^1^

##### ^1^ Columbia University Mailman School of Public Health, New York, NY, USA; ^2^ Roswell Park Cancer Institute, Buffalo, NY, USA; ^3^ Icahn School of Medicine at Mount Sinai, New York, NY, USA

###### **Correspondence:** Matthew Lee (ml3440@cumc.columbia.edu)

**Background:** Social network analysis (SNA) is an innovative methodology with the potential to inform the design, dissemination, implementation, and sustainability of public health prevention and behavioral interventions. Using SNA may also advance understanding of the processes through which interventions are effective.

**Methods:** We conducted a systematic review of 15,599 articles published from 2004 through 2016 in order to better understand how researchers are applying SNA to inform intervention research. To be eligible, articles had to focus on adults, include SNA, and discuss how they were (or could be) used to inform the design, dissemination, implementation and sustainability of a health behavior intervention.

**Findings:** We identified 45 articles that met these criteria, representing a range of health behaviors and interventions across institutional, health-care, and community settings. The two most common health topics represented were sexual health, particularly HIV prevention (n=12 articles), and smoking cessation (n=7 articles). Most of the articles (n=32) were published from 2013-2016. Most articles (N=28) described how to use SNA findings to inform potential behavioral interventions, but did not apply those findings to either a new or existing intervention. Several articles (N=16) applied SNA findings to either a new or existing intervention. Only one article accomplished both. Describing the use of SNA findings to inform intervention *design* was most common (n=38), followed by *dissemination* (n=7), *implementation* (n=6), and *sustainability* (n=6). The majority of studies were conducted in a US context (n=24) and 11 were conducted using online or internet-mediated networks. The majority did not define SNA (n=33), and only four mentioned social network theory. The most common health behavior theories applied were Social Support Theory (n=6) and Diffusion of Innovations (n=5). Over 50 different network measures were reported, with network size (n=20), density (n=11), and degree centrality (n=10) appearing most frequently. Additional findings regarding study design, network types, and theory application will be discussed, as well as examples of how SNA could more optimally be used to inform dissemination, implementation, and sustainability.

**Implications for D&I Research:** This review reveals how advancements in SNA and social network theory can be applied to enhance interventions, and discusses opportunities for improving the application of SNA in implementation science.

**Primary Funding Source:** Dr. Shelton is funded through a Mentored Research Scholar Grant at The American Cancer Society (124793-MRSG-13-152-01-CPPB)

### S164 Assessing implementation of tobacco treatment into cancer care: feasibility, treatment fidelity, and acceptability

#### Elyse Park^1^, Giselle Perez^1^, Susan Regan^1^, Alona Muzikansky^1^, Sarah Borderud^2^, Julia Rabin^1^, Emily Friedman^1^, Nancy Rigotti^1^, Jamie Ostroff^2^

##### ^1^ Massachusetts General Hospital, Boston, MA, USA; ^2^ Memorial Sloan Kettering Cancer Center, New York, NY, USA

###### **Correspondence:** Elyse Park (epark@mgh.harvard.edu)

**Background**: Several leading national oncology organizations (NCCN, ASCO) recommend smoking cessation as part of comprehensive cancer care, yet implementation of evidence-based tobacco treatment into the care of cancer patients who are smokers has not been established.

**Methods:** We conducted a multisite effectiveness-implementation trial assessing the integration of two evidence-based tobacco treatments into the care of recently diagnosed cancer patients. The standard care (SC) group received 4 telephone-based counseling sessions and education about cessation medications; the intensive care (IC) group received 7 additional counseling sessions and FDA approved smoking cessation medication. 303 patients from 8 cancer sites were randomized between 11/13-07/17 (mean age = 58.3, sd= 9.7; 83.1% white, 56.1% female). We sought to determine 1) reach of eligible patients, 2) fidelity of cessation treatment delivery, and 3) treatment acceptability.

**Findings:** 2948 adult smokers (any cigarette use in the past month) who were recently diagnosed and receiving cancer treatment at the study institutions were identified. 2016 (68.3%) refused; the most common reason for refusal was preferred self-quitting. Among the 446 confirmed eligible patients, 67.9% enrolled. To date, 167 have completed the final study period; 6-month follow-up survey response rate = 86.0% (5.5% died). 76.5% in the SC group completed ≥3/4 sessions and 64.6% in the IC group completed ≥7/11 sessions. 79% in the IC group accepted smoking cessation medications; combination nicotine replacement therapy (patch + lozenge) was the most common medication of choice. Acceptability ratings were collected at 3-month follow-up. 85.9% of the IC group reported most/all of their needs were met vs 61.6% of the SC group (p<.001). 60.4% of the IC group vs. 27.4% of the SC group (p<.001) reported they definitely received the kind of smoking cessation assistance needed. Lastly, 75.6% of IC group vs. 45.8% of the SC group (p<.001) reported they would definitely recommend the treatment if a friend were in need of similar help.

**Implications for D&I Research:** Implementing evidence-based tobacco treatment into the care of cancer patients at the beginning of their treatment is challenging. Treatment fidelity was relatively high for both counseling and cessation medication. Moreover, patients reported high acceptability and treatment satisfaction.

**Primary Funding Source:** National Institutes of Health

